# Orthogonal IMiD-Degron Pairs Induce Selective Protein Degradation in Cells

**DOI:** 10.1021/acschembio.5c00751

**Published:** 2025-11-02

**Authors:** Patrick J. Brennan, Rebecca E. Saunders, Mary Spanou, Sarah E. Singleton, Marta Serafini, Liang Sun, Guillaume P. Heger, Agnieszka Konopacka, Ryan D. Beveridge, C. Cameron Taylor, Peter DePaola, Laurie Gordon, Shenaz B. Bunally, Aurore Saudemont, Andrew B. Benowitz, Carlos Martinez-Fleites, Danielle L. Schmitt, Robert Damoiseaux, Markus A. Queisser, Heeseon An, Charlotte M. Deane, Michael M. Hann, Lewis L. Brayshaw, Stuart J. Conway

**Affiliations:** † Department of Chemistry, Chemistry Research Laboratory, 6396 University of Oxford, OX1 3TA Oxford, U.K.; ‡ Department of Chemistry & Biochemistry, 8783 University of California Los Angeles, Los Angeles, 90095 California, United States; § GSK, Medicines Research Centre, SG1 2NY Stevenage, U.K.; ∥ PerkinElmer, HP9 2FX Beaconsfield, U.K.; ⊥ Chemical Biology Program, 5803 Memorial Sloan Kettering Cancer Center, 10065-6007 New York, United States; # Virus Screening Facility, Weatherall Institute of Molecular Medicine, University of Oxford, OX3 9DS Oxford, U.K.; ∇ California NanoSystems Institute, University of California Los Angeles, Los Angeles, 90095 California, United States; ○ Molecular Biology Institute, University of California Los Angeles, Los Angeles, 90095 California, United States; ◆ Institute for Quantitative and Computational Biosciences, University of California Los Angeles, Los Angeles, 90095 California, United States; ¶ Department of Molecular and Medical Pharmacology, University of California Los Angeles, Los Angeles, 90095 California, United States; †† Department of Bioengineering, University of California Los Angeles, Los Angeles, 90095 California, United States; ‡‡ Jonsson Comprehensive Cancer Center, University of California Los Angeles, Los Angeles, 90095 California, United States; §§ Department of Statistics, University of Oxford, OX1 3LB Oxford, U.K.

## Abstract

Immunomodulatory imide drugs (IMiDs), including thalidomide, lenalidomide, and pomalidomide, can be used to induce degradation of a protein of interest that is fused to a short degron motif, which often comprises a zinc finger (ZF). These IMiDs, however, also induce the degradation of endogenous ZF-containing neosubstrates, including IKZF1, IKZF3, and SALL4. To improve degradation selectivity, we took a bump-and-hole approach to design and screen bumped IMiD analogues against 8380 ZF mutants. This yielded a bumped IMiD analogue that induces efficient degradation of a mutant ZF degron, while not affecting other cellular proteins, including IKZF1, IKZF3, and SALL4. In proof-of-concept studies, this system was applied to induce degradation of the optimum degron fused to CDK9, HPRT1, NanoLuc, or TRIM28. We anticipate that this system will be a valuable addition to the current arsenal of degron systems for use in target validation.

## Graphical abstract

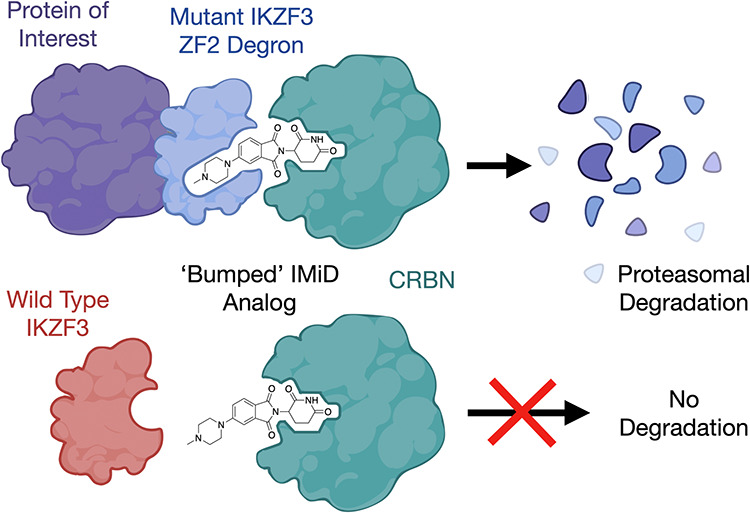

## Introduction

Chemically induced degradation of proteins is a powerful complement to traditional occupancy-based small-molecule modulation of a biological target. This strategy allows all functions of a protein to be neutralized, including scaffolding roles and the action of undruggable domains that would otherwise be difficult to inhibit. Within this approach, two distinct small-molecule-based strategies have been developed: proteolysis targeting chimeras (PROTACs) and molecular glues.^1^ PROTACs are bifunctional molecules that induce proximity between the target protein of interest (POI) and an E3 ligase to promote POI degradation.^1,2^ Alternatively, molecular glues are small molecules that bind at the interface of two proteins, enhancing or inducing their interaction.^3,4^ Molecules that function as molecular glues include the natural products auxin, cyclosporin, FK506, and rapamycin and synthetic stabilizers of the hub protein 14–3–3.^5^ Immunomodulatory imide drugs (IMiDs) are a well-studied family of molecular glues that includes thalidomide, lenalidomide, pomalidomide, and CC-220.^6–8^ IMiDs possess a glutarimide ring that binds to a tritryptophan pocket in cereblon (CRBN), a substrate adaptor of the Cullin4 RING E3 ligase (CRL4^CRBN^).^9,10^ Binding of the IMiD remodels the CRL4^CRBN^ protein surface, inducing an affinity between the CRBN-IMiD binary complex and a series of β-hairpin-containing proteins, known as neosubstrates. Subsequent ubiquitination by the CRL4^CRBN^ machinery results in proteasomal degradation of the neosubstrate. Recent work by Heim et al. and Ichikawa et al. has shown that the true endogenous targets of CRBN are proteins that possess similar cyclic imides as a C-terminal post-translational modification (PTM), formed by intramolecular cyclization of glutamine or asparagine residues.^11,12^

At least ten CRL4^CRBN^ neosubstrates are known, including Ikaros family zinc finger protein 1 (IKZF1; Ikaros) and Ikaros family zinc finger protein 3 (IKZF3; Aiolos), ZFP91 zinc finger protein (ZFP91), G1 to S phase transition 1 protein (GSPT1), casein kinase 1 α 1 (CK1α), and spalt-like transcription factor 4 (SALL4); the latter of which is thought to be the effector of the teratogenic properties of thalidomide.^13–23^ Although these neosubstrates do not possess a specific consensus sequence, they all share a β-hairpin motif with a conserved glycine residue, which binds at the CRBN-IMiD interface,^6,24^ and is frequently part of a C2H2 zinc finger (ZF). This motif is an example of a degron, broadly defined as a targeting signal that confers metabolic instability on some, or all, of the peptide bonds in a protein.^25–27^ This definition encompasses inducible domains, which require the presence of a small molecule to promote protein degradation. Genetic knock-in methods enable a degron to be used in a complementary manner to PROTACs, allowing induced degradation of POIs that lack a small-molecule ligand.^25,28^ A number of these technologies are well established, including the auxin inducible degron system (AID), small molecule-assisted shutoff (SMASh-tag), a destabilizing domain (DD) stabilized by small molecule Shld1, a degron based on methyl guanine methyltransferase (MGMT), and systems that utilize bifunctional small molecules, including dTAG (degron = 11.9 kDa), HaloPROTAC (degron = 33.6 kDa), Bromotag (degron = 14.9 kDa), and an approach based on NanoLuciferase.^28–37^ While these techniques have been elegantly applied in numerous studies, a potential drawback is the use of large degron motifs, which can negatively affect the feasibility of CRISPR knock-in and the function of the POI.^25,34^ Recent work by Tsang et al. offers a potential solution to this using HiBiT-SpyTag and SpyCatcher to add a degron motif to a POI.^38^ However, while this approach does allow minimal alteration of the POI, it also requires further transfection steps.^39–41^

A combination of IMiD small molecules and ZF-based degrons offers an attractive alternative as inducible degrons; the low molecular weight inducers employed can readily enter cells and do not display a hook effect. In addition, the ZF degron motif can be as small as 23 amino acid residues, but works optimally at 60 amino acids,^6,24^ minimally perturbing the POI (degron = 7.0 kDa). Existing implementations of such systems have used degron motifs derived from IKZF1/3, SALL4, and hybrid sequences comprising halves of two different neosubstrate ZF degron sequences, such as Superdegron and iTAG.^24,42–45^ These IMiD-degron systems have been used to degrade chimeric antigen receptors (CARs) in engineered T-cells, and modulate CRISPR Cas9 genetic editing.^44–47^ While this elegant approach overcomes some limitations of other inducible devices, the use of existing small-molecule IMiDs presents important selectivity-related limitations. Sievers et al. identified 11 zinc finger-containing transcription factors that were degraded in the presence of thalidomide, lenalidomide, or pomalidomide.^6^ Computational analysis suggested that more than 150 zinc fingers could bind to the drug-CRBN complex. Therefore, the use of IMiDs that can bind to the wild-type (WT) ZF degrons in CAR T-cell therapy or target validation risks degradation of additional neosubstrates, leading to unwanted effects.

To overcome the above limitations, we employed a bump-and-hole approach to develop IMiD-degron pairs with orthogonality to the existing IMiD-WT ZF combinations (Figure 1A,B). Our approach involved making a mutant protein with a “hole” that can accommodate a “bumped” ligand that can bind to this mutant, but not the original wild-type (WT) protein. This approach was pioneered by Schreiber using FKBP12 and cyclophilin, and Shokat, who applied this approach to generating selective kinase-ligand pairs.^48–51^ More recently, Ciulli and Fischer have independently published elegant applications of the “bump-and-hole” approach to distinguish between the structurally similar first and second bromodomains of BRD4.^28,32,52^

**Figure 1:**
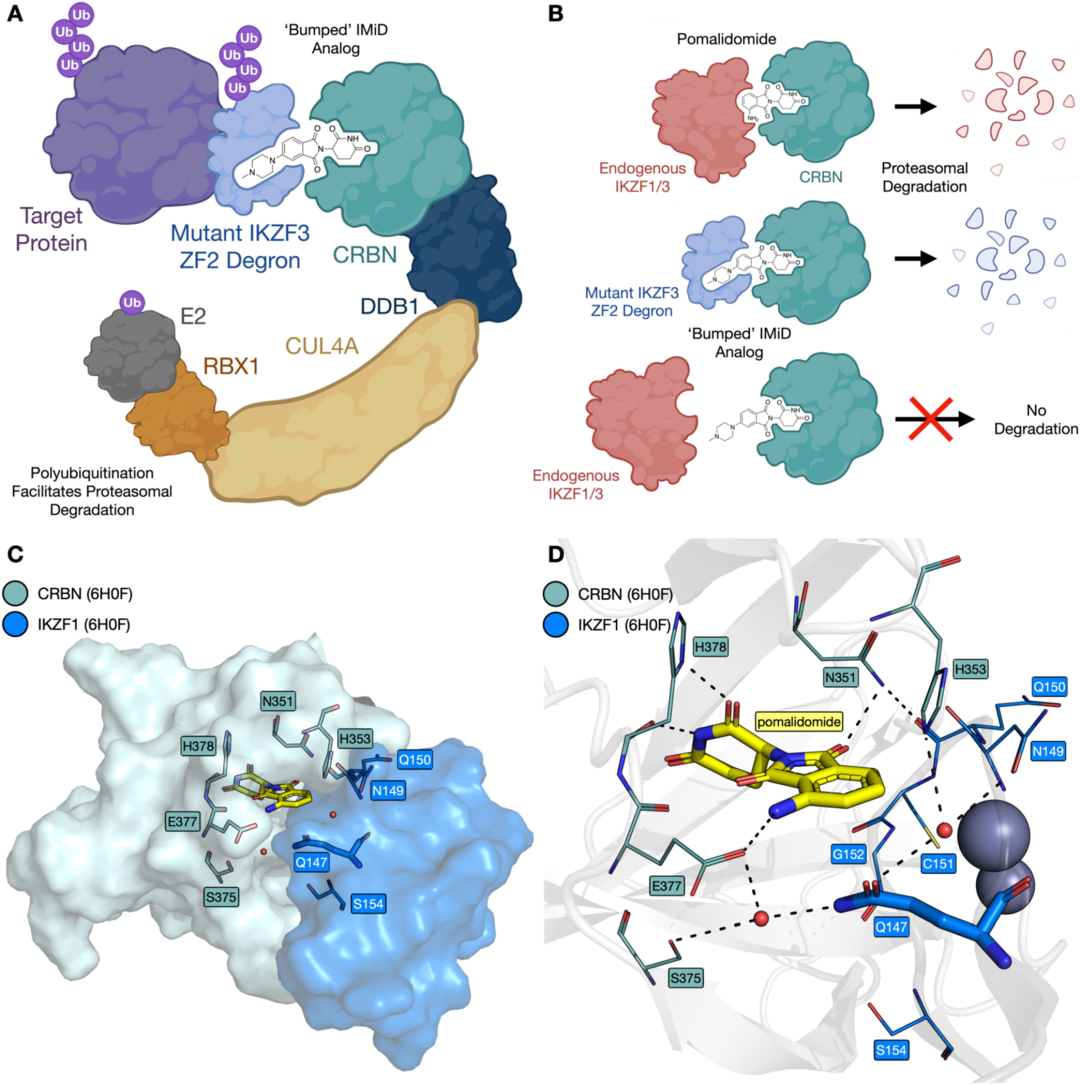
Schematic depicting the mechanism of action for the bumped IMiD-degron system. (A) The IMiD molecule binds to CRBN, and the resulting binary complex binds to the mutant IKZF3 ZF2-derived degron. This brings the degron-tagged target protein into proximity of the CRBN-recruited ubiquitination machinery, effecting proteasomal degradation. Ub, ubiquitin. (B) The bump-and-hole strategy: traditional IMiDs such as pomalidomide induce degradation of neosubstrates such as IKZF1 and IKZF3; a bumped IMiD analogue will recruit a degron with a suitable “hole” mutation but will ignore endogenous neosubstrates. (C) Structural details of the CRBN-IKZF1 ZF2 interface. Key residues at the interface between CRBN (teal) and IKZF1 ZF2 (blue) are with pomalidomide (carbon = yellow) and two structured water molecules (red spheres) bound at the interface (PDB ID: 6H0F). (D) Key predicted hydrogen bonds (dashed lines) formed between pomalidomide, two structured water molecules (red spheres), CRBN (carbon = teal), and IKZF1 ZF2 (carbon = blue; PDB ID: 6H0F).^6^

Here, we designed a series of bumped IMiD derivatives and selected compounds that showed only modest degradation of the WT IKZF3-derived degron attached to the enhanced green fluorescent protein (EGFP). We then designed a library of 8380 ZF mutants, using a similar approach to Sievers et al. and Jan et al.,^6,44^ and screened this against the bumped IMiDs. This approach resulted in the discovery of an IMiD analogue mutant ZF degron pairing that efficiently degrades a tagged POI. Proteomics studies revealed this pairing has exquisite selectivity over other cellular proteins, including known CRL4^CRBN^ neosubstrates. During the course of this work, Mercer et al. used a complementary, phage-assisted continuous evolution (PACE) platform to also generate IMiD-degron pairs with orthogonality to the existing IMiD-WT ZF combinations.^53^

## Results and Discussion

### Bumped IMiD Analogues Degrade the Q147A Mutant of the IKZF3-EGFP Fusion

Initial work focused on the identification of appropriate ZF residues to mutate, based on the crystal structure of pomalidomide bound to CRBN and the IKZF1 ZF (PDB ID: 6H0F, Figure 2).^6^ IKZF3 residues Q147, N149, Q150, C151, and G152 form the interface with pomalidomide-bound CRBN (Figure 1C; IKZF1 and IKZF3 possess identical ZF2 sequences with numbering schemes that differ by one residue; for consistency, IKZF3 ZF2 numbering is used here). While residues N149, Q150, and C151 are proximal to pomalidomide, their backbones rather than their side chains are oriented toward pomalidomide, again making them unsuitable candidates for mutation in a bump-and-hole approach. The conserved G152 residue is also close to pomalidomide, but cannot be mutated to an amino acid with a smaller side chain. We, therefore, decided to focus on a Q147A mutation (Figure 1D). This residue is located close to pomalidomide and is a key component of the CRBN-IKZF1 interface, forming a water-mediated hydrogen bond with E378 from CRBN.

**Figure 2:**
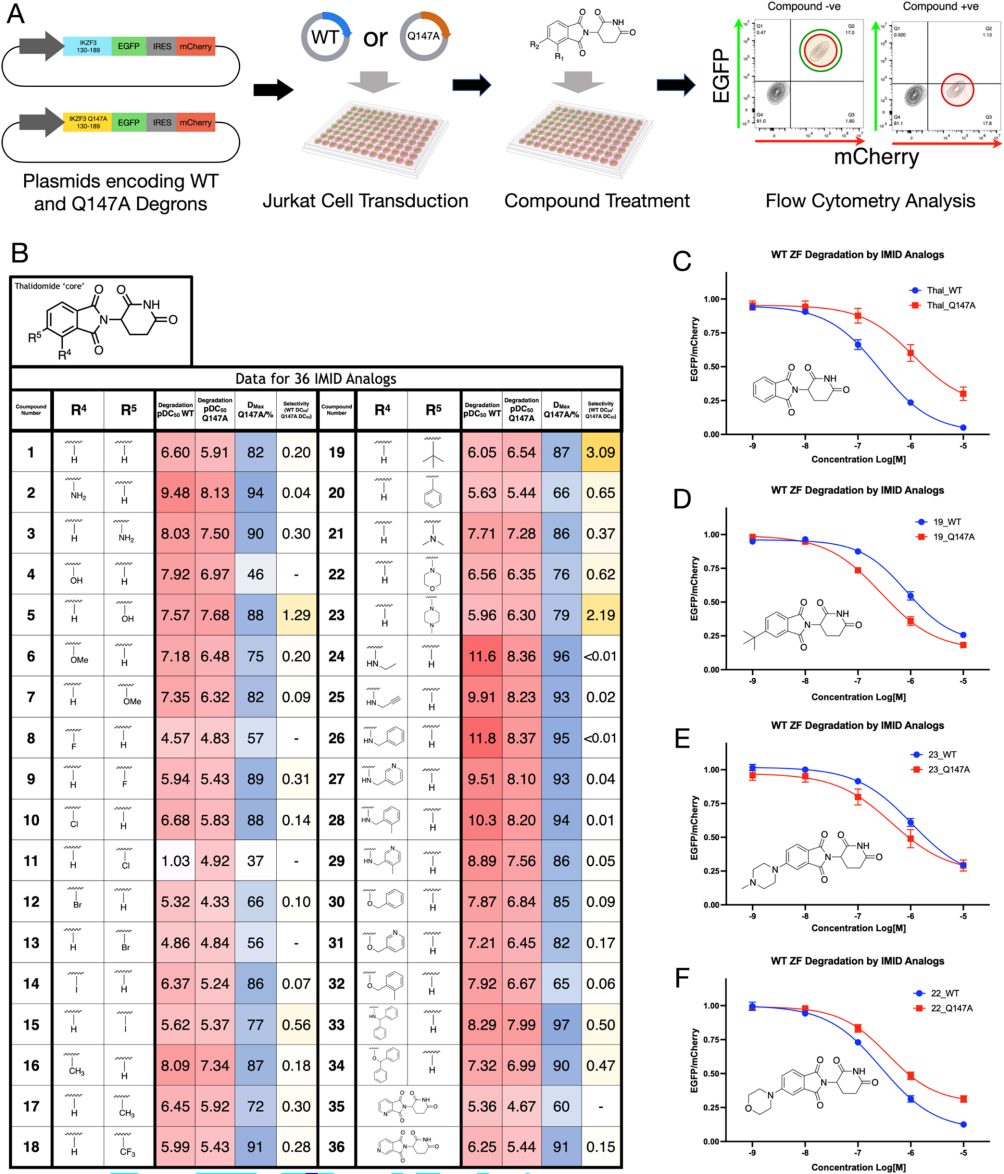
Degradation studies on the Q147A mutant. (A) Schematic for the ratiometric flow cytometry assay used to assess degradation properties of 36 IMiD analogues against the WT IKZF3-derived degron and a Q147A mutant degron (18 h incubation time). (B) Table showing pDC_50_ (WT), pDC_50_ (Q147A), *D*_max_ (Q147A), and selectivity values for 36 IMiD analogues. Selectivity value = DC_50_(WT)/DC_50_(Q147A). (C–F). Jurkat cells stably expressing either WT or Q147A degrons fused to EGFP were treated with a concentration range of thalidomide (C), compound **19** (D), compound **23** (E), or compound **22** (F) to yield degradation curves. *n* = 3.

Next, we designed and synthesized a set of 36 IMiD analogues. These compounds are modified at either the 4- or 5-position and were intended to probe the degradation structure–activity relationships (SAR) in this region of the molecule (Figures 2 and S1). We assessed the ability of each of these compounds to induce degradation of the mutant degrons using a ratiometric fluorescence-based assay.^6^ The degron spans residues 130–189 of IKZF3, which incorporate the minimal degron ZF2 (residues 146–168) and regions of the flanking ZFs on either side. The degron was fused to EGFP upstream from a red fluorescent protein (mCherry) separated by an internal ribosomal entry site (IRES). This construct was expressed in Jurkat cells; the cells were incubated with a given compound for 18 h and then analyzed using flow cytometry. EGFP:mCherry ratios from treated cell populations were then normalized against the EGFP:mCherry ratio from an untreated cell population to determine the level of compound-induced degradation. A reduction in EGFP, relative to mCherry, indicates that degradation is being induced (Figure 2A).

pDC_50_ values (−log_10_DC_50_, where DC_50_ = the compound concentration that induces half-maximal protein degradation) obtained for both the WT and Q147A mutant were compared for every compound exhibiting a Q147A *D*_max_ of >60% (*D*_max_ is the maximum level of degradation observed). This analysis revealed that the 4-position-modified IMiD analogues generally induced higher degradation of the WT degron compared to thalidomide. However, most of the 5-position-modified analogues were less potent degraders of the WT degron, compared to thalidomide, which is the directionality of change that we required (Figure 2B). Of particular interest were the 5-hydroxy (**5**), 5-*tert*-butyl (**19**; Figure 2D), and 5-*N*-methylpiperazine (**23**; Figure 2E) analogues, which all induced greater degradation of the Q147A-containing degron, compared to the WT degron. It is notable that compound **22**, which possesses a similarly sized ‘bump’ group to compounds **19** and **23**, preferentially degraded the WT degron over the Q147A mutant (Figure 2F). This indicates that protein–ligand interactions beyond simple steric bulk could be important for inducing the selective degradation observed. As 5-hydroxythalidomide (**5**) is a known degrader of SALL4, our studies focused on **19** and **23**.^17^

This initial work served as a proof-of-concept that a bump-and-hole approach could be applied to the CRBN-IMiD-ZF system. Importantly, compounds **19** and **23** showed only moderate degradation of the WT degron, with pDC_50_ values of 6.05 and 5.96, respectively, compared to 6.60 for thalidomide and 9.48 for pomalidomide. Despite this, the selectivity gained from a single Q147A mutation was modest. We therefore decided to explore further ZF mutations at a wider range of positions with the aim of identifying more selective IMiD-degron pairs.

### Design of a Mutant Library Identifies 8380 Mutants for Evaluation

Inspection of the X-ray crystal structure of pomalidomide bound to CRBN and IKZF1 (PDB ID: 6H0F) shows that, in addition to Q147, residues N149, Q150, G152, A153, S154, F155, and L167 form important components of the IKZF1 protein interface with CRBN. The side chain of residue S154 is proximal to that of Q147. Residues N149, Q150, A153, and L167 interface directly with CRBN; residue A153 also forms part of a three-residue motif with residues F146 and F155, providing a “core” to the ZF structure. Residue G152 is notable as being common to all IMiD-binding ZF degrons.^44^ The full library design is summarized in Figure 3A.

**Figure 3:**
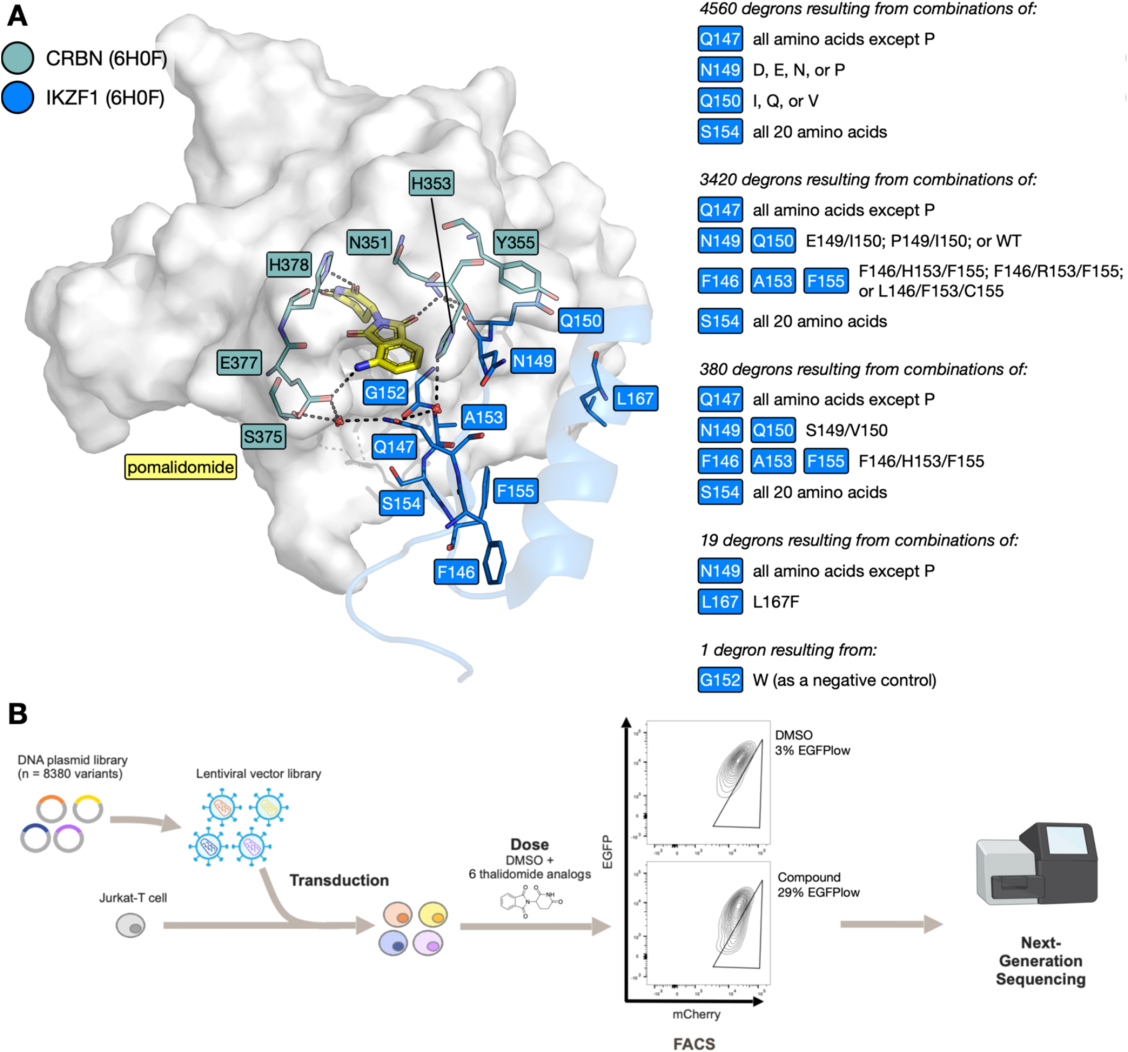
Design of the mutant library and the library screening workflow. (A) Key residues at the interface between CRBN (gray surface; teal sticks) and IKZF1 ZF2 (blue cartoon and sticks) with bridging pomalidomide (yellow sticks) and waters (red spheres) (PDB ID: 6H0F);^6^ key interactions shown as dashed lines. (i–v) Description of mutations comprising the mutant ZF library. (B) Schematic for the ZF degron library screen: plasmids encoding 8380 mutant ZFs were transduced into a population of Jurkat cells, cells were treated with one of 6 compounds—**1** (thalidomide), **19**, **22**, **23**, **33** or lenalidomide—or left untreated (DMSO control) for 18 h, then sorted by FACS into EGFP_+_ and EGFP_low_ populations; Next-generation sequencing (NGS) was then performed on both populations.

Exploring all possible combinations of mutations at these nine positions would result in 512 billion combinations and was therefore impractical, so steps were taken to narrow down the number of mutants explored. Given our focus on position 147 we decided to explore all possible mutations; however, proline was not included as the backbone angles observed for position 147 in the X-ray crystal structure (Φ of −111.3°, Ψ of 148.4°) did not fall within the acceptable ranges for proline (Φ angle ≈ −60°; Ψ angle ≈ −45° or 135°). Similarly, position 154 was significant enough for all 20 proteinogenic amino acids to be included at this position.

To determine the ability of mutations at residues 149, 150, 153, and 167 to help stabilize the CRBN-ZF protein–protein interaction (PPI), Rosetta and FoldX software were employed to perform an *in silico* screen using a region of the CRBN-IMiD-ZF complex crystal structure (PDB ID: 6H0F)^6^ in the absence of the IMiD (Figure S4).^54,55^ These data, and inspection of known endogenous neosubstrate sequences, predicted that residues D, E, N, or P at position 149, and I, Q, or V at position 150 (Figure 3Ai) might help to stabilize the PPI. Using this approach, no stabilizing mutations were predicted at positions 153 and 167. However, mutations of residue 153 were considered in combination with mutations at positions 146 and 155. IKZF3 possesses the F146, A153, and F155 (FAF) motif, but inspection of endogenous neosubstrate sequences showed that the FHF, FRF, and LFC motifs are also found at these positions. Consequently, these motifs were also explored in the mutant library (Figure 3Aii), with the idea that they might lead to ZF sequences showing enhanced interactions with CRBN.^6,44^ Another subset of mutations was included that focused on positions 147 and 154, while keeping residues 146, 149, 150, 153, and 155 the same as the equivalent positions in the ZF degron of SALL4 (F, S, V, H, and F respectively) (Figure 3Aiii).^16^

Mutations at position 167 were deprioritized as no CRBN-ZF stabilizing mutations were identified in the *in silico* screen, but a small subset of mutants exploring 19 mutations at 147 (all except proline) alongside an L167 K mutation was included, as inspection of endogenous neosubstrate sequences suggested a high frequency of lysine at position 167 in naturally occurring degrons (Figure 3Aiv).^6^ Finally, a single G152W mutant was included as a negative control, as any residue other than glycine at this position interferes with IMiD-induced degradation (Figure 3Av).^16^ Therefore, the total number of ZF sequences explored was 8380.

### A Mutant Library Screen Identifies Degrons That Undergo Bumped IMiD-Induced Degradation

As we sought to screen a large number of mutant ZF degrons with different mutation combinations, we pursued a library screen approach similar to that previously employed by Sievers et al. and Jan et al.^6,44^ Only the most promising compounds from the original set of 36 were used for the library screen. To help narrow the candidates, a subset of compounds was tested for endogenous IKZF1 degradation in Jurkat cells using a fluorescent antibody flow cytometry assay (Figure S5). Consistent with the initial work, compounds **19**, **22**, and **23** did not induce degradation of endogenous IKZF1 in this assay, and so were taken forward. While the diphenyl derivative **33** showed modest degradation of IKZF1 in this assay, it progressed due to both its relatively high selectivity value in the initial screen and its structural difference from compounds **19**, **22**, and **23**. Thalidomide and lenalidomide were also included as controls. All compounds taken forward were observed to display suitable CRBN IC_50_ values and acceptable solubility in phosphate-buffered saline (PBS) (Figure S3).

The library screen was carried out by transducing Jurkat cells with a lentiviral vector pool containing all 8380 sequences, using the same plasmid construct as the previous screen. Low-level transduction (<30%) was carried out to maximize the number of cells with a single integration. An initial round of fluorescence-activated cell sorting (FACS) was used to isolate cells expressing mCherry; these mCherry-positive (mCherry+) cells were then treated with one of the selected compounds for 18 h, and then sorted into 2 populations by FACS: EGFP_low_ and EGFP_+_. The EGFP_low_ population contains cells in which the EGFP-degron fusion protein is depleted due to compound-induced degradation. Next-generation sequencing (NGS) was then conducted to determine which mutant ZF sequences were overrepresented in the EGFP_low_ population compared to the rest of the EGFP_+_ population. Data for all screens are a mean of 3 biological replicates (Figure 3B).

Raw library data (Figures S6 and S7) show that the three repeats of the compound screen correlate well, while the three DMSO control repeats exhibit a lower correlation with each other. This observation indicates that highly similar sets of sequences are enriched for each repeat of the same compound. High correlation is also observed between screens of compounds with similar structures, such as between the three compounds with “bump” groups at the 5 position (compounds **19**, **22**, and **23**), or between thalidomide and lenalidomide. This observation indicates that structurally related compounds induce degradation of similar sequence spaces.

Volcano plots for thalidomide and lenalidomide screens show that the WT degron sequence is overrepresented in the EGFP_low_ population, indicating that, as expected, WT degron degradation is induced relatively efficiently by these compounds, compared to the rest of the library sequences (Figures 4A and S8). However, the opposite is observed for the four bumped compounds (**19**, **22**, **23**, and **33**), where the WT sequence is underrepresented in the EGFP_low_ population (Figures 4A and S8). As the −log 10 *p*-values are generally high, it was decided that log_2_ fold change (log_2_FC) values could be used to assess all sequences going forward.

**Figure 4:**
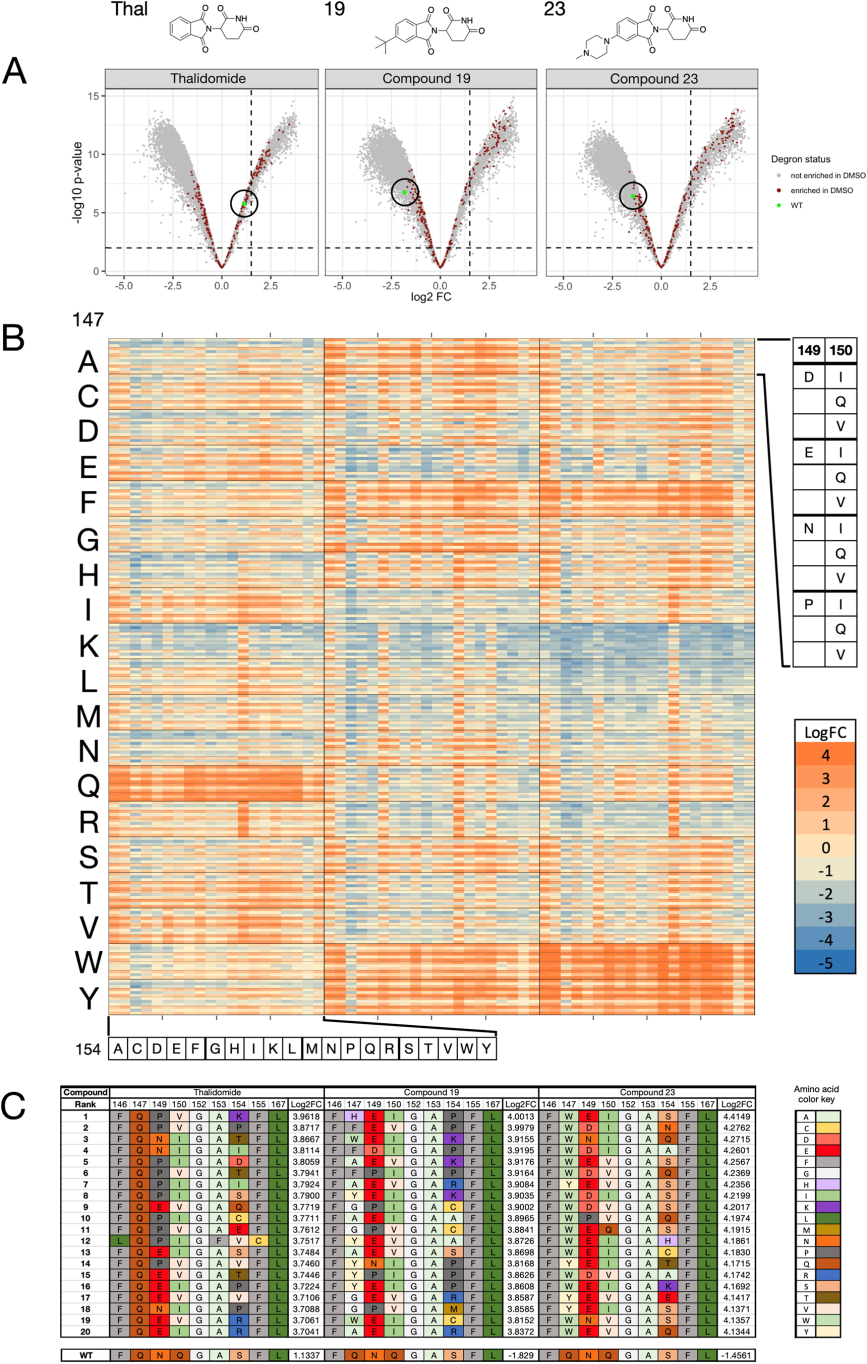
Data obtained from the library screen. (A). Volcano plots for thalidomide (**1**), **19**, and **23** tested against the mutant library. Brown data points represent sequences that were enriched in the DMSO control; the green data point in each plot represents the WT sequence (no change in sequence from the original IKZF1/3 ZF degron). (B). Log_2_FC data for thalidomide (**1**), **19**, and **23** tested against the 4560 library mutant ZF degrons in (i) of the library. Log_2_FC values are arranged according to compound and residue 154 on the *x*-axis, and values are arranged according to residues 147, 149, and 150 on the *y*-axis. All other degron residues are identical to those of the WT IKZF1/3 degron. Color scale shows high log_2_FC as orange, representing high sequence enrichment in the EGFP_low_ population, and low log_2_FC as blue, representing low sequence occurrence. A log_2_FC score of zero, colored as tan, represents equal representation of a sequence in the “degraded” cell population and the remaining cell population after FACS. (C). Table showing the 20 highest ranked mutant ZF sequences by log_2_FC, for compounds thalidomide (**1**), **19**, or **23**; the WT (IKZF1/3 ZF2) sequence and log_2_FC for each of the 3 compound screens are also shown for comparison.

We have conceptually divided the library into three sections: Section i features the “FAF” motif at positions 146, 153, and 155, while sections ii and iii have the “FHF”, “FRF”, and “LFC” motifs at these positions. Inspection of the full library data for library sections i, ii, and iii highlights several key observations (Figure S9). Library section i encompasses more sequences with high log_2_FC values compared to sections ii and iii, indicating that these sequences are effectively degraded when treated with the given IMiD. Although the “LFC” section does have a large number of sequences with higher log_2_FC values, these sequences also tend to have higher log_2_FC values for the DMSO control. This observation suggests that these sequences either show poor expression levels or are susceptible to degradation in the absence of an IMiD derivative.

Within library section i, thalidomide and lenalidomide preferentially degrade sequences that have the WT glutamine at position 147 (Figures 4B and S9). The ^*t*^Bu derivative **19** exhibits the strongest preferences for ZFs with alanine, phenylalanine, tryptophan, or tyrosine at position 147. Smaller numbers of sequences with cysteine, glycine, histidine, or serine at this position are also efficiently degraded by this compound. Compounds **22** and **23** share similar strong overall preferences for ZFs with phenylalanine, tryptophan, or tyrosine at position 147. Interestingly, the *N*-methylpiperazine derivative **23** is especially efficient at degrading degrons with tryptophan at position 147. The diphenyl derivative **33** does not, however, exhibit a clear preference for a specific residue at position 147 (Figures 4B and S9).

For all compounds screened, there is a clear preference for either glutamic acid or proline over the WT asparagine at position 149; likewise, both isoleucine and valine are favored over the WT glutamine at position 150. However, the residue preference at position 154 across all compounds appears to be context dependent, and the trends are less straightforward. Residue preference trends for all compounds can be seen mirrored in the top 20 sequences ranked by log_2_FC for each compound screen (Figures 4C and S10).

Having identified the most efficiently degraded ZF degron sequences for each compound, the most selective compound-sequence pairs were chosen for validation using the same ratiometric fluorescence flow cytometry assay as that previously used. Only sequences degraded by compounds **19** and **23** were progressed, as these compound-degron pairings were expected to show the highest selectivity over the WT. Sequences tested at this stage only have mutations at positions 147, 149, 150, or 154, and so are named based on their residues at these positions, for example, the Q147A mutant has A at 147, N at 149, Q at 150, and S at 154, and so is called ANQS.

Compound **19** degrades the HEIP and AEVK sequences most effectively, with these compound-degron pairs showing 25-fold and 28-fold selectivity over WT, respectively (Figures 5A,B and S11E). It is notable that all five of the library sequences degraded most efficiently by compound **19**, ranked by log_2_FC, possess an acidic residue (either D or E) at position 149. To confirm our earlier results, compound **19** was also tested against the single Q147A mutant sequence ANQS, with only 1.4-fold selectivity over the WT observed in this case (Figures 5A and S11C).

**Figure 5:**
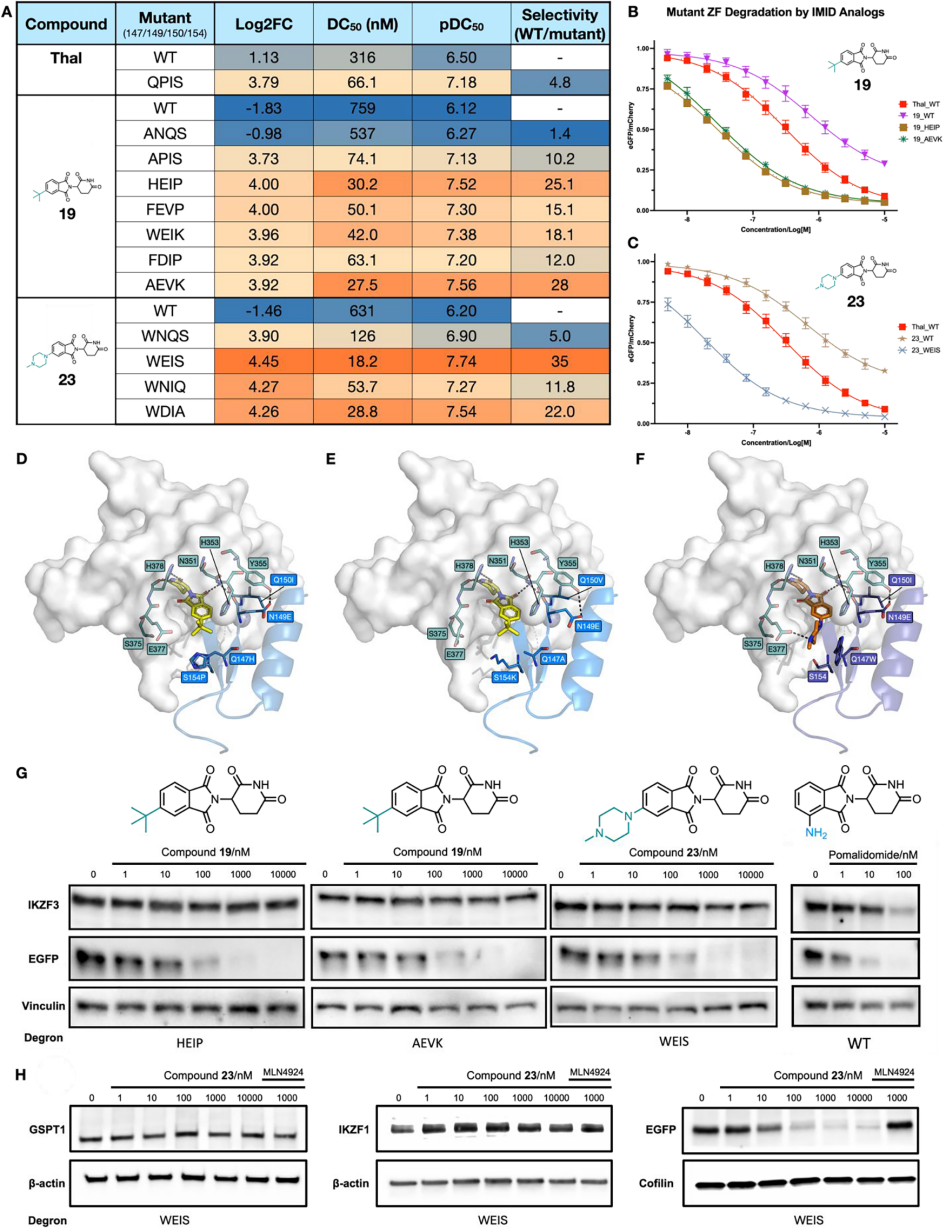
Investigation of the selectivity for certain degrons. (A) Table showing library log_2_FC, DC_50_, pDC_50_, and selectivity values for different compound-mutant ZF degron combinations (18 h incubation time); selectivity = DC_50_(WT)/DC_50_(mutant). (B) Jurkat cells stably expressing either WT or mutant (HEIP or AEVK) ZF degrons fused to EGFP were treated with a concentration curve of thalidomide or compound **19** to yield degradation curves. (C) Jurkat cells stably expressing either WT or WEIS mutant ZF degrons fused to EGFP were treated with a concentration curve of thalidomide or compound **23** to yield degradation curves. (D) Docking model of compound **19** at the interface between CRBN and the HEIP mutant ZF. (E) Docking model of compound **19** at the interface between CRBN and the AEVK mutant ZF. (F) Docking model of compound **23** at the interface between CRBN and the WEIS mutant ZF; all three models derived from the crystal structure of CRBN-pomalidomide-IKZF1 ZF2 (PDB ID: 6H0F).^6^ (G). Western blots were performed on transduced Jurkat cells expressing a ZF-EGFP fusion protein with the HEIP, AEVK, WEIS, or WT ZF degron, treated for 18 h with five concentrations of compound **19** or compound **23**, or 3 concentrations of pomalidomide, and an untreated DMSO control. Bands show levels of IKZF3, EGFP, and vinculin loading control. (H) Western blot performed on transduced Jurkat cells expressing the WEIS-EGFP degron, treated for 18 h with five concentrations [as shown] of compound **23**, 1 μM MLN4924 and 1 μM compound **23**, and an untreated DMSO control. Bands show either endogenous GSPT1, IKZF1, β-actin, cofilin, or exogenous EGFP.

The *N*-methylpiperazine derivative **23** induces degradation of the triple mutant sequence WEIS most effectively, with this ligand-degron pair showing the highest selectivity we observed in this assay; 35-fold over WT ZF degron. Interestingly, most sequences for which **23** efficiently induced degradation possess a tryptophan residue at position 147. Even the single Q147W mutant, WNQS, shows 5-fold selectivity compared to that of WT, indicating that incorporation of this residue at position 147 is favorable for **23** stabilizing the CRBN-**23**-IKZF1/3 ternary complex (Figures 5A and S11D). Like the sequences above, WEIS also incorporates an acidic glutamate residue at position 149, again suggesting that this is beneficial for CRBN-compound-IKZF1/3 ternary complex stability. As W147 is a relatively large residue, this suggests that compound **23** forms a favorable interaction with W147 that stabilizes CRBN-IMiD-ZF, resulting in an enhanced degradation of the ZF-fusion protein.

Thalidomide was tested against an N149P-Q150I double mutant (QPIS) to assess the effect of including mutations that were predicted to be favorable at these positions. Thalidomide induced degradation of the QPIS degron with 4.8-fold selectivity over the WT (Figures 5A and S11B). Interestingly, a similar result was observed when compound **19** was tested against the Q147A-N149P-Q150I triple mutant, APIS (Figures 5A and S11C). Compound **19** induced degradation of the APIS degron 10-fold more effectively compared to the WT, which is a substantial improvement over the 1.4-fold selectivity displayed by ANQS. This demonstrates that these mutations increase degradation induced by IMiD, despite these residues not being located immediately proximal to the IMiD-binding site.

Docking studies were carried out to assess the potential structure of the CRBN-IMiD-ZF interfaces for the three most selective compound mutant pairings: **19**-HEIP, **19**-AEVK, and **23**-WEIS (Figure 5D,E, and F). FoldX 5 was used to generate the mutant structures from the DDB1-CRBN-pomalidomide complex bound to IKZF1­(ZF2) (PDB ID: 6H0F). The bumped ligands were docked into these structures using GOLD. While these studies are useful to help rationalize our experimental findings, we note that further verification using experimental structural approaches is needed to confirm our computational predictions.

In all three cases, a hydrogen bond is predicted to form between E149 in the ZF and Y355 of CRBN. The WT N149 is not observed to form this interaction in PDB ID: 6H0F, suggesting that this additional interaction stabilizes the CRBN-ZF protein–protein interaction (PPI). WT Q150 does not form any polar interactions with CRBN, but its side chain forms part of the hydrophobic interface between ZF and CBRN. HEIP and WEIS possess a Q150I mutation at this position, while AEVK has a Q150 V mutation. This observation suggests that the I150 or V150 residues replace the WT Q150 hydrophobic interactions, again helping to stabilize the CRBN-ZF PPI.

AEVK possesses a Q147A mutation, which seems to have a classic bump-and-hole effect, where the smaller A147 residue can accommodate the larger ^*t*^Bu moiety of compound **19**. This degron incorporates a S154K mutation, which is spatially adjacent to position 147, with K154 predicted to form an ionic interaction with CRBN E378. This interaction places the lysine side chain away from the bumped IMiD and also likely further stabilizes the CRBN-ZF PPI. It is interesting to note that the three additional mutations are having a substantial effect as the AEVK degron has 28-fold selectivity vs WT, compared to only 1.4-fold selectivity for the simple Q147A mutant ANQS. The HEIP degron has Q147H and S154P mutations. Docking studies predict that the conformationally restricted P154 residue enables H147 to move, accommodating the ^*t*^Bu bump of compound **19**. The WEIS degron, which is selectively degraded by compound **23**, possesses only three mutations, as S154 is the same as WT. The E149 and I150 mutations are thought to have the same effect as in the HEIP degron above, and serve to stabilize the CRBN-ZF PPI. At pH 7.4, *N*-methyl-piperazine amine of **23** is predicted to be >93% protonated on the methylated piperazine nitrogen (Chemicalize), and docking suggests that this moiety will form a salt bridge with CRBN E378. This salt bridge orients the piperazine ring so that it can form a cation-π interaction with W147. This observation explains why most of the mutants that are preferentially degraded by compound **23** possess a tryptophan at position 147.

It is interesting to note that in the WT DDB1-CRBN-pomalidomide complex bound to IKZF1­(ZF2) (PDB ID: 6H0F), two structured water molecules are observed at the CRBN-ZF interface, forming hydrogen bonds with CRBN S376 and E378, and IKZF1 Q147 and N149. The structures predicted by our docking studies indicate that these water molecules will not be accommodated in the CRBN-IMiD mutant ZF ternary complexes. It is therefore possible that, in addition to forming new interactions with the mutant degrons, the bumped ligands are being accommodated by the loss of two water molecules at the CRBN-ZF interface. However, this hypothesis must be verified by further structural studies. We note that this is one of the first examples of a bump-and-hole approach on a ternary complex, and therefore, the effects of the mutant combinations are harder to predict than in a ligand-protein binary complex setting. Consequently, while we do have an example of a Q147A mutant, where a smaller residue is included at position 147, we also have an example of a Q147W mutant, where W is at least the same size as Q if not larger.

Analysis using Western blotting, on a presorted population of mCherry+ cells, confirmed that compounds **19** or **23** induced complete degradation of EGFP tagged with either the HEIP (**19**), AEVK (**19**), or WEIS (**23**) mutant degrons in transduced Jurkat cells, while endogenous IKZF3 levels remained unaffected (Figures 5G, S12, S13, and S14). In Jurkat cells transduced with the WT degron-tagged EGFP, pomalidomide not only induced degradation of EGFP but also depleted endogenous IKZF3 (Figure S15). In untransduced Jurkat cells, compounds **19** and **23** do not induce degradation of endogenous IKZF3, but, as expected, pomalidomide did cause IKZF3 degradation (Figure S16). In Jurkat cells transduced with EGFP tagged with WEIS, endogenous proteins IKZF1 and GSPT1 showed no degradation when treated with compound **23**, while EGFP-WEIS showed complete degradation under these conditions (Figures 5H and S17).

As the **23**-WEIS compound mutant pairing exhibited the highest selectivity value, we conducted tandem mass tag (TMT) mass spectrometry proteomics to determine the whole cell selectivity of compound **23**. Quantitative proteomics screens were carried out in untransduced Jurkat cells, embryonic stem cells (ESCs), and the multiple myeloma cell line, MM.1S. We used Jurkat cells, as our work up to this point had employed this cell line, and we selected ESCs and MM.1S cells as they express known CRBN neosubstrates, including IKZF1, IKZF3, RAB28, RNF166, SALL4, ZFP91, and ZNF827. The cells were treated with compound **23** (10 μM), thalidomide (10 μM), lenalidomide (1 μM), or pomalidomide (1 μM) for 16 h using a 15-plex TMTpro SPS-MS^56^ workflow (Figures 6A–F, S18, and S19).

**Figure 6:**
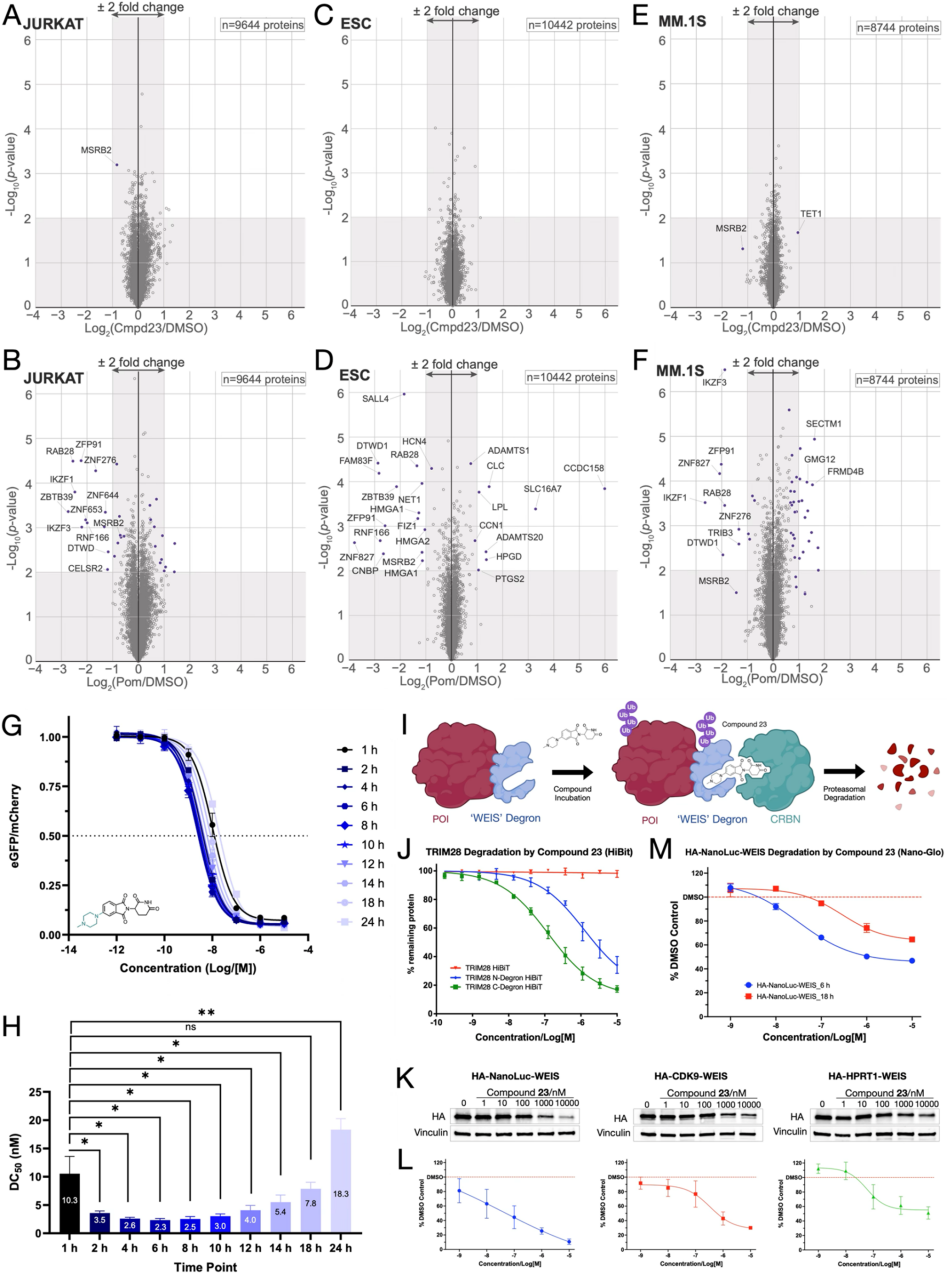
WEIS degron and compound 23 selectivity. Jurkat, ESC, and MM.1S cells were treated for 16 h with the corresponding compound or DMSO control, and the protein abundance was analyzed using multiplexed TMTpro quantification mass spectrometry. Quantitative proteomics profiling following cell treatment with compound **23** (10 μM) or pomalidomide (1 μM). (A) Jurkat cells were treated with compound **23**. (B). Jurkat cells treated with pomalidomide. (C) ESCs were treated with compound **23**. (D) ESCs were treated with pomalidomide. (E) MM.1S cells treated with compound **23**. (F) MM.1S cells treated with pomalidomide. log_2_FC is shown on the *x*-axis, and the −log_10_(*p*-value) is shown on the *y*-axis. Values shown are the mean of three biological replicates. WEIS degron and compound 23 kinetic degradation profile. (G) Jurkat cells were transduced with the WEIS-EGFP degron, then incubated with a concentration range of compound **23** (1 pM – 10 μM) for 1–24 h [as shown] and analyzed using flow cytometry. The DMSO-normalized ratiometric values of EGFP/mCherry are shown. (H). The half-maximal (DC_50_) values from the assay in panel A are shown. Mean and SD values are plotted from three biological replicates. Multiple paired two-tailed *t* tests were performed *p-value <0.05; **p-value <0.01; “ns” nonsignificant. Application of the WEIS degron to POI degradation. (I) Schematic of degradation of a POI tagged with the WEIS degron by compound **23**. (J) Jurkat cells were lentivirally transduced with TRIM28 with either N-terminal or C-terminal WEIS degron tag (or untagged control) and HiBit peptide tag, then incubated with a concentration curve of compound **23** (100 pM – 10 μM) for 24 h and analyzed using a HiBit luminescence assay. C-terminal WEIS degron DC_50_ = 125 nM; *D*_max_ = 83%; N-terminal WEIS degron DC_50_ = 1268 nM; and *D*_max_ = 66%. (K). HEK293T cells were lentivirally transduced with one of three POI fusion constructs–NanoLuc, CDK9, or HPRT1–with an N-terminal HA tag and a C-terminal WEIS degron, then incubated with a concentration range of compound **23** (1 nM – 10 μM) for 18 h and analyzed using HA antibody immunoblotting. One representative blot is shown, and all blots are included in Figure S23. (L). Quantification of the Western blots showed that compound **23** induced degradation of the WEIS-POI fusion proteins. WEIS-NanoLuc DC_50_ = 47.8 nM; *D*_max_ = 89%; WEIS-CDK9 DC_50_ = 299 nM; *D*_max_ = 70%; WEIS-HPRT1 DC_50_ = 67.1 nM; and *D*_max_ = 49%. (M). HEK293T cells were lentivirally transduced with NanoLuc with an N-terminal HA tag and a C-terminal WEIS degron, then incubated with a concentration curve of compound **23** (1 nM – 10 μM) for either 6 or 18 h, and analyzed using a NanoGlo luminescence assay. DC_50_ = 31.0 nM; *D*_max_ = 53%; 18 h: DC_50_ = 290 nM; *D*_max_ = 35%.

In Jurkat cells, compound **23** (10 μM) had no major effect on any of the 9644 quantified proteins (Figure 6A), while pomalidomide (1 μM) induced significant (Welch’s *t* test) degradation of at least 20 proteins (Figure 6B). Thalidomide (10 μM) did not induce major protein degradation, and lenalidomide (1 μM) induced significant (Welch’s *t* test) degradation of at least 6 proteins (Figure S18). In ESCs, compound **23** (10 μM) did not induce any protein degradation (Figure 6C), while pomalidomide (1 μM) induced the significant (Welch’s *t* test) degradation of approximately 25 proteins of the 10442 proteins assessed, including SALL4 (Figures 6D and S19). In MM.1S cells treated with pomalidomide (1 μM), IKZF1, IKZF3, and at least 6 other proteins were significantly degraded (Welch’s *t* test). Treatment with compound **23** (10 μM) did not significantly (Welch’s *t* test) induce degradation of any protein in this cell line (Figures 6E and 7F).

**Figure 7:**
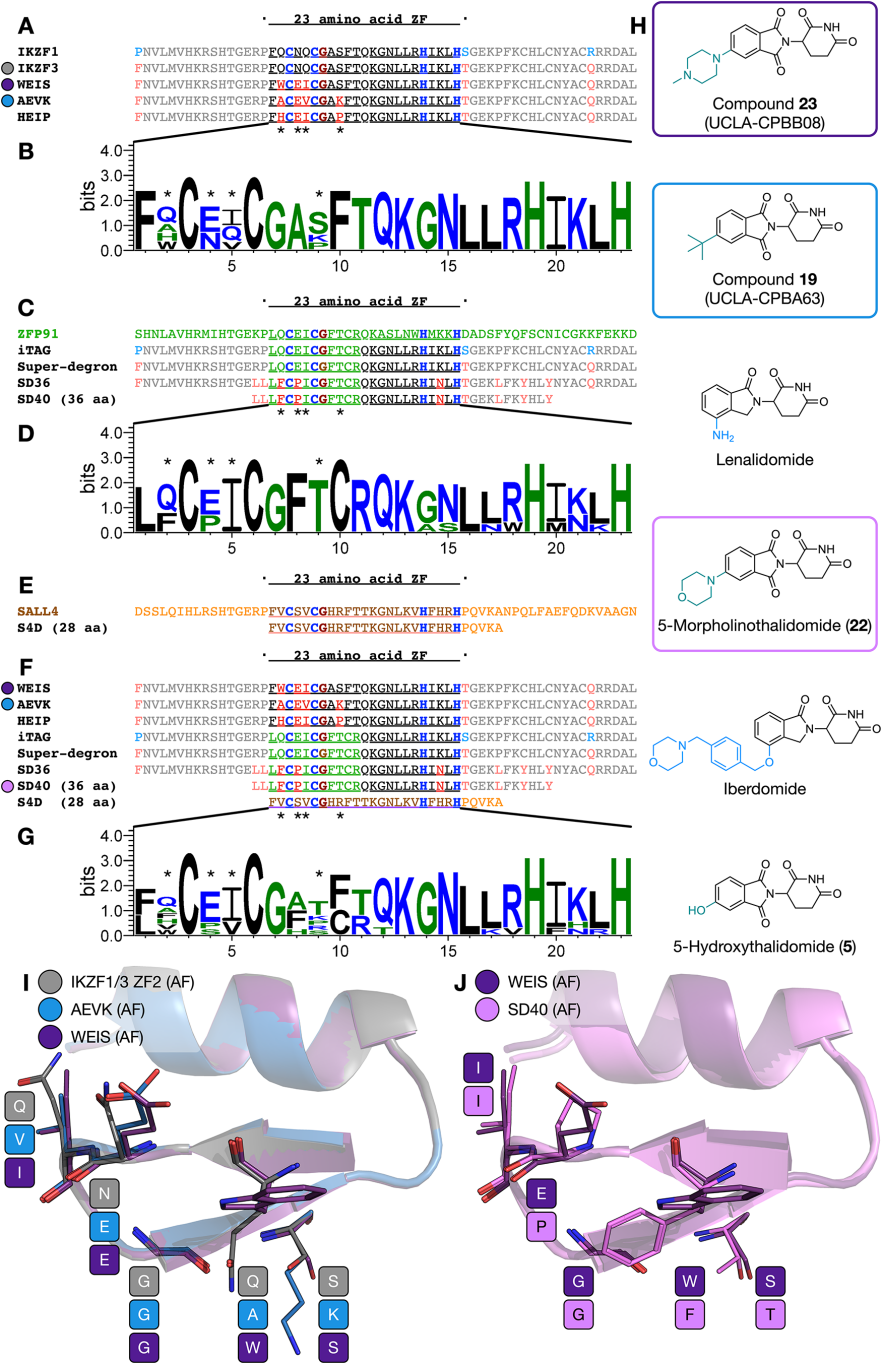
Comparison of endogenous and unnatural ZF-based degrons. (A) Comparison of the amino acid sequences of the IKZF1/3-based degrons discussed in this study. (B) The sequence logo of the ZFs sequences is shown in panel A. (C) Comparison of the amino acid sequences of the ZFP91 ZF4-based degrons discussed in this study. (D). The sequence logo of the ZFs sequences is shown in panel C. (E) Comparison of SALL4 ZF3 and the S4D degron on which it is based. (F). Comparison of the amino acid sequences of the unnatural ZF-based defects discussed in this study. (G). The sequence logo of the ZFs sequences is shown in panel F. (H) The structures of the IMiD derivatives that induce proximity between CRBN and the ZF degrons are discussed in this study. (I) Overlay of the AlphaFold 3-generated structures of IKZF1/3 ZF2 (carbon = gray), AEVK (carbon = blue), and WEIS (carbon = purple). (J) Overlay of the AlphaFold 3-generated structures of WEIS (carbon = purple) and SD40 (carbon = pink). The sequence logos were generated by using WebLogo3. In primary sequences, the C2H2 residues are shown in blue, the CXXCG motif glycine is shown in maroon, residues derived from IKZF1/3 are shown in black, residues derived from ZFP91 are shown in green, and altered residues are shown in red.

Having demonstrated the selective nature of the WEIS-**23** pair, the kinetics of degradation were investigated. We performed a time-course experiment in which Jurkat cells transduced with the WEIS-EGFP degron were treated with compound **23** at a range of concentrations (1 pM–10 μM) at a selection of time points, and the ratiometric degradation values of EGFP/mCherry were assessed using flow cytometry, as above. A set of samples included carfilzomib, a well-characterized proteasome inhibitor, to assess the rescue of EGFP-WEIS degradation,^57^ and by implication the involvement of the proteasome in the observed degradation. Compound **23** induced the degradation of WEIS-EGFP in a manner consistent with the above findings (Figure 5A). Interestingly, we observed that the DC_50_ value varied significantly (paired *t* test) over time, compared to the DC_50_ value determined at 1 h. The DC_50_ value was 10.3 nM at 1 h, decreased significantly (two-tailed *t* test) to a value of 2.3 nM at 6 h, and then increased to 18.3 nM at 24 h (Figure 6G,H). Degradation was rescued by the addition of carfilzomib (1 μM), with reduced WEIS-EGFP degradation evident, even at higher concentrations of **23**, and proteasome inhibition waning as 24 h approaches (Figure S20). This result is consistent with the proposed proteasomal mechanism of degradation (Figure 1).

We hypothesized that the time-dependent variation in DC_50_ values for compound **23** might result from instability in the cellular environment or export from the cells, leading to a lower cellular concentration after the 24 h period. To assess its cellular stability, we incubated Jurkat cells with 25 μM of compound **23** and analyzed how much compound was recovered from the cells after 0.5, 6, and 24 h (Figure S21) using HPLC analysis. After 0.5 h, we observe 130 nM of compound remaining, 30 nM at 6 h, and almost no compound remaining at 24 h. These data suggest that at 6 h, substantial protein degradation has occurred, and our HPLC data show that there is also a sufficient concentration of compound **23** remaining in the cells to induce continued degradation. The data also indicate that compound **23** is being metabolized, transported out of the cells, or both, over a 24 h period, explaining the increase in DC_50_ value by this time point (Figure 6G,H).

Having demonstrated the selectivity of the **23**-WEIS degron system, we applied it to induce degradation of TRIM28, a disease-relevant bromodomain- and PHD-containing protein, for which there are no ligands.^58^ Jurkat cells were CRISPR-edited to knockout the endogenous TRIM28 gene. Genes for TRIM28 tagged with the WEIS mutant ZF degron at either the N- or C-terminus (or untagged) and also tagged with HiBiT at the other terminus were then transduced into Jurkat cells and incubated with compound **23** for 24 h. The linker region used was the same rigid design as used for the previously tested EGFP fusion. Degradation levels were then assessed using a HiBiT luminescence assay (Figure 6J). Compound **23** induced degradation of both the N- and C-terminal-tagged fusion protein, with DC_50_ values of 1268 and 125 nM, respectively. For the C-terminal-tagged protein, a *D*_max_ value of 83% was observed with 10 μM of **23**. Compound **23** did not affect the cell viability of any of the cell lines at any concentration (Figure S22). This result demonstrates that our technology can be used to efficiently induce the degradation of an unliganded protein. We note that no optimization of the TRIM28-ZF linker was undertaken, and more potent degradation could likely be achieved through investigating the linker region.

Having demonstrated the applicability of the WEIS degron to TRIM28, we investigated the degradation of further POIs, with an interest in illustrating the applicability of this system across different cellular compartments. To this end, a nuclear protein, CDK9, and a cytoplasmic protein, HPRT1, were selected, in addition to a well-characterized enzyme capable of generating bioluminescence, NanoLuc.^24,59^ Fusion constructs comprising a POI with an N-terminal HA tag linked to the POI by two glycine residues and a C-terminal WEIS motif linked to the POI by a flexible GGGGSGGGGS linker region were exogenously expressed in HEK293T cells. Degradation of each fusion protein was demonstrated using Western blot (Figures 6K,L and S23) after 18 h incubation with a concentration range (1 nM - 10 μM) of compound **23**. The WEIS-CDK9 fusion protein was degraded with DC_50_ = 299 nM and *D*_max_ = 70%, the WEIS-HPRT1 fusion protein was degraded with DC_50_ = 67.1 nM and *D*_max_ = 49%, and the WEIS-NanoLuc fusion protein was degraded with DC_50_ = 47.8 nM and *D*_max_ = 89%. Degradation of the HA-NanoLuc-WEIS fusion construct was further confirmed using a NanoGlo assay (Figure 6M), with a difference in DC_50_ observed between 6 h (30.7 nM) and 18 h (290 nM) incubation times, corroborating the time-course results seen for WEIS-EGFP degradation in Figure 6G,H. We note that for all these constructs, no optimization of either the choice of N- or C-terminus for the degron, or linker length or type, was undertaken, and more potent degradation could likely be achieved through optimization of either of these fusion protein design aspects.

It is also noteworthy that this system is not yet optimized for use in mouse cells, as mouse cereblon contains a key V388I mutation that prevents IMiD-induced recruitment of ZF neosubstrates.^23^ To test the compatibility of the WEIS degron with mouse cereblon, we lentivirally transduced 3T3 mouse cells with the same WEIS-EGFP-IRES-mCherry plasmid as used in previous tests, after which the cells were incubated with compound **23** for 18 h (Figure S24), yielding modest levels of WEIS-EGFP degradation compared to human cells. We anticipate that mutations could be introduced to this degron to make it mouse-compatible for future *in vivo* studies.

Induced protein degradation has emerged as a powerful tool to assist validation of putative therapeutic targets. Key considerations in ensuring that this approach is as effective as possible are the size of the degron tag, the physicochemical properties of the small molecule inducer of degradation, and the selectivity for induced degradation of the POI over other cellular proteins. Here, we report a new chemically inducible degron system with distinct advantages over many of the existing systems that address these key points. The most selective degron-IMiD pair that we have identified is WEIS combined with compound **23**. This degron is only 60 amino acids, with a molecular weight of 7.0 kDa, and can be easily attached to the N- or C-terminus of a POI. Compound **23** has a molecular weight of 356 and a solubility forecast index (SFI) of 0.27, correlating with high solubility and cell permeability.^60^ The smaller degron and chemical properties of the IMiD analogues described here offer advantages over systems such as dTAG (degron = 11.9 kDa), HaloTag (degron = 33.6 kDa), and Bromotag (degron = 14.9 kDa), which employ much larger bifunctional molecules and degrons. The small size of both the mutant WEIS degron (7.0 kDa) and the bumped IMiD analogue **23** offers solutions to challenges associated with low efficiency of endogenous degron tagging using CRISPR Cas9 knock-in, or effects of large tags on protein function. In addition, the small molecule inducers have higher cell penetration than the bifunctional molecules affiliated with systems, such as dTAG and HaloPROTAC.

The bump-and-hole approach that we have employed minimizes degradation of endogenous neosubstrates that are affected by established IMiDs such as thalidomide, lenalidomide, and pomalidomide. TMT proteomics studies have confirmed the high selectivity of the bumped IMiD analogue **23** in Jurkat cells, a model cancer T-cell line, as well as human embryonic stem cells (ESCs) and MM.1S cells. The library screen approach we adopted has not only identified a degron design that is complementary to compound **23**, but also afforded insight into the nature of the CRBN-IMiD-ZF interface that is central to the mode of action of all IMiDs.

Prior to and during the course of this work, a number of other ZF-based unnatural degron-small molecule pairs have been reported (Figure 7).^24,42,44,53^ As is the case for our work, the majority of these degrons are 60 amino acids in length, with a central 23 amino acids that is the key ZF region that engages with the IMiD and CRBN. Our degrons are based on IKZF3 ZF2, with three (WEIS) or four (AEVK, HEIP) mutations in the central zinc finger region, and with the flanking regions remaining identical to IKZF3 (Figure 7A,B). Superdegron, reported by Jan et al., results from a combination of the first 12 residues of ZFP91 ZF4 and the last 11 residues of IKZF3 ZF2, flanked by regions from IKZF3.^44^ iTAG, reported by Bouguenina et al. is almost identical, but is based on IKZF1 rather than IKZF3 in the flanking regions (Figure 7C,D).^24^ The work of Mercer et al. used Superdegron as a starting point to evolve the 60-amino acid SD36, which introduces three mutations (compared to Superdegron) in the 23-amino acid ZF region, and an additional five in the flanking region.^53^ This degron was further developed to give SD40, which was reduced to 36 amino acids in length by shortening the length of the flanking regions (Figure 7C,D), and S4D, reported by Yamanaka et al., is a 28-amino acid degron based on SALL4 ZF3 (Figure 7E).^42^

It is interesting to note that our three domains and those of others have converged on a number of common features. While the residue at position 149 of IKZF1/3 ZF2 is N, it is E in all of our domains, which is the same as the residue found in the equivalent position of ZFP91 ZF4. Likewise, position 150 of IKZF1/3 ZF2 is Q, but I or V in our degrons, V in SALL4, and I in ZFP91 ZF4 and related degrons, including SD40 (Figure 7F,G). We have proposed (above) that these two residues help to stabilize the ZF-CRBN interaction, and the similarities observed here support that idea.

The residues at IKZF1/3 positions 147 and 154 together help to define the size of the IMiD-binding pocket in the ternary complex. In the case of AEVK, this combination is A and K, with the smaller A residue acting as a classic bump-and-hole replacement. The flexibility of the K side chain also likely allows accommodation of the larger ^*t*^Bu-thalidomide derivative (**19**). Interestingly, in HEIP, WEIS, and SD40, the residue at the equivalent of position 147 is either H, F, or W, which are not typically considered to be small residues. In HEIP, the presence of the conformationally restrained P at position 154, coupled with the flexibility of H, likely helps to provide room for the larger ^*t*^Bu-thalidomide (**19**) at the ternary complex interface (Figure 7I). The residue at position 154 is S in WEIS and T in SD40. S is relatively small and is also the same residue as found in IKZF1/3, while the T residue in SD40 is likely able to form similar hydrogen bonding interactions with CRBN or water as S. We propose that compound **23** forms a cation-π interaction with the W residue of WEIS, which at a minimum allows the accommodation of the *N*-methylpiperazine moiety, and potentially contributes toward stabilization of the complex (Figure 7J). While the morpholine ring of **22** lacks a basic nitrogen atom, and therefore cannot form a cation-π interaction with the W residue, a cryo-EM structure obtained by Mercer et al. shows this group forming a stacking interaction with the F residue.^53^

Of the small molecules used to induce proximity between the degrons and CRBN, it has been shown that compounds **22** and **23** do not induce significant degradation of any endogenous ZFs, while lenalidomide, iberdomide, and **5** all induce degradation of multiple endogenous ZFs (Figure 7H). The structures of these compounds suggest that the inclusion of a bulky substituent at position 5 of the IMiD ring confers selectivity over the majority of endogenous CRBN neosubstrates, while substitution at position 4 is tolerated by some of these neosubstrates. This observation is consistent with the studies of Nguyen et al. in which they identified PROTACs with reduced CRBN neosubstrate off targets.^61^

We anticipate that our new degron system will be a powerful addition to the existing approaches available for target validation studies. We have shown that this degron system can be used to efficiently degrade TRIM28, a disease-relevant protein for which no small molecule ligands are known, even without optimization of the degron size or nature of the linker region. We have also demonstrated degradation of a nuclear protein, CDK9, and a cytoplasmic protein, HPRT1, in addition to NanoLuc. Our approach can be applied to other POIs that do not possess small-molecule ligands as a method of testing their suitability as therapeutic targets. This system could also be useful in scenarios where degradation of typical IMiD neosubstrates, such as IKZF1 or IKZF3, is undesirable, such as in CAR T-cell therapy. Although we note that this would require regulatory approval of compound **23**. Application of this system to the CAR degron approaches reported by Jan et al. and Carboneau et al.^44,46^ could mitigate any unwanted effects observed from the use of established IMiD molecules while retaining the ability to rapidly and reversibly degrade CARs, and therefore control cytotoxicity of infused CAR T-cells in patients.^44,46^

## Supplementary Material









## Notes

Chemistry experimental section, materials and methods, plasmid information, sequences for next-generation sequencing, computational methods, NMR spectra for compounds, HPLC traces for compounds, HPLC traces for compound stability assay, and degron screen data, proteomics data (Figures S1 to S24) (PDF)
degron screen data pack edited for SI v1 (XLSX)
Figure S18 ALL thal len **19, 22, 23, 33** DMSO large heat map linearversion1 copy (XLSX)
Figure S19—Brennan P. et al. (XLSX)

## ∥∥

Department of Drug Science and Technology, University of Turin, 10125 Turin, Italy
