# Supplementary material for: Orthogonal IMiD-Degron Pairs Induce Selective Protein Degradation in Cells
Source: ACS Chem Biol. 2025 Nov 2;20(11):2827–43. doi: 10.1021/acschembio.5c00751 (PMC12645437; doi:10.1021/acschembio.5c00751)
Supplement: Supplementary file 1 [file cb5c00751_si_001.pdf]

Orthogonal IMiD-Degron Pairs Induce Selective Protein Degradation in Cells

Patrick J. Brennan,[1, 2] Rebecca E. Saunders,[3] Mary Spanou,[4] Sarah E. Singleton,[2] Marta Serafini, [1, †] Liang Sun,[5] Guillaume P. Heger,[3] Agnieszka Konopacka,[3] Ryan D. Beveridge,[6] C. Cameron Taylor,[7] Peter DePaola IV,[8] Laurie Gordon,[3] Shenaz B. Bunally,[3] Aurore Saudemont,[3] Andrew B. Benowitz,[3] Carlos Martinez-Fleites,[3] Danielle L. Schmitt,[2, 8, 9] Robert Damoiseaux,[7, 10, 11, 12] Markus A. Queisser,[3] Heeseon An,[5] Charlotte M. Deane,[13] Michael M. Hann,[3] Lewis L. Brayshaw,[3] Stuart J. Conway.[1, 2, 7, 8, 12]**

Supporting Information

[1]Department of Chemistry, Chemistry Research Laboratory, University of Oxford; Oxford, UK.

[2]Department of Chemistry & Biochemistry, University of California Los Angeles, Los Angeles, California, USA.

[3]GSK, Medicines Research Centre; Stevenage, UK.

[4]PerkinElmer; Beaconsfield, UK.

[5]Chemical Biology Program, Memorial Sloan Kettering Cancer Center; New York, USA.

[6]Virus Screening Facility, Weatherall Institute of Molecular Medicine, University of Oxford; Oxford, UK.

[7]California NanoSystems Institute, University of California Los Angeles, Los Angeles, California, USA.

[8]Molecular Biology Institute, University of California Los Angeles, Los Angeles, California, USA.

[9]Institute for Quantitative and Computational Biosciences, University of California Los Angeles, Los Angeles, California, USA.

[10]Department of Molecular and Medical Pharmacology, University of California Los Angeles, Los Angeles, California, USA.

[11]Department of Bioengineering, University of California, Los Angeles, California, USA.

[12]Jonsson Comprehensive Cancer Center, University of California, Los Angeles, California, USA.

[13]Department of Statistics, University of Oxford; Oxford, UK.

[†]Current address: Department of Drug Science and Technology, University of Turin, 10125 Turin, Italy

*Corresponding author. Email: brayshaw.lewis@gmail.com; stuartconway@ucla.edu

The PDF file includes:

Figs. S1 to S24

Materials and Methods

Supplementary Text

Other Supplementary Materials for this manuscript include the following:

Data S1 (Separate File)

Data S2 (Separate File)

Data S3 (Separate File)

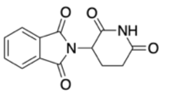

1

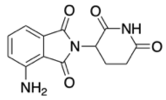

2

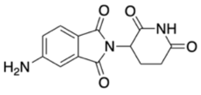

3

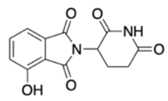

4

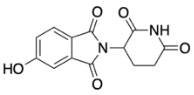

5

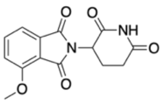

6

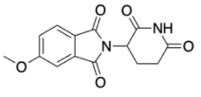

7

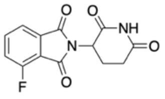

8

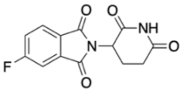

9

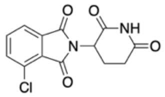

10

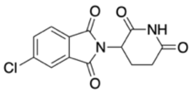

11

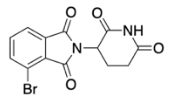

12

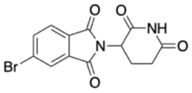

13

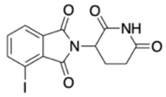

14

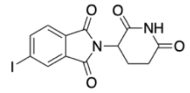

15

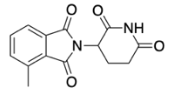

16

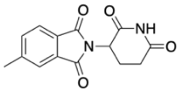

17

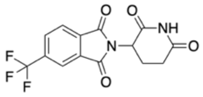

18

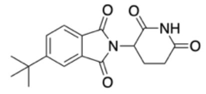

19

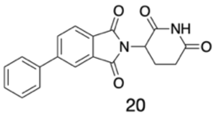

20

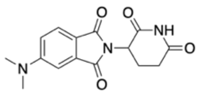

21

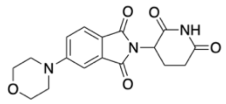

22

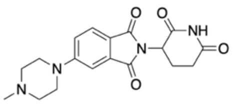

23

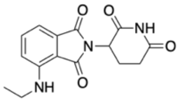

24

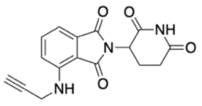

25

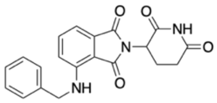

26

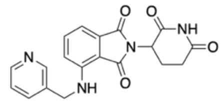

27

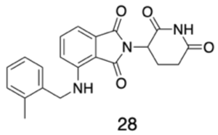

28

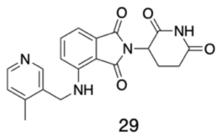

29

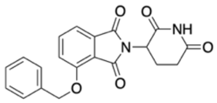

30

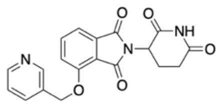

31

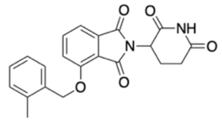

32

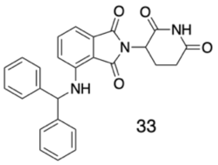

33

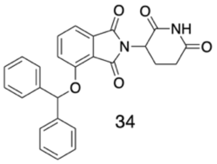

34

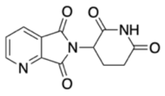

35

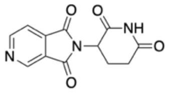

36

Figure S1. Full compound library of 36 IMiD analogs used in biological testing.

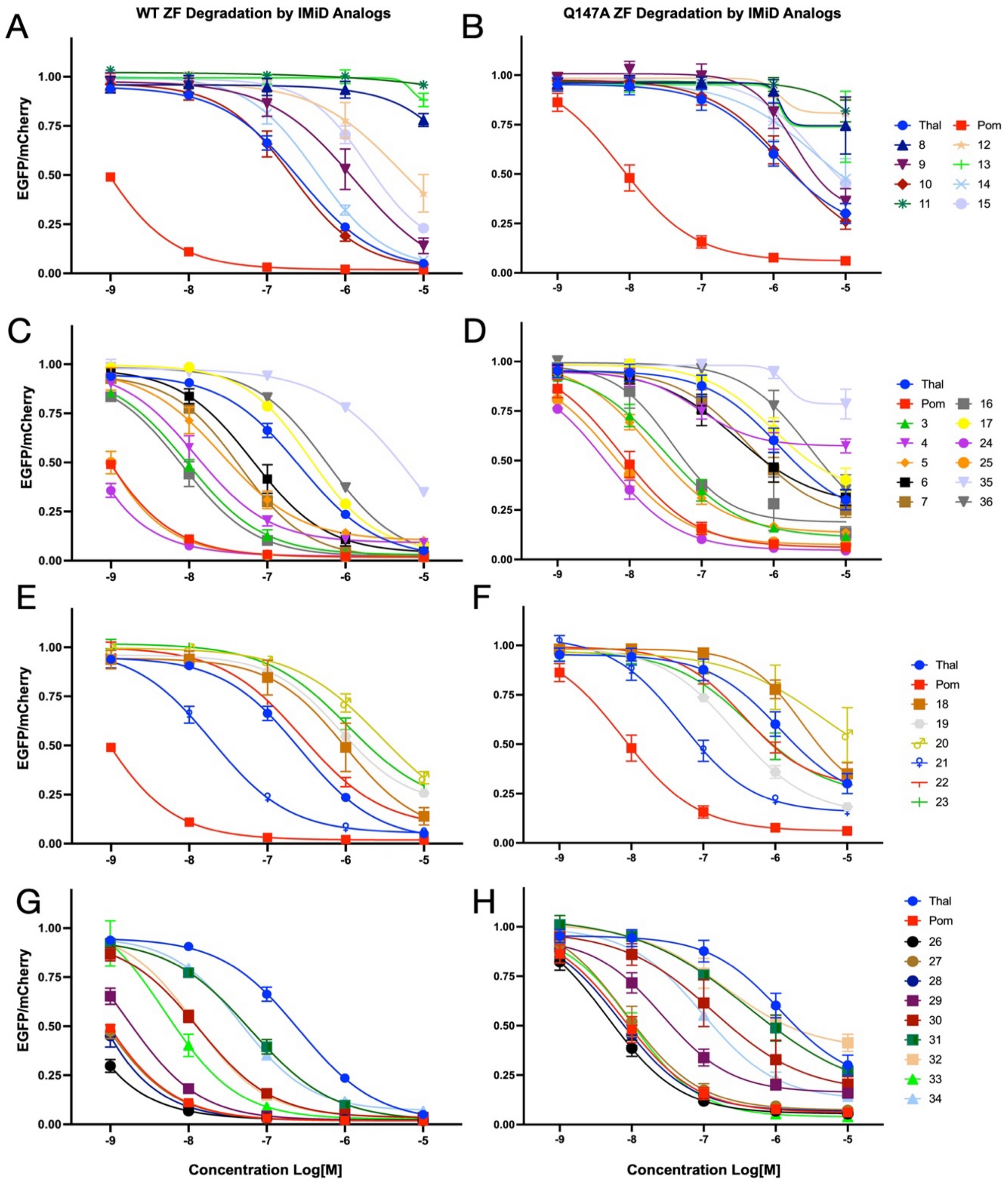

Figure S2. Jurkat cells lentivirally transduced with either WT-EGFP-IRES-mCherry or WEIS-EGFP-IRES-mCherry were treated with compound for 18 h: **A.** Degradation curves for halogenated IMiD analogs against WT ZF degron. **B.** Degradation curves for halogenated IMiD analogs against Q147A ZF degron. **C.** Degradation curves for IMiD analogs with small bump

groups against WT ZF degron. **D.** Degradation curves for IMiD analogs with small bump groups against Q147A ZF degron. **E.** Degradation curves for IMiD analogs with large bump groups at the 5-position against WT ZF degron. **F.** Degradation curves for IMiD analogs with large bump groups at the 5-position against Q147A ZF degron. **G.** Degradation curves for analogs of compound 26 against WT ZF degron. **H.** Degradation curves for analogs of compound 26 against Q147A ZF degron.

Thalidomide 'core'

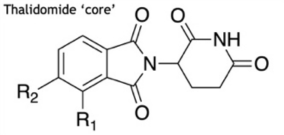

Data for 36 IMID Analogs

Coumpound Number	R_1	R_2	Cereblon pIC_{50}	Solubility /μM	Coumpound Number	R_1	R_2	Cereblon pIC_{50}	Solubility /μM
**1**	H	H	6.90	321	**19**	H		7.07	265
**2**	NH_2	H	7.19	341	**20**	H		7.06	34
**3**	H	NH_2	7.32	301	**21**	H	N	7.00	254
**4**	OH	H	7.57	342	**22**	H	N, O	6.96	254
**5**	H	OH	7.39	348	**23**	H	N, N	7.00	287
**6**	OMe	H	7.02	387	**24**	HN	H	7.47	307
**7**	H	OMe	7.08	287	**25**	HN	H	7.78	389
**8**	F	H	6.62	162	**26**	HN	H	7.79	54
**9**	H	F	6.73	191	**27**	HN, N	H	7.61	377
**10**	Cl	H	6.86	111	**28**	HN	H	7.85	20
**11**	H	Cl	6.47	53	**29**	HN, N	H	7.64	257
**12**	Br	H	7.10	23	**30**	O	H	7.57	7
**13**	H	Br	6.58	9	**31**	O, N	H	7.08	22
**14**	I	H	7.23	18	**32**	O	H	7.65	17
**15**	H	I	6.79	153	**33**	HN	H	7.61	1
**16**	CH_3	H	7.14	48	**34**	O	H	7.48	2
**17**	H	CH_3	7.12	279	**35**	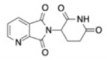		6.00	2
**18**	H	CF_3	6.32	69	**36**	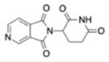		6.05	1

Figure S3. CRBN pIC_{50} and solubility values (in PBS buffer) for all IMiD analog library compounds.

	ΔΔG				ΔΔG				ΔΔG				ΔΔG		
Mutation	Foldx	Rosetta	Mean	Mutation	Foldx	Rosetta	Mean	Mutation	Foldx	Rosetta	Mean	Mutation	Foldx	Rosetta	Mean
N149A	-0.26	-0.23	-0.25	Q150A	-0.22	-1.51	-0.87	A153A	0.01	N/A	0.01	L167A	0.41	1.61	1.01
N149C	0.02	1.76	0.89	Q150C	0.20	1.46	0.83	A153C	2.32	N/A	2.32	L167C	0.46	3.64	2.05
N149D	-0.91	-1.59	-1.25	Q150D	0.53	1.30	0.92	A153D	3.76	N/A	3.76	L167D	1.34	2.91	2.13
N149E	-1.28	-0.71	-0.99	Q150E	-0.34	-1.27	-0.81	A153E	5.33	N/A	5.33	L167E	0.81	2.67	1.74
N149F	-0.58	1.98	0.70	Q150F	-1.11	4.65	1.77	A153F	23.77	N/A	23.77	L167F	0.29	3.04	1.67
N149G	0.45	2.50	1.48	Q150G	0.46	2.66	1.56	A153G	1.42	5.32	3.37	L167G	0.71	3.49	2.10
N149H	0.74	0.98	0.86	Q150H	-0.59	1.44	0.43	A153H	49.24	N/A	49.24	L167H	0.85	2.26	1.55
N149I	-0.23	1.89	0.83	Q150I	-0.99	-2.31	-1.65	A153I	8.08	N/A	8.08	L167I	0.38	1.42	0.90
N149K	-0.91	-0.59	-0.75	Q150K	-0.92	1.36	0.22	A153K	8.20	N/A	8.20	L167K	0.53	0.96	0.75
N149L	-0.58	3.55	1.48	Q150L	-1.32	2.36	0.52	A153L	5.25	N/A	5.25	L167L	0.00	N/A	0.00
N149M	-0.29	1.65	0.68	Q150M	-1.30	1.63	0.16	A153M	4.46	N/A	4.46	L167M	-0.05	1.32	0.63
N149N	0.00	N/A	0.00	Q150N	0.47	1.47	0.97	A153N	5.33	N/A	5.33	L167N	0.83	1.17	1.00
N149P	-1.49	-2.19	-1.84	Q150P	0.60	N/A	0.60	A153P	5.46	N/A	5.46	L167P	0.50	N/A	0.50
N149Q	-0.56	-0.19	-0.38	Q150Q	0.00	N/A	0.00	A153Q	7.81	N/A	7.81	L167Q	0.11	1.79	0.95
N149R	-0.71	-0.04	-0.38	Q150R	-0.27	1.71	0.72	A153R	15.58	N/A	15.58	L167R	0.23	0.89	0.56
N149S	0.41	-0.25	0.08	Q150S	0.15	-0.09	0.03	A153S	1.99	4.85	3.42	L167S	0.26	1.18	0.72
N149T	0.72	0.04	0.38	Q150T	0.02	-1.06	-0.52	A153T	4.97	N/A	4.97	L167T	0.04	2.25	1.14
N149V	0.12	0.51	0.32	Q150V	-0.64	-1.37	-1.00	A153V	4.64	N/A	4.64	L167V	0.83	2.33	1.58
N149W	0.11	2.13	1.12	Q150W	-0.94	3.42	1.24	A153W	22.72	N/A	22.72	L167W	0.56	0.93	0.74
N149Y	0.19	1.22	0.71	Q150Y	0.28	3.88	2.08	A153Y	25.84	N/A	25.84	L167Y	0.08	1.57	0.82

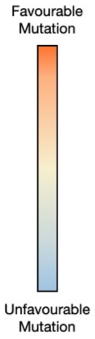

Figure S4. Change in predicted free energies for binding of CRBN with the IKZF3 ZF2 degron when incorporating single mutations at positions 149, 150, 153, or 167 of the ZF, predicted by FoldX and Rosetta. Color scale highlights mutations resulting in a more stable complex in orange, and mutations resulting in less stable complex in blue.

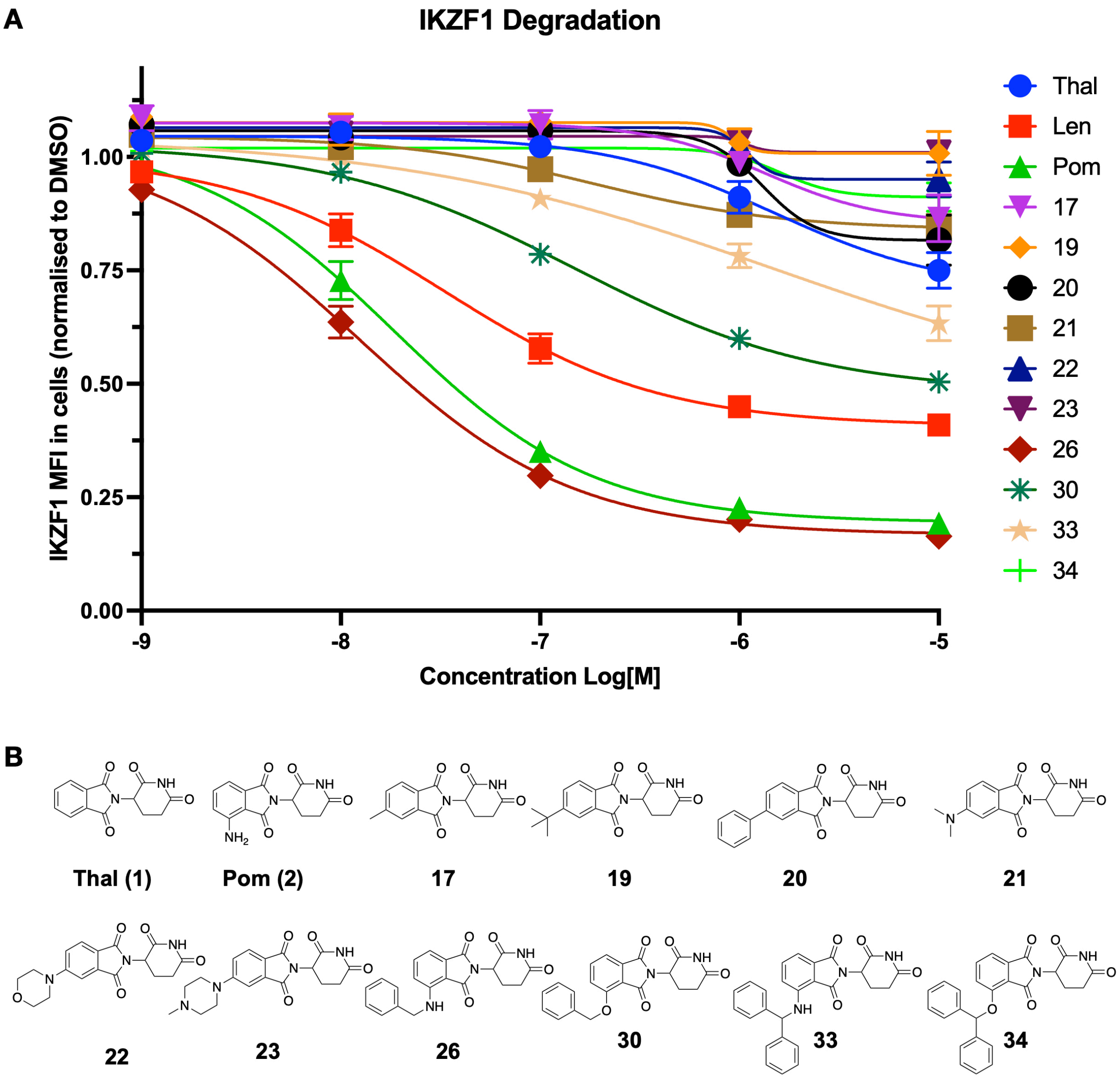

Figure S5. A. Degradation curves for a subset of IMiD analog library compounds (**1**, **3**, **17**, **19**, **20**, **21**, **22**, **23**, **26**, **30**, **33**, **34** and commercially acquired lenalidomide) against endogenous IKZF1 in Jurkat cells after 18 h compound treatment, determined using a fluorescent antibody flow cytometry assay. **B.** Chemical structures for compounds **1**, **3**, **17**, **19**, **20**, **21**, **22**, **23**, **26**, **30**, **33**, and **34**.

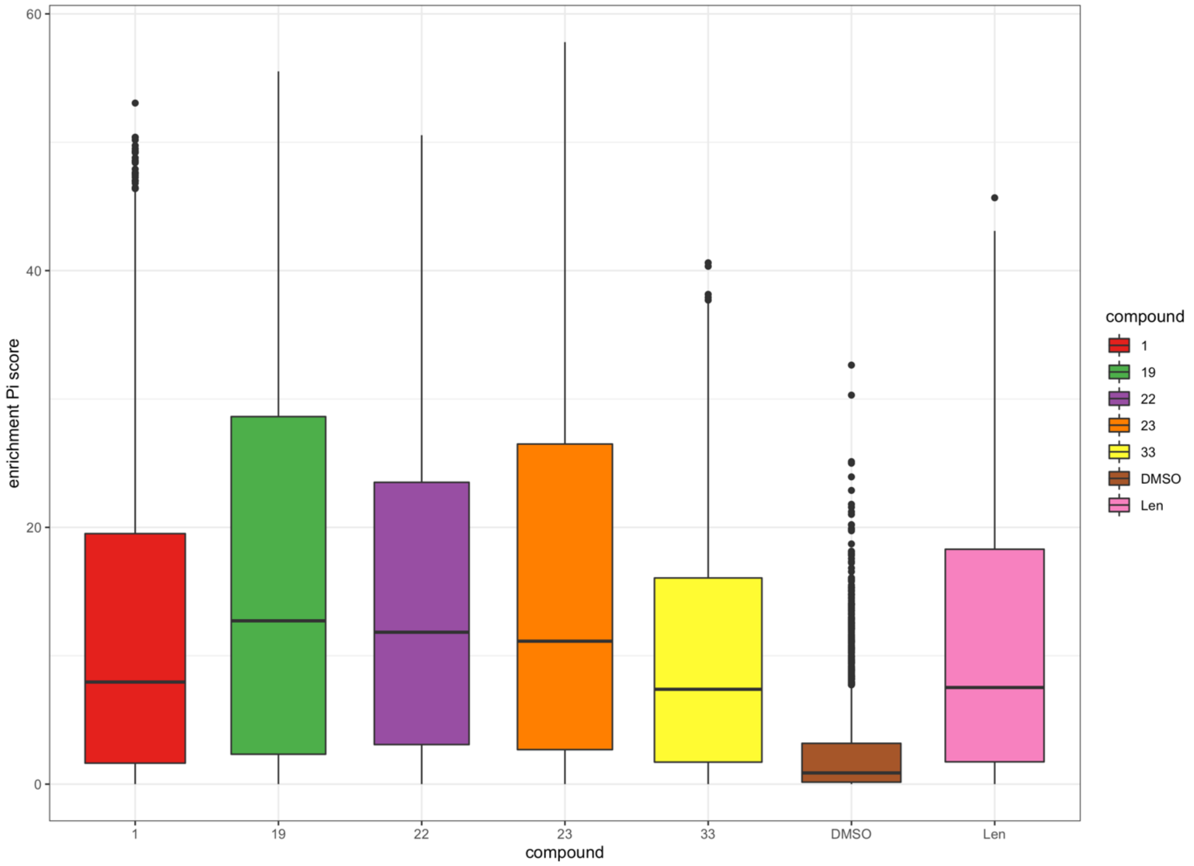

Figure S6. Enrichment Pi score box plots for library screens for compounds **1** (thalidomide), **19**, **22**, **23**, **33** and lenalidomide, and a DMSO control. Pi score is calculated by $\log_2$FC × log10(*p*-value), where *p*-value represents confidence in the result and $\log_2$FC represents fold-enrichment; pi score enables the identification of statistically significant fold-changes.

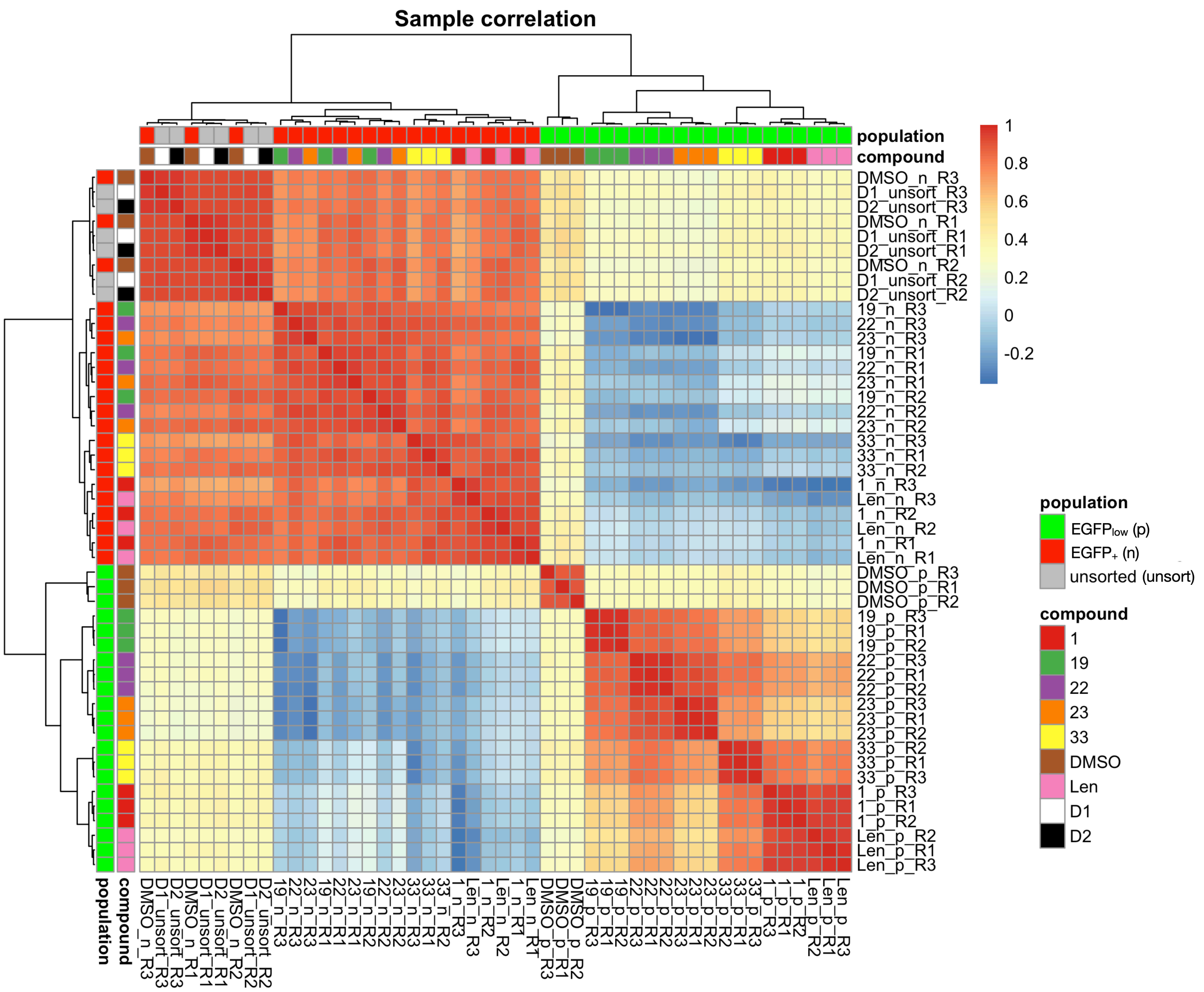

Figure S7. A representation of the similarity of enriched sequences observed in populations of library ZF-EGFP-expressing Jurkat cell populations. Data are shown for three replicates of each condition. A value of 1 represents identical sequences; lower values represent increasing dissimilarity. The plot shows three replicates (R1, R2 and R3) for each condition: sorted $EGFP_{low}$ populations (p) for compound screens (**1** (thalidomide), **19**, **22**, **23**, **33** and lenalidomide) and an untreated control (DMSO); sorted $EGFP_{+}$ populations (n) for the same screens; and two populations of unsorted library cells (D1 and D2; unsort). The blue to red color scale represents sample correlation, with 1 (red) meaning complete sequence correlation.

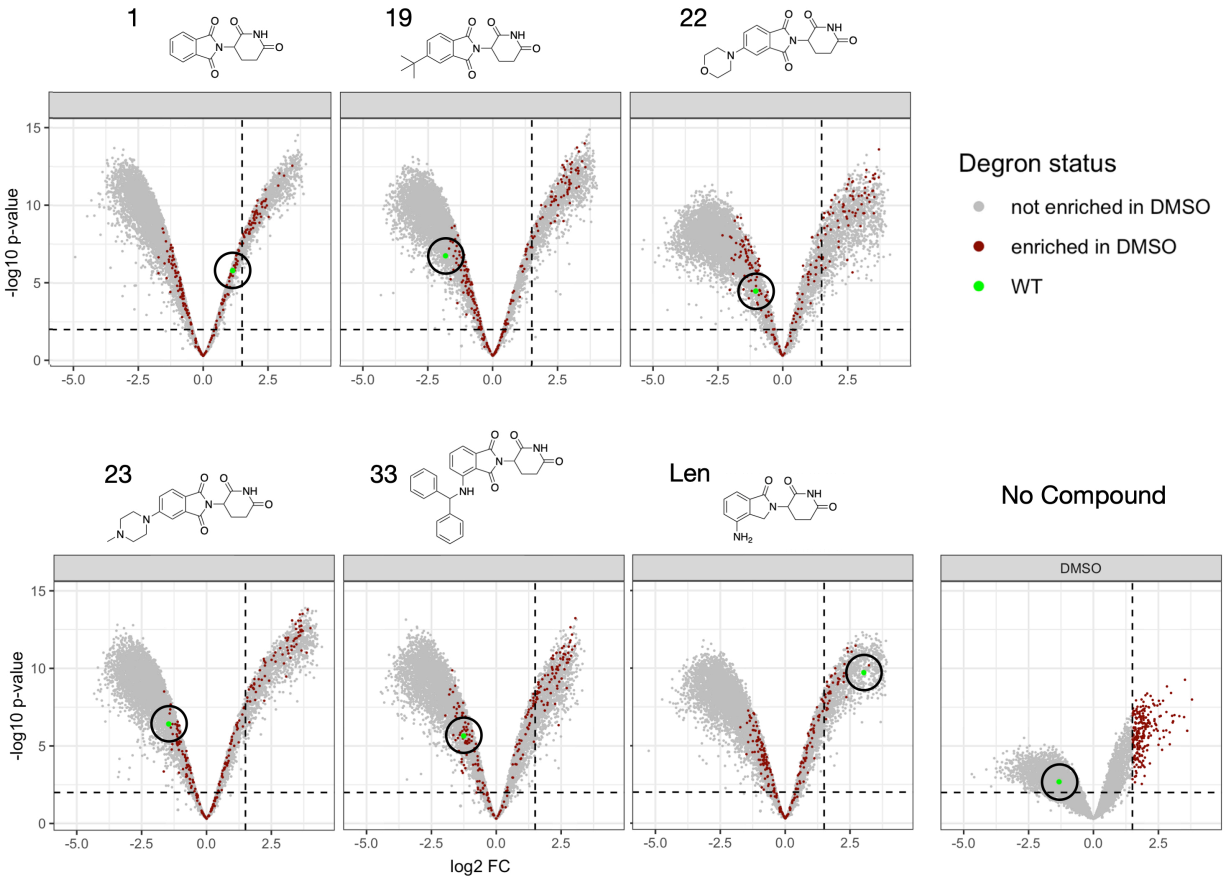

Figure S8. Volcano plots for thalidomide (**1**), lenalidomide, **19**, **22**, **23** and **33** tested against the mutant library, and a DMSO control (18 h compound incubation time). Brown data points represent sequences that were enriched in the DMSO control; the green data point in each plot represents the WT sequence (i.e. no change in sequence from the original IKZF1/3 ZF degron).

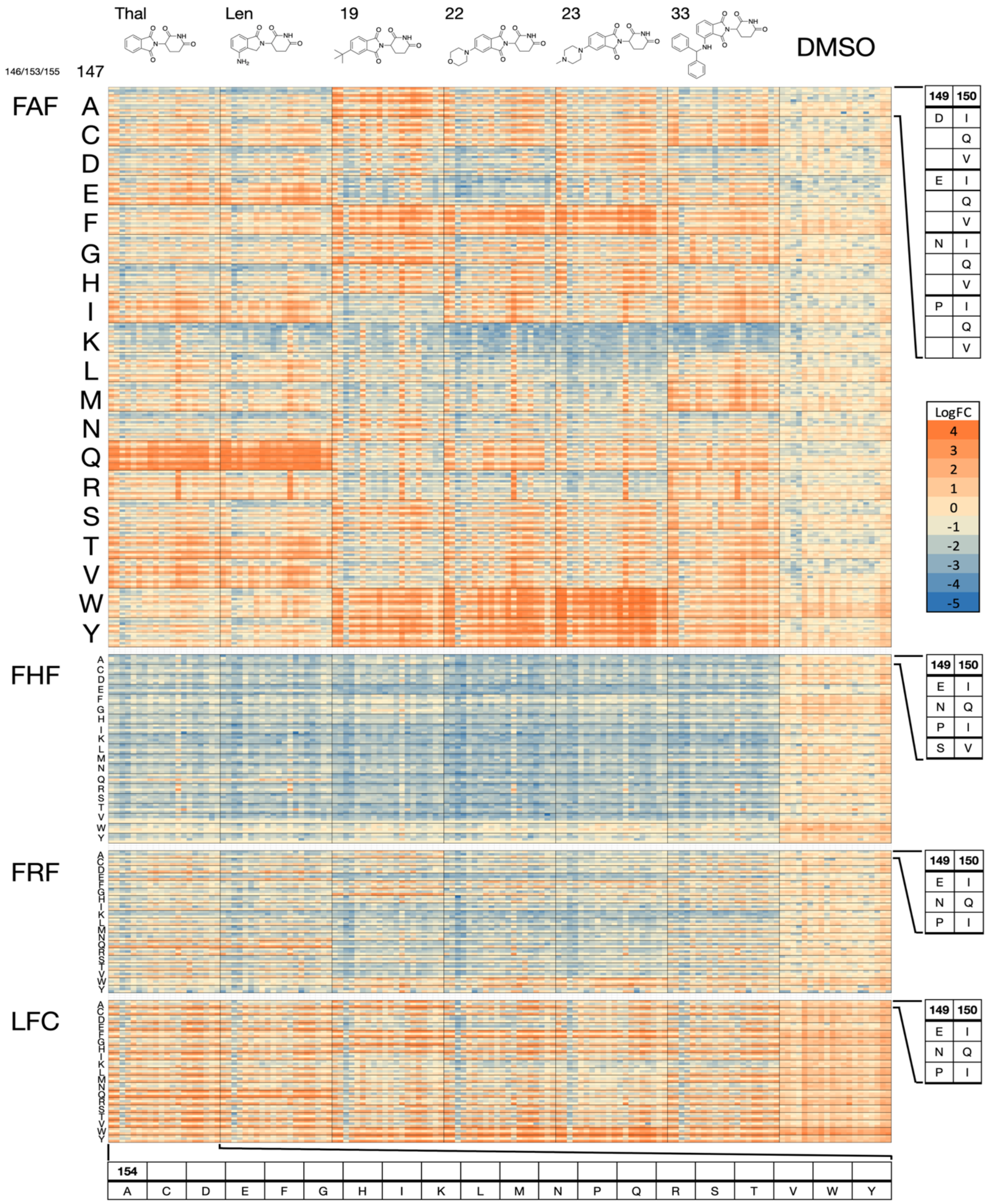

Figure S9. log_2FC data for six compounds and a DMSO control against 8360 mutant ZF degrons. log_2FC values are arranged according to compound and residue 154 on the x-axis, and values are arranged according to residues 146/153/155, 147, 149, and 150 on the y-axis. Color scale shows

high log_2FC as orange, representing high sequence enrichment in the $EGFP_{low}$ population, and low log_2FC as blue, representing low sequence occurrence. A log_2FC score of zero, colored as tan, represents equal representation of a sequence in the 'degraded' cell population and the remaining cell population after FACS.

Compound	Thalidomide									
Rank	146	147	149	150	152	153	154	155	167	Log2FC
1	F	Q	P	V	G	A	K	F	L	3.9618
2	F	Q	P	V	G	A	P	F	L	3.8717
3	F	Q	N	I	G	A	T	F	L	3.8667
4	F	Q	N	I	G	A	I	F	L	3.8114
5	F	Q	P	I	G	A	D	F	L	3.8059
6	F	Q	P	V	G	A	T	F	L	3.7941
7	F	Q	P	I	G	A	I	F	L	3.7924
8	F	Q	P	I	G	A	S	F	L	3.7900
9	F	Q	E	V	G	A	Q	F	L	3.7719
10	F	Q	P	I	G	A	C	F	L	3.7711
11	F	Q	P	V	G	A	E	F	L	3.7612
12	L	Q	P	I	G	F	V	C	L	3.7517
13	F	Q	E	I	G	A	S	F	L	3.7484
14	F	Q	P	V	G	A	V	F	L	3.7460
15	F	Q	E	V	G	A	T	F	L	3.7446
16	F	Q	E	I	G	A	P	F	L	3.7224
17	F	Q	E	V	G	A	V	F	L	3.7106
18	F	Q	N	I	G	A	P	F	L	3.7088
19	F	Q	E	V	G	A	R	F	L	3.7061
20	F	Q	E	I	G	A	R	F	L	3.7041
WT	F	Q	N	Q	G	A	S	F	L	1.1337

Compound	Lenalidomide									
Rank	146	147	149	150	152	153	154	155	167	Log2FC
1	F	Q	N	I	G	A	E	F	L	3.9142
2	F	Q	P	V	G	A	D	F	L	3.8922
3	F	Q	N	V	G	A	V	F	L	3.8861
4	F	Q	P	V	G	A	T	F	L	3.8852
5	F	Q	N	I	G	A	Q	F	L	3.8639
6	F	Q	P	V	G	A	N	F	L	3.8156
7	F	Q	P	I	G	A	L	F	L	3.8345
8	F	Q	N	I	G	A	T	F	L	3.8290
9	F	Q	N	I	G	A	A	F	L	3.8170
10	F	Q	E	V	G	A	A	F	L	3.7915
11	F	Q	N	I	G	A	I	F	L	3.7833
12	F	Q	E	I	G	A	R	F	L	3.7803
13	F	Q	E	Q	G	A	C	F	L	3.7802
14	F	Q	P	I	G	A	S	F	L	3.7741
15	F	Q	P	V	G	A	V	F	L	3.7703
16	F	Q	E	V	G	A	I	F	L	3.7619
17	F	Q	P	Q	G	A	V	F	L	3.7592
18	F	Q	P	Q	G	A	C	F	L	3.7152
19	F	Q	E	Q	G	A	V	F	L	3.7519
20	F	Q	P	Q	G	A	A	F	L	3.7513
WT	F	Q	N	Q	G	A	S	F	L	3.0311

Compound	Compound 19									
Rank	146	147	149	150	152	153	154	155	167	Log2FC
1	F	H	E	I	G	A	P	F	L	4.0013
2	F	F	E	V	G	A	P	F	L	3.9979
3	F	W	E	I	G	A	K	F	L	3.9155
4	F	F	D	I	G	A	P	F	L	3.9195
5	F	A	E	V	G	A	K	F	L	3.9176
6	F	F	P	I	G	A	P	F	L	3.9164
7	F	A	E	V	G	A	R	F	L	3.9084
8	F	Y	E	I	G	A	K	F	L	3.9035
9	F	G	P	I	G	A	C	F	L	3.9002
10	F	A	E	I	G	A	A	F	L	3.8965
11	F	G	P	V	G	A	C	F	L	3.8841
12	F	Y	E	V	G	A	A	F	L	3.8726
13	F	A	E	V	G	A	S	F	L	3.8698
14	F	Y	N	I	G	A	P	F	L	3.8168
15	F	Y	P	I	G	A	P	F	L	3.8626
16	F	Y	E	I	G	A	P	F	L	3.8608
17	F	G	E	V	G	A	R	F	L	3.8587
18	F	G	P	V	G	A	M	F	L	3.8585
19	F	W	E	I	G	A	C	F	L	3.8152
20	F	A	E	I	G	A	R	F	L	3.8372
WT	F	Q	N	Q	G	A	S	F	L	-1.8291

Compound	Compound 22									
Rank	146	147	149	150	152	153	154	155	167	Log2FC
1	F	Y	E	V	G	A	T	F	L	4.1392
2	F	Y	E	I	G	A	T	F	L	4.0154
3	F	R	E	I	G	A	P	F	L	3.9835
4	F	I	P	V	G	A	P	F	L	3.9370
5	F	W	E	I	G	A	Q	F	L	3.9298
6	F	W	P	V	G	A	Q	F	L	3.9190
7	F	W	E	V	G	A	H	F	L	3.9186
8	F	W	E	V	G	A	A	F	L	3.9041
9	L	Y	E	I	G	F	R	C	L	3.9018
10	F	W	D	V	G	A	N	F	L	3.8903
11	F	Y	E	V	G	A	N	F	L	3.8762
12	F	Y	E	V	G	A	S	F	L	3.8615
13	F	W	E	V	G	A	K	F	L	3.8571
14	F	W	E	V	G	A	N	F	L	3.8564
15	F	W	P	V	G	A	N	F	L	3.8155
16	F	W	D	V	G	A	Q	F	L	3.8513
17	F	Y	E	V	G	A	C	F	L	3.8457
18	F	Y	E	I	G	A	K	F	L	3.8391
19	F	F	E	I	G	A	C	F	L	3.8315
20	F	W	E	I	G	A	A	F	L	3.8306
WT	F	Q	N	Q	G	A	S	F	L	-1.0370

Compound	Compound 23									
Rank	146	147	149	150	152	153	154	155	167	Log2FC
1	F	W	E	I	G	A	S	F	L	4.4149
2	F	W	D	I	G	A	N	F	L	4.2762
3	F	W	N	I	G	A	Q	F	L	4.2715
4	F	W	D	I	G	A	A	F	L	4.2601
5	F	W	E	V	G	A	S	F	L	4.2567
6	F	W	D	V	G	A	Q	F	L	4.2369
7	F	Y	E	V	G	A	S	F	L	4.2356
8	F	W	D	I	G	A	S	F	L	4.2199
9	F	W	D	V	G	A	S	F	L	4.2017
10	F	W	P	V	G	A	Q	F	L	4.1974
11	F	W	E	Q	G	A	S	F	L	4.1915
12	F	W	E	I	G	A	H	F	L	4.1861
13	F	W	E	I	G	A	C	F	L	4.1830
14	F	Y	E	I	G	A	T	F	L	4.1715
15	F	W	D	V	G	A	A	F	L	4.1742
16	F	W	E	I	G	A	K	F	L	4.1692
17	F	Y	E	V	G	A	E	F	L	4.1417
18	F	Y	E	I	G	A	S	F	L	4.1371
19	F	W	N	V	G	A	S	F	L	4.1357
20	F	W	E	V	G	A	Q	F	L	4.1344
WT	F	Q	N	Q	G	A	S	F	L	-1.4561

Compound	Compound 33									
Rank	146	147	149	150	152	153	154	155	167	Log2FC
1	F	R	E	V	G	A	P	F	L	3.3928
2	F	R	D	I	G	A	P	F	L	3.3226
3	F	R	E	I	G	A	P	F	L	3.3123
4	F	R	P	V	G	A	P	F	L	3.2684
5	F	M	N	I	G	A	P	F	L	3.2358
6	F	R	D	V	G	A	P	F	L	3.2095
7	F	M	E	I	G	A	A	F	L	3.1949
8	L	Y	E	I	G	F	T	C	L	3.1905
9	L	C	P	I	G	F	Y	C	L	3.1590
10	L	C	P	I	G	F	T	C	L	3.1410
11	F	Y	E	I	G	A	Q	F	L	3.1346
12	F	M	E	I	G	A	P	F	L	3.1328
13	F	M	E	V	G	A	P	F	L	3.1157
14	F	G	P	V	G	A	F	F	L	3.1139
15	F	Y	E	V	G	A	C	F	L	3.1049
16	L	Y	E	I	G	F	S	C	L	3.0997
17	L	Y	P	I	G	F	S	C	L	3.0902
18	F	M	P	I	G	A	P	F	L	3.0896
19	F	M	P	V	G	A	P	F	L	3.0869
20	F	M	P	V	G	A	A	F	L	3.0783
WT	F	Q	N	Q	G	A	S	F	L	-1.2556

Compound	DMSO Control									
Rank	146	147	149	150	152	153	154	155	167	Log2FC
1	L	W	P	I	G	F	F	C	L	3.7923
2	L	W	P	I	G	F	L	C	L	3.6101
3	L	W	P	I	G	F	P	C	L	3.5229
4	L	W	P	I	G	F	Y	C	L	3.4893
5	L	W	P	I	G	F	I	C	L	3.3061
6	L	W	P	I	G	F	D	C	L	3.1185
7	L	W	P	I	G	F	M	C	L	3.0653
8	L	W	P	I	G	F	W	C	L	3.0491
9	L	W	E	I	G	F	F	C	L	3.0278
10	L	W	P	I	G	F	C	C	L	2.9859
11	L	W	N	Q	G	F	I	C	L	2.9385
12	L	W	N	Q	G	F	F	C	L	2.9343
13	L	W	E	I	G	F	I	C	L	2.8293
14	L	W	P	I	G	F	P	C	L	2.8090
15	L	W	N	Q	G	F	W	C	L	2.8031
16	F	T	P	I	G	H	Q	F	L	2.7099
17	F	W	P	I	G	H	G	F	L	2.6906
18	L	W	N	Q	G	F	L	C	L	2.6802
19	F	W	P	I	G	A	F	F	L	2.6417
20	L	W	P	I	G	F	V	C	L	2.5893
WT	F	Q	N	Q	G	A	S	F	L	-1.325

A	
C	
D	
E	
F	
G	
H	
I	
K	
L	
M	
N	
P	
Q	
R	
S	
T	
V	
W	
Y	

Figure S10. Table showing the 20 highest ranked mutant ZF sequences by log_2FC for compounds **1** (thalidomide), **19**, **22**, **23**, **33**, lenalidomide and a DMSO control; the WT (IKZF1/3 ZF2) sequence and log_2FC for each of the 3 compound screens are also shown for comparison.

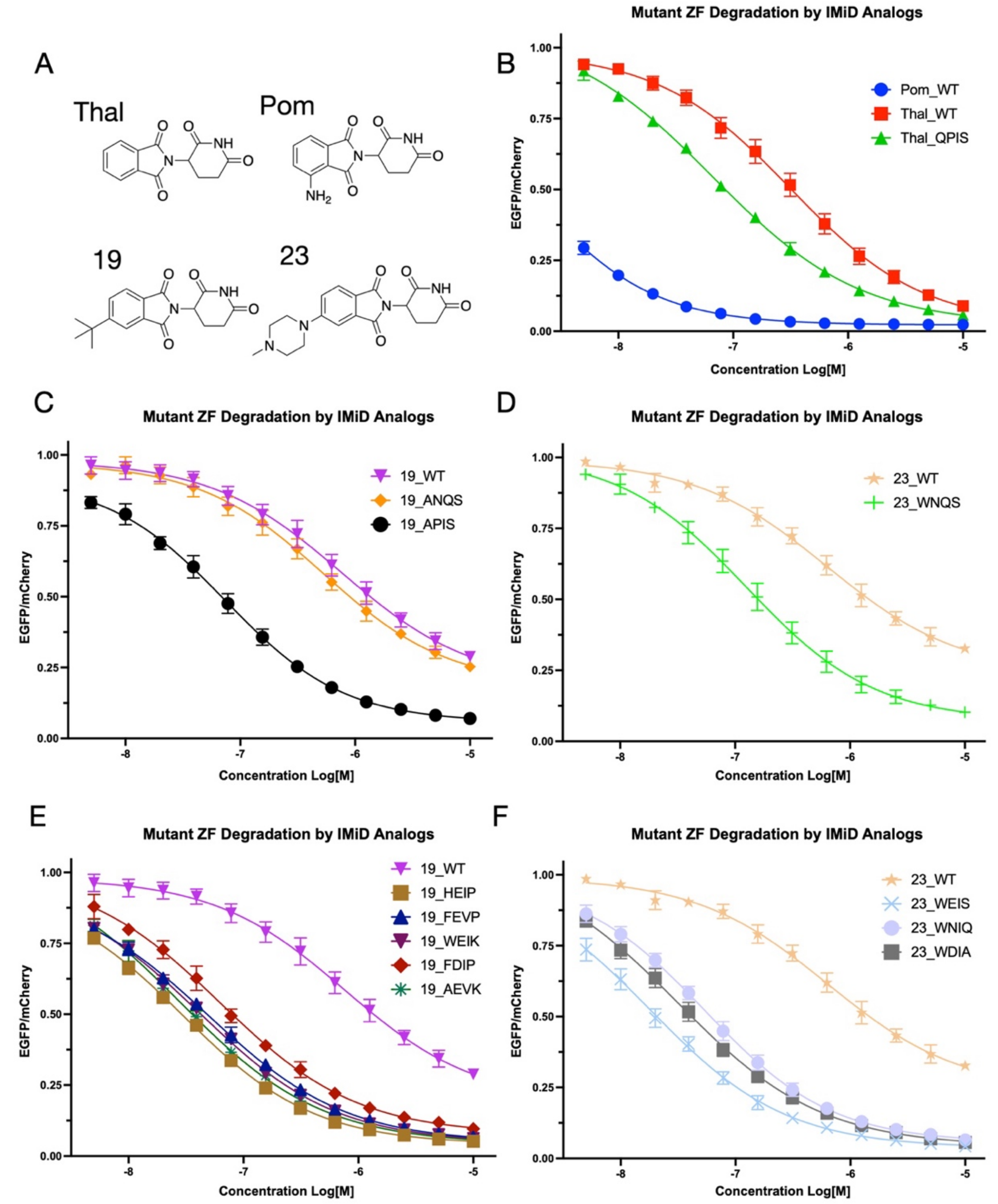

Figure S11. A. Chemical structure of four IMiD analogs. Dose-response curves in Jurkat cells after 18 h compound treatment for: **B.** pomalidomide and thalidomide degradation of the WT degron; thalidomide degradation of the QPIS degron. **C.** Compound **19** degradation of the WT,

ANQS and APIS degrons. **D.** Compound **23** degradation of the WT and WNQS degrons. **E.** Compound **19** degradation of the WT degron and five top ranking mutant degrons from the compound **19** mutant library screen. **F.** Compound **23** degradation of the WT degron and three (of five) top ranking mutant degrons from the compound **23** mutant library screen.

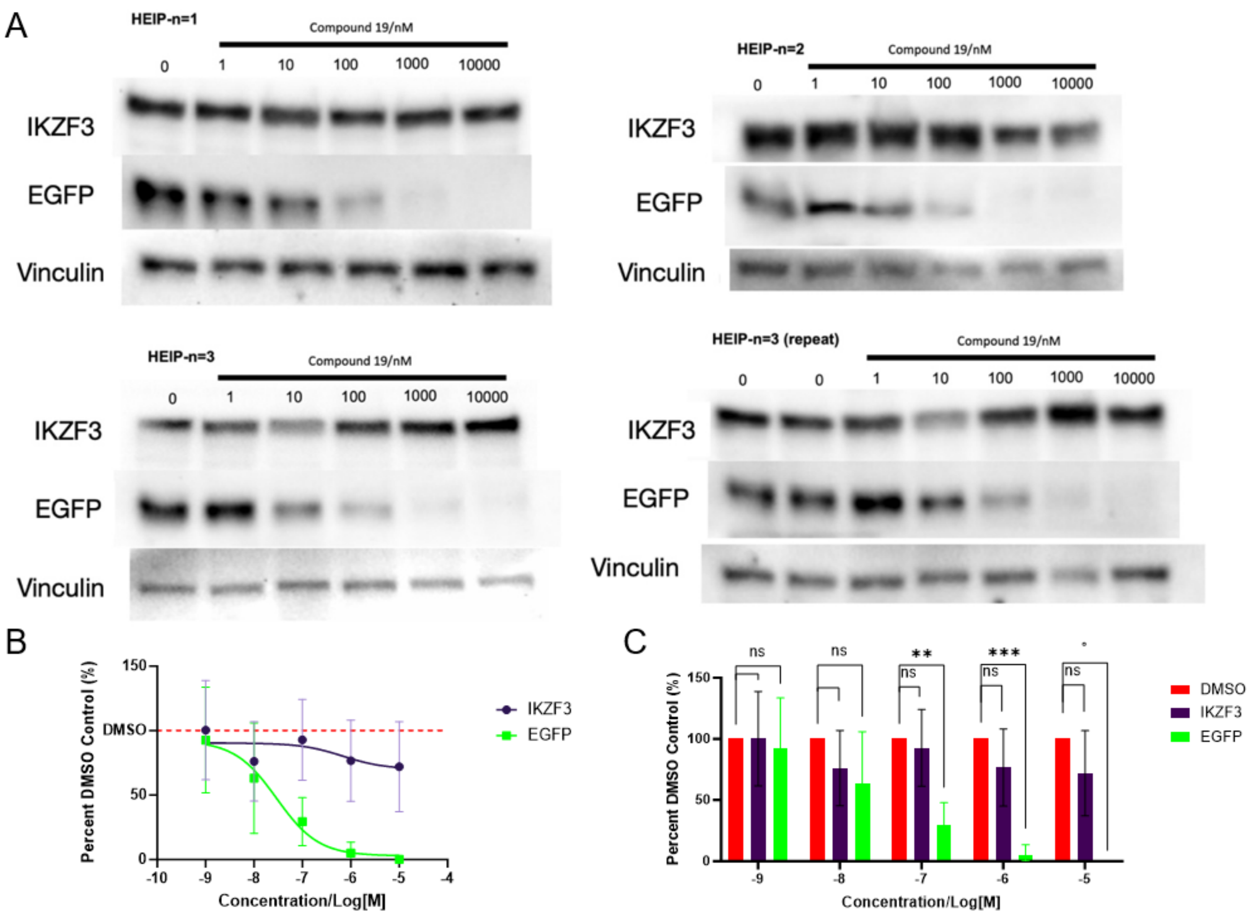

Figure S12. A. Western blot performed on transduced Jurkat cells expressing a HEIP-EGFP fusion protein degron treated for 18 h with five concentrations [as shown] of compound **19** and an untreated DMSO control. Bands show levels of IKZF3, EGFP, or vinculin. **B.** Vinculin-normalized band densitometry of the western blot in **A,** as a percentage of the DMSO control. Mean values are plotted (n=3) error bars represent SD. **C.** Multiple unpaired two-tailed t-tests were performed *p-value < 0.05; **p-value < 0.01; ***p-value < 0.001; 'ns' non-significant; ° denotes a comparison to data with the value zero, for which no p-value can be generated.

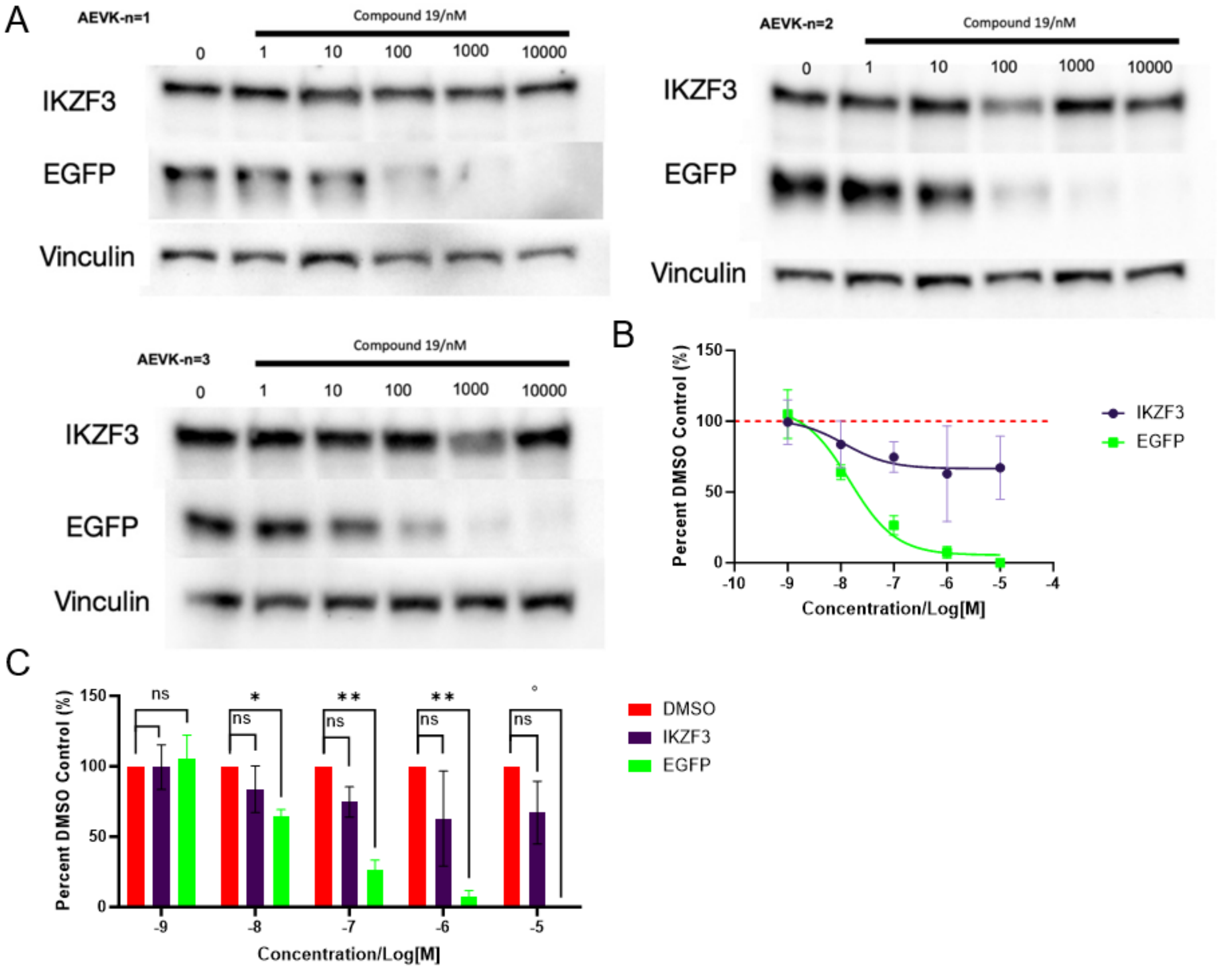

Figure S13. A. Western blots performed on transduced Jurkat cells expressing an AEVK-EGFP fusion protein degron treated for 18 h with five concentrations [as shown] of compound **19** and an untreated DMSO control. Bands show levels of IKZF3, EGFP, or vinculin. **B.** Vinculin-normalized band densitometry of the western blots in **A,** as a percentage of the DMSO control. Mean values are plotted (n=3) error bars represent S.D. **C.** Multiple unpaired two-tailed t-tests were performed *p-value < 0.05; **p-value < 0.01; ***p-value < 0.001; 'ns' non-significant; ° denotes a comparison to data of the value zero, for which no p-value can be generated.

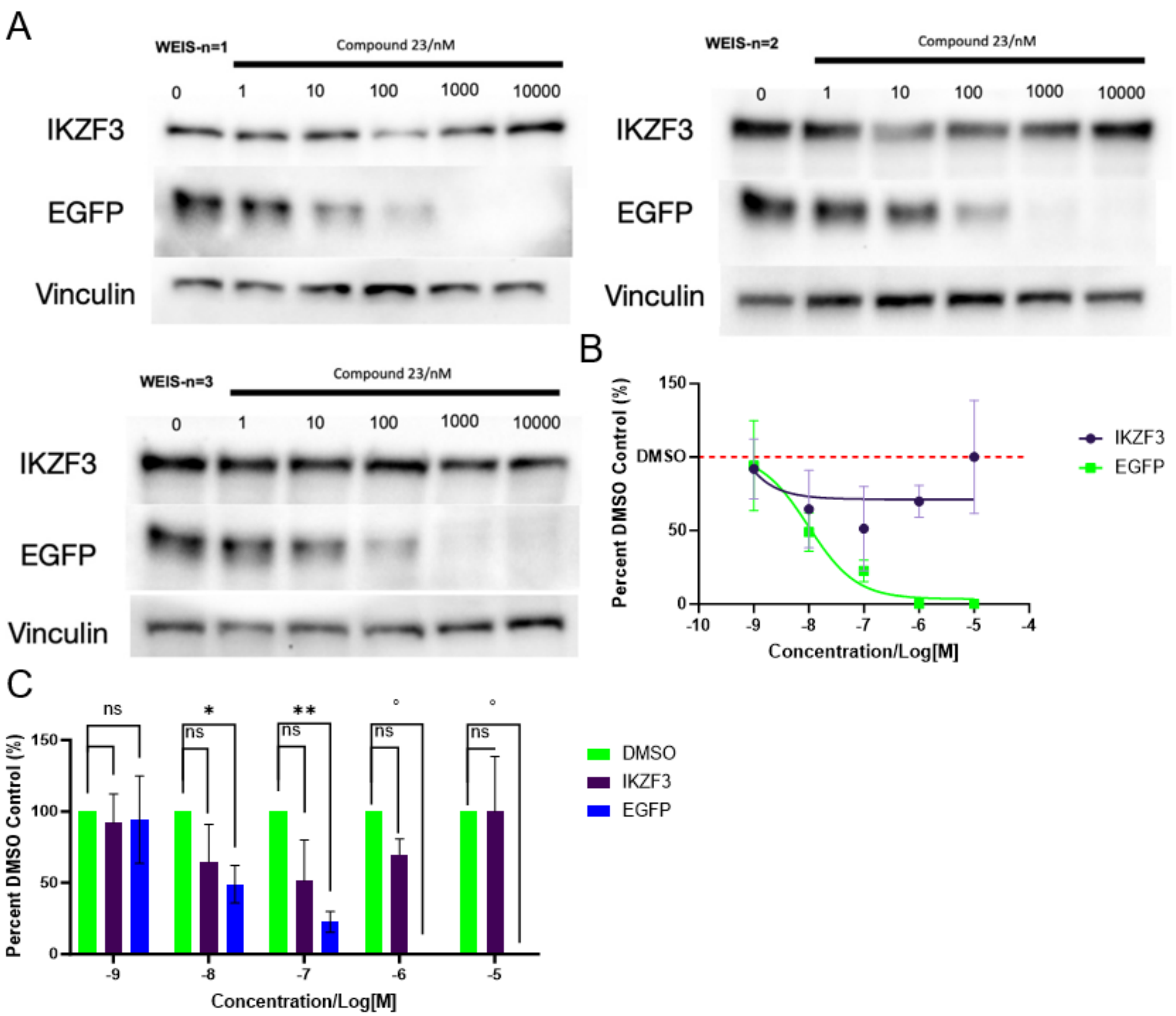

Figure S14. A. Western blots performed on transduced Jurkat cells expressing an WEIS-EGFP fusion protein degron treated for 18 h with five concentrations [as shown] of compound **23** and an untreated DMSO control. Bands show levels of IKZF3, EGFP, or vinculin. **B.** Vinculin-normalized band densitometry of the western blots in **A,** as a percentage of the DMSO control. Mean values are plotted (n=3) error bars represent S.D. **C.** Multiple unpaired two-tailed t-tests were performed *p-value < 0.05; **p-value < 0.01; ***p-value < 0.001; 'ns' non-significant; ° denotes a comparison to data of the value zero, for which no p-value can be generated.

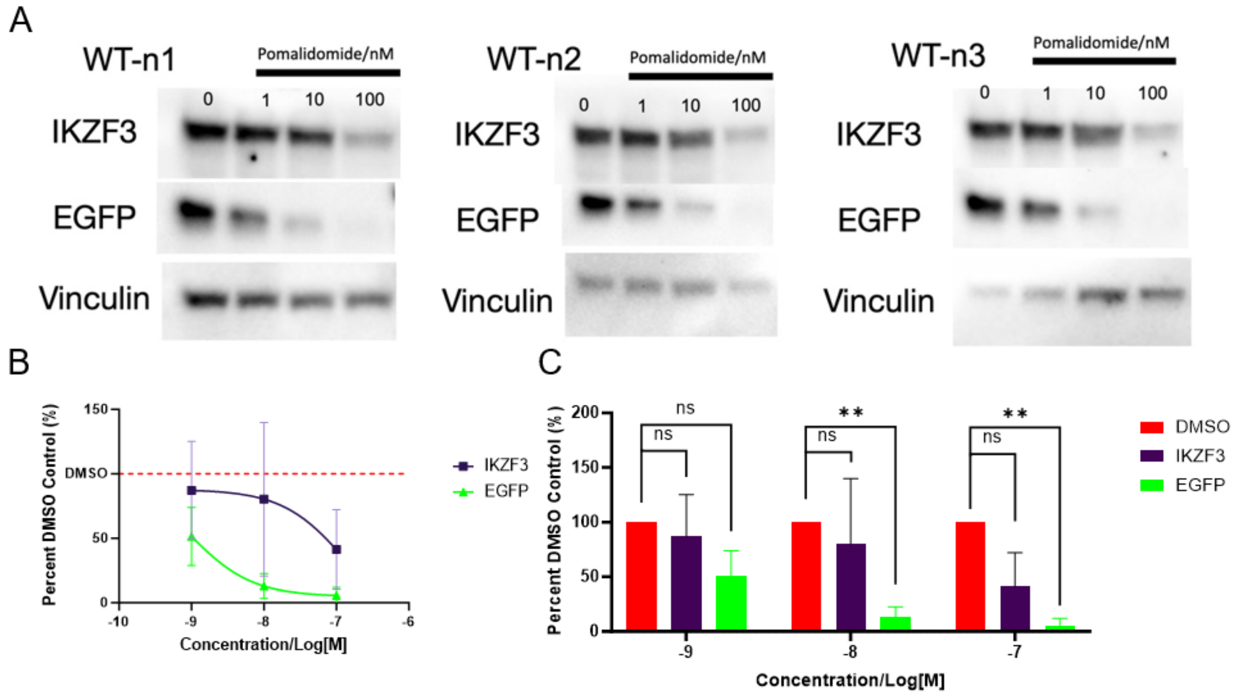

Figure S15. A. Western blots performed on transduced Jurkat cells expressing the WT-EGFP degron fusion protein, treated for 18 h with three concentrations [as shown] of compound **2** (pomalidomide) and an untreated DMSO control. Bands show levels of IKZF3, EGFP, or vinculin. **B.** Vinculin-normalized band densitometry of the western blots in **A,** as a percentage of the DMSO control. Mean values are plotted (n=3) error bars represent S.D. **C.** Multiple unpaired two-tailed t-tests were performed **p*-value < 0.05; ***p*-value < 0.01; ****p*-value < 0.001; 'ns' non-significant.

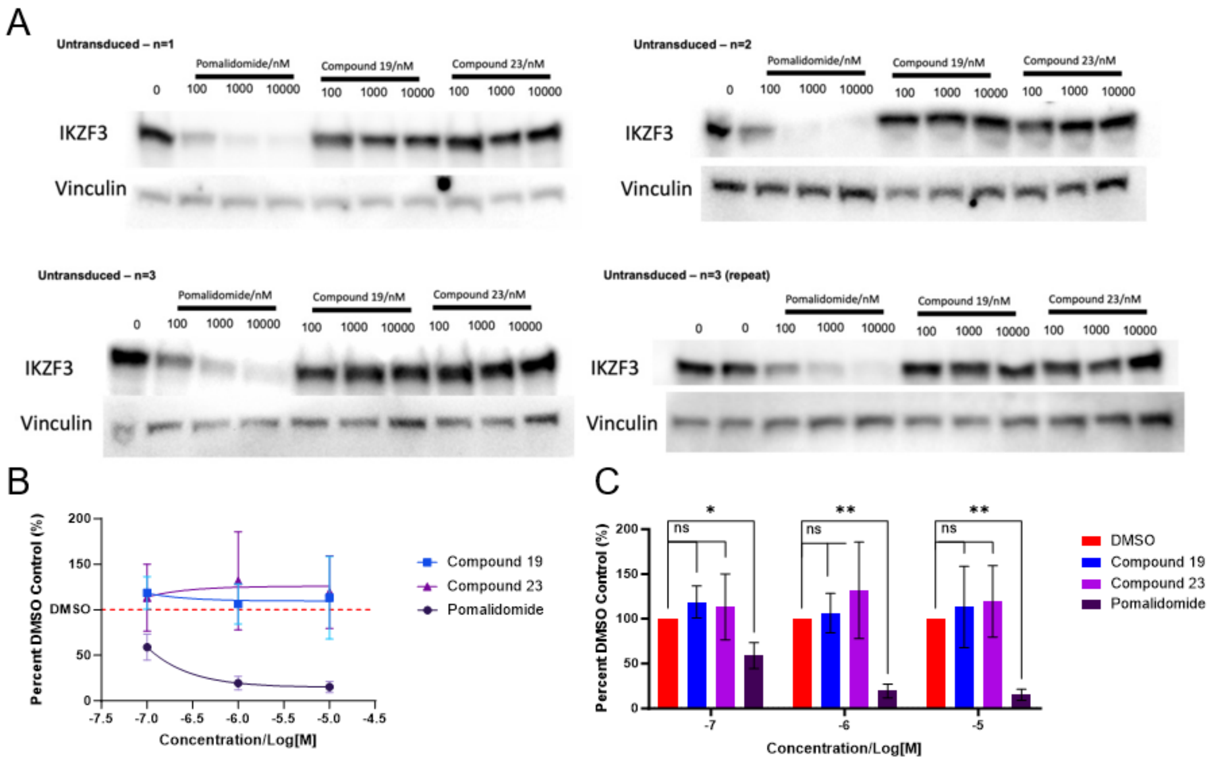

Figure S16. A. Western blots performed on untransduced Jurkat cells, treated for 18 h with three concentrations of compound **2** (pomalidomide), compound **19**, or compound **23**, and an untreated DMSO control [as shown]. Bands show levels of IKZF3, EGFP, or vinculin. **B.** Vinculin-normalized band densitometry of the Western blots in **A,** as a percentage of the DMSO control. Mean values are plotted (n=3) error bars represent S.D. **C.** Multiple unpaired two-tailed t-tests were performed **p*-value < 0.05; ***p*-value < 0.01; ****p*-value < 0.001; *ns* non-significant.

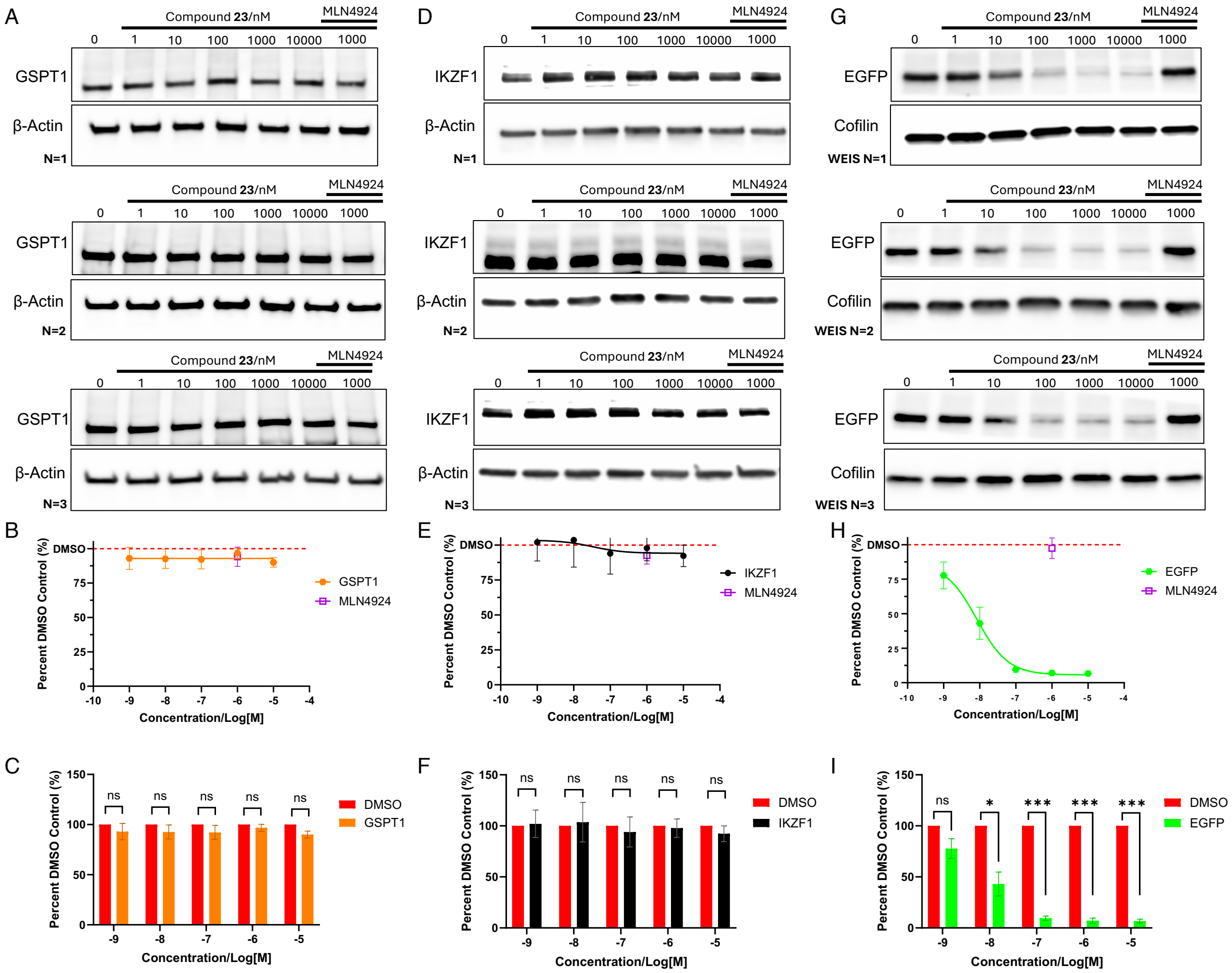

Figure S17. A. Western blots performed on transduced Jurkat cells expressing a WEIS-EGFP degron, treated for 18 hours with five concentrations [as shown] of compound **23**, 1µM MLN4924 and 1µM compound **23**, and an untreated DMSO control. Bands show levels of GSPT1 or β-actin. **B.** β-Actin-normalized band densitometry of the western blots in (**A**)**,** as a percentage of the DMSO control. **C**. Multiple unpaired two-tailed t-tests were performed on the band densitometry from (**B**), **p*-value < 0.05; ***p*-value < 0.01; ****p*-value < 0.001; *ns* non-significant. **D**. Western blots performed on transduced Jurkat cells expressing a WEIS-EGFP degron, treated for 18 hours with five concentrations [as shown] of compound **23**, 1µM MLN4924 and 1µM compound **23**, and an untreated DMSO control. Bands show levels of IKZF1 or β-actin. **E**. β-Actin-normalized band

densitometry of the western blots in (**D**), as a percentage of the DMSO control. **F**. Multiple unpaired two-tailed t-tests were performed on the band densitometry from (**E**), **p*-value < 0.05; ***p*-value < 0.01; ****p*-value < 0.001; *ns* non-significant. **G**. Western blots performed on transduced Jurkat cells expressing a WEIS-EGFP degron, treated for 18 hours with five concentrations [as shown] of compound **23**, 1μM MLN4924 and 1μM compound **23**, and an untreated DMSO control. Bands show levels of EGFP or cofilin. **H**. Cofilin-normalized band densitometry of the western blots in (**G**), as a percentage of the DMSO control. **I**. Multiple unpaired two-tailed t-tests were performed on the band densitometry from (**H**), **p*-value < 0.05; ***p*-value < 0.01; ****p*-value < 0.001; *ns* non-significant. Mean values are plotted (n=3) error bars represent S.D.

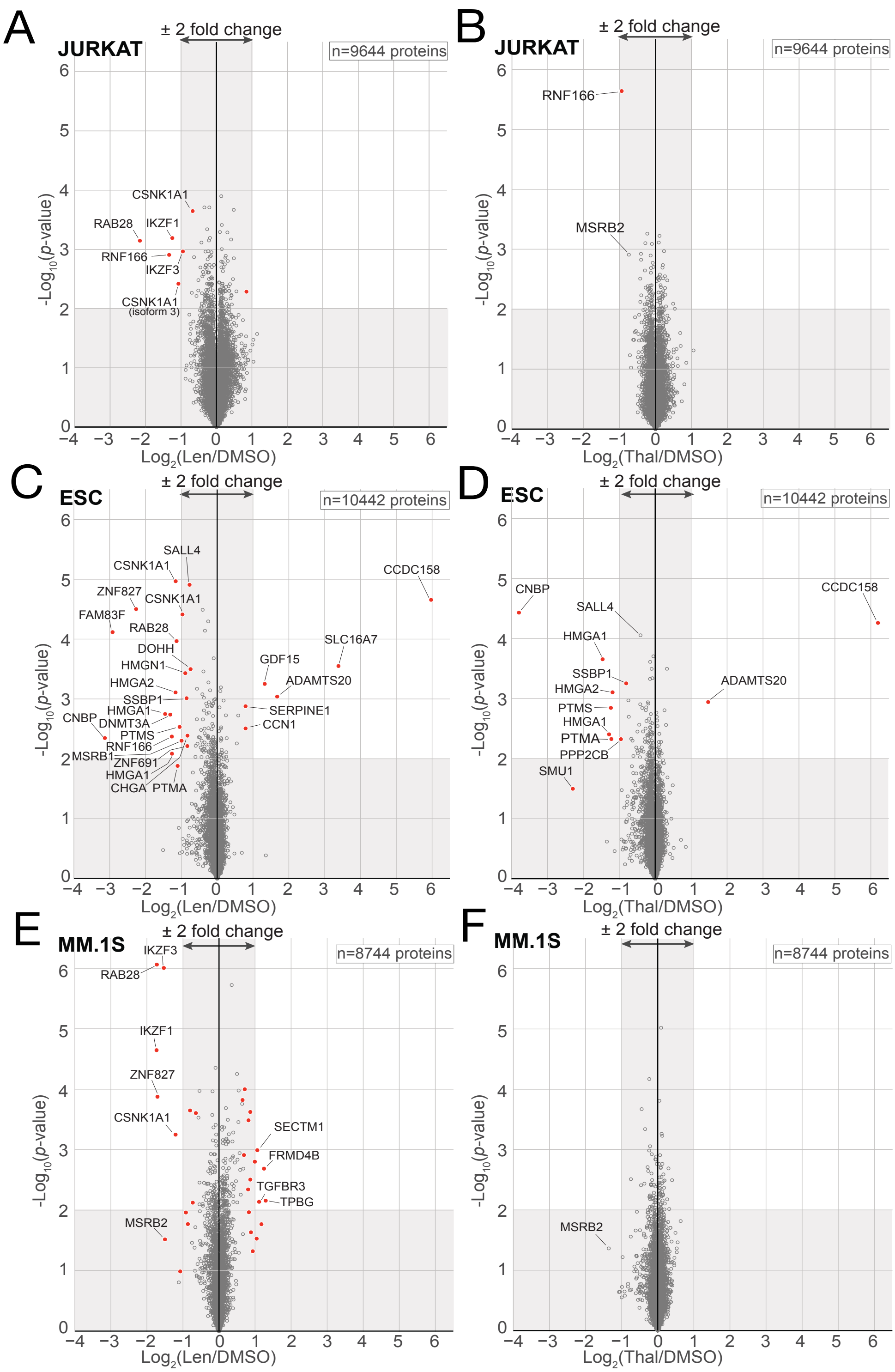

Figure S18. Quantitative proteomics profiling following cell treatment with lenalidomide (1 µM) or thalidomide (10 µM) for 16 h. **A.** Jurkat cells treated with lenalidomide. **B.** Jurkat cells treated

with thalidomide. **C.** ESCs treated with lenalidomide. **D.** ESCs treated with thalidomide. **E.** MM.1S cells treated with lenalidomide. **F.** MM.1S cells treated with thalidomide. $\log_2$FC is shown on the x-axis, and $-\log_{10}$(*p*-value) is shown on the y-axis. Values shown are the mean of three biological replicates.

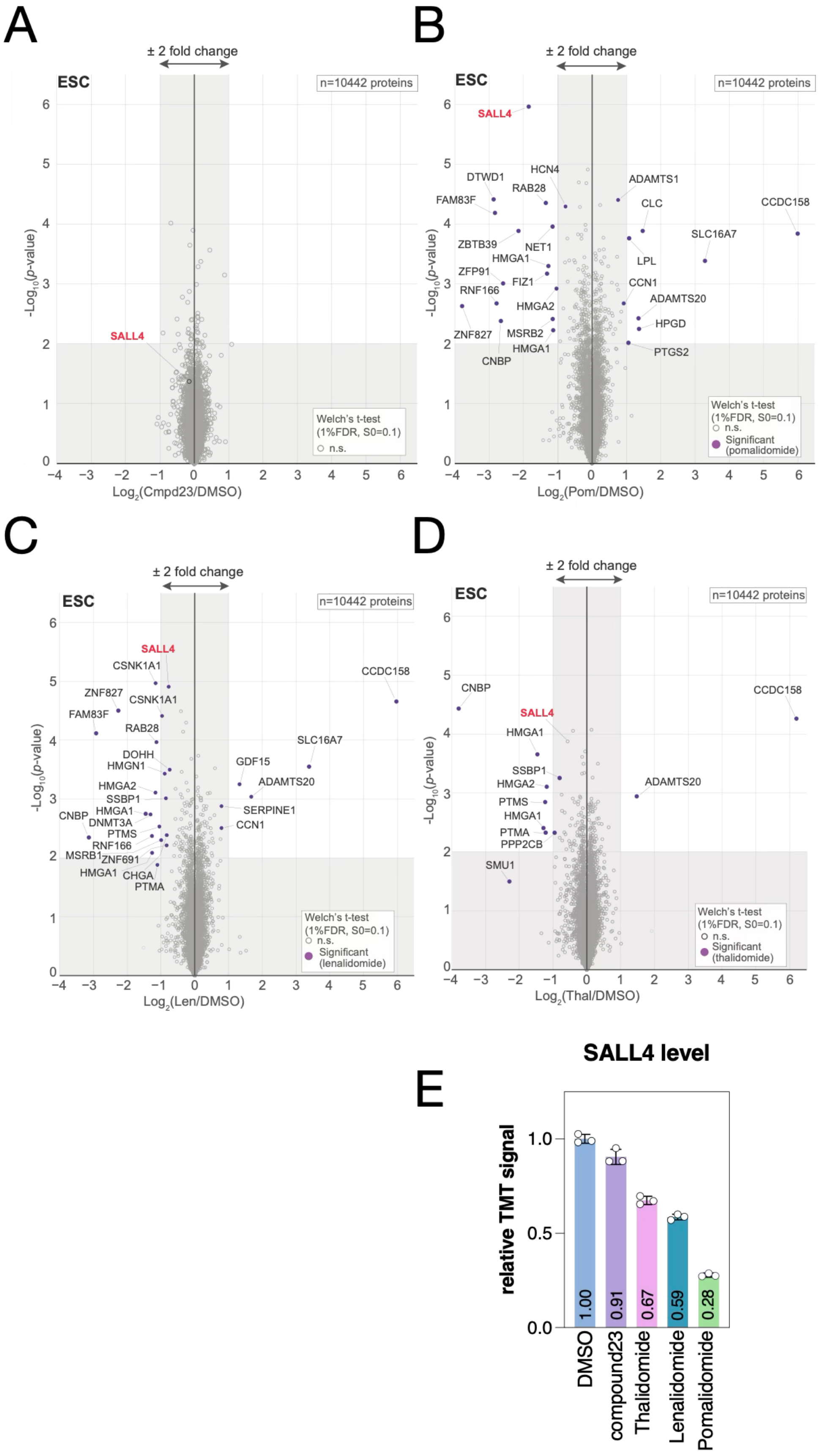
A
B
C
D
E
± 2 fold change
ESC
n=10442 proteins
-Log10(p-value)
Log2(Cmpd23/DMSO)
Log2(Pom/DMSO)
Log2(Len/DMSO)
Log2(Thal/DMSO)
SALL4
Welch's t-test (1%FDR, S0=0.1)
n.s.
Significant (pomalidomide)
Significant (lenalidomide)
Significant (thalidomide)
DTWD1
HCN4
RAB28
ADAMTS1
FAM83F
CLC
CCDC158
ZBTB39
NET1
SLC16A7
HMGA1
LPL
ZFP91
FIZ1
CCN1
RNF166
ADAMTS20
HMGA2
HPGD
ZNF827
MSRB2
PTGS2
CNBP
CSNK1A1
ZNF827
DOHH
HMGN1
GDF15
SSBP1
SERPINE1
DNMT3A
PTMS
MSRB1
ZNF691
CHGA
PTMA
SSBP1
PPP2CB
SMU1
SALL4 level
relative TMT signal
1.00
0.91
0.67
0.59
0.28
DMSO
compound23
Thalidomide
Lenalidomide
Pomalidomide

Figure S19. A-D. Quantitative proteomics profiling of human embryonic stem cells (ESC) following treatment with compound **23** (10 μM) (**A**), pomalidomide (1 μM) (**B**), lenalidomide (1 μM) (**C**), or thalidomide (10 μM) (**D**) for 16 h - (identical ESC plots to those in Figures 7 and S18, but now with SALL4 highlighted specifically in all plots). $\log_2$FC is shown on the x-axis, and $-\log_{10}$(*p*-value) is shown on the y-axis. Values shown are the mean of three biological replicates. **E.** Relative TMT levels of SALL4 for ESCs treated as described in **A-D**.

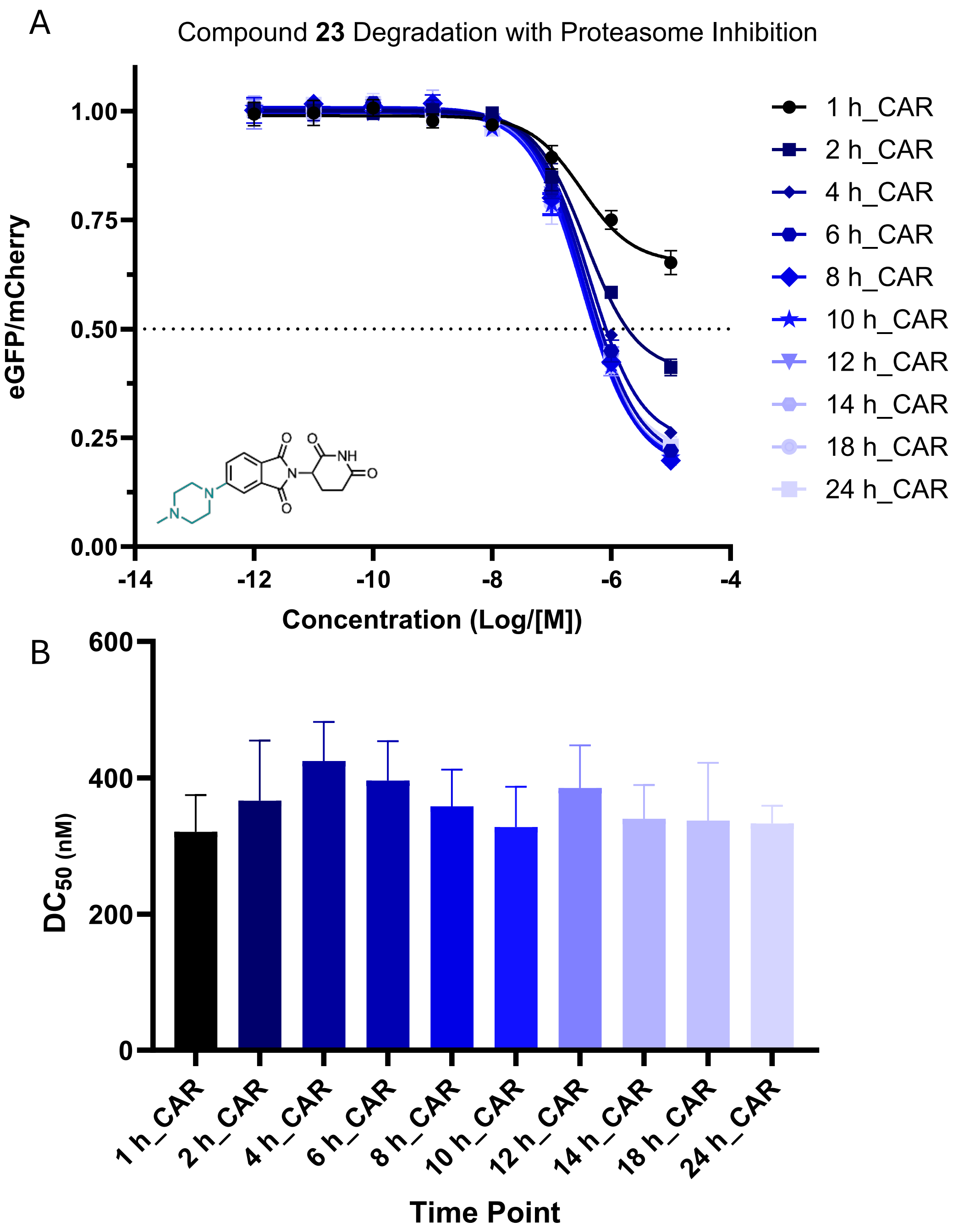

Figure S20. A. Jurkat cells were transduced with the WEIS-EGFP degron, pre-incubated for 1 h with carfilzomib (CAR) (1 μM), then incubated with a concentration curve of compound **23** (1 pM – 10 μM) and for 1-24 h [as shown] and analyzed using flow cytometry. The DMSO-normalized

ratiometric values of EGFP/mCherry are shown. **B.** The half maximal (DC_{50}) values from the assay in (**A**) are shown. Mean and SD values are plotted from three biological replicates.

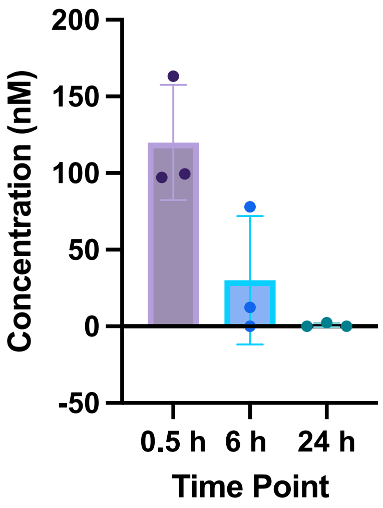

Figure S21. Assessing crude cellular stability of compound in **23** in mammalian cells using HPLC. Jurkat cells were incubated with 25 µM of compound **23** for 0.5 h, 6 h, or 24 h. Cells were washed with PBS and lysed with MeOH, clarified, and analyzed by HPLC. Compound **23** presence in the lysate was assessed using HPLC at a *UV/Vis* absorbance of 190 nm. After 0.5 h we observed 130 nM of compound remaining, 30 nM at 6 h, and almost no compound remaining at 24 h. Data are mean ± SD (*N*=3).

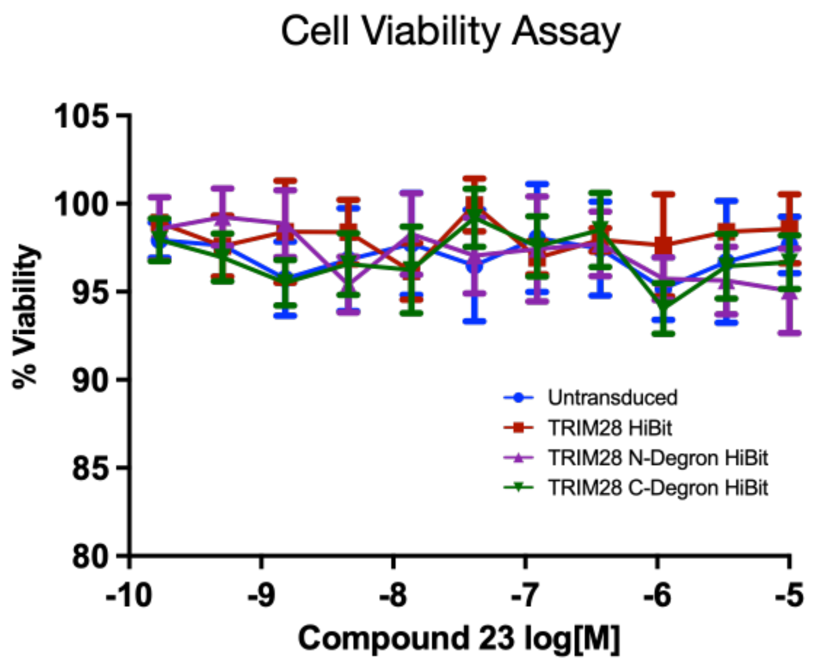

Figure S22. CellTiter-Glo assay to assess cell viability of Jurkat cells lentivirally transduced with TRIM28 with either N-terminal or C-terminal WEIS degron tag (or untagged control) and HiBit peptide tag, then incubated (alongside untransduced Jurkat cells) with a concentration curve of compound **23** (100 pM – 10 μM) for 24 h.

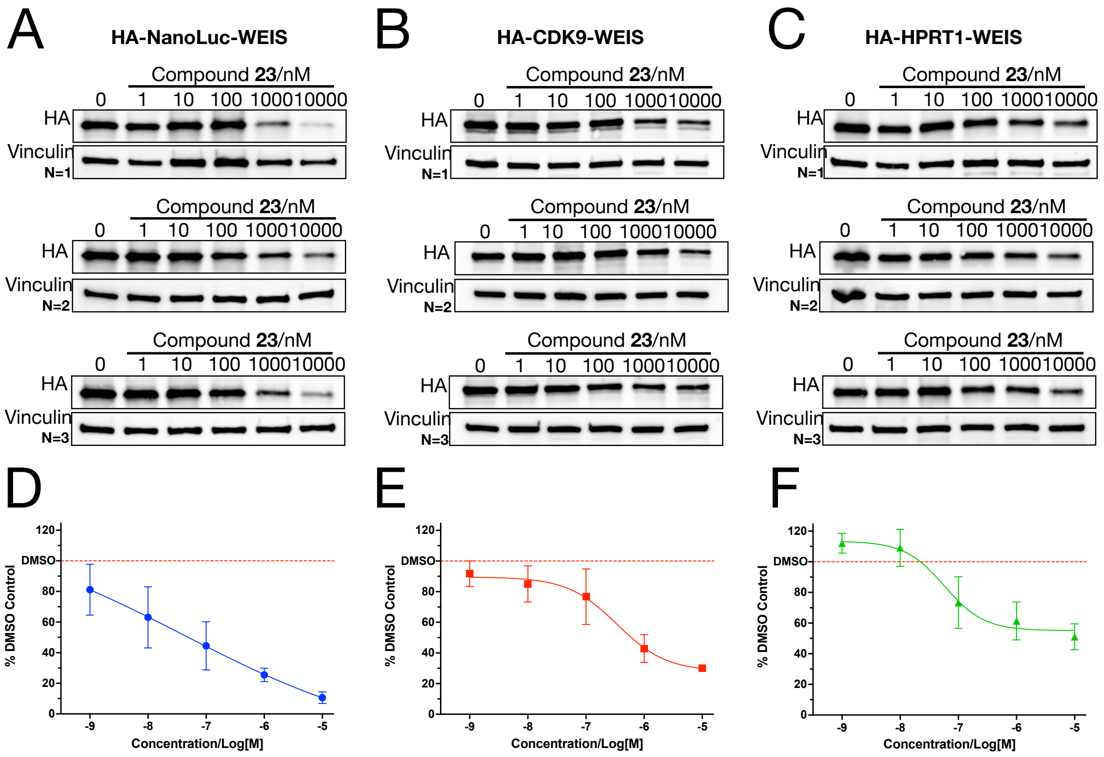

Figure S23. HEK293T cells were lentivirally transduced with one of three POI fusion constructs – NanoLuc (**A**), CDK9 (**B**) or HPRT1 (**C**) – with an N-terminal HA tag and a C-terminal WEIS degron, then incubated with a concentration curve of compound **23** (1 nM – 10 µM) and an untreated control (DMSO) for 18 h then analyzed using HA antibody immunoblotting (three biological replicates). Vinculin-normalized band densitometry of the blots in **A**, **B** and **C** as a percentage of the DMSO control shown in **D**, **E** and **F** respectively. Mean values are plotted (n=3) and error bars represent S.E.M.

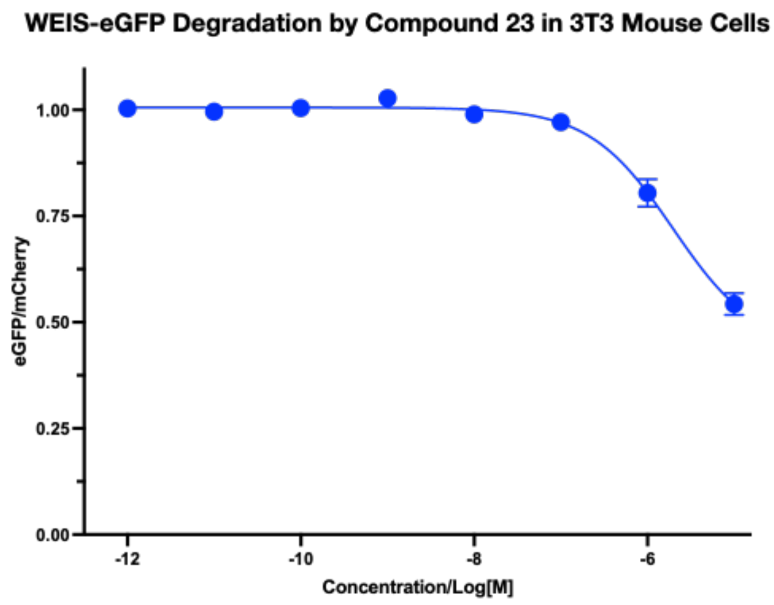

Figure S24. Dose response curve for 3T3 mouse cells lentivirally transduced with WEIS-EGFP-IRES-mCherry incubated with a concentration curve of compound **23** (1pM – 10 μM) for 18 h and analyzed by flow cytometry.

Data S1 (separate file).

Raw pooled library screen data.

Data S2 (separate file).

log_2FC data for six compounds and a DMSO control against 8380 library mutant ZF degrons. log_2FC values are arranged according to residue 154 on the x-axis, and values are arranged according to residues 146/153/155, 147, 149 and 150 on the y-axis. Color scale shows high log_2FC as orange, representing high sequence enrichment in the EGFP-low population, and low log_2FC as blue, representing low sequence occurrence. A log_2FC score of zero, colored as tan, represents equal representation of a sequence in the 'degraded' cell population and the remaining cell population after FACS.

Data S3 (separate file).

Raw proteomics data - all quantified proteins and the associated TMTpro reporter ratio to control channels used for quantitative analysis.

Materials and Methods

Chemistry Experimental Section

Reagents and solvents used were of commercially available reagent grade quality from Alfa Aesar, Fluorochem, Merck (formally known as Sigma-Aldrich), or Tokyo Chemical Industry, and were used without further purification unless stated. Anhydrous solvents were obtained from an MBRAUN Solvent Purification Systems 5 and stored under an argon atmosphere over 3 Å molecular sieves. Concentration *in vacuo* was performed at 40 °C for organic solvents using a Buchi™ rotary evaporator. Brine refers to a saturated aqueous solution of sodium chloride. Petroleum ether refers to the fraction of light petroleum ether boiling in the range 40-60 °C. Celite® refers to Celite® 545 filter aid, treated with sodium carbonate, flux-calcined (Merck).

^1H NMR spectra were measured on a Bruker AV400 (400 MHz), a Bruker AVII 500 (500 MHz) or a Bruker AV600 (600 MHz) spectrometer in the stated solvents as a reference for the internal deuterium lock. The chemical shift data for each signal are given as δ in units of parts per million (ppm) relative to tetramethylsilane (TMS) where δ(TMS) = 0.00. The spectra are calibrated using the solvent peak with the data provided by Fulmer *et al.* [1]. The multiplicity of each signal is indicated by: s (singlet); bs (broad singlet); d (doublet); t (triplet); q (quartet); p (pentet); sept (septet); m (multiplet) or combinations thereof. The number of protons (n) for a given resonance signal is indicated by nH. Where appropriate, coupling constants (*J*) are quoted in Hz, recorded to the nearest 0.1 Hz, and were determined by analysis using Bruker TopSpin v3.2 software or MestreNova software. The mean value of identical coupling constants is reported. Spectra were assigned using COSY, HSQC and HMBC experiments as necessary. Spectra acquired at high temperature (353 K or 363 K) are indicated.

^{13}C NMR spectra were measured on a Bruker AV400 (101 MHz), a Bruker AVII 500 (126 MHz) or a Bruker AV600 (151 MHz) spectrometer in the stated solvents as a reference for the internal deuterium lock using the standard ^{13}C experiment. The chemical shift data for each signal are given as δ in units of parts per million (ppm) relative to tetramethylsilane (TMS) where δ(TMS) = 0.00. The spectra are calibrated using the solvent peak with the data provided by Fulmer *et al.* [1]. Signals are quoted to one decimal place unless peaks are indistinguishable, in which case two decimal places are used. Where appropriate, coupling constants (*J*) are quoted in Hz, recorded to the nearest 0.1 Hz, and were determined by analysis using Bruker TopSpin v3.2 software or MestreNova software. Spectra were assigned using HSQC and HMBC experiments as necessary. Spectra acquired at high temperature (353 K or 363 K) are indicated.

^{19}F NMR spectra were measured on a Bruker AVIII HD 400 (376 MHz) spectrometer in the solvent stated, and are ^{13}C decoupled. Chemical shifts are given as δ in units of parts per million (ppm), to the nearest 0.1 ppm. The multiplicity of each signal is a singlet unless stated otherwise.

Mass spectra were acquired on either an Agilent 6120 (low resolution) or Bruker microToF spectrometer (high resolution) using electrospray ionisation (ESI) from solutions of either methanol or water. *m/z* values are reported in Daltons and are followed by their percentage abundance in parentheses.

Melting points were determined using a Griffin capillary tube melting point apparatus (Registered Design No. 889339) and are uncorrected. The solvent(s) from which the sample was crystallised is given in parentheses. Dec. indicates that the sample decomposed at the stated temperature.

Infrared spectra were obtained from thin films, using a diamond ATR module on a Bruker Tensor 27 spectrometer. Absorption maxima are reported in wavenumbers (cm^{-1}) and reported as s (strong), m (medium), w (weak) or br (broad).

Analytical high performance liquid chromatography (HPLC) was performed on a PerkinElmer Flexar system with a binary LC Pump and UV/vis LC detector set at 254 nm or Agilent 1260 Infinity II® system equipped with a Poroshell 120 EC-C18 column [4 µm, 4.6 × 100 mm] with a diode array UV/vis detector. To determine compound purity, a Dionex Acclaim® 120 C18 [5 µm, 12 Å, 150 mm × 4.6 mm] reverse phase column was used with a constant flow rate of 1.5 mL min^{-1} and gradient method of 10 min from 95:5 H_2O:acetonitrile (0.1% TFA) to 5:95 H_2O:acetonitrile (0.1% TFA) with a 5 min hold. Samples injected were prepared by dissolving in methanol, water, or acetonitrile, and filtered. All samples are run with 0.1% trifluoroacetic acid or 0.1% formic acid added unless indicated.

All biologically assessed compounds had a purity of ≥95% determined using analytical HPLC.

Normal Phase silica gel analytical Thin Layer Chromatography (NP TLC) to monitor reaction progress was carried out on normal phase Merck silica gel 60 F254 aluminium-supported thin layer chromatography sheets. Visualisation was carried out using absorption of UV light (λ_{max} = 254 nm and 365 nm) or thermal development after staining in an ethanolic solution of ninhydrin or an aqueous solution of potassium permanganate.

Normal phase silica gel flash column chromatography was carried out manually on Merck Geduran® silica gel 60 (40–63 µm), eluting with solvents as supplied, under a positive pressure of compressed, gaseous, nitrogen.

Compound synthesis

2-(2,6-Dioxopiperidin-3-yl)isoindoline-1,3-dione (1)

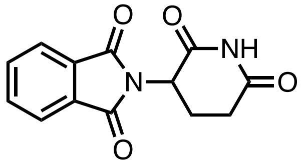

Phthalic anhydride (200 mg, 1.35 mmol, 1.0 eq), 3-aminopiperidine-2,6-dione hydrochloride (222 mg, 1.35 mmol, 1.0 eq), and NaOAc (166 mg, 2.03 mmol, 1.5 eq) were dissolved in AcOH (5 mL) and stirred at 120 °C for 16.5 h. After this time the reaction solution was cooled to room temperature (rt), and the solvent removed *in vacuo* (azeotrope with cyclohexane). The product was purified using flash column chromatography (2/98–10/90 MeOH/CH_2Cl_2) to afford the title compound (270 mg, 77%) as a colorless solid: R_f 0.60 (10/90 MeOH/CH_2Cl_2); m.p. 263–266 °C (from CH_2Cl_2) [lit. [2] 269–271 °C, lit. [3] 258–260 °C, lit. [4] 270–272 °C]; ^{1}H NMR (400 MHz, D_6-DMSO) δ 11.12 (1H, s), 7.98 – 7.85 (4H, m), 5.16 (1H, dd, *J* 12.9, 5.4 Hz), 2.96 – 2.84 (1H, m), 2.66 – 2.52 (2H, m), 2.13 – 1.97 (1H, m); LRMS *m/z* (ESI^-) 257 ($[M-H]^-$, 100%); HPLC Retention time 220 nm: 7.0 min, 98.7%; 254 nm: 7.0 min, 100.0%. These data are in good agreement with the literature values.[2]

4-Amino-2-(2,6-dioxopiperidin-3-yl)isoindoline-1,3-dione (2)

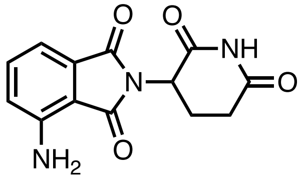

2-(2,6-Dioxopiperidin-3-yl)-4-nitroisoindoline-1,3-dione (497 mg, 1.64 mmol, 3.0) and palladium on carbon (10% *w/w*; 50 mg, 0.47 mmol, 1.0 eq) were dissolved in DMF (20 mL) and heated to 35 °C under an atmosphere of H_2 for 16 h. After this time the mixture was cooled to rt and filtered through Celite®. The DMF was removed from the filtrate *in vacuo* (azeotrope with toluene). The residual light green powder was dissolved in 20 mL of EtOAc and heated under reflux at 77 °C for 30 min. After this time, the solution was cooled, filtered, and the solvent was removed *in vacuo* to afford the title compound (363 mg, 81%) as a yellow, fluorescent powder: R_f 0.22 (50/50 EtOAc/petroleum ether); m.p. 290 °C – Dec. (from CH_2Cl_2) [lit. [5] 319–322 °C, lit. [6] 252 °C]; ^{1}H NMR (400 MHz, D_6-DMSO) δ 11.07 (1H, s), 7.47 (1H, dd, *J* 8.5, 7.0 Hz), 7.05 – 6.97 (2H, m), 6.51 (2H, s), 5.04 (1H, dd, *J* 12.9, 5.4 Hz), 2.94 – 2.82 (1H, m), 2.64 – 2.51 (2H, m), 2.07 – 1.96 (1H, m); LRMS *m/z* (ESI$^-$) 272 ([M−H]$^-$, 100%); HPLC Retention time 220 nm: 6.4 min, 99.9%; 254 nm: 6.4 min, 100.0%. These data are in good agreement with the literature values.[7]

5-Amino-2-(2,6-dioxopiperidin-3-yl)isoindoline-1,3-dione (3)

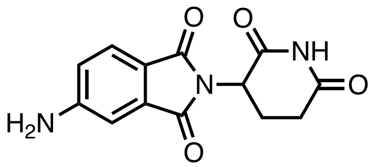

2-(2,6-Dioxopiperidin-3-yl)-5-nitroisoindoline-1,3-dione (691 mg, 2.28 mmol, 3.0 eq) and palladium on carbon (10% *w/w*; 70 mg, 0.74 mmol, 1.0 eq) were dissolved in DMF and heated to 35 °C under an atmosphere of argon. H_2 (g) was then bubbled through the solution for 10 min and the reaction was stirred at rt for 16 h under an atmosphere of H_2 (g). The mixture was then filtered through Celite® and the residue washed with toluene. The DMF was removed from the filtrate *in vacuo* (azeotrope with toluene). The residual dark green powder was dissolved in 25 mL of EtOAc and heated under reflux at 77 °C for 30 min. The solution was cooled, filtered, and the solvent was removed *in vacuo* to afford a yellow, fluorescent powder (369 mg, 52%): R_f 0.40 (10% MeOH: CH_2Cl_2); m.p. >300 °C – Dec. (from CH_2Cl_2) [lit. [8] 318–320 °C, lit. [9] 320–322 °C]; ^{1}H NMR (400 MHz, D_6-DMSO) δ 11.06 (1H, s), 7.52 (1H, d, *J* 8.3 Hz), 6.94 (1H, d, *J* 2.1 Hz), 6.83 (1H, dd, *J* 8.3, 2.1 Hz), 6.55 (2H, s), 5.01 (1H, dd, *J* 12.8, 5.5 Hz), 2.94 – 2.80 (1H, m), 2.62 – 2.52 (2H, m), 2.04 – 1.93 (1H, m); LRMS *m/z* (ESI$^-$) 272 ([M−H]$^-$, 100%); HPLC Retention time 220 nm: 6.2 min, 98.7%; 254 nm: 6.2 min, 100.0%. These data are in good agreement with the literature values.[10]

2-(2,6-Dioxopiperidin-3-yl)-4-hydroxyisoindoline-1,3-dione (4)

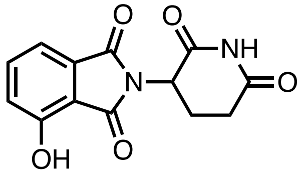

3-Hydroxyphthalic anhydride (2.00 g, 12.2 mmol, 1.0 eq), 3-aminopiperidine-2,6-dione hydrochloride (2.01 g, 12.2 mmol, 1.0 eq) and NaOAc (1.50 g, 18.3 mmol, 1.5 eq) were dissolved in AcOH (50 mL) and heated at 140 °C under reflux for 17.5 h. The AcOH was removed *in vacuo* (azeotrope with cyclohexane). The residue was dissolved in water and extracted with EtOAc, washed with water and brine, then dried with sodium sulfate, and filtered. The EtOAc was removed *in vacuo* to afford a salmon-pink solid (1.14 g, 34%). No further purification was required: R_f 0.31 (5/95 MeOH/CH_2Cl_2); m.p. 257–259 °C – Dec. (from CH_2Cl_2) [lit. [11] 243–244 °C, lit. [9] 275–276 °C, lit. [12] 281–282 °C]; ^{1}H NMR (400 MHz, D_6-DMSO) δ 11.19 (1H, s), 11.08 (1H, s), 7.65 (1H, dd, *J* 8.4, 7.2 Hz), 7.32 (1H, dd, *J* 7.2, 0.8 Hz), 7.25 (1H, dd, *J* 8.4, 0.8 Hz), 5.07 (1H, dd, *J* 12.8, 5.4 Hz), 2.95 – 2.81 (1H, m), 2.64 – 2.50 (2H, m), 2.08 – 1.96 (1H, m); LRMS *m/z* (ESI^-) 273 ($[M-H]^-$, 100%); HPLC Retention time 220 nm: 6.2 min, 97.0%; 254 nm: 6.2 min, 100%. These data are in good agreement with the literature values.[13]

2-(2,6-Dioxopiperidin-3-yl)-5-hydroxyisoindoline-1,3-dione (5)

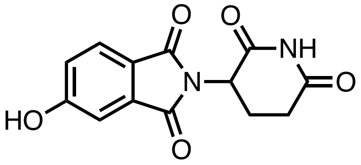

4-Hydroxyphthalic acid (246 mg, 1.35 mmol, 1.0 eq), 3-aminopiperidine-2,6-dione hydrochloride (222 mg, 1.35 mmol, 1.0 eq) and NaOAc (166 mg, 2.03 mmol, 1.5 eq) were dissolved in AcOH (5 mL) and heated at 120 °C under reflux for 3 h. The AcOH was removed *in vacuo* and the residue purified using flash column chromatography (5% MeOH/CH_2Cl_2) to afford a colorless solid (274 mg, 74%) which fluoresces yellow when dissolved: R_f 0.43 (10% MeOH/CH_2Cl_2); m.p. >300 °C (from H_2O/MeCN) [lit. [14] 317–318 °C]; ^{1}H NMR (400 MHz, D_6-DMSO) δ 11.14 (1H, br s), 11.11 (1H, s), 7.74 (1H, dd, *J* 7.9, 0.8 Hz), 7.24 – 7.08 (2H, m), 5.08 (1H, dd, *J* 12.9, 5.3 Hz), 2.94 – 2.82 (1H, m), 2.64 – 2.51 (2H, m), 2.11 – 1.97 (1H, m). LRMS *m/z* (ESI$^-$) 273 ([M−H]$^-$, 100%); HPLC Retention time 220 nm: 6.3 min, 95.3%; 254 nm: 6.4 min, 99.7%. These data are in good agreement with the literature values.[15]

2-(2,6-Dioxopiperidin-3-yl)-4-methoxyisoindoline-1,3-dione (6)

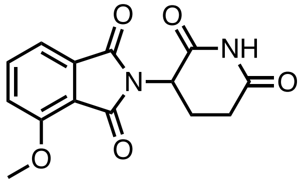

3-Methoxyphthalic acid (125 mg, 0.637 mmol, 1.0 eq), 3-aminopiperidine-2,6-dione hydrochloride (115 mg, 0.699 mmol, 1.0 eq) and NaOAc (86.0 mg, 1.05 mmol, 1.5 eq) were dissolved in AcOH (5 mL) and heated at 120 °C under reflux for 16.5 h. The AcOH was removed *in vacuo* and the residue purified using flash column chromatography (2/98 MeOH/CH_2Cl_2) to afford a colorless solid (96 mg, 47%): R_f 0.50 (2/98 MeOH/CH_2Cl_2); m.p. 276–278 °C (from CH_2Cl_2) [lit.[17] 281–282 °C]; ^{1}H NMR (400 MHz, D_6-DMSO) δ 11.10 (1H, s), 7.84 (1H, dd, *J* 8.6, 7.3 Hz), 7.53 (1H, dd, *J* 8.6, 0.7 Hz), 7.46 (1H, dd, *J* 7.3, 0.7 Hz), 5.09 (1H, dd, *J* 12.7, 5.4 Hz), 3.97 (3H, s), 2.94 – 2.83 (1H, m), 2.63 – 2.51 (2H, m), 2.11 – 1.97 (1H, m); LRMS *m/z* (ESI$^-$) 287 ([M−H]$^-$, 100%); HPLC Retention time 220 nm: 6.9 min, 96.7%; 254 nm: 6.9 min, 100.0%. These data are in good agreement with the literature value.[16]

2-(2,6-Dioxopiperidin-3-yl)-5-methoxyisoindoline-1,3-dione (7)

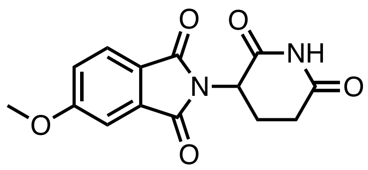

4-Methoxyphthalic acid (167 mg, 0.851 mmol, 1.0 eq), 3-aminopiperidine-2,6-dione hydrochloride (140 mg, 0.851 mmol, 1.0 eq) and NaOAc (105 mg, 1.28 mmol, 1.5 eq) were dissolved in AcOH (5 mL) and heated at 120 °C under reflux for 20 h. The AcOH was removed *in vacuo* and the residue purified using flash column chromatography (2/98 MeOH/CH_2Cl_2) to afford a colorless solid (122 mg, 50%): R_f 0.61 (2% MeOH/CH_2Cl_2); m.p. 218–219 °C (from CH_2Cl_2) [lit. [17] 218–220 °C]; ν_{max} (thin film)/cm^{-1} 1717 (C=O, s); ^{1}H NMR (400 MHz, D_6-DMSO) δ 11.11 (1H, s), 7.85 (1H, d, *J* 8.3 Hz), 7.44 (1H, d, *J* 2.3 Hz), 7.36 (1H, dd, *J* 8.3, 2.3 Hz), 5.12 (1H, dd, *J* 12.9, 5.5 Hz), 3.94 (3H, s), 2.94 – 2.83 (1H, m), 2.65 – 2.51 (2H, m), 2.09 – 1.99 (1H, m); ^{13}C NMR (151 MHz, D_6-DMSO) δ 172.7, 169.9, 166.9, 166.8, 164.7, 133.9, 125.3, 123.0, 120.3, 108.5, 56.4, 49.0, 30.9, 22.0; HRMS *m/z* (ESI$^-$) [Found: 287.0676, $C_{14}H_{11}N_2O_5$ requires [M−H$^-$] 287.0673)]; LRMS *m/z* (ESI$^-$) 287 ([M−H$^-$, 100%); HPLC Retention time 220 nm: 7.6 min, 95.5%; 254 nm: 7.6 min, 100.0%.

2-(2,6-Dioxopiperidin-3-yl)-4-fluoroisoindoline-1,3-dione (8)

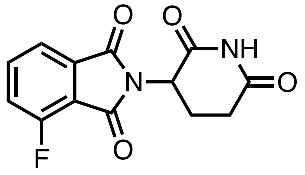

3-Fluorophthalic anhydride (2.00 g, 12.0 mmol, 1.0 eq), 3-aminopiperidine-2,6-dione hydrochloride (1.98 g, 12.0 mmol, 1.0 eq) and NaOAc (1.27 g, 18.1 mmol, 1.5 eq) were dissolved in AcOH (50 mL) and heated at 140 °C under reflux for 17 h. The mixture was cooled to rt and the AcOH removed *in vacuo* (azeotrope with cyclohexane). The residue was extracted from an aqueous solution of LiCl (0.5 M) with EtOAc. The EtOAc was removed *in vacuo* and the residue purified using flash column chromatography (60/40 EtOAc/petroleum ether) to afford the title compound (3.18 g, 67%) as a colorless solid: R_f 0.50 (10% MeOH/CH_2Cl_2); m.p. 243–247 °C (from CH_2Cl_2) [lit. [18] 255–257 °C, lit. [19] 289 °C]; ^{1}H NMR (400 MHz, D_6-DMSO) δ 11.16 (1H, s), 7.99 – 7.88 (1H, m), 7.82 – 7.68 (2H, m), 5.16 (1H, dd, *J* 12.7, 5.5 Hz), 2.95 – 2.82 (1H, m), 2.70 – 2.50 (2H, m), 2.10 – 2.02 (1H, m); ^{19}F NMR (376 MHz, D_6-DMSO) δ −114.69; LRMS *m/z* (ESI$^-$) 275 ([M−H]$^-$, 100%); HPLC Retention time 220 nm: 6.8 min, 97.5%; 254 nm: 6.8 min, 98.5%. These data are in good agreement with the literature values[20]

2-(2,6-Dioxopiperidin-3-yl)-5-fluoroisoindoline-1,3-dione (9)

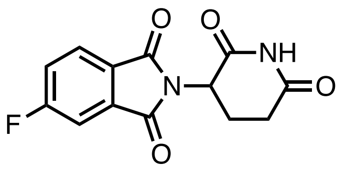

4-Fluorophthalic anhydride (2.00 g, 12.0 mmol, 1.0 eq), 3-aminopiperidine-2,6-dione hydrochloride (1.98 g, 12.0 mmol, 1.0 eq) and NaOAc (1.27 g, 18.1 mmol, 1.5 eq) were dissolved in AcOH (50 mL) and heated at 120 °C under reflux for 1.5 h. The AcOH was removed *in vacuo*, and the residue purified using flash column chromatography (5/95 MeOH/CH_2Cl_2) to afford a light lilac solid (1.92 g, 58%): R_f 0.50 (5/95 MeOH/CH_2Cl_2); m.p. 245–250 °C (from CH_2Cl_2); ^{1}H NMR (400 MHz, D_6-DMSO) δ 11.15 (1H, s), 8.02 (1H, dd, *J* 8.3, 4.5 Hz), 7.86 (1H, dd, *J* 7.5, 2.4 Hz), 7.73 (1H, ddd, *J* 9.5, 8.3, 2.4 Hz), 5.17 (1H, dd, *J* 12.7, 5.4 Hz), 2.95 – 2.83 (1H, m), 2.65 – 2.51 (2H, m), 2.12 – 2.02 (1H, m); ^{19}F NMR (376 MHz, D_6-DMSO) δ −102.35; LRMS *m/z* (ESI^-) 275 ($[M-H]^-$, 100%); HPLC Retention time 220 nm: 7.1 min, 99.5%; 254 nm: 7.1 min, 100.0%. These data are in good agreement with the literature values.[21]

4-Chloro-2-(2,6-dioxopiperidin-3-yl)isoindoline-1,3-dione (10)

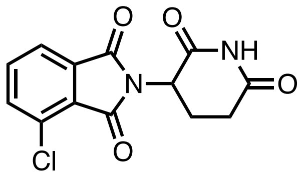

3-Chlorophthalic anhydride (155 mg, 0.849 mmol, 1.0 eq), 3-aminopiperidine-2,6-dione hydrochloride (140 mg, 0.851 mmol, 1.0 eq) and NaOAc (105 mg, 1.28 mmol, 1.5 eq) were dissolved in AcOH (5 mL) and heated at 120 °C under reflux for 20 h. After this time, the AcOH was removed *in vacuo* and the residue purified using flash column chromatography (2/98 MeOH/CH_2Cl_2) to afford a colorless solid (19 mg, 8%): R_f 0.64 (5/95 MeOH/CH_2Cl_2); m.p. 273–277 °C (from CH_2Cl_2) [lit. [16] 290–291 °C]; ^{1}H NMR (400 MHz, D_6-DMSO) δ 11.15 (1H, s), 7.95 – 7.84 (3H, m), 5.17 (1H, dd, *J* 12.8, 5.5 Hz), 2.94 – 2.85 (1H, m), 2.66 – 2.50 (2H, m), 2.12 – 2.02 (1H, m); LRMS *m/z* (ESI^-) 291 ($[M-H]^-$, 100%), 293 ($[M-H]^-$, 33%); HPLC Retention time 220 nm: 7.7 min, 100.0 %; 254 nm: 7.7 min, 96.4%. These data are in good agreement with the literature values.[16]

5-Chloro-2-(2,6-dioxopiperidin-3-yl)isoindoline-1,3-dione (11)

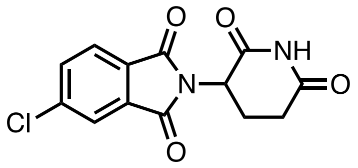

4-Chlorophthalic anhydride (155 mg, 0.849 mmol, 1.0 eq), 3-aminopiperidine-2,6-dione hydrochloride (140 mg, 0.851 mmol, 1.0 eq) and NaOAc (105 mg, 1.28 mmol, 1.5 eq) were dissolved in AcOH (5 mL) and heated at 120 °C under reflux for 20 h. The AcOH was removed *in vacuo* and the residue purified using flash column chromatography (2/98 MeOH/CH_2Cl_2) to afford a pale pink solid (68 mg, 28%): R_f 0.50 (2/98 MeOH/CH_2Cl_2); m.p. >300 °C (from CH_2Cl_2) [lit. [9] 312–313 °C]; ^{1}H NMR (400 MHz, D_6-DMSO) δ 11.15 (1H, s), 8.04 (1H, dd, *J* 1.2, 1.2 Hz), 7.97 – 7.93 (2H, m), 5.17 (1H, dd, *J* 12.8, 5.5 Hz), 2.95 – 2.85 (1H, m), 2.65 – 2.52 (2H, m), 2.11 – 2.01 (1H, m); LRMS *m/z* (ESI$^-$) 291 ([M−H]$^-$, 100%), 293 ([M−H]$^-$, 33%); HPLC Retention time 220 nm: 7.9 min, 95.2%; 254 nm: 7.9 min, 100.0%. These data are in good agreement with the literature values.[9]

4-Bromo-2-(2,6-dioxopiperidin-3-yl)isoindoline-1,3-dione (12)

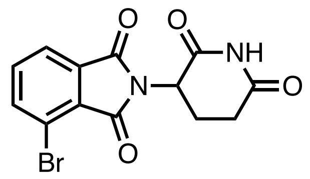

3-Bromophthalic anhydride (189 mg, 0.832 mmol, 1.0 eq), 3-aminopiperidine-2,6-dione hydrochloride (137 mg, 0.832 mmol, 1.0 eq) and NaOAc (103 mg, 1.25 mmol, 1.5 eq) were dissolved in AcOH (5 mL) and heated at 120 °C under reflux for 15 h. The AcOH was removed *in vacuo* and the residue purified using flash column chromatography (2/98 MeOH/CH_2Cl_2) to afford a colorless solid (115 mg, 41%): R_f 0.68 (5.95 MeOH/CH_2Cl_2); m.p. 283–286 °C (from CH_2Cl_2); ^{1}H NMR (400 MHz, D_6-DMSO) δ 11.15 (1H, s), 8.07 (1H, dd, *J* 8.1, 0.9 Hz), 7.94 (1H, dd, *J* 7.4, 0.9 Hz), 7.78 (1H, dd, *J* 8.1, 7.4 Hz), 5.17 (1H, dd, *J* 12.8, 5.4 Hz), 2.94 – 2.85 (1H, m), 2.66 – 2.50 (2H, m), 2.12 – 2.01 (1H, m); LRMS *m/z* (ESI^-) 335 ($[M-H]^-$, 97%), 337 ($[M-H]^-$, 100%); HPLC Retention time 220 nm: 7.9 min, 100.0%; 254 nm: 7.9 min, 98.8%. These data are in good agreement with the literature values.[22]

5-Bromo-2-(2,6-dioxopiperidin-3-yl)isoindoline-1,3-dione (13)

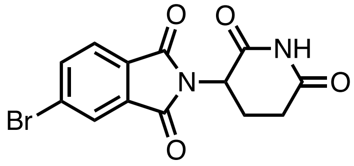

4-Bromophthalic anhydride (1.00 g, 4.18 mmol, 1.0 eq), 3-aminopiperidine-2,6-dione hydrochloride (688 mg, 4.18 mmol, 1.0 eq) and NaOAc (514 mg, 6.27 mmol, 1.5 eq) were dissolved in AcOH (30 mL) and heated at 120 °C under reflux for 1 h. The AcOH was removed *in vacuo*, and the residue purified using flash column chromatography (5/95 MeOH/CH_2Cl_2) to afford a colorless solid (585 mg, 42%): R*f* 0.53 (5/95 MeOH/CH_2Cl_2); m.p. 292–297 °C – Dec. (from CH_2Cl_2) [lit. [23] 230 °C, lit. [11] 240–241 °C, lit. [24] 303 °C]; ^{1}H NMR (400 MHz, D_6-DMSO) δ 11.15 (1H, s), 8.15 (1H, dd, *J* 1.7, 0.6 Hz), 8.10 (1H, dd, *J* 7.9, 1.7 Hz), 7.87 (1H, dd, *J* 7.9, 0.6 Hz), 5.17 (1H, dd, *J* 12.8, 5.4 Hz), 2.93 – 2.84 (1H, m), 2.65 – 2.52 (2H, m), 2.11 – 2.01 (1H, m); LRMS *m/z* (ESI^-) 335 ($[M-H]^-$, 97%), 337 ($[M-H]^-$, 100%); HPLC Retention time 220 nm: 8.3 min, 96.2%; 254 nm: 8.3 min, 97.6%. These data are in good agreement with the literature values.[15]

2-(2,6-Dioxopiperidin-3-yl)-4-iodoisoindoline-1,3-dione (14)

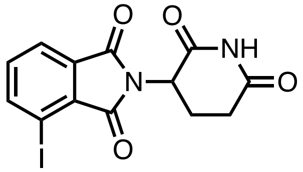

3-Iodophthalic acid (300 mg, 1.03 mmol, 1.0 eq), 3-aminopiperidine-2,6-dione hydrochloride (169 mg, 1.03 mmol, 1.0 eq) and NaOAc (126 mg, 1.54 mmol, 1.5 eq) were dissolved in AcOH (5 mL) and heated at 120 °C under reflux for 1 h. After this time the AcOH was removed *in vacuo* and the residue purified using flash column chromatography (2/98 MeOH/CH_2Cl_2) to afford a colorless solid (156 mg, 40%): R_f 0.60 (5/95 MeOH/CH_2Cl_2); m.p. 286–290 °C (from CH_2Cl_2) [lit.[23] 304–305 °C]; ^{1}H NMR (400 MHz, D_6-DMSO) δ 11.15 (1H, s), 8.28 (1H, dd, *J* 7.9, 0.9 Hz), 7.93 (1H, dd, *J* 7.4, 0.9 Hz), 7.58 (1H, dd, *J* 7.9, 7.4 Hz), 5.16 (1H, dd, *J* 12.8, 5.4 Hz), 2.94 – 2.83 (1H, m), 2.65 – 2.52 (2H, m), 2.11 – 2.01 (1H, m); LRMS *m/z* (ESI^-) 382.9 ($[M-H]^-$, 100%); HPLC Retention time 220 nm: 8.2 min, 95.9%; 254 nm: 8.2 min, 100.0%. These data are in good agreement with the literature values.[19]

2-(2,6-Dioxopiperidin-3-yl)-5-iodoisoindoline-1,3-dione (15)

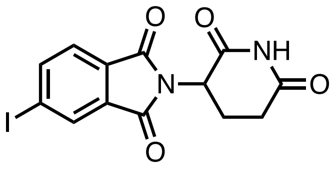

4-Iodophthalic acid (124 mg, 0.425 mmol, 1.0 eq), 3-aminopiperidine-2,6-dione hydrochloride (70 mg, 0.425 mmol, 1.0 eq) and NaOAc (53 mg, 0.646 mmol, 1.5 eq) were dissolved in AcOH (5 mL) and heated at 120 °C under reflux for 2 h. The AcOH was removed *in vacuo* and the residue purified using flash column chromatography (2/98 MeOH/CH_2Cl_2) to afford a colorless solid (93 mg, 57%): R_f 0.48 (2/98 MeOH/CH_2Cl_2); m.p. 243–245 °C (from CH_2Cl_2); ν_{max} (thin film)/cm^{-1} 1726 (C=O, s); ^{1}H NMR (400 MHz, D_6-DMSO) δ 11.14 (1H, s), 8.31 – 8.24 (2H, m), 7.69 (1H, d, *J* 8.1 Hz), 5.15 (1H, dd, *J* 12.9, 5.4 Hz), 2.93 – 2.82 (1H, m), 2.65 – 2.51 (2H, m), 2.10 – 2.00 (1H, m); ^{13}C NMR (151 MHz, D_6-DMSO) δ 172.7, 169.7, 166.8, 165.9, 143.5, 132.7, 131.8, 130.4, 124.9, 102.8, 49.1, 30.9, 21.9; HRMS *m/z* (ESI$^-$) [Found: 382.9538, $C_{13}H_8IN_2O_4$ requires [M−H$^-$] 382.9534)]; LRMS *m/z* (ESI$^-$) 382.9 ([M−H]$^-$, 100%); HPLC Retention time 220 nm: 8.6 min, 96.6%; 254 nm: 8.6 min, 98.7%.

2-(2,6-Dioxopiperidin-3-yl)-4-methylisoindoline-1,3-dione (16)

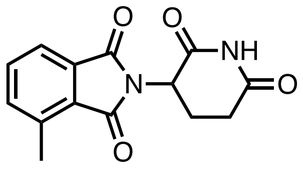

3-Methylphthalic anhydride (200 mg, 1.20 mmol, 1.0 eq), 3-aminopiperidine-2,6-dione hydrochloride (203 mg, 1.20 mmol, 1.0 eq) and NaOAc (152 mg, 1.81 mmol, 1.5 eq) were dissolved in AcOH (5 mL) and heated at 140 °C under reflux for 24 h. The residue was extracted from an aqueous solution of LiCl (0.5 M) with EtOAc. The EtOAc was removed *in vacuo*, and the residue purified using flash column chromatography (2/98 MeOH/CH_2Cl_2) to afford an off-white solid (135 mg, 40%): R_f 0.64 (10/90 MeOH/CH_2Cl_2); m.p. 284–288 °C (from CH_2Cl_2) [lit. [16] 290–292 °C]; ^{1}H NMR (400 MHz, D_6-DMSO) δ 11.11 (1H, s), 7.72 – 7.64 (3H, m), 5.13 (1H, dd, *J* 12.9, 5.4 Hz), 2.95 – 2.83 (1H, m), 2.63 (3H, s), 2.61 – 2.50 (2H, m), 2.11 – 1.99 (1H, m); LRMS *m/z* (ESI$^-$) 271 ([M−H]$^-$, 100%); HPLC Retention time 220 nm: 7.9 min, 96.7%; 254 nm: 7.9 min, 100.0%. These data are in good agreement with the literature values.[15]

2-(2,6-Dioxopiperidin-3-yl)-5-methylisoindoline-1,3-dione (17)

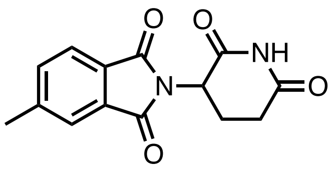

4-Methylphthalic anhydride (200 mg, 1.20 mmol, 1.0 eq), 3-aminopiperidine-2,6-dione hydrochloride (203 mg, 1.20 mmol, 1.0eq) and NaOAc (152 mg, 1.81 mmol, 1.5 eq) were dissolved in AcOH (5 mL) and heated at 120 °C under reflux for 2 h. The AcOH was removed *in vacuo* and the residue purified using flash column chromatography (5/95 MeOH/CH_2Cl_2) to afford a colorless solid (277 mg, 84%): R_f 0.60 (5/95 MeOH/CH_2Cl_2); m.p. 260–266 °C (from CH_2Cl_2) [lit. [25] 265–267 °C]; ^{1}H NMR (400 MHz, D_6-DMSO) δ 11.12 (1H, s), 7.88 (1H, d, J 7.7), 7.83 (1H, q, J 0.8), 7.76 (1H, dq, J 7.7, 0.8), 5.18 – 5.09 (1H, m), 2.96 – 2.82 (1H, m), 2.65 – 2.52 (5H, m), 2.11 – 2.00 (1H, m); LRMS *m/z* (ESI^-) 271 ($[M-H]^-$, 100%); HPLC Retention time 220 nm: 7.8 min, 98.7%; 254 nm: 7.8 min, 98.4%. These data are in good agreement with the literature values.[15]

2-(2,6-Dioxopiperidin-3-yl)-5-(trifluoromethyl)isoindoline-1,3-dione (18)

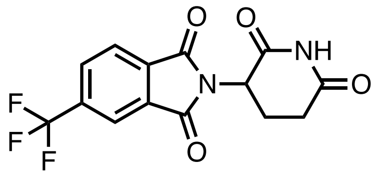

4-(Trifluoromethyl)phthalic acid (200 mg, 0.854 mmol, 1.0 eq), 3-aminopiperidine-2,6-dione hydrochloride (140 mg, 0.851 mmol, 1.0 eq) and NaOAc (105 mg, 1.28 mmol, 1.5 eq) were dissolved in AcOH (5 mL) and heated at 120 °C under reflux for 20 h. After this time the AcOH was removed *in vacuo* and the residue purified using flash column chromatography (5/95 MeOH/CH_2Cl_2) to afford a pale pink solid (192 mg, 69%): R_f 0.75 (10/90 MeOH/CH_2Cl_2); m.p. 190–192 °C (from CH_2Cl_2); ν_{max} (thin film)/cm^{-1} 1730 (C=O, s); ^{1}H NMR (400 MHz, D_6-DMSO) δ 11.18 (1H, s), 8.30 (1H, s), 8.29 (1H, d, J 7.7), 8.15 (1H, d, J 7.7), 5.22 (1H, dd, *J* 12.8, 5.4 Hz), 2.96 – 2.84 (1H, m), 2.67 – 2.52 (2H, m), 2.13 – 2.03 (1H, m); ^{13}C NMR (151 MHz, D_6-DMSO) δ 172.7, 169.6, 166.0, 165.8, 134.7, 134.5 (q, $J_{C\text{-}F}$ = 32.8 Hz), 132.2, 132.0 (d, $J_{C\text{-}F}$ = 21.2 Hz), 124.5 (d, $J_{C\text{-}F}$ = 22.4 Hz), 123.2 (q, $J_{C\text{-}F}$ = 273.1 Hz), 120.4 (d, $J_{C\text{-}F}$ = 21.2 Hz), 49.3, 30.87, 21.8; ^{19}F NMR (565 MHz, $CDCl_3$) δ −56.61; HRMS *m/z* (ESI$^-$) [Found: 325.0439, $C_{14}H_8F_3N_2O_4$ requires [M−H$^-$] 325.0442)]; LRMS *m/z* (ESI$^-$) 325 ([M−H]$^-$, 100%); HPLC Retention time 220 nm: 8.5 min, 100.0%; 254 nm: 9.5 min, 100.0%.

5-(*tert*-Butyl)-2-(2,6-dioxopiperidin-3-yl)isoindoline-1,3-dione (19)

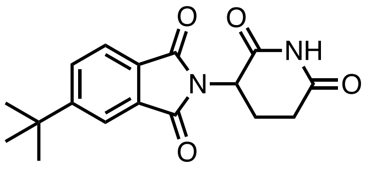

4-*tert*-Butylphthalic anhydride (200 mg, 0.979 mmol, 1.0 eq), 3-aminopiperidine-2,6-dione hydrochloride (161 mg, 0.978 mmol, 1.0 eq) and NaOAc (121 mg, 1.47 mmol, 1.5 eq) were dissolved in AcOH (5 mL) and heated at 120 °C under reflux for 21 h. The AcOH was removed *in vacuo* and the residue purified using flash column chromatography (10% MeOH/CH_2Cl_2) to afford a colorless solid (139 mg, 45%): R_f 0.69 (10/90 MeOH/CH_2Cl_2); m.p. 145–155 °C (from $CHCl_3$); ^{1}H NMR (400 MHz, D_6-DMSO) δ 11.12 (1H, s), 7.96 – 7.82 (3H, m), 5.14 (1H, dd, *J* 12.9, 5.4 Hz), 2.96 – 2.82 (1H, m), 2.65 – 2.52 (2H, m), 2.11 – 1.99 (1H, m), 1.36 (9H, s); LRMS *m/z* (ESI^-) 313 ($[M-H]^-$, 100%); HPLC Retention time 220 nm: 9.7 min, 99.8%; 254 nm: 9.7 min, 100.0%. These data are in good agreement with the literature values.[15]

2-(2,6-Dioxopiperidin-3-yl)-5-phenylisoindoline-1,3-dione (20)

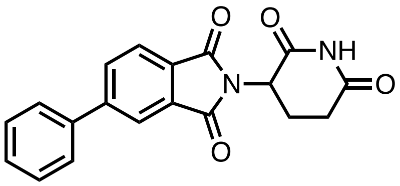

5-Phenylisobenzofuran-1,3-dione (125 mg, 0.557 mmol, 1.0 eq), 3-aminopiperidine-2,6-dione hydrochloride (92.0 mg, 0.559 mmol, 1.0 eq) and NaOAc (69.0 mg, 0.841 mmol, 1.5 eq) were dissolved in AcOH (5 mL) and heated at 120 °C under reflux for 2 h. The AcOH was removed *in vacuo* and the residue purified using flash column chromatography (2/98 MeOH/CH_2Cl_2) to afford a colorless solid (119 mg, 64%): R_f 0.65 (5/95 MeOH/CH_2Cl_2); m.p. 216–220 °C (from CH_2Cl_2) [lit. [23] 230 °C]; ^{1}H NMR (400 MHz, D_6-DMSO) δ 11.15 (1H, s), 8.23 – 8.15 (2H, m), 8.05 – 7.96 (1H, m), 7.88 – 7.81 (2H, m), 7.59 – 7.44 (3H, m), 5.19 (1H, dd, *J* 12.9, 5.5 Hz), 2.97 – 2.85 (1H, m), 2.66 – 2.52 (2H, m), 2.14 – 2.03 (1H, m); LRMS *m/z* (ESI^-) 333 ($[M-H]^-$, 100%); HPLC Retention time 220 nm: 9.5 min, 98.2%; 254 nm: 9.5 min, 99.7%. These data are in good agreement with the literature values.[23]

5-(Dimethylamino)-2-(2,6-dioxopiperidin-3-yl)isoindoline-1,3-dione (21)

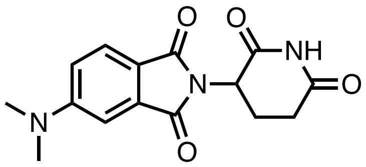

2-(2,6-Dioxopiperidin-3-yl)-5-fluoroisoindoline-1,3-dione (50.0 mg, 0.181 mmol, 1.0 eq), dimethylamine (2.0 M in THF, 2.90 mmol, 16.0 eq) and DIPEA (0.50 mL, 2.90 mmol, 16.0 eq) were dissolved in DMF (4 mL) and heated at 110 °C for 21 h. The DMF was removed by vigorously blowing nitrogen over the reaction vessel. The residual dark brown oil was purified using flash chromatography (45/55–50/50 EtOAc/petroleum ether) to afford a bright yellow powder (48 mg, 88%): R_f 0.07 (45/55 EtOAc/petroleum ether); m.p. 241–243 °C (from EtOAc) [lit. [26] 240–242 °C]; ^{1}H NMR (400 MHz, $CDCl_3$) δ 7.99 (1H, s), 7.67 (1H, d, *J* 8.6 Hz), 7.09 (1H, d, *J* 2.4 Hz), 6.82 (1H, dd, *J* 8.6, 2.4 Hz), 4.98 – 4.89 (1H, m), 3.13 (6H, s), 2.94 – 2.64 (3H, m), 2.21 – 2.08 (1H, m); LRMS *m/z* (ESI^-) 300 ($[M-H]^-$, 100%); HPLC Retention time 220 nm: 7.5 min, 98.9%; 254 nm: 7.5 min, 98.6%. These data are in good agreement with the literature values.[26]

2-(2,6-Dioxopiperidin-3-yl)-5-morpholinoisoindoline-1,3-dione (22)

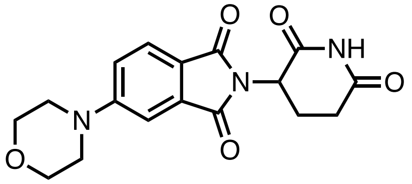

In a microwave vial, 2-(2,6-dioxopiperidin-3-yl)-5-fluoroisoindoline-1,3-dione (50.0mg, 0.181 mmol, 1.0 eq), morpholine (32 µL, 0.36 mmol, 2.0 eq) and DIPEA (0.13 mL, 0.72 mmol, 4.0 eq) were dissolved in NMP (2 mL) and heated at 110 °C for 2 h in a microwave. The NMP was removed by vigorously blowing nitrogen over the reaction vessel while heating to 60 °C. The residual dark brown oil was purified using flash chromatography (40/60 EtOAc/petroleum ether) to afford a bright yellow solid (23 mg, 37%): R_f 0.09 (50/50 EtOAc/petroleum ether); m.p. 207–208 °C (from EtOAc); ν_{max} (thin film)/cm^{-1} 1707 (C=O, s); ^{1}H NMR (400 MHz, $CDCl_3$) δ 7.95 (1H, s), 7.73 (1H, d, *J* 8.5 Hz), 7.30 (1H, d, *J* 2.4 Hz), 7.08 (1H, dd, *J* 8.5, 2.4 Hz), 4.95 (1H, dd, *J* 12.3, 5.3 Hz), 3.88 (4H, dd, *J* 6.0, 3.8 Hz), 3.41 – 3.34 (4H, m), 2.95 – 2.68 (3H, m), 2.19 – 2.09 (1H, m); ^{13}C NMR (151 MHz, $CDCl_3$) δ 170.9, 168.2, 167.9, 167.3, 155.8, 134.4, 125.5, 120.5, 118.1, 108.8, 66.5, 49.4, 47.8, 31.6, 22.9; HRMS *m/z* (ESI$^-$) [Found: 342.1098, $C_{17}H_{16}N_3O_5$ requires [M−H$^-$] 342.1095)]; LRMS *m/z* (ESI$^-$) 342 ([M−H]$^-$, 100%); HPLC Retention time 220 nm: 7.4 min, 96.5%; 254 nm: 7.4 min, 97.5%.

2-(2,6-Dioxopiperidin-3-yl)-5-(4-methylpiperazin-1-yl)isoindoline-1,3-dione (23)

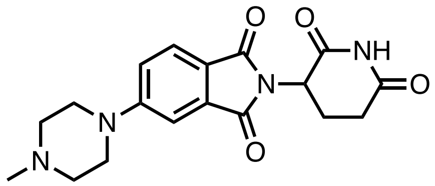

In a microwave vial, 2-(2,6-dioxopiperidin-3-yl)-5-fluoroisoindoline-1,3-dione (50.0 mg, 0.181 mmol, 1.0 eq), 1-methylpiperazine (40 µL, 0.36 mmol, 2.0 eq) and DIPEA (0.13 mL, 0.72 mmol, 4.0 eq) were dissolved in NMP (2 mL) and heated at 110 °C for 2 h in a microwave. The NMP was removed by vigorously blowing nitrogen over the reaction vessel while heating to 60 °C. The residual dark brown oil was purified using flash chromatography (2/98–10/90 MeOH/CH_2Cl_2) to afford a bright yellow solid (40 mg, 62%): R_f 0.44 (10/90 MeOH/CH_2Cl_2); m.p. 192–194 °C (from EtOAc); ν_{max} (thin film)/cm^{-1} 1709 (C=O, s); ^{1}H NMR (400 MHz, $CDCl_3$) δ 8.40 (1H, s), 7.69 (1H, d, *J* 8.6 Hz), 7.28 (1H, d, *J* 2.4 Hz), 7.06 (1H, dd, *J* 8.6, 2.4 Hz), 4.98 – 4.88 (1H, m), 3.47 – 3.40 (4H, m), 2.93 – 2.64 (3H, m), 2.62 – 2.53 (4H, m), 2.35 (3H, s), 2.19 – 2.02 (1H, m); ^{13}C NMR (151 MHz, $CDCl_3$) δ 171.0, 168.3, 168.0, 167.3, 155.6, 134.4, 125.5, 119.7, 118.2, 108.9, 54.6, 49.3, 47.6, 46.1, 31.6, 22.9; HRMS *m/z* (ESI$^-$) [Found: 355.1417, $C_{18}H_{19}N_4O_4$ requires [M−H$^-$] 355.1412)]; LRMS *m/z* (ESI$^-$) 355 ([M−H]$^-$, 100%); HPLC Retention time 220 nm: 5.1 min, 100.0%; 254 nm: 5.1 min, 100.0%.

2-(2,6-Dioxopiperidin-3-yl)-4-(ethylamino)isoindoline-1,3-dione (24)

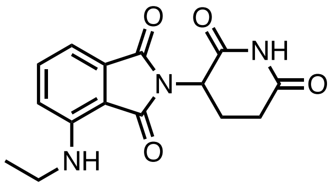

In a microwave vial, 2-(2,6-dioxopiperidin-3-yl)-4-fluoroisoindoline-1,3-dione (200 mg, 0.724 mmol, 1.0 eq), ethylamine (2.0 M in THF; 1.44 mL, 2.90 mmol, 4.0 eq) and DIPEA (0.50 mL, 2.9 mmol, 4.0 eq) were dissolved in NMP (4 mL) and heated at 110 °C for 2 h in a microwave. The NMP was removed by vigorously blowing nitrogen over the reaction vessel while heating to 60 °C. The residual oil was purified using flash column chromatography (40/60 EtOAc/petroleum ether) to afford a yellow powder (6 mg, 3%): R_f 0.30 (30/70 EtOAc/petroleum ether); m.p. 209–211 °C (from EtOAc) [lit. [26] 221–224 °C]; ^{1}H NMR (400 MHz, $CDCl_3$) δ 7.96 (1H, s), 7.50 (1H, ddd, *J* 8.5, 7.1, 0.6 Hz), 7.10 (1H, dd, *J* 7.1, 0.6 Hz), 6.89 (1H, d, *J* 8.5 Hz), 6.17 (1H, s), 4.96 – 4.87 (1H, m), 3.32 (2H, qd, *J* 7.2, 5.4 Hz), 2.98 – 2.64 (3H, m), 2.19 – 2.08 (1H, m), 1.31 (3H, t, *J* 7.2 Hz); LRMS *m/z* (ESI$^-$) 300 ([M−H]$^-$, 100%); HPLC Retention time 220 nm: 8.5 min, 97.9%; 254 nm: 8.5 min, 98.6%. These data are in good agreement with the literature values.[26]

2-(2,6-Dioxopiperidin-3-yl)-4-(prop-2-yn-1-ylamino)isoindoline-1,3-dione (25)

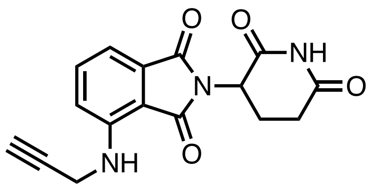

In a microwave vial, 2-(2,6-dioxopiperidin-3-yl)-4-fluoroisoindoline-1,3-dione (200 mg, 0.724 mmol, 1.0 eq), propargylamine (0.19 mL, 2.9 mmol, 4.0 eq) and DIPEA (0.50 mL, 2.9 mmol, 4.0 eq) were dissolved in NMP (4 mL) and heated at 110 °C for 2 h in a microwave. The NMP was removed by vigorously blowing nitrogen over the reaction vessel while heating to 60 °C. The residual oil was purified using flash column chromatography (30/70–50/50 EtOAc/petroleum ether) to afford a bright yellow solid (123 mg, 54%): R_f 0.55 (60/40 EtOAc/petroleum ether); m.p. 174–176 °C (from EtOAc); ^{1}H NMR (400 MHz, $CDCl_3$) δ 8.03 (1H, s), 7.57 (1H, dd, *J* 8.5, 7.2 Hz), 7.20 (1H, d, *J* 7.2 Hz), 7.03 (1H, d, *J* 8.5 Hz), 6.45 (1H, t, *J* 6.2 Hz), 4.92 (1H, dd, *J* 12.2, 5.3 Hz), 4.09 (2H, dd, *J* 6.2, 2.5 Hz), 2.94 – 2.67 (3H, m), 2.27 (1H, t, *J* 2.5 Hz), 2.18 – 2.08 (1H, m); LRMS *m/z* (ESI^-) 310 ($[M-H]^-$, 100%); HPLC Retention time 220 nm: 8.0 min, 96.7%; 254 nm: 8.1 min, 97.7%. These data are in good agreement with the literature values.[27]

4-(Benzylamino)-2-(2,6-dioxopiperidin-3-yl)isoindoline-1,3-dione (26)

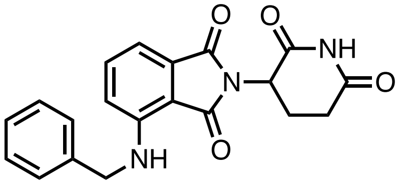

In a microwave vial, 2-(2,6-dioxopiperidin-3-yl)-4-fluoroisoindoline-1,3-dione (200 mg, 0.724 mmol, 1.0 eq), benzylamine (0.16 mL, 1.5 mmol, 2.0 eq) and DIPEA (0.50 mL, 2.9 mmol, 4.0 eq) were dissolved in NMP (4 mL) and heated at 110 °C for 2 h in a microwave. The NMP was removed by blowing nitrogen over it overnight while heating the vessel to 60 °C. The resultant oil was purified using column chromatography (50/50 EtOAc/petroleum ether) to afford a yellow solid (125 mg, 48%): R_f 0.40 (50/50 EtOAc/petroleum ether); m.p. 199–201 °C (from EtOAc) [lit.[28] 209–211 °C]; ^{1}H NMR (400 MHz, $CDCl_3$) δ 8.00 (1H, s), 7.45 (1H, dd, *J* 8.5, 7.1 Hz), 7.40 – 7.27 (5H, m), 7.12 (1H, d, *J* 7.1 Hz), 6.84 (1H, d, *J* 8.5 Hz), 6.70 (1H, t, *J* 6.0 Hz), 4.97 – 4.88 (1H, m), 4.52 (2H, d, *J* 6.0 Hz), 2.94 – 2.68 (3H, m), 2.20 – 2.10 (1H, m); LRMS *m/z* (ESI^-) 362 ($[M-H]^-$, 100%); HPLC Retention time 220 nm: 9.8 min, 99.5%; 254 nm: 9.8 min, 97.7%. These data are in good agreement with the literature values.[29]

2-(2,6-Dioxopiperidin-3-yl)-4-((pyridin-3-ylmethyl)amino)isoindoline-1,3-dione (27)

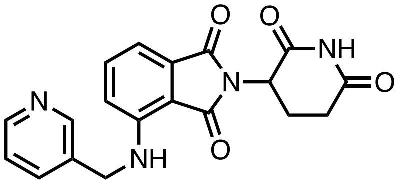

In a microwave vial, 2-(2,6-dioxopiperidin-3-yl)-4-fluoroisoindoline-1,3-dione (200 mg, 0.724 mmol, 1.0 eq), 3-picolylamine (0.15 mL, 1.5 mmol, 2.0 eq) and DIPEA (0.50 mL, 2.9 mmol, 4.0 eq) were dissolved in NMP (4 mL) and heated at 110 °C for 2 h in a microwave. The NMP was removed by vigorously blowing nitrogen over the reaction vessel while heating to 60 °C. The residual orange oil was purified using flash column chromatography (EtOAc) to afford a bright, light yellow solid (149 mg, 57%): R_f 0.31 (EtOAc); m.p. 217–220 °C (from EtOAc); ν_{max} (thin film)/cm^{-1} 1696 (C=O, s); ^{1}H NMR (400 MHz, $CDCl_3$) δ 8.63 (1H, d, *J* 2.2 Hz), 8.56 (1H, dd, *J* 4.8, 1.6 Hz), 8.05 (1H, s), 7.68 (1H, ddd, *J* 7.9, 2.2, 1.6 Hz), 7.47 (1H, dd, *J* 8.5, 7.2 Hz), 7.30 (1H, dd, *J* 7.9, 4.8 Hz), 7.16 (1H, d, *J* 7.2 Hz), 6.82 (1H, d, *J* 8.5 Hz), 6.71 (1H, t, *J* 6.0 Hz), 4.93 (1H, dd, *J* 12.2, 5.3 Hz), 4.55 (2H, d, *J* 6.0 Hz), 2.95 – 2.68 (3H, m), 2.20 – 2.12 (1H, m); ^{13}C NMR (151 MHz, $CDCl_3$) δ 170.9, 169.6, 168.3, 167.5, 149.4, 149.0, 146.4, 136.5, 134.8, 133.4, 132.7, 123.9, 117.0, 112.7, 111.1, 49.1, 44.6, 31.6, 22.9; HRMS *m/z* (ESI$^-$) [Found: 363.1100, $C_{19}H_{15}N_4O_4$ requires [M−H$^-$] 363.1099)]; LRMS *m/z* (ESI$^-$) 363 ([M−H]$^-$, 100%); HPLC Retention time 220 nm: 5.8 min, 97.7%; 254 nm: 5.8 min, 100.0%.

2-(2,6-Dioxopiperidin-3-yl)-4-((2-methylbenzyl)amino)isoindoline-1,3-dione (28)

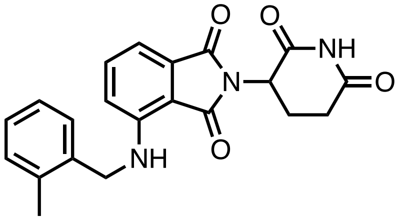

In a microwave vial, 2-(2,6-dioxopiperidin-3-yl)-4-fluoroisoindoline-1,3-dione (200 mg, 0.724 mmol, 1.0 eq), 2-methylbenzylamine (0.18 mL, 1.5 mmol, 2.0 eq) and DIPEA (0.50 mL, 2.9 mmol, 4.0 eq) were dissolved in NMP (4 mL) and heated at 110 °C for 2 h in a microwave. The NMP was removed by vigorously blowing nitrogen over the reaction vessel while heating to 60 °C. The residual orange oil was purified using flash column chromatography (40/60–60/40 EtOAc/petroleum ether) to afford a bright, light yellow solid (107 mg, 39%): R_f 0.50 (50/50 EtOAc/petroleum ether); m.p. 218–221 °C (from EtOAc); ν_{max} (thin film)/cm^{-1} 1690 (C=O, s); ^{1}H NMR (400 MHz, $CDCl_3$) δ 7.97 (1H, s), 7.48 (1H dd, *J* 8.5, 7.1 Hz), 7.24 – 7.16 (4H, m), 7.12 (1H, d, *J* 7.1 Hz), 6.85 (1H, d, *J* 8.5 Hz), 6.52 (1H, t, *J* 5.6 Hz), 4.96 – 4.86 (1H, m), 4.45 (2H, d, *J* 5.6 Hz), 2.95 – 2.66 (3H, m), 2.38 (3H, s), 2.20 – 2.09 (1H, m); ^{13}C NMR (151 MHz, $CDCl_3$) δ 171.0, 169.6, 168.3, 167.7, 146.88, 136.4, 136.1, 135.3, 132.6, 130.8, 127.9, 127.6, 126.5, 117.1, 112.1, 110.6, 49.1, 45.1, 31.6, 23.0, 19.2; HRMS *m/z* (ESI$^-$) [Found: 376.1306, $C_{21}H_{18}N_3O_4$ requires [M−H$^-$] 376.1303)]; LRMS *m/z* (ESI$^-$) 376 ([M−H]$^-$, 100%); HPLC Retention time 220 nm: 10.3 min, 95.4%; 254 nm: 10.3 min, 98.8%.

2-(2,6-Dioxopiperidin-3-yl)-4-(((4-methylpyridin-3-yl)methyl)amino)isoindoline-1,3-dione (29)

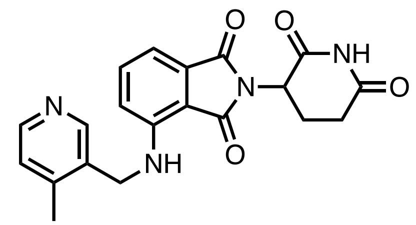

In a microwave vial, 2-(2,6-dioxopiperidin-3-yl)-4-fluoroisoindoline-1,3-dione (282 mg, 1.02 mmol, 1.0 eq), (4-methylpyridin-3-yl)methylamine (250 mg, 2.05 mmol, 2.0 eq) and DIPEA (0.71 mL, 4.1 mmol, 4.0 eq) were dissolved in NMP (4 mL) and heated at 110 °C for 2 h in a microwave. The NMP was removed by vigorously blowing nitrogen over the reaction vessel while heating to 60 °C. The residual orange oil was purified using flash column chromatography (EtOAc) to afford a bright yellow oil. TLC showed that the product was still impure; further flash column chromatography (2/98 MeOH/CH_2Cl_2) yielded a bright yellow/green glass-like oil, which was placed under vacuum for 72 h. Redissolving this in $CHCl_3$ and removal of solvent *via* nitrogen stream yielded a bright yellow powder (338 mg, 87%): R_f 0.21 (2/98 MeOH/CH_2Cl_2); m.p. 199–202 °C (from EtOAc); ν_{max} (thin film)/cm^{-1} 1694 (C=O, s); ^{1}H NMR (400 MHz, $CDCl_3$) δ 8.51 (1H, s), 8.50 (1H, s), 8.45 (1H, d, *J* 4.9 Hz,), 7.50 (1H, dd, *J* 8.5, 7.1 Hz), 7.19 – 7.10 (2H, m), 6.89 (1H, d, *J* 8.5 Hz), 6.45 (1H, t, *J* 5.6 Hz), 4.90 (1H, dd, *J* 12.1, 5.3 Hz), 4.47 (2H, d, *J* 5.6 Hz), 2.92 – 2.66 (3H, m), 2.39 (3H, s), 2.18 – 2.08 (1H, m); ^{13}C NMR (151 MHz, $CDCl_3$) δ 170.9, 169.6, 168.3, 167.5, 149.3, 148.9, 146.4, 146.3, 136.5, 132.7, 131.4, 125.8, 116.8, 112.6, 111.1, 49.1, 43.1, 31.6, 22.9, 18.8; HRMS *m/z* (ESI$^-$) [Found: 377.1253, $C_{20}H_{17}N_4O_4$ requires [M−H$^-$] 377.1255)]; LRMS *m/z* (ESI$^-$) 377 ([M−H]$^-$, 100%); HPLC Retention time 220 nm: 5.9 min, 99.5%; 254 nm: 5.9 min, 100.0%.

4-(Benzyloxy)-2-(2,6-dioxopiperidin-3-yl)isoindoline-1,3-dione (30)

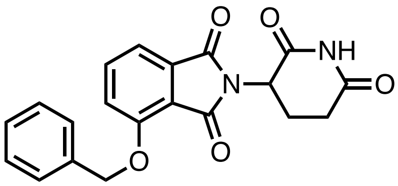

2-(2,6-Dioxopiperidin-3-yl)-4-hydroxyisoindoline-1,3-dione (100 mg, 0.364 mmol, 1.0 eq) was dissolved in dry DMF (5 mL) under an argon atmosphere. Benzyl bromide (43 µL, 0.36 mmol, 1.0 eq) and potassium carbonate (76.0 mg, 0.550 mmol, 1.5 eq) were added and the mixture stirred at room temperature for 1.5 h. The reaction mixture was dissolved in an aqueous solution of LiCl (0.5 M) and extracted with EtOAc. The organic layer was washed with water, brine, then dried with sodium sulfate, filtered, and the solvent removed *in vacuo*. The residue was purified using flash chromatography (5/95 MeOH/CH_2Cl_2). Further purification was achieved using a second round of flash chromatography (3/97 MeOH/CH_2Cl_2), as residual benzyl bromide was still present after the first column. This afforded a colorless solid (71 mg, 53%): R_f 0.65 (10/90 MeOH/CH_2Cl_2); m.p. 223–226 °C (from CH_2Cl_2) [lit. [14] 230–234 °C, lit. [30] 238–240 °C]; ^{1}H NMR (400 MHz, D_6-DMSO) δ 11.10 (1H, s), 7.83 (1H, dd, *J* 8.5, 7.3 Hz), 7.60 (1H, d, *J* 8.5 Hz), 7.55 – 7.30 (6H, m), 5.38 (2H, s), 5.09 (1H, dd, *J* 12.8, 5.4 Hz), 2.95 – 2.83 (1H, m), 2.64 – 2.51 (1H, m), 2.09 – 1.99 (1H, m); LRMS *m/z* (ESI$^-$) 363 ([M−H]$^-$, 100%); HPLC Retention time 220 nm: 9.2 min, 96.7%; 254 nm: 9.2 min, 96.8%. These data are in good agreement with the literature values.[12]

2-(2,6-Dioxopiperidin-3-yl)-4-(pyridin-3-ylmethoxy)isoindoline-1,3-dione (31)

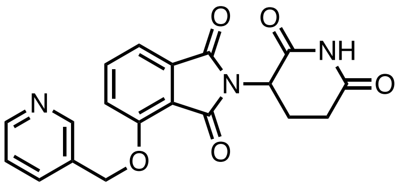

2-(2,6-Dioxopiperidin-3-yl)-4-hydroxy-1*H*-isoindole-1,3(2*H*)-dione (100 mg, 0.364 mmol, 1.0 eq) was dissolved in DMF (5 mL). Potassium carbonate (76.0 mg, 0.550 mmol, 1.5 eq) was then added and mixed. 3-(Bromomethyl)pyridine hydrobromide (82.0 mg, 0.324 mmol, 0.9 eq) was added and the mixture stirred at room temperature for 1.5 h. The DMF was removed by vigorously blowing nitrogen over the reaction vessel, and the residue was purified using flash chromatography (2/98 MeOH/CH_2Cl_2) to afford a colorless solid (69 mg, 59%): R_f 0.38 (10/90 MeOH/CH_2Cl_2); m.p. 217–220 °C (from CH_2Cl_2); ν_{max} (thin film)/cm^{-1} 1709 (C=O, s); ^{1}H NMR (400 MHz, D_6-DMSO) δ 11.12 (1H, s), 8.73 (1H, s), 8.57 (1H, d, *J* 4.8 Hz), 7.93 (1H, d, *J* 6.8 Hz), 7.86 (1H, dd, *J* 7.7, 7.7 Hz), 7.64 (1H, d, *J* 8.6 Hz), 7.50 (1H, d, *J* 8.6 Hz), 7.46 (1H, dd, *J* 6.8, 4.8 Hz), 5.42 (1H, s), 5.09 (1H, dd, *J* 12.9, 5.2 Hz), 2.93 – 2.83 (1H, m), 2.65 – 2.52 (2H, m), 2.07 – 1.99 (1H, m); ^{13}C NMR (151 MHz, D_6-DMSO) δ 172.7, 169.9, 166.7, 165.3, 155.3, 149.3, 148.7, 137.1, 135.3, 133.3, 131.8, 123.6, 120.2, 116.8, 115.8, 67.9, 48.8, 30.9, 22.0; HRMS *m/z* (ESI$^-$) [Found: 364.0940, $C_{19}H_{14}N_3O_5$ requires [M−H$^-$] 364.0939)]; LRMS *m/z* (ESI$^-$) 364 ([M−H]$^-$, 100%); HPLC Retention time 220 nm: 5.7 min, 98.3%; 254 nm: 5.7 min, 100.0%.

2-(2,6-Dioxopiperidin-3-yl)-4-((2-methylbenzyl)oxy)isoindoline-1,3-dione (32)

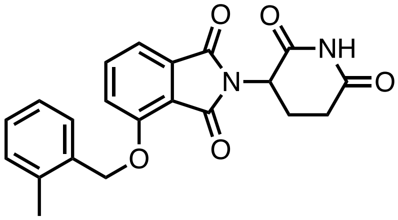

2-(2,6-Dioxopiperidin-3-yl)-4-hydroxy-1*H*-isoindole-1,3(2*H*)-dione (100 mg, 0.364 mmol, 1.0 eq) was dissolved in DMF (5 mL). Potassium carbonate (76.0 mg, 0.550 mmol, 1.5 eq) was then added and mixed. 2-Methylbenzyl bromide (44 µL, 0.32 mmol, 0.9 eq) was then added and the mixture stirred at room temperature for 1.5 h. The DMF was removed by vigorously blowing nitrogen over the reaction vessel, and the residue was purified using flash chromatography (2/98–5/95 MeOH/CH_2Cl_2) to afford a colorless solid (117 mg, 97%): R_f 0.56 (10% MeOH/CH_2Cl_2); m.p. 213–215 °C (from CH_2Cl_2); ν_{max} (thin film)/cm^{-1} 1704 (C=O, s); ^{1}H NMR (400 MHz, D_6-DMSO) δ 11.12 (1H, s), 7.85 (1H, dd, *J* 8.6, 7.2 Hz), 7.68 (1H, d, *J* 8.6 Hz), 7.55 (1H, d, *J* 7.0 Hz), 7.48 (1H, d, *J* 7.2 Hz), 7.31 – 7.18 (3H, m), 5.35 (2H, s), 5.09 (1H, dd, *J* 12.8, 5.4 Hz), 2.93 – 2.82 (1H, m), 2.63 – 2.51 (2H, m), 2.36 (3H, s), 2.08 – 1.99 (1H, m); ^{13}C NMR (151 MHz, D_6-DMSO) δ 172.7, 169.9, 166.8, 165.3, 155.5, 137.0, 136.5, 134.1, 133.3, 130.1, 128.2, 128.0, 125.8, 120.2, 116.6, 115.5, 68.8, 48.8, 30.9, 22.0, 18.5; HRMS *m/z* (ESI$^-$) [Found: 377.1144, $C_{21}H_{17}N_2O_5$ requires [M−H$^-$] 377.1143)]; LRMS *m/z* (ESI$^-$) 377 ([M−H]$^-$, 100%); HPLC Retention time 220 nm: 9.7 min, 96.2%; 254 nm: 9.7 min, 97.4%.

4-(Benzhydrylamino)-2-(2,6-dioxopiperidin-3-yl)isoindoline-1,3-dione (33)

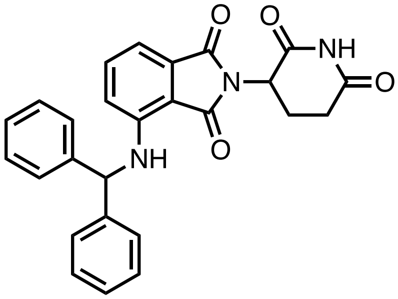

2-(2,6-Dioxopiperidin-3-yl)-4-fluoroisoindoline-1,3-dione (100 mg, 0.362 mmol, 1.0 eq), benzhydrylamine (0.25 mL, 1.5 mmol, 4.0 eq) and DIPEA (0.25 mL, 1.5 mmol, 4.0 eq) were dissolved in NMP (4 mL) and heated at 110 °C for 20 h. The NMP was removed by vigorously blowing nitrogen over the reaction vessel while heating to 60 °C. The residual dark brown oil was purified using flash column chromatography (50/50 EtOAc/petroleum ether) from which an impure product was gained; further flash column chromatography (30/70–40/60 EtOAc/petroleum ether) yielded a bright yellow powder (22 mg, 14%): R_f 0.36 (50/50 EtOAc/petroleum ether); m.p. 86–88 °C (from EtOAc); ν_{max} (thin film)/cm^{-1} 1701 (C=O, s); ^{1}H NMR (600 MHz, $CDCl_3$) δ 7.92 (1H, s), 7.41 – 7.27 (11H, m), 7.12 (1H, d, *J* 8.5 Hz), 6.86 (1H, d, *J* 5.5 Hz), 6.71 (1H, d, *J* 8.5 Hz), 5.69 (1H, d, *J* 5.5 Hz), 4.92 (1H, dd, *J* 12.5, 5.4 Hz), 2.92 – 2.68 (3H, m), 2.18 – 2.08 (1H, m); ^{13}C NMR (151 MHz, $CDCl_3$) δ 170.8, 169.6, 168.3, 167.6, 145.9, 141.44, 141.41, 136.2, 132.5, 129.22, 129.21, 128.06, 128.03, 127.35, 127.31, 118.3, 112.4, 111.0, 61.9, 49.1, 31.6, 23.0; HRMS *m/z* (ESI$^-$) [Found: 438.1460, $C_{26}H_{20}N_3O_4$ requires [M−H$^-$] 438.1459)]; LRMS *m/z* (ESI$^-$) 438 ([M−H]$^-$, 100%); HPLC Retention time 220 nm: 11.0 min, 96.0%; 254 nm: 11.1 min, 99.1%.

4-(Benzhydryloxy)-2-(2,6-dioxopiperidin-3-yl)isoindoline-1,3-dione (34)

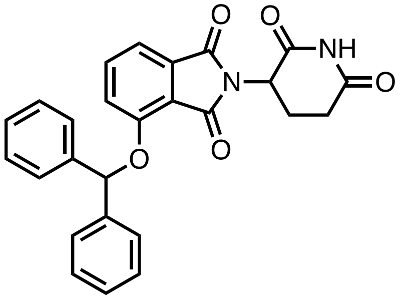

2-(2,6-Dioxopiperidin-3-yl)-4-hydroxyisoindoline-1,3-dione (100 mg, 0.364 mmol, 1.0 eq) was dissolved in dry DMF (5 mL) under an argon atmosphere. Benzhydryl bromide (95.0 mg, 0.364 mmol, 1.0 eq) and potassium carbonate (76.0 mg, 0.550 mmol, 1.5 eq) were added and the mixture stirred at room temperature for 1.5 h. The DMF was then removed by vigorously blowing nitrogen over the reaction vessel. The crude product was purified using flash chromatography (2/98 MeOH/CH_2Cl_2) to afford a colorless solid (100 mg, 62%): R_f 0.53 (10/90 MeOH/CH_2Cl_2); m.p. 201–204 °C (from CH_2Cl_2); ν_{max} (thin film)/cm^{-1} 1713 (C=O, s); ^{1}H NMR (400 MHz, D_6-DMSO) δ 11.15 (1H, s), 7.72 (1H, dd, *J* 8.6, 7.3 Hz), 7.63 – 7.56 (4H, m), 7.50 (1H, d, *J* 8.6 Hz), 7.42 (1H, d, *J* 7.3 Hz), 7.40 – 7.34 (4H, m), 7.30 – 7.23 (2H, m), 6.90 (1H, s), 5.14 (1H, dd, *J* 12.9, 5.4 Hz), 2.97 – 2.85 (1H, m), 2.67 – 2.51 (2H, m), 2.12 – 2.02 (1H, m); ^{13}C NMR (151 MHz, D_6-DMSO) δ 173.3, 170.4, 167.2, 165.9, 154.8, 141.37, 141.35, 137.2, 133.8, 129.20, 129.18, 128.30, 128.28, 126.67, 126.64, 121.7, 117.9, 116.2, 80.8, 49.3, 31.5, 22.5; HRMS *m/z* (ESI$^-$) [Found: 439.1300, $C_{26}H_{19}N_2O_5$ requires [M−H$^-$] 439.1299)]; LRMS *m/z* (ESI$^-$) 439 ([M−H]$^-$, 100%); HPLC Retention time 220 nm: 10.5 min, 95.9%; 254 nm: 10.5 min, 96.3%.

6-(2,6-Dioxopiperidin-3-yl)-5*H*-pyrrolo[3,4-*b*]pyridine-5,7(6*H*)-dione (35)

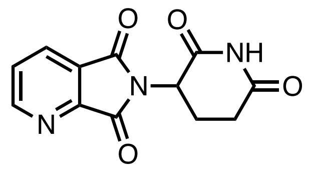

2,3-Pyridinedicarboxylic anhydride (201 mg, 1.35 mmol, 1.0 eq), 3-aminopiperidine-2,6-dione hydrochloride (222 mg, 1.35 mmol, 1.0 eq) and NaOAc (166 mg, 2.03 mmol, 1.5 eq) were dissolved in AcOH (5 mL) and heated at 120 °C under reflux for 19 h. The AcOH was removed *in vacuo* and the residue purified using flash column chromatography (5/95 MeOH/CH_2Cl_2) to afford a colorless solid (200 mg, 57%): R_f 0.50 (10/90 MeOH/CH_2Cl_2); m.p. 245–247 °C (from H_2O/MeCN) [lit. [31] 258–259 °C, lit. [32] 266–267 °C]; ^{1}H NMR (400 MHz, D_6-DMSO) δ 11.17 (1H, s), 9.04 (1H, dd, J 4.9, 1.4), 8.38 (1H, dd, J 7.7, 1.4), 7.85 (1H, dd, J 7.7, 4.9), 5.24 (1H, dd, *J* 12.9, 5.4 Hz), 2.96 – 2.84 (1H, m), 2.67 – 2.52 (2H, m), 2.13 – 2.03 (1H, m); LRMS *m/z* (ESI^-) 258 ($[M-H]^-$, 100%); HPLC Retention time 220 nm: 5.1 min, 99.7%; 254 nm: 5.2 min, 100.0%. These data are in good agreement with the literature values.[15]

2-(2,6-Dioxopiperidin-3-yl)-1*H*-pyrrolo[3,4-*c*]pyridine-1,3(2*H*)-dione (36)

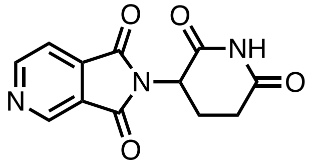

3,4-Pyridinedicarboxylic anhydride (201 mg, 1.35 mmol, 1.0 eq), 3-aminopiperidine-2,6-dione hydrochloride (222 mg, 1.35 mmol, 1.0 eq) and NaOAc (166 mg, 2.03 mmol, 1.5 eq) were dissolved in AcOH (5 mL) and heated at 120 °C under reflux for 19.5 h. The AcOH was removed *in vacuo* and the residue purified using flash column chromatography (5/95 MeOH/CH_2Cl_2) to afford a colorless solid (314 mg, 90%): R_f 0.61 (10/90 MeOH/CH_2Cl_2); m.p. 219–221 °C (from H_2O/MeCN) [lit. [33] 233–235 °C, lit. [32] 234–235 °C]; ^{1}H NMR (400 MHz, D_6-DMSO) δ 11.18 (1H, s), 9.22 – 9.11 (2H, m), 7.97 (1H, dd, *J* 4.8, 1.1 Hz), 5.22 (1H, dd, *J* 12.9, 5.4 Hz), 2.97 – 2.85 (1H, m), 2.67 – 2.50 (2H, m), 2.12 – 2.02 (1H, m); LRMS *m/z* (ESI$^-$) 258 ([M−H]$^-$, 100%); HPLC Retention time 220 nm: 5.2 min, 99.4%; 254 nm: 5.2 min, 100.0%. These data are in good agreement with the literature values.[15]

Biological Methods

Cell Culture

Jurkat cells were cultured in a media of RPMI (Gibco), 10% heat-inactivated FBS (Gibco), 1% Glutamax (Gibco), 1% MEM non-essential amino acids (Gibco), 1% sodium pyruvate (Gibco) and 1% PenStrep (Sigma). Lenti-X 293-T cells (Takara) were cultured in a media of DMEM high glucose (Gibco), 10% Tet-free FBS (Takara), 1% sodium pyruvate and 1% PenStrep. HEK293T cells were cultured in a media of DMEM, 10% heat-inactivated FBS, 1% MEM non-essential amino acids and 1% PenStrep. Cells were incubated at 37 °C and 5% CO_2. Cells expressing plasmids derived from Artichoke (Addgene #73320) or Cilantro2 (#74450) were cultured with additional puromycin (Gibco) at 1 μg/mL for HEK293T and 3T3 cells, or 78 ng/mL for Jurkat cells.

Cloning

The degradation reporter plasmid pLVX-Sprout was generated by inserting a gene fragment encoding for IKZF3 130aa-142aa 170-189aa fused to EGFP *via* a linker into pLVX-EF1a-IRES-mCherry (Takara) using XbaI/BamHI restriction sites. Gene fragments encoding for IKZF3 143aa-169aa sequences were cloned into pLVX-Sprout using the Golden Gate Assembly Kit BsmBI-v2 (New England Biolabs).

Gene fragments encoding for either HiBit-TRIM28, HiBit-TRIM28 N-terminal degron or HiBit-TRIM28 C-terminal degron were inserted into pLVX-EF1a-IRES-mCherry (Takara) using XbaI/BamHI restriction sites.

HA-POI-WEIS plasmids were assembled *via* Cilantro2 (Addgene #74450); the EGFP gene upstream of the IRES-mCherry region was removed, and a region coding for the relevant HA- and WEIS- tagged POI was inserted in its place. Using Cilantro2 as a template, primers were designed to yield two linear sequences comprising the full plasmid minus the region coding EGFP (collectively referred to as Cil2_no_eGFP). Linearisation was carried out using Phusion DNA polymerase (New England Biolabs/ThermoFisher) according to manufacturer's 50 µL rxn/3-step protocol instructions, with excess template (10-30 ng), GC buffer, 1.5 µL DMSO, 34 cycles, 20 s denaturation, 30 s annealing at 70 °C, 3 min extension, and 10 min final extension. For plasmids encoding HA-POI-WEIS constructs (for POI = NanoLuc, CDK9 or HPRT1), Gblocks encoding each HA-POI-WEIS construct were ligated with the two Cil2_no_eGFP vector halves using NEBuilder HiFi DNA Assembly Master Mix (New England Biolabs) according to the manufacturer's instructions (nebuildercalculator.neb.com), with both vector halves treated as 'vector'; incubation was carried out for 60 min and 5 µL of PCR product was carried forward for transformation into One Shot™ Stbl3™ Chemically Competent *E. coli* cells (ThermoFisher).

Lentivirus Vector Production and Transduction

The below method was used to generate lentiviral vectors for plasmids used in initial compound screening (WT and Q147A) and TRIM28 degradation (TRIM28, TRIM28 N-degron and TRIM28 C-degron). The following method was also used to generate the library lentiviral vector pool. Lenti-X 293-T cells were seeded into 100 mm TC-treated cell culture dishes (Falcon). When cells were 90% confluent, 7 µg transgene plasmid was mixed with Lenti-X™ Packaging Single Shot (VSV-G) (Takara) and incubated for 10 min at room temperature before being added to cells. After 6 h, Viralboost (Alstem) was added at a 500X dilution. After 3 days, the supernatant was harvested and centrifuged at 1000 x g for 10 minutes, then supernatant was passed through a 0.22 µm PES

filter. Lenti-X concentrator (Takara) was added at a 4X dilution, and the supernatant was incubated at 4 °C for 6 h. Supernatant was centrifuged at 4 °C 1500 x g for 45 min, supernatant was removed, and virus pellet was resuspended at a 50X concentration in cell culture media used for Jurkat cells. Lentivirus vector aliquots were frozen at −80 °C. For transductions, lentivirus vectors were added to Jurkat cells in 6 well, TC-treated, cell culture dishes (Falcon) with 3×10^6 cells per well and incubated for 2 days at 37 °C and 5% CO_2. After 2 days, the supernatant was removed and replaced with fresh media.

The following method was used to generate lentiviral vectors for plasmids tested in library hit confirmation experiments: WT, QPIS, ANQS, APIS, HEIP, FEVP, WEIK, FDIP, AEVK, WNQS, WEIS, WNIQ, and WDIA. HEK293T cells were seeded into 6 well, TC-treated, cell culture dishes (Falcon) at 7×10^5 cells per well and incubated overnight at 37 °C and 5% CO_2. 888 ng of transgene plasmid, 444 ng of psPAX2 (Addgene Cat# 12260), and 222 ng of pMD2.G (Addgene Cat#12259) were combined in 100 µL of OptiMEM (Thermo). In a separate tube, of PEIPro (Polyplus, 2 µL) was added to OptiMEM (100 µL). The solution containing PEIPro and DNA was mixed and incubated at room temperature for 15 min to create the transfection mixture. The transfection mixture was added to cells and incubated for 5–6 h at 37 °C and 5% CO_2. After 2 days, the supernatant was harvested, and fresh media was added to cells. After 24 h, the supernatant was harvested and combined with the first supernatant harvested. Supernatant was centrifuged at 2000 x g room temperature for 5 min, then the supernatant was filtered through a 0.45 µm cellulose acetate syringe filter and frozen at −80 °C. For transductions, lentivirus vectors were added to Jurkat cells in 6 well, TC-treated, cell culture dishes (Falcon) with 2.5×10^6 cells per well in media containing 4 ug/mL polybrene (Sigma). The cells were centrifuged at 500 x g for 60 min at 30 °C.

Cells were incubated for 3 days at 37 °C and 5% CO_2 prior to replacing supernatant with fresh media. (psPAX2 and pMD2.G were a gift from Didier Trono's lab).

The following methods were used to generate the following cell lines: HA-NanoLuc-WEIS, HA-CDK9-WEIS, and HA-HPRT1-WEIS HEK293T cell lines; WEIS-EGFP-IRES-mCherry (Artichoke) 3T3 cell line, and WT-EGFP-IRES-mCherry (Artichoke) and WEIS- EGFP-IRES-mCherry (Artichoke) Jurkat cell lines.

Plasmids of interest were normalized to a concentration of 20 ng/µL in 900 µL of AE Buffer and plated in 100 mm TC-treated cell culture dishes (Greiner). 108 µL of TransIT-LT1 (Mirus) was added to 4.5 mL of OptiMEM (Gibco). Next, pCMV-VSVg and pCMV-d8.91 were added to a 4 ng/µL concentration and the OptiMEM solution was plated into the cell culture dish containing normalized plasmid of interest and allowed to incubate at room temperature for 20 minutes. HEK 293T cells were then seeded into the dish at 18×10^6 cells per dish in 8.1 mL of DMEM (with FBS and PSG) and incubated at 37 °C and 5% CO_2 for 24 hours.

After 24 hours, the media was exchanged for fresh DMEM and allowed to incubate at 37°C and 5% CO_2 for an additional 24 hours. After 48 hours, the supernatant was harvested and centrifuged 1000 x G for 10 minutes. The virus was concentrated using Lenti-X Concentrator (Takara) according to manufacturer’s suggestion and reconstituted at a 100X in complete cell culture media. Aliquots of 300 µL were then stored at -80°C for later use.

For lentiviral transduction of adherent cell lines, 300 µL of each virus suspension was added dropwise to a well of a 6-well plate containing 3×10^5 cells HEK293T or 3T3 cells in complete DMEM media supplemented with 8 µg/mL protamine sulfate. Cells were allowed to incubate for 72 hours, then cells were split and seeded into a fresh 6-well plate in DMEM complete media

supplemented with 1 μg/mL puromycin. Selection media was replaced every 2-3 days until the appearance of visibly selected cells began to grow. Cells were split and expanded for future use.

For lentiviral transduction of Jurkat cells, 6-well plates were coated using Retronectin (Takara Bio) according to the manufacturer's instructions. Jurkat cells were seeded at 3×10^5 cells per well in complete RPMI media, and 300 μL of virus suspension was added dropwise into each well. Cells were allowed to incubate for 72 hours, then cells were split and seeded into a fresh 6-well plate in RPMI complete media supplemented with 78 ng/mL puromycin. Selection media was replaced every 2-3 days until the appearance of visibly selected cells began to grow. Cells were split and expanded for future use.

GFP Degradation Flow Cytometry Assay

Test compounds (10 mM stock in DMSO) were either serially diluted 10-fold to create a 6 point dilution spanning 1 nM–100 μM (final assay concentration) or serially diluted 2-fold to create a 12 point dilution spanning 5 nM–10 μM. Jurkat cells were added to 96 well U-bottom assay plates (Falcon) at 1×10^4 cells per well and treated with compounds for 18 hours at 37 °C and 5% CO_2. Cells were stained with 1μg/mL DAPI (ThermoFisher) and acquired on a Cytoflex S (Beckman Coulter). Data were analyzed using FlowJo 10.8.1 (BD).

Compound Solubility

A DMSO stock solution of IMiD analog (5 μL, 10 mM) was diluted to a volume of 100 μL with pH 7.4 PBS, equilibrated for 1 h at room temperature and filtered through Millipore Multiscreen HTS-PCF filter plates (MSSL BPC). The filtrate was quantified by suitably calibrated Charged Aerosol Detector [34].

Protein Expression and Purification

Wild-type human 6×His-TEV-Cereblon and 6×His-TEV-DDB1ΔBPB were cloned in pFastbac-HTb (Genscript), and recombinant proteins were expressed in Super 9 SFX insect cells (SH3A3187.02, Cytiva) using the BacMam baculovirus expression system (ThermoFisher). For protein purification cells were resuspended in buffer containing 500 mM NaCl (Sigma), 10% glycerol (Sigma), 50 mM Tris pH 7.7 (Sigma), 15 mM imidazole (Sigma), 90.5 mM TCEP (Sigma), 5 μL/mL protease inhibitor cocktail (Sigma) and 0.66 μL/mL benzonase (Novagen). Cells were lysed by sonication and pelleted by ultra-centrifugation. The supernatant was passed over Ni-NTA affinity resin (Qiagen) and eluted in buffer containing 500 mM NaCl, 10% glycerol, 50 mM Tris pH 7.7 and 90.5 mM TCEP supplemented with 350 mM imidazole. Proteins were further purified using size exclusion chromatography in 50 mM Tris pH 7.5, 5% glycerol, 2 mM DTT (Sigma), and 150 mM NaCl. Protein fractions were concentrated by ultrafiltration using an Ultra 10 K MWCO device (Amicon), then flash frozen in liquid nitrogen and stored at −80 °C.

CRBN Binding

Test compounds (10 mM stock in DMSO) were serially diluted 4-fold to create an 11-point dilution spanning 0.1 nM–100 μM. 100 nL of compounds were added to black 384 well low volume assay plates (Greiner) using an Echo 555 acoustic dispenser (Labcyte). 5 μL 6×His-TEV-Cereblon/6×His-TEV-DDB1ΔBPB (5 nM final assay concentration) containing Alexa647 labelled lenalidomide (50 nM final assay concentration) were added to the assay plates using a Multidrop Combi dispenser (ThermoFisher) in assay buffer (50 mM HEPES (Sigma), 150 mM NaCl (Sigma), 5% Glycerol (Sigma), 1 mM CHAPS (Sigma) and 1 mM DTT (Sigma) pH 7.4). Assay plates were centrifuged at 1000 rpm for 1 min and then incubated for 15 min at rt. 5 μL Eu-W1024-labeled

Anti-6×His antibody (1 nM final assay concentration) (Perkin Elmer) in assay buffer was added to all wells, plates were centrifuged at 1000 rpm for 1 min and then incubated for 30 min at room temperature. After excitation of europium fluorescence at 337 nm, emission at 615 nm (donor, europium) and 665 nm (acceptor, Alexa647) were recorded with a 20 µs delay to reduce background fluorescence on an Envision 2104 microplate reader (Perkin Elmer). The data were reported as a 665/615 ratio and normalized between high (no test compound) and low (no protein) controls.

Endogenous IKZF1 Immunostaining

Test compounds (10 mM stock in DMSO) were serially diluted 10-fold to create a 6-point dilution spanning 1 nM–100 µM (final assay concentration). Jurkat cells were added to 96 well U-bottom assay plates (Falcon) at 1×10^5 cells per well and treated with compounds for 18 hours at 37 °C and 5% CO_2. Following incubation, cells were stained with LIVE/DEAD™ Fixable Aqua Dead Cell Stain (Thermo) diluted 1000X in PBS for 30 min at room temperature and then incubated with Human TruStain FcX block (Biolegend) diluted 40X in PBS + 1% FBS (Gibco) for 10 min at room temperature. Intranuclear staining of IKZF1 was carried out using the Transcription Factor Buffer Set (BD Bioscience) according to the manufacturer's protocol and staining with IKZF1 AF488 antibody (clone R32-1149, BD Bioscience) diluted 40X for 30 minutes at room temperature. Cells were acquired on a Cytoflex S (Beckman Coulter) and data analysed using FlowJo 10.8.1 9 (BD).

Pooled Library Construction

A library of 8380 IKZF3 degrons was cloned into pLVX-Sprout to create the EGFP/mCherry protein degradation reporter plasmid pool. A lentivirus pool was then generated using the Lenti-X lentiviral vector production method.

Pooled Library Screen

Jurkat cells were transduced with the lentivirus vector library at a low level of <30% to maximise the number of cells with a single integration. Transductions were carried out in 6 well, TC-treated, cell culture dishes (Falcon) with 3×10^6 cells per well. Cells were incubated for 2 days at 37 °C and 5% CO_2 before the supernatant was removed and replaced with fresh media. Six days post-transduction, mCherry+ cells were isolated *via* FACS using the FACS Aria Fusion (BD) in a buffer containing PBS (Gibco), 2 mM EDTA (Gibco), 25 mM HEPES (Sigma) and 1% BSA (Sigma). After 8 days, cells were treated with DMSO, or a thalidomide analog, for 18 h at 37 °C and 5% CO_2. $EGFP_{low}$ and EGFP cells were sorted by FACS using the FACS Aria Fusion (BD). DMSO treated cells were used to set the $EGFP_{low}$ gate and dead cells were excluded from sorting by staining cells with 1 μg/mL DAPI (ThermoFisher). gDNA was isolated using the Maxwell RSC 48 (Promega). gDNA from cells that were unsorted and untreated were also isolated to determine the library representation on the day of compound treatment. Throughout the screen >600X coverage of the library was maintained. Three biological replicates were carried out with a new transduction taking place each time.

Next-Generation Sequencing (NGS)

A two-step PCR was used where the first PCR (PCR1) amplified the integrated degron sequence from gDNA and the second PCR (PCR2) added Illumina adaptors and barcodes to permit multiplexing. See **Sequences for NGS** for PCR1 and PCR2 primers. Q5 Hot Start High-Fidelity 2X Master Mix (New England Biolabs) was used for both PCRs. Sufficient gDNA was amplified in PCR1 in order to maintain >600X coverage and amplicons were purified using solid phase reversible immobilization (SPRI) beads (Beckman Coulter). Purified PCR1 products were analysed on the Tapestation 4200 (Agilent) to confirm purity prior to proceeding to PCR2. Sufficient PCR1 product was amplified in PCR2 so as to maintain >600X coverage and amplicons were run on a 2% agarose TAE gel. Target bands were excised and purified using the NucleoSpin Gel and PCR Clean up Kit (Machery Nagel). Amplicons were analysed on the Tapestation 4200 and quantified using Qubit dsDNA HS kit (ThermoFisher). PCR products were pooled and sequenced as paired-reads on the NovaSeq (Illumina) using a SP2 200 cycles kit (Illumina).

NGS Data Analysis

Sample fastq files were processed to align reads to reference degron sequences using the alignment tool Vsearch [35] to count the number of times each degron was present within each sample. Count distributions, percentage of read mapping, library coverage and replicate correlation per sample were assessed for QC purposes. Counts for each degron were then subject to Limma Voom linear regression analysis [36] to generate an estimated log fold change between replicate samples in the positively ($EGFP_{low}$) sorted fraction *vs* replicate samples in the negatively ($EGFP_{+}$) sorted fraction for each degron sequence.

Immunoblotting

General procedure (assay-specific conditions listed below): test compounds (10 mM stock in DMSO) were serially diluted 10-fold to create a dilution curve spanning either 100 nM–10 µM or 1 nM–10 µM (final assay concentration) and an untreated control, with all concentrations at 0.1% DMSO. Either Jurkat or HEK293T cells were added to T25 flasks (Falcon) at 2×10^6 or 3×10^6 cells per flask respectively and were incubated with compounds for 18 hours at 37 °C and 5% CO_2. Cells were lysed using one of two lysis buffers containing the following: 1) urea (Sigma) at either 6 M or 9 M, 75 mM Tris-HCl (Sigma), 0.15 M β-mercaptoethanol (Merck) and 10X diluted protease inhibitor cocktail (Merck or Sigma-Aldrich), or 2) RIPA buffer (Thermo) with 10X diluted protease inhibitor cocktail (Merck/Sigma-Aldrich). If lysed with urea buffer, cells lysates were sonicated for 10 seconds three times; if lysed with RIPA buffer, cell lysates were kept on ice for 10 minutes. Lysates were then centrifuged at 13,000 rpm for 10 minutes at 4 °C and the supernatants were transferred into fresh tubes. Protein concentrations were measured using either a Pierce BCA Protein Assay Kit (Thermo) if lysed with RIPA buffer, or a Bradford Protein Assay Kit (Thermo) if lysed with urea buffer. Proteins were boiled in Laemmli sample buffer (Bio-Rad) for 5 min at 100 °C and separated by SDS-PAGE (either 7.5% Mini-PROTEAN TGX gels, Bio-Rad; or 4-20% Mini-PROTEAN TGX gels, Bio-Rad) and compared to either the PageRuler Prestained Protein Ladder (Thermo) or the Precision Plus Protein Dual Color Standards (Bio-Rad). Semi-dry blotting was done on a Trans-Blot Turbo Transfer System (Bio-Rad) with the Trans-Blot Turbo RTA Mini 0.2 µm PVDF Transfer Kit (Bio-Rad) using the default mixed molecular weight mode. Membranes were blocked in 5% non-fat dry milk (Bio Rad) in Tris-buffered saline with Tween® (TBS-T). Primary antibodies were diluted (concentrations for specific assays listed below) in either 5% non-fat dry milk or 0.3% bovine serum albumin (BSA) in TBS-T. Membranes were

incubated with primary antibodies overnight at 4 °C with shaking, washed with TBS-T three times, incubated with HRP conjugated secondary antibodies (Promega) for 1 h at room temperature with shaking, washed with TBS-T three times and imaged with Clarity Western ECL substrate (Bio-Rad). Membranes were scanned with a Bio-Rad ChemiDoc XRS+ Imaging System (Bio-Rad) or an Azure 300 Chemiluminescent Imaging System (Azure Biosystems) and signal intensities were quantified using Image Lab software (Bio Rad). Band densitometry was assessed, normalized to vinculin bands, and reported as percentage of the DMSO control lane.

The following conditions were applied for blotting assays with Jurkat cells expressing ZF-EGFP constructs, blotting for IKZF3, EGFP, and a vinculin loading control.

- Cell seeding density: 2×10^6 cells per flask
- Lysis buffer: urea (9 M)
- Gel: 7.5% Mini-PROTEAN TGX
- Ladder: PageRuler Prestained Protein Ladder
- Primary antibodies: IKZF3 (D1C1E, Cell Signaling Technology Europe) 1:1000; EGFP (F56-6A1.2.3, Thermo) 1:500; and vinculin (E1E9V, Cell Signaling Technology Europe and sc-25336, Santa Cruz Biotechnologies) 1:50

The following conditions were applied for blotting assays with Jurkat cells expressing WEIS-EGFP, blotting for GSPT1 and a β-actin loading control.

- Cell seeding density: 2×10^6 cells per flask

- Lysis buffer: urea (6 M)
- Gel: 4-20% Mini-PROTEAN TGX
- Ladder: PageRuler Prestained Protein Ladder
- Primary antibodies: Anti-eRF3/GSPT1 (ab49878, Abcam) 1:1500; and β-actin (sc-69879, Santa Cruz Biotechnology) 1:1000

The following conditions were applied for blotting assays with Jurkat cells expressing WEIS-EGFP, blotting for IKZF1 and a β-actin loading control.

- Cell seeding density: 2×10^6 cells per flask
- Lysis buffer: urea (6 M)
- Gel: 4-20% Mini-PROTEAN TGX
- Ladder: PageRuler Prestained Protein Ladder
- Primary antibodies: IKZF1 (12016-1-AP, Proteintech) 1:1500; and β-actin (sc-69879, Santa Cruz Biotechnology) 1:1000

The following conditions were applied for blotting assays with Jurkat cells expressing WEIS-EGFP, blotting for EGFP and a cofilin loading control.

- Cell seeding density: 2×10^6 cells per flask
- Lysis buffer: RIPA buffer

- Gel: 4-20% Mini-PROTEAN TGX
- Ladder: PageRuler Prestained Protein Ladder
- Primary antibodies: EGFP (F56-6A1.2.3, Thermo) 1:1000; cofilin (5175S, Cell Signaling Technologies) 1:2000

The following conditions were applied for blotting assays with HEK293T cells expressing HA-POI-WEIS constructs, blotting for HA and a vinculin loading control.

- Cell seeding density: 3×10^6 cells per flask
- Lysis buffer: RIPA buffer
- Gel: 4-20% Mini-PROTEAN TGX
- Ladder: Precision Plus Protein Dual Color Standards (Bio-Rad)
- Primary antibodies: HA (C29F4 3724S (#3724) cell signaling technology) 1:1000; and vinculin (4650S (#4650) cell signaling technology) 1:1000

CRISPR Editing

Jurkat cells were edited eight days after lentiviral transduction. For each gene, three sgRNAs targeting a single exon were designed using the Synthego CRISPR Design tool. For sequences see 'sgRNA sequences' below. sgRNA were resuspended to 100 µM in IDT duplex buffer and then combined to create pools of sgRNA. Cas9 RNPs were prepared by combining 2.5 µL 36.6 µM Alt-R® S.p. Cas9 Nuclease V3 (IDT) and 2 µL 100 µM sgRNA pool in IDT duplex buffer and incubating for 10 minutes at room temperature. 1.5×10^6 cells in 20 µL SE buffer (Lonza) were

mixed with the Cas9 RNPs and then electroporated using a 4D-Nucleofector (Lonza) on pulse code CL-120. Estimation of knockout efficiency was performed by PCR amplification of the sgRNA target site using primers described in 'PCR primers for amplifying sgRNA target site' below. PCR products were Sanger sequenced and editing efficiency was assessed using the online ICE webtool [37].

HiBit and CellTiter-Glo® (CTG) Assays

Compound **23** (10 mM stock in DMSO) was serially diluted 3-fold to create an 11-point dilution spanning 169 pM–10 μM (final assay concentration). Jurkat cells transduced with HiBit-TRIM28 lentiviral vectors were added to white opaque-bottom 384-well plates (ThermoFisher) at 1×10^4 cells per well in 25 μL volume, and treated with compound for 24 h at 37 °C and 5% CO_2. The HiBit signal was measured by adding 25 μL of the lytic detection reagent from the Nano-Glo® HiBiT Extracellular Detection System (Promega), prepared as per manufacturer protocol. The CTG signal was developed using 25 μL of CTG reagent (Promega). Both assays were read using PHERAstar microplate luminescence reader (BMG Labtech). The data have been expressed as % of DMSO control and represent three independent biological replicates.

Nano-Glo® Assay

Compound **23** (10 mM stock in DMSO) was serially diluted 10-fold to create an 5-point dilution spanning 1n M–10 μM (final assay concentration) and an untreated control, with all concentrations at 0.1% DMSO. HEK293T cells transduced with HA-NanoLuc-WEIS lentiviral vectors were seeded in white, opaque, flat-bottom 96-well plate (Falcon) at 2×10^4 cells per well in 40 μL volume, and treated with compound for either 6 h or 18 h at 37 °C and 5% CO_2. The luminescence signal was measured by adding 40 μL of the Nano-Glo lytic detection reagent (Promega),

incubating at rt for 3 minutes, then reading using an EnVision multimode plate reader (PerkinElmer). The data have been expressed as % of DMSO control. Three biological repeats were carried out for this assay, with each biological replicate plate comprising three replicate concentration curves.

Compound Cellular Stability HPLC Assay

Analytical HPLC assay to identify compound **23** in mammalian cell lysate. Jurkat cells were cultured as described previously and seeded at a density of 2.5×10^5 cells per well in a 6-well plate. Cells were treated with 25 µM of compound **23** or DMSO, keeping the final concentration of DMSO consistent (0.167% DMSO *v/v*); cells were then incubated at 37 °C and 5% CO_2 for either 0.5 h, 6 h, or 24 h. Cells were then pelleted, supernatant removed, and cells were washed twice with 1 mL of PBS, centrifuging each time at 6,500 rpm on a tabletop centrifuge to avoid prematurely lysing the cells. PBS was then removed, and cells were lysed with 100 µL of HPLC-grade MeOH by vortexing for 1 minute. The lysates were then clarified at 21,000 rpm in a tabletop centrifuge for 10 minutes at rt. The supernatant was carefully removed without disturbing the cell pellet, placed in a HPLC sample vial, and samples taken for analysis. All samples were run with 0.1% formic acid. Injections of 10 µL were made. Samples were read using a Shimadzu system with Binary LC-40D Pump and UV/Vis SPD-M40 Detector using a Shim-pack GIS CN column [5 µm, 4.6 mm x 250 mm] with a constant flow rate of 1.0 mL min^{-1} and a gradient method of 10 min from H_2O:MeCN (95:5) to H_2O:MeCN (5:95) with a 5 min hold. HPLC data were processed using LabSolutions (Version 5.117). Sample components were measured using the UV absorbance at 190 nm and 254 nm. The reported analysis is the UV absorbance at 190 nm. This protocol was based on assays featured in O'Connor *et al.* and Skwarska *et al.* [38,39].

GFP Degradation Flow Cytometry Assay with Proteasome Inhibitor Carfilzomib

Jurkat cells were pre-incubated with carfilzomib (1 μM) for 45 min at 37 °C and 5% CO_2. Compound **23** (10 mM stock in DMSO) was serially diluted to create an 8-point dilution spanning 1 pM–10 μM (final assay concentration) using an Echo 555 (Labcyte). Pre-incubated Jurkat cells were then added to 96 well U-bottom assay plates (Greiner) dosed with compound **23** at 1×10^4 cells per well and incubated for 24 h at 37 °C and 5% CO_2. Flow cytometry was performed on an Attune NxT (Thermofisher). Data were analyzed using FlowJo 10.8.1 (BD).

Proteomics - cell lysis and protein digestion

The viability of Jurkat cells was tested after 16-h treatment with corresponding chemicals followed by trypan blue staining (CytoSmart cell counter, Corning) and confirmed to be >95 % viable cells in each condition. Next, ~10^7 cells per condition were collected by centrifugation (1000 rpm), washed twice with ice-cold PBS, and lysed in RIPA buffer [50 mM HEPES pH 7.4, 150 mM NaCl, 1% sodium deoxycholate, 1% NP-40, 0.1% SDS, 10 mM sodium pyrophosphate, 10 mM β-glycerophosphate, 2.5 mM $MgCl_2$, 200 μM TCEP, phosphatase and protease inhibitor cocktail (in-house)], to produce whole cell extracts. Whole-cell extracts were sonicated, and protein concentrations were determined using the Bradford assay. Protein extracts (100 μg) were subjected to disulfide bond reduction with 5 mM TCEP (10 min) and alkylation with 25 mM chloroacetamide (20 min). Methanol–chloroform precipitation was performed prior to protease digestion. In brief, four parts of neat methanol were added to each sample and vortexed, one part chloroform was added to the sample and vortexed, and finally, three parts water was added to the sample and vortexed. The sample was centrifuged at 8000 rpm for 5 min at room temperature and subsequently washed twice with 100% methanol. Samples were resuspended in 100 mM EPPS pH8.5 containing

0.1% RapiGest and digested at 37 °C for 6 h with trypsin at a 100:1 protein-to-protease ratio. The digestion efficiency of a small aliquot was tested. Samples were then subjected to TMTpro labeling. The preparation of MM1S cell extracts was conducted using the same procedure as for Jurkat cells. ESC cells were plated onto 6 cm dishes 24 hours prior to the chemical treatment. Following the chemical treatment, the cells on the dish were washed with DPBS four times, after which RIPA buffer was added directly to the dish, and the lysate was collected using a scraper. The whole cell lysates were then processed similarly to the Jurkat cell lysates.

Proteomics - Tandem mass tag labeling

Proline-based reporter isobaric Tandem Mass Tag (TMTpro) labeling of dried peptide samples resuspended in 100 mM EPPS pH 8.5, was carried out as follows. TMTpro reagent was added to samples (100 µg peptide), along with acetonitrile, to achieve a final acetonitrile concentration of approximately 30% (*v/v*). Following incubation at room temperature for 1 h, the labeling efficiency of a small aliquot was tested. The reaction was then quenched with hydroxylamine to a final concentration of 0.5% (*v/v*) for 15 min. The TMTpro-labeled samples were pooled together at a 1:1 ratio. The sample was vacuum centrifuged to near dryness and subjected to C18 solid-phase extraction (SPE).

Proteomics - off-line basic pH reversed-phase (BPRP) fractionation

The dried TMTpro-labeled sample was resuspended in 100 µL of 10 mM NH_4HCO_3 pH 8.0 and fractionated using basic pH reverse phase HPLC [40]. Briefly, samples were offline fractionated over a 90 min run, into 96 fractions by high pH reverse-phase HPLC (Agilent LC1260) through an aeris peptide xb-c18 column (Phenomenex; 250 mm × 3.6 mm) for total proteome with mobile phase A containing 5% acetonitrile and 10 mM NH_4HCO_3, and mobile phase B containing 90% acetonitrile

and 10 mM NH_4HCO_3 (both pH 8.0). The 96 resulting fractions were then pooled non-continuously into 24 fractions (as outlined in Supplemental Figure 5 of Paulo et al.) [41] used for subsequent mass spectrometry analysis. Fractions were vacuum centrifuged to near dryness. Each consolidated fraction was desalted via StageTip, dried again *via* vacuum centrifugation, and reconstituted in 5% acetonitrile, 1% formic acid for LC-MS/MS processing.

Proteomics – total proteomics analysis using TMTpro

Mass spectrometry data were collected using an Orbitrap Eclipse Tribrid mass spectrometer (Thermo Fisher Scientific, San Jose, CA) coupled to an UltiMate 3000 RSLCnano system liquid chromatography (LC) pump (Thermo Fisher Scientific). Peptides were separated on a 100 μm inner diameter microcapillary column packed in-house with ~40 cm of HALO Peptide ES-C18 resin (2.7 μm, 160 Å, Advanced Materials Technology, Wilmington, DE) with a gradient consisting of 5%–24% (0-85 min), 24-35% (85-110min) (ACN, 0.1% FA) over a total 125 min run at ~500 nL/min. For analysis, we loaded 1/10 of each fraction onto the column. Each analysis used the Multi-Notch MS^3-based TMT method, [42] to reduce ion interference compared to MS^2 quantification, [43] combined with the FAIMS Pro Interface (using previously optimized 3 CV parameters for TMT multiplexed samples) [44] and combined with Real-Time Search analysis software [45,46]. The scan sequence began with an MS^1 spectrum (Orbitrap analysis; resolution 120,000 at 200 Th; mass range 400−1500 *m/z*; automatic gain control (AGC) target 4×10^5; maximum injection time 50 ms). Precursors for MS^2 analysis were selected using a cycle type of 1.25 sec/CV method (FAIMS CV=-40/-60/-80). MS^2 analysis consisted of collision-induced dissociation (quadrupole ion trap analysis; Rapid scan rate; AGC 1.0×10^4; isolation window 0.5 Th; normalized collision energy (NCE) 35; maximum injection time 35 ms). Monoisotopic peak assignment was used, and previously interrogated precursors were excluded using a dynamic

window (120 s ±10 ppm). Following the acquisition of each MS^2 spectrum, a synchronous-precursor-selection (SPS) API-MS3 scan was collected on the top 10 most intense ions b or y-ions matched by the online search algorithm in the associated MS^2 spectrum [45,46]. MS^3 precursors were fragmented by high energy collision-induced dissociation (HCD) and analyzed using the Orbitrap (NCE 45; AGC 2.5×10^5; maximum injection time 200 ms, resolution was 50,000 at 200 Th). The closeout was set at two peptides per protein per fraction so that MS^3s were no longer collected for proteins having two peptide-spectrum matches (PSMs) that passed the quality filters [46].

Proteomics – data analysis

Mass spectra were converted to mzXML [47] and processed using the open-source Comet search engine (2020.01 rev. 4) software pipeline (Eng et al., 2013) with the Human Reference Proteome (2020-03 - SwissProt (w isoforms) entries only) UniProt database with contaminants and reverse decoy sequences appended. For analysis, searches were performed with a 50 ppm precursor ion tolerance, and product ion parameters for ion trap MS/MS were used. The theoretical fragment ions were set to 1 with a tolerance of 1.0005 and a 0.4 offset (mono masses). TMTpro tags on lysine residues and peptide N termini (+304.207 Da) and carbamidomethylation of cysteine residues (+57.021 Da) were set as static modifications, while oxidation of methionine residues (+15.995 Da) was set as a variable modification. Search results were first filtered to a 1% peptide FDR using linear discriminant analysis employing a target-decoy strategy and further filtered to obtain a protein level FDR of 1% [48–50]. Moreover, protein assembly was guided by principles of parsimony to produce the smallest set of proteins necessary to account for all observed peptides. For TMTpro-based reporter ion quantitation, we extracted the summed signal-to-noise (S:N) ratio for each TMTpro channel and found the closest matching centroid to the expected mass of the TMT reporter ion (integration tolerance of 0.003 Da). Reporter ion intensities were adjusted to

correct for the isotopic impurities of the different TMTpro reagents according to manufacturer specifications. Proteins were quantified by summing reporter ion signal-to-noise measurements across all matching PSMs, yielding a ''summed signal-to-noise'' measurement. PSMs with poor quality, MS^3 spectra with 8 or more TMTpro reporter ion channels missing, or isolation specificity less than 0.5, or with TMT reporter summed signal-to-noise ratio that was less than 160 or had no MS^3 spectra were excluded from quantification.

Protein quantification values were exported for further analysis in Microsoft Excel, GraphPad Prism, and Perseus [51]. Each reporter ion channel was summed across all quantified proteins and normalized, assuming equal protein loading of all samples. A two-sided Welch's t-test was conducted on the specified sample comparisons with FDR correction applied for multiple comparisons (Figures 7, S18 and S19).

Supplemental Data Tables list all quantified proteins and the associated TMTpro reporter ratio to control channels used for quantitative analysis.

Plasmid Information

Plasmid: pLVX-Sprout

Backbone: pLVX-EF1a-IRES-mCherry (Takara)

Insert sequence cloned into MCS:

cagtgttctagaggaatagccaccatgtccggattcaatgtcttaatggttcataagcgaagccatactggtgaagagacggacgtctcagaa
aaaccttttaagtgtcacctctgcaactatgcatgccaaagaagagatgcgctcacgcgtgctgaagctgctgcaaaggaagctgcagctaa
ggaggctgcagctaaggctgtgagcaagggcgaggagctgttcaccggggtggtgcccatcctggtcgagctggacggcgacgtaaac
ggccacaagttcagcgtgtccggcgagggcgagggcgatgccacctatggcaaactgaccctgaaattcatctgcaccaccggcaaact
gcccgtgccctggcccaccctcgtgaccaccctgacctatggcgtgcagtgcttcagccgctatcccgaccacatgaaacagcacgacttc
ttcaagtccgccatgcccgaaggctacgtccaggagcgcaccatcttcttcaaggacgacggcaactacaagacccgcgccgaggtgaa
gttcgagggcgacaccctggtgaaccgcatcgagctgaagggcatcgacttcaaggaggacggcaacatcctggggcacaagctggag
tacaactacaacagccacaacgtctatatcatggccgacaagcagaagaacggcatcaaggtgaacttcaagatccgccacaacatcgag
gacggcagcgtgcagctcgccgaccactatcagcagaacacccccatcggcgacggccccgtgctgctgcccgacaaccactatctgag
cacccagtccgccctgagcaaagaccccaacgagaaacgcgatcacatggtcctgctggagttcgtgaccgccgccgggatcactctcg
gcatggatgaactgtataaataaggatccccacat

Plasmid map:

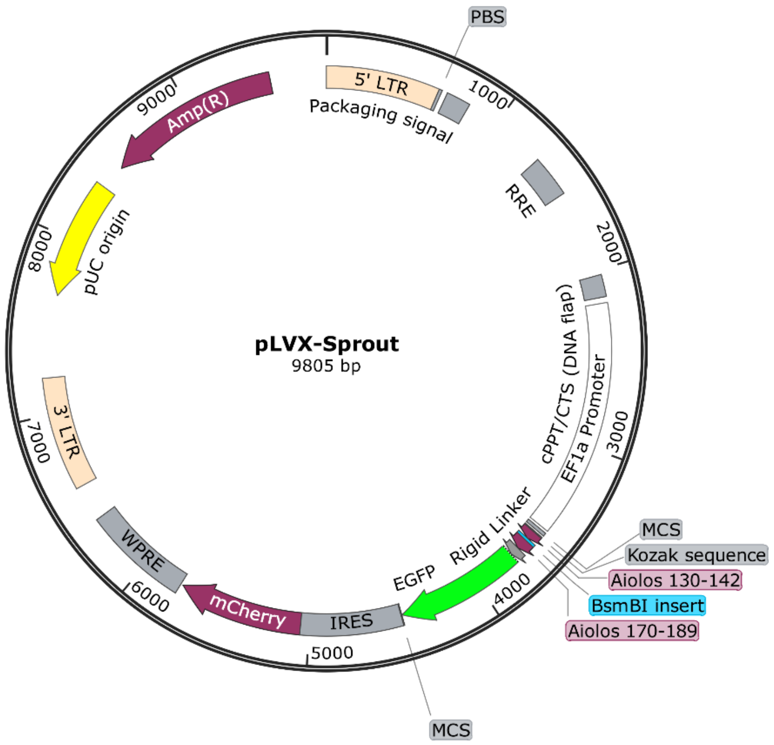

Plasmid: pLVX-Sprout-IKZF3 130-189aa (WT)

Backbone: pLVX-Sprout

Insert sequence cloned into pLVX-Sprout:

cagtgtgggctaccgtctcagtgaacgcccattccagtgtaatcagtgtggggcatcttttactcagaaaggtaacctcctccgccacattaaa

ctgcacacaggggaaacgagacggtagccgccacat

Plasmid map:

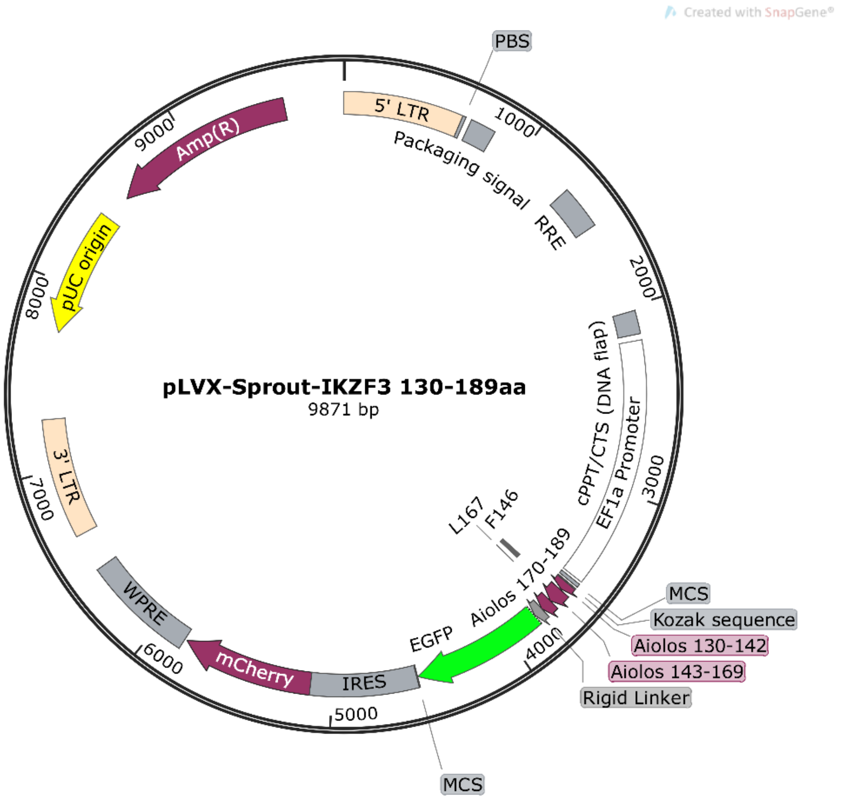

Plasmid: pLVX-Sprout-IKZF3 130-189aa Q147A (ANQS)

Backbone: pLVX-Sprout

Insert sequence cloned into pLVX-Sprout:

cagtgtgggctaccgtctcagtgaacgcccattcgcctgtaatcagtgtggggcatcttttactcagaaaggtaacctcctccgccacattaaa ctgcacacaggggaaacgagacggtagccgccacat

Plasmid: pLVX-Sprout-IKZF3 130-189aa Q147A N149P Q150I (APIS)

Backbone: pLVX-Sprout

Insert sequence cloned into pLVX-Sprout:

cagtgtgggctaccgtctcagtgaacgcccattcgcctgtccaatctgtggggcatcttttactcagaaaggtaacctcctccgccacattaaa ctgcacacaggggaaacgagacggtagccgccacat

Plasmid: pLVX-Sprout-IKZF3 130-189aa Q147W (WNQS)

Backbone: pLVX-Sprout

Insert sequence cloned into pLVX-Sprout:

cagtgtgggctaccgtctcagtgaacgcccattctggtgtaatcagtgtggggcatcttttactcagaaaggtaacctcctccgccacattaaa ctgcacacaggggaaacgagacggtagccgccacat

Plasmid: pLVX-Sprout-IKZF3 130-189aa Q147H N149E Q150I S154P (HEIP)

Backbone: pLVX-Sprout

Insert sequence cloned into pLVX-Sprout:

cagtgtgggctaccgtctcagtgaacgcccattccattgtgagatctgtggggcaccatttactcagaaaggtaacctcctccgccacatt aaactgcacacaggggaaacgagacggtagccgccacat

Plasmid: pLVX-Sprout-IKZF3 130-189aa Q147F N149E Q150V S154P (FEVP)

Backbone: pLVX-Sprout

Insert sequence cloned into pLVX-Sprout:

cagtgtgggctaccgtctcagtgaacgcccattcttctgtgaggtgtgtggggcacccttactcagaaaggtaacctcctccgccacattaa actgcacacaggggaaacgagacggtagccgccacat

Plasmid: pLVX-Sprout-IKZF3 130-189aa Q147W N149E Q150I S154K (WEIK)

Backbone: pLVX-Sprout

Insert sequence cloned into pLVX-Sprout:

cagtgtgggctaccgtctcagtgaacgcccattctggtgtgaaatctgtggggcaaagtttactcagaaaggtaacctcctccgccacattaa actgcacacaggggaaacgagacggtagccgccacat

Plasmid: pLVX-Sprout-IKZF3 130-189aa Q147F N149D Q150I S154P (FDIP)

Backbone: pLVX-Sprout

Insert sequence cloned into pLVX-Sprout:

cagtgtgggctaccgtctcagtgaacgcccattcttctgtgacatatgtggggcacccttactcagaaaggtaacctcctccgccacatta aactgcacacaggggaaacgagacggtagccgccacat

Plasmid: pLVX-Sprout-IKZF3 130-189aa Q147A N149E Q150V S154K (AEVK)

Backbone: pLVX-Sprout

Insert sequence cloned into pLVX-Sprout:

cagtgtgggctaccgtctcagtgaacgcccattcgcttgtgaagtgtgtggggcaaaatttactcagaaaggtaacctcctccgccacatt aaactgcacacaggggaaacgagacggtagccgccacat

Plasmid: pLVX-Sprout-IKZF3 130-189aa Q147W N149E Q150I (WEIS)

Backbone: pLVX-Sprout

Insert sequence cloned into pLVX-Sprout:

cagtgtgggctaccgtctcagtgaacgcccattctggtgtgagatttgtggggcatcttttactcagaaaggtaacctcctccgccacatta aactgcacacaggggaaacgagacggtagccgccacat

Plasmid: pLVX-Sprout-IKZF3 130-189aa Q147W Q150I S154Q (WNIQ)

Backbone: pLVX-Sprout

Insert sequence cloned into pLVX-Sprout:

cagtgtgggctaccgtctcagtgaacgcccattctggtgtaacatctgtggggcacagtttactcagaaaggtaacctcctccgccacatt aaactgcacacaggggaaacgagacggtagccgccacat

Plasmid: pLVX-Sprout-IKZF3 130-189aa Q147W N149D Q150I S154A (WDIA)

Backbone: pLVX-Sprout

Insert sequence cloned into pLVX-Sprout:

cagtgtgggctaccgtctcagtgaacgcccattctggtgtgatatctgtggggcagcctttactcagaaaggtaacctcctccgccacattaa
actgcacacaggggaaacgagacggtagccgccacat

Plasmid: pLVX-Sprout-IKZF3 130-189aa N149P Q150I (QPIS)

Backbone: pLVX-Sprout

Insert sequence cloned into pLVX-Sprout:

cagtgtgggctaccgtctcagtgaacgcccattccagtgtcctatctgtggggcaagctttactcagaaaggtaacctcctccgccacattaa
actgcacacaggggaaacgagacggtagccgccacat

Plasmid: pLVX-HiBit-TRIM28

Backbone: pLVX-EF1a-IRES-mCherry (Takara)

Insert sequence cloned into MCS:

cagtgttctagaggaatagccaccatggtgagcggctggcggctgttcaagaagattagcggcggaggcggaggtggcgcagcaagtg
cagctgctgcaagcgccgctgctgcttccgcagctagtggttcaccgggtccgggagagggatctgctggaggtgagaagcgcagcaca
gccccaagtgctgctgcgtccgcttcagcttcagctgcagcaagtagcccagcggggggtggcgcagaggctctcgaactgttggaacat
tgtggcgtctgtagagagaggcttcgcccggagagggagcctaggctccttccgtgtcttcattctgcctgcagcgcatgtttgggcccagc

ggcaccggcggctgcaaattcatccggggatgggggcgcagcaggtgacggtaccgtggtagactgtccagtatgcaagcagcaatgttt
ttcaaaagacatagttgagaactattttatgagagatagcggtagcaaggctgcaacagacgcgcaagatgcgaatcaatgctgtacctcat
gtgaagataatgctccggccacgagttactgtgtggagtgttcagaaccgctttgtgaaacttgcgtcgaggcgcaccaaagggtcaaatac
actaaagatcataccgtaaggtcaacaggaccagcgaaatcccgcgacggagaacgaacggtctactgcaacgtccataagcatgagcc
gttggtccttttctgcgagtcttgcgatacgctcacctgtagggattgtcaactgaacgcgcataaggatcatcaatatcagtttctggaagatg
ctgtccggaatcagcgaaaacttctcgcaagcctcgtgaaaaggctcggcgacaaacatgcgactctccagaagtcaaccaaggaagtcc
ggagcagcatacgacaagtgtctgatgttcaaaagcgagtgcaagtagatgtcaagatggccatactccaaataatgaaggagctcaataa
acggggtcgagtccttgtcaacgatgcacagaaggtcacggaggggcaacaggaaaggctggaacgacaacactggactatgaccaag
attcaaaagcaccaagagcacatacttcgctttgcatcatgggcgttggagtctgataacaatacggcactgctcctgtctaagaaattgatct
attttcagctgcacagggctctgaaaatgatagtcgatccagttgaaccccacggggagatgaaatttcagtgggacctgaatgcatggaca
aagtccgctgaagcattcggaaagattgtagctgaaaggcccgggactaactcaacaggacccgctcctatggctcctccacgcgcacct
ggtcctctgtcaaaacaggggagtggctcatcacaacccatggaagtgcaagagggttatggttttggaagcggagatgatccctattctag
cgcggaacctcacgtatcaggggtcaaacgatcccgctcaggtgagggcgaggtcagtgggctgatgcgaaaggtaccccgcgtcagtc
tggagcggttggatcttgatcttaccgcggatagtcaacctcccgttttcaaagtttttccgggttcaactacggaagattataacctcatagtaa
tcgagagaggagctgcagccgcggctacaggccaacctgggacagcaccagcaggtacacccggggcccctccacttgcggggatgg
ccattgttaaagaagaagagacggaagcagcgattggggctcctccaaccgccaccgaaggacccgaaacaaaaccagttctcatggca
ctggctgaaggccctggggctgaagggccgagattggccagtccgtcagggagcacttcatctgggttggaagtcgtggcccctgaggg
cacaagtgcacccgggggggggcccaggcactctcgatgactctgctacgatctgtcgggtatgtcaaaaaccaggcgaccttgttatgtgc
aatcagtgcgaattctgcttccacctggactgtcaccttccggccctgcaggacgttccaggtgaggaatggagctgtagcctgtgtcacgta
ctcccggatctgaaggaggaagatggctcactgtcactggacggtgcagattccaccggagttgtggccaagttgtccccagcaaatcaga
gaaagtgcgaaagggtcctgcttgcgttgttctgccacgaaccgtgtcggcctctgcaccagttggcgacggactccacgttctctctggatc
agcccggtgggaccttggatcttactctcatacgggctcgactccaagagaagctttcacctccttatagttccccccaggagttcgctcaag
acgtaggcaggatgttcaagcaatttaataagttgactgaggacaaagcggatgttcaaagcattatcggattgcaaagattctttgagacac

gaatgaatgaagcattcggggacaccaaatttagcgcggtacttgtagagccccctcctatgtccttgccaggtgcaggtttgtcctcccagg

aattgtctgggggaccgggtgatggaccctgaggatccccacat

Plasmid map:

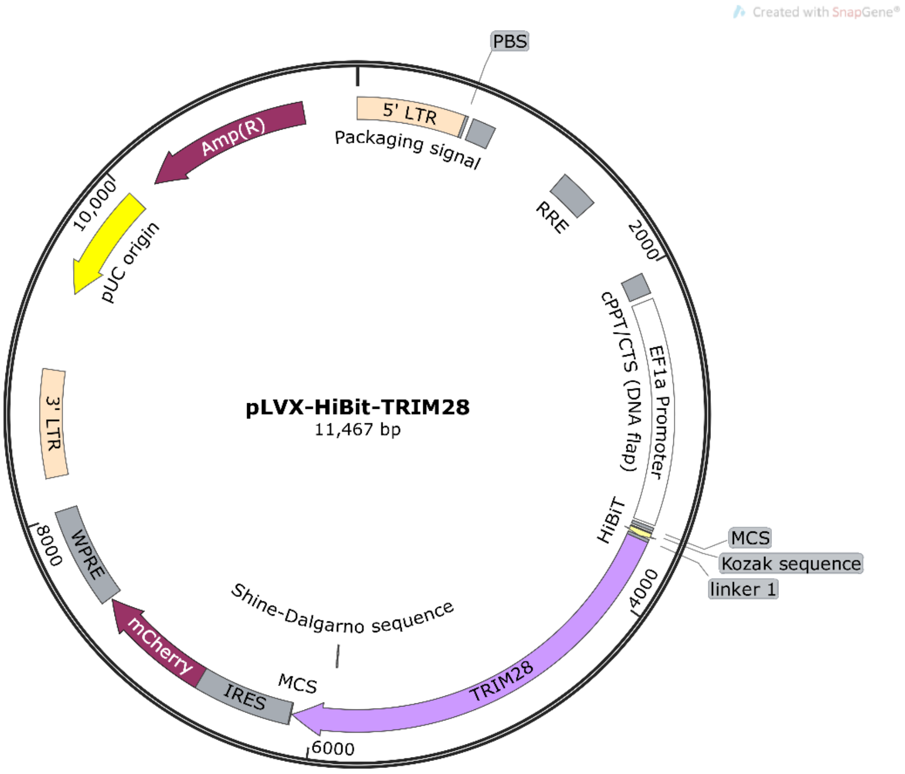

Plasmid: pLVX-HiBit-TRIM28 N-degron

Backbone: pLVX-EF1a-IRES-mCherry (Takara)

Insert sequence cloned into MCS:

cagtgttctagaggaatagccaccatggtgagcggctggcggctgttcaagaagattagcggcggaggcggaggtggctccggattcaat

gtcttaatggttcataagcgaagccatactggtgaacgcccattctggtgtgagatttgtggggcatcttttactcagaaaggtaacctcctccg

ccacattaaactgcacacaggggaaaaaccttttaagtgtcacctctgcaactatgcatgccaaagaagagatgcgctcacgcgtgctgaag

ccgctgcaaaggaagccgcagctaaggaggcagctgccaaggccgcagcaagtgcagctgctgcaagcgccgctgctgcttccgcag

ctagtggttcaccgggtccgggagagggatctgctggaggtgagaagcgcagcacagccccaagtgctgctgcgtccgcttcagcttcag

ctgcagcaagtagcccagcggggggtggcgcagaggctctcgaactgttggaacattgtggcgtctgtagagagaggcttcgcccggag

agggagcctaggctccttccgtgtcttcattctgcctgcagcgcatgtttgggcccagcggcaccggcggctgcaaattcatccggggatg

ggggcgcagcaggtgacggtaccgtggtagactgtccagtatgcaagcagcaatgtttttcaaaagacatagttgagaactattttatgaga

gatagcggtagcaaggctgcaacagacgcgcaagatgcgaatcaatgctgtacctcatgtgaagataatgctccggccacgagttactgtg

tggagtgttcagaaccgctttgtgaaacttgcgtcgaggcgcaccaaagggtcaaatacactaaagatcataccgtaaggtcaacaggacc

agcgaaatcccgcgacggagaacgaacggtctactgcaacgtccataagcatgagccgttggtccttttctgcgagtcttgcgatacgctca

cctgtagggattgtcaactgaacgcgcataaggatcatcaatatcagtttctggaagatgctgtccggaatcagcgaaaacttctcgcaagcc

tcgtgaaaaggctcggcgacaaacatgcgactctccagaagtcaaccaaggaagtccggagcagcatacgacaagtgtctgatgttcaaa

agcgagtgcaagtagatgtcaagatggccatactccaaataatgaaggagctcaataaacggggtcgagtccttgtcaacgatgcacagaa

ggtcacggaggggcaacaggaaaggctggaacgacaacactggactatgaccaagattcaaaagcaccaagagcacatacttcgctttg

catcatgggcgttggagtctgataacaatacggcactgctcctgtctaagaaattgatctattttcagctgcacagggctctgaaaatgatagtc

gatccagttgaaccccacggggagatgaaatttcagtgggacctgaatgcatggacaaagtccgctgaagcattcggaaagattgtagctg

aaaggcccgggactaactcaacaggacccgctcctatggctcctccacgcgcacctggtcctctgtcaaaacaggggagtggctcatcac

aacccatggaagtgcaagagggttatggttttggaagcggagatgatccctattctagcgcggaacctcacgtatcaggggtcaaacgatc

ccgctcaggtgagggcgaggtcagtgggctgatgcgaaaggtaccccgcgtcagtctggagcggttggatcttgatcttaccgcggatagt

caacctcccgttttcaaagtttttccgggttcaactacggaagattataacctcatagtaatcgagagaggagctgcagccgcggctacaggc

caacctgggacagcaccagcaggtacacccggggcccctccacttgcggggatggccattgttaaagaagaagagacggaagcagcga

ttggggctcctccaaccgccaccgaaggacccgaaacaaaaccagttctcatggcactggctgaaggccctggggctgaagggccgag

attggccagtccgtcagggagcacttcatctgggttggaagtcgtggcccctgagggcacaagtgcacccgggggggcccaggcactc

tcgatgactctgctacgatctgtcgggtatgtcaaaaaccaggcgaccttgttatgtgcaatcagtgcgaattctgcttccacctggactgtcac

cttccggccctgcaggacgttccaggtgaggaatggagctgtagcctgtgtcacgtactcccggatctgaaggaggaagatggctcactgt

cactggacggtgcagattccaccggagttgtggccaagttgtccccagcaaatcagagaaagtgcgaaagggtcctgcttgcgttgttctgc

cacgaaccgtgtcggcctctgcaccagttggcgacggactccacgttctctctggatcagcccggtgggaccttggatcttactctcatacg

ggctcgactccaagagaagctttcacctccttatagttccccccaggagttcgctcaagacgtaggcaggatgttcaagcaatttaataagttg

actgaggacaaagcggatgttcaaagcattatcggattgcaaagattctttgagacacgaatgaatgaagcattcggggacaccaaatttag

cgcggtacttgtagagccccctcctatgtccttgccaggtgcaggtttgtcctcccaggaattgtctgggggaccgggtgatggaccctgag

gatccccacat

Plasmid map:

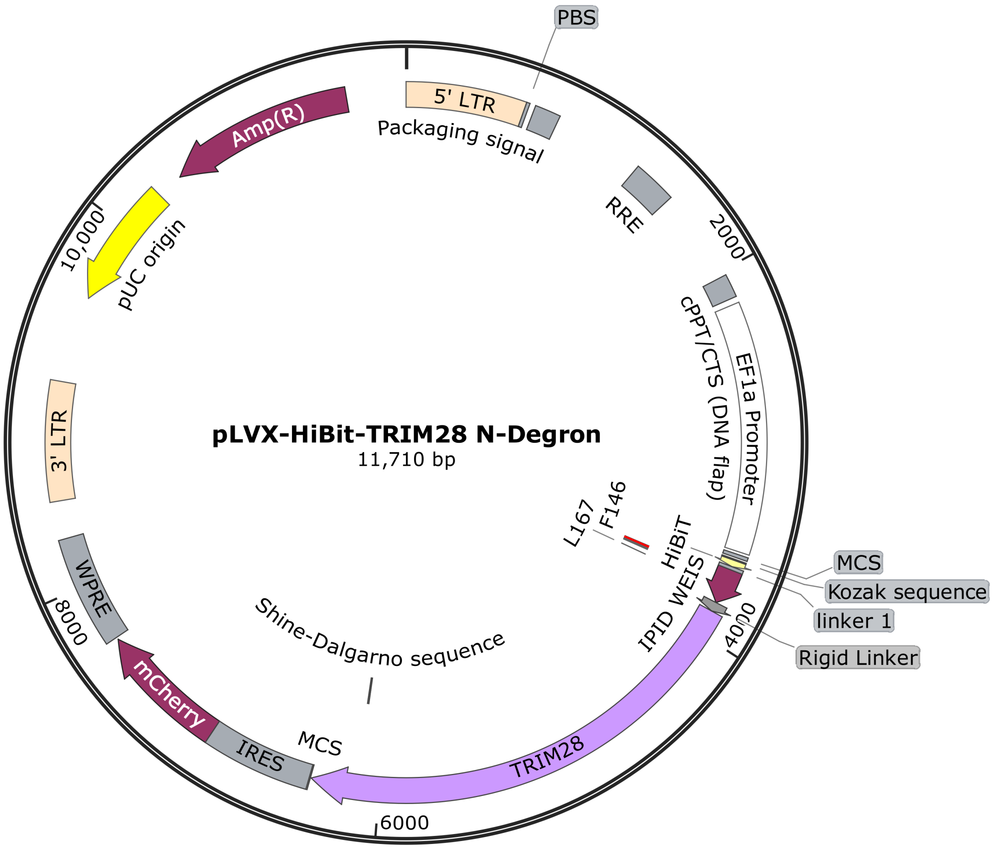

Plasmid: pLVX-HiBit-TRIM28 C-degron

Backbone: pLVX-EF1a-IRES-mCherry (Takara)

Insert sequence cloned into MCS:

cagtgttctagaggaatagccaccatggtgagcggctggcggctgttcaagaagattagcggcggaggcggaggtggagcagcaagtg
cagctgctgcaagcgccgctgctgcttccgcagctagtggttcaccgggtccgggagagggatctgctggaggtgagaagcgcagcaca
gccccaagtgctgctgcgtccgcttcagcttcagctgcagcaagtagcccagcgggggggtggcgcagaggctctcgaactgttggaacat
tgtggcgtctgtagagagaggcttcgcccggagagggagcctaggctccttccgtgtcttcattctgcctgcagcgcatgtttgggcccagc
ggcaccggcggctgcaaattcatccggggatgggggcgcagcaggtgacggtaccgtggtagactgtccagtatgcaagcagcaatgttt
ttcaaaagacatagttgagaactattttatgagagatagcggtagcaaggctgcaacagacgcgcaagatgcgaatcaatgctgtacctcat
gtgaagataatgctccggccacgagttactgtgtggagtgttcagaaccgctttgtgaaacttgcgtcgaggcgcaccaaagggtcaaatac
actaaagatcataccgtaaggtcaacaggaccagcgaaatcccgcgacggagaacgaacggtctactgcaacgtccataagcatgagcc
gttggtccttttctgcgagtcttgcgatacgctcacctgtagggattgtcaactgaacgcgcataaggatcatcaatatcagtttctggaagatg
ctgtccggaatcagcgaaaacttctcgcaagcctcgtgaaaaggctcggcgacaaacatgcgactctccagaagtcaaccaaggaagtcc
ggagcagcatacgacaagtgtctgatgttcaaaagcgagtgcaagtagatgtcaagatggccatactccaaataatgaaggagctcaataa
acggggtcgagtccttgtcaacgatgcacagaaggtcacggaggggcaacaggaaaggctggaacgacaacactggactatgaccaag
attcaaaagcaccaagagcacatacttcgctttgcatcatgggcgttggagtctgataacaatacggcactgctcctgtctaagaaattgatct
attttcagctgcacagggctctgaaaatgatagtcgatccagttgaaccccacggggagatgaaatttcagtgggacctgaatgcatggaca
aagtccgctgaagcattcggaaagattgtagctgaaaggcccgggactaactcaacaggacccgctcctatggctcctccacgcgcacct
ggtcctctgtcaaaacaggggagtggctcatcacaacccatggaagtgcaagagggttatggttttggaagcggagatgatccctattctag
cgcggaacctcacgtatcaggggtcaaacgatcccgctcaggtgagggcgaggtcagtgggctgatgcgaaaggtaccccgcgtcagtc
tggagcggttggatcttgatcttaccgcggatagtcaacctcccgttttcaaagtttttccgggttcaactacggaagattataacctcatagtaa
tcgagagaggagctgcagccgcggctacaggccaacctgggacagcaccagcaggtacacccggggcccctccacttgcggggatgg
ccattgttaaagaagaagagacggaagcagcgattggggctcctccaaccgccaccgaaggacccgaaacaaaaccagttctcatggca

ctggctgaaggccctggggctgaagggccgagattggccagtccgtcagggagcacttcatctgggttggaagtcgtggcccctgaggg

cacaagtgcacccgggggggggcccaggcactctcgatgactctgctacgatctgtcgggtatgtcaaaaaccaggcgaccttgttatgtgc

aatcagtgcgaattctgcttccacctggactgtcaccttccggccctgcaggacgttccaggtgaggaatggagctgtagcctgtgtcacgta

ctcccggatctgaaggaggaagatggctcactgtcactggacggtgcagattccaccggagttgtggccaagttgtccccagcaaatcaga

gaaagtgcgaaagggtcctgcttgcgttgttctgccacgaaccgtgtcggcctctgcaccagttggcgacggactccacgttctctctggatc

agcccggtgggaccttggatcttactctcatacgggctcgactccaagagaagctttcacctccttatagttccccccaggagttcgctcaag

acgtaggcaggatgttcaagcaatttaataagttgactgaggacaaagcggatgttcaaagcattatcggattgcaaagattctttgagacac

gaatgaatgaagcattcggggacaccaaatttagcgcggtacttgtagagccccctcctatgtccttgccaggtgcaggtttgtcctcccagg

aattgtctgggggaccgggtgatggacccgctgaagccgctgcaaaggaagccgcagctaaggaggcagctgccaaggcctccggatt

caatgtcttaatggttcataagcgaagccatactggtgaacgcccattctggtgtgagatttgtggggcatcttttactcagaaaggtaacctcc

tccgccacattaaactgcacacaggggaaaaaccttttaagtgtcacctctgcaactatgcatgccaaagaagagatgcgctcacgcgttga

ggatccccacat

Plasmid map:

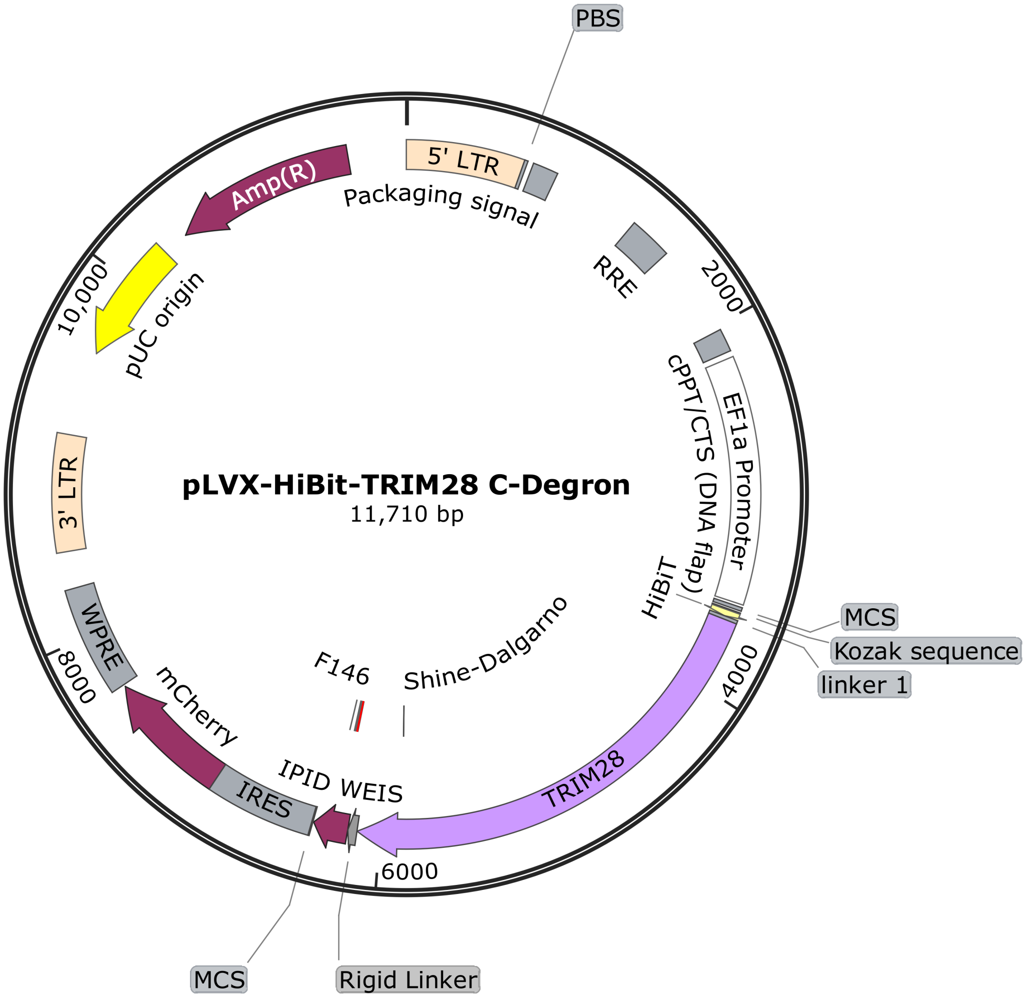

pLVX-HiBit-TRIM28 C-Degron
11,710 bp
PBS
5' LTR
Packaging signal
Amp(R)
RRE
2000
10,000
pUC origin
cPPT/CTS (DNA flap)
EF1a Promoter
3' LTR
HiBiT
MCS
Kozak sequence
linker 1
4000
Shine-Dalgarno
F146
WPRE
8000
mCherry
TRIM28
IPID WEIS
IRES
6000
MCS
Rigid Linker

Plasmid: Artichoke 130-189 (WT)

Backbone: Artichoke (Addgene #73320)

Insert cloned into Artichoke:

ggctaccgtctcaccatgtccggattcaatgtcttaatggttcataagcgaagccatactggtgaacgcccattccagtgtaatcagtgtgg ggcatcttttactcagaaaggtaacctcctccgccacattaaactgcacacaggggaaaaaccttttaagtgtcacctctgcaactatgcatgc caaagaagagatgcgctcacgcgtgctgagagacgagtagc

Plasmid: Artichoke 130-189 (WEIS)

Backbone: Artichoke (Addgene #73320)

Insert cloned into Artichoke:

ggctaccgtctcaccatgtccggattcaatgtcttaatggttcataagcgaagccatactggtgaacgcccattctggtgtgagatttgtgg ggcatcttttactcagaaaggtaacctcctccgccacattaaactgcacacaggggaaaaaccttttaagtgtcacctctgcaactatgcatgc caaagaagagatgcgctcacgcgtgctgagagacgagtagc

Plasmid: Cil2_no_eGFP Backbone:

Cilantro2 (Addgene #74450)

Primers for linear backbone fragment A:

gtaaagcactaaatcggaaccctaaagggagccc

atgcatgagtaactgaggatccgcccctc

Backbone fragment A sequence:

atgcatgagtaactgaggatccgcccctctccctcccccccccctaacgttactggccgaagccgcttggaataaggccggtgtgcgttt
gtctatatgttattttccaccatattgccgtcttttggcaatgtgagggcccggaaacctggccctgtcttcttgacgagcattcctaggggtcttt
cccctctcgccaaaggaatgcaaggtctgttgaatgtcgtgaaggaagcagttcctctggaagcttcttgaagacaaacaacgtctgtagcg
accctttgcaggcagcggaaccccccacctggcgacaggtgcctctgcggccaaaagccacgtgtataagatacacctgcaaaggcggc
acaaccccagtgccacgttgtgagttggatagttgtggaaagagtcaaatggctctcctcaagcgtattcaacaaggggctgaaggatgccc
agaaggtaccccattgtatgggatctgatctggggcctcggtacacatgctttacatgtgtttagtcgaggttaaaaaaacgtctaggccccccg
aaccacggggacgtggttttcctttgaaaaacacgatgataatatggccacaaccctggaattcgccaccatggtgagcaagggcgagg
aggataacatggccatcatcaaggagttcatgcgcttcaaggtgcacatggagggctccgtgaacggccacgagttcgagatcgagggcg
agggcgagggccgcccctacgagggcacccagaccgccaagctgaaggtgaccaagggtggccccctgcccttcgcctgggacatcc
tgtcccctcagttcatgtacggctccaaggcctacgtgaagcaccccgccgacatccccgactacttgaagctgtccttccccgagggcttc
aagtgggagcgcgtgatgaacttcgaggacggcggcgtggtgaccgtgacccaggactcctccctgcaggacggcgagttcatctacaa
ggtgaagctgcgcggcaccaacttcccctccgacggccccgtaatgcagaagaagaccatgggctgggaggcctcctccgagcggatgt
accccgaggacggcgccctgaagggcgagatcaagcagaggctgaagctgaaggacggcggccactacgacgctgaggtcaagacc
acctacaaggccaagaagcccgtgcagctgcccggcgcctacaacgtcaacatcaagttggacatcacctcccacaacgaggactacac
catcgtggaacagtacgaacgcgccgagggccgccactccaccggcggcatggacgagctgtacaagtaaactagtaagcttggcgtaa
ctagatcttgagacaaatggcagtattcatccacaattttaaaagaaaaggggggattggggggtacagtgcaggggaaagaatagtagac
ataatagcaacagacatacaaactaaagaattacaaaaacaaattacaaaaattcaaaattttcgggtttattacagggacagcagagatcca
ctttgggctcgagggggcccgggtgcaaagatggataaagttttaaacagagaggaatctttgcagctaatggaccttctaggtcttgaaag
gagtgggaattggctccggtgcccgtcagtgggcagagcgcacatcgcccacagtccccgagaagttggggggaggggtcggcaattg
atccggtgcctagagaaggtggcgcggggtaaactgggaaagtgatgtcgtgtactggctccgcctttttcccgagggtgggggagaacc
gtatataagtgcagtagtcgccgtgaacgttctttttcgcaacgggtttgccgccagaacacaggtaagtgccgtgtgtggttcccgcgggcc

tggcctctttacgggttatggcccttgcgtgccttgaattacttccacctggctgcagtacgtgattcttgatcccgagcttcgggttggaagtg
ggtgggagagttcgaggccttgcgcttaaggagccccttcgcctcgtgcttgagttgaggcctggcctgggcgctggggccgccgcgtgc
gaatctggtggcaccttcgcgcctgtctcgctgctttcgataagtctctagccatttaaaatttttgatgacctgctgcgacgctttttttctggcaa
gatagtcttgtaaatgcgggccaagatctgcacactggtatttcggtttttggggccgcgggcggcgacggggcccgtgcgtcccagcgca
catgttcggcgaggcggggcctgcgagcgcggccaccgagaatcggacgggggtagtctcaagctggccggcctgctctggtgcctgg
cctcgcgccgccgtgtatcgccccgccctgggcggcaaggctggcccggtcggcaccagttgcgtgagcggaaagatggccgcttccc
ggccctgctgcagggagctcaaaatggaggacgcggcgctcgggagagcgggcgggtgagtcacccacacaaaggaaaagggccttt
ccgtcctcagccgtcgcttcatgtgactccacggagtaccgggcgccgtccaggcacctcgattagttctcgagcttttggagtacgtcgtctt
taggttgggggggaggggttttatgcgatggagtttccccacactgagtgggtggagactgaagttaggccagcttggcacttgatgtaattct
ccttggaatttgccctttttgagtttggatcttggttcattctcaagcctcagacagtggttcaaagtttttttcttccatttcaggtgtcgtgacgtac
ggccaccatgaccgagtacaagcccacggtgcgcctcgccacccgcgacgacgtccccagggccgtacgcaccctcgccgccgcgttc
gccgactaccccgccacgcgccacaccgtcgatccggaccgccacatcgagcgggtcaccgagctgcaagaactcttcctcacgcgcgt
cgggctcgacatcggcaaggtgtgggtcgcggacgacggcgccgccgtggcggtctggaccacgccggagagcgtcgaagcggggg
cggtgttcgccgagatcggcccgcgcatggccgagttgagcggttcccggctggccgcgcagcaacagatggaaggcctcctggcgcc
gcaccggcccaaggagcccgcgtggttcctggccaccgtcggagtctcgcccgaccaccagggcaagggtctgggcagcgccgtcgt
gctccccggagtggaggcggccgagcgcgccggggtgcccgccttcctggagacctccgcgccccgcaacctccccttctacgagcgg
ctcggcttcaccgtcaccgccgacgtcgaggtgcccgaaggaccgcgcacctggtgcatgacccgcaagcccggtgcctgaacgcgtta
agtcgacaatcaacctctggattacaaaatttgtgaaagattgactggtattcttaactatgttgctccttttacgctatgtggatacgctgctttaat
gcctttgtatcatgctattgcttcccgtatggctttcattttctcctccttgtataaatcctggttgctgtctctttatgaggagttgtggcccgttgtca
ggcaacgtggcgtggtgtgcactgtgtttgctgacgcaacccccactggttggggcattgccaccacctgtcagctcctttccgggactttcg
ctttccccctccctattgccacggcggaactcatcgccgcctgccttgcccgctgctggacaggggctcggctgttgggcactgacaattcc
gtggtgttgtcggggaaatcatcgtcctttccttggctgctcgcctgtgttgccacctggattctgcgcgggacgtccttctgctacgtcccttc
ggccctcaatccagcggaccttccttcccgcggcctgctgccggctctgcggcctcttccgcgtcttcgccttcgccctcagacgagtcgga

tctccctttgggccgcctccccgcgtcgactttaagaccaatgacttacaaggcagctgtagatcttagccactttttaaaagaaaagggggg
actggaagggctaattcactcccaacgaagacaagatctgctttttgcttgtactgggtctctctggttagaccagatctgagcctgggagctc
tctggctaactagggaacccactgcttaagcctcaataaagcttgccttgagtgcttcaagtagtgtgtgcccgtctgttgtgtgactctggtaa
ctagagatccctcagacccttttagtcagtgtggaaaatctctagcagtacgtatagtagttcatgtcatcttattattcagtatttataacttgcaa
agaaatgaatatcagagagtgagaggaacttgtttattgcagcttataatggttacaaataaagcaatagcatcacaaatttcacaaataaagc
atttttttcactgcattctagttgtggtttgtccaaactcatcaatgtatcttatcatgtctggctctagctatcccgcccctaactccgcccatcccg
cccctaactccgcccagttccgcccattctccgccccatggctgactaattttttttatttatgcagaggccgaggccgcctcggcctctgagct
attccagaagtagtgaggaggcttttttggaggcctagggacgtacccaattcgccctatagtgagtcgtattacgcgcgctcactggccgtc
gttttacaacgtcgtgactgggaaaaccctggcgttacccaacttaatcgccttgcagcacatccccctttcgccagctggcgtaatagcgaa
gaggcccgcaccgatcgcccttcccaacagttgcgcagcctgaatggcgaatgggacgcgccctgtagcggcgcattaagcgcggcgg
gtgtggtggttacgcgcagcgtgaccgctacacttgccagcgccctagcgcccgctcctttcgctttcttcccttcctttctcgccacgttcgcc
ggctttccccgtcaagctctaaatcgggggctccctttagggttccgatttagtgctttac

Primers for linear backbone fragment B:

catggtggctattccggagtcgacgg

gggctccctttagggttccgatttagtgctttac

Backbone fragment B sequence:

gggctccctttagggttccgatttagtgctttacggcacctcgaccccaaaaaacttgattagggtgatggttcacgtagtgggccatcgcc
ctgatagacggtttttcgccctttgacgttggagtccacgttctttaatagtggactcttgttccaaactggaacaacactcaaccctatctcggtc
tattcttttgatttataagggattttgccgatttcggcctattggttaaaaaatgagctgatttaacaaaaatttaacgcgaattttaacaaaatattaa
cgcttacaatttaggtggcacttttcggggaaatgtgcgcggaacccctatttgtttatttttctaaatacattcaaatatgtatccgctcatgagac

aataaccctgataaatgcttcaataatattgaaaaaggaagagtatgagtattcaacatttccgtgtcgcccttattcccttttttgcggcattttgc

cttcctgtttttgctcacccagaaacgctggtgaaagtaaaagatgctgaagatcagttgggtgcacgagtgggttacatcgaactggatctca

acagcggtaagatccttgagagttttcgccccgaagaacgttttccaatgatgagcacttttaaagttctgctatgtggcgcggtattatcccgt

attgacgccgggcaagagcaactcggtcgccgcatacactattctcagaatgacttggttgagtactcaccagtcacagaaaagcatcttac

ggatggcatgacagtaagagaattatgcagtgctgccataaccatgagtgataacactgcggccaacttacttctgacaacgatcggagga

ccgaaggagctaaccgcttttttgcacaacatgggggatcatgtaactcgccttgatcgttgggaaccggagctgaatgaagccataccaaa

cgacgagcgtgacaccacgatgcctgtagcaatggcaacaacgttgcgcaaactattaactggcgaactacttactctagcttcccggcaac

aattaatagactggatggaggcggataaagttgcaggaccacttctgcgctcggcccttccggctggctggtttattgctgataaatctggag

ccggtgagcgtgggtctcgcggtatcattgcagcactggggccagatggtaagccctcccgtatcgtagttatctacacgacggggagtca

ggcaactatggatgaacgaaatagacagatcgctgagataggtgcctcactgattaagcattggtaactgtcagaccaagtttactcatatata

ctttagattgatttaaaacttcatttttaatttaaaaggatctaggtgaagatcctttttgataatctcatgaccaaaatcccttaacgtgagttttcgtt

ccactgagcgtcagaccccgtagaaaagatcaaaggatcttcttgagatcctttttttctgcgcgtaatctgctgcttgcaaacaaaaaaacca

ccgctaccagcggtggtttgtttgccggatcaagagctaccaactctttttccgaaggtaactggcttcagcagagcgcagataccaaatact

gttcttctagtgtagccgtagttaggccaccacttcaagaactctgtagcaccgcctacatacctcgctctgctaatcctgttaccagtggctgc

tgccagtggcgataagtcgtgtcttaccgggttggactcaagacgatagttaccggataaggcgcagcggtcgggctgaacggggggttc

gtgcacacagcccagcttggagcgaacgacctacaccgaactgagatacctacagcgtgagctatgagaaagcgccacgcttcccgaag

ggagaaaggcggacaggtatccggtaagcggcagggtcggaacaggagagcgcacgagggagcttccagggggaaacgcctggtat

ctttatagtcctgtcgggtttcgccacctctgacttgagcgtcgatttttgtgatgctcgtcaggggggcggagcctatggaaaaacgccagca

acgcggcctttttacggttcctggccttttgctggccttttgctcacatgttctttcctgcgttatcccctgattctgtggataaccgtattaccgcct

ttgagtgagctgataccgctcgccgcagccgaacgaccgagcgcagcgagtcagtgagcgaggaagcggaagagcgcccaatacgca

aaccgcctctccccgcgcgttggccgattcattaatgcagctggcacgacaggtttcccgactggaaagcgggcagtgagcgcaacgcaa

ttaatgtgagttagctcactcattaggcaccccaggctttacactttatgcttccggctcgtatgttgtgtggaattgtgagcggataacaatttca

cacaggaaacagctatgaccatgattacgccaagcgcgcaattaaccctcactaaagggaacaaaagctggagctgcaagcttaatgtagt

cttatgcaatactcttgtagtcttgcaacatggtaacgatgagttagcaacatgccttacaaggagagaaaaagcaccgtgcatgccgattggt

ggaagtaaggtggtacgatcgtgccttattaggaaggcaacagacgggtctgacatggattggacgaaccactgaattgccgcattgcaga

gatattgtatttaagtgcctagctcgatacataaacgggtctctctggttagaccagatctgagcctgggagctctctggctaactagggaacc

cactgcttaagcctcaataaagcttgccttgagtgcttcaagtagtgtgtgcccgtctgttgtgtgactctggtaactagagatccctcagaccc

ttttagtcagtgtggaaaatctctagcagtggcgcccgaacagggacttgaaagcgaaagggaaaccagaggagctctctcgacgcagga

ctcggcttgctgaagcgcgcacggcaagaggcgaggggcggcgactggtgagtacgccaaaaattttgactagcggaggctagaagga

gagagatgggtgcgagagcgtcagtattaagcgggggagaattagatcgcgatgggaaaaaattcggttaaggccagggggaaagaaa

aaatataaattaaaacatatagtatgggcaagcagggagctagaacgattcgcagttaatcctggcctgttagaaacatcagaaggctgtag

acaaatactgggacagctacaaccatcccttcagacaggatcagaagaacttagatcattatataatacagtagcaaccctctattgtgtgcat

caaaggatagagataaaagacaccaaggaagctttagacaagatagaggaagagcaaaacaaaagtaagaccaccgcacagcaagcg

gccgctgatcttcagacctggaggaggagatatgagggacaattggagaagtgaattatataaatataaagtagtaaaaattgaaccattag

gagtagcacccaccaaggcaaagagaagagtggtgcagagagaaaaaagagcagtgggaataggagctttgttccttgggttcttggga

gcagcaggaagcactatgggcgcagcgtcaatgacgctgacggtacaggccagacaattattgtctggtatagtgcagcagcagaacaat

ttgctgagggctattgaggcgcaacagcatctgttgcaactcacagtctggggcatcaagcagctccaggcaagaatcctggctgtggaaa

gatacctaaaggatcaacagctcctggggatttggggttgctctggaaaactcatttgcaccactgctgtgccttggaatgctagttggagtaa

taaatctctggaacagatttggaatcacacgacctggatggagtgggacagagaaattaacaattacacaagcttaatacactccttaattgaa

gaatcgcaaaaccagcaagaaaagaatgaacaagaattattggaattagataaatgggcaagtttgtggaattggtttaacataacaaattgg

ctgtggtatataaaattattcataatgatagtaggaggcttggtaggtttaagaatagtttttgctgtactttctatagtgaatagagttaggcaggg

atattcaccattatcgtttcagacccacctcccaaccccgaggggacccttgcgccttttccaaggcagccctgggtttgcgcagggacgcg

gctgctctgggcgtggttccgggaaacgcagcggcgccgaccctgggtctcgcacattcttcacgtccgttcgcagcgtcacccggatctt

cgccgctacccttgtgggccccccggcgacgcttcctgctccgcccctaagtcgggaaggttccttgcggttcgcggcgtgccggacgtg

acaaacggaagccgcacgactcactagtaccctcgcagacggacagcgccagggagcaatggcagcgcgccgaccgcgatgggctgt

ggccaatagcggctgctcagcagggcgcgccgagagcagcggccgggaaggggcggtgcgggaggcggggtgtggggcggtagtg

tgggccctgttcctgcccgcgcggtgttccgcattctgcaagcctccggagcgcacgtcggcagtcggctccctcgttgaccgaatcaccg
acctctctccccagggatcgataccgtcgactccggaatagccaccatg

Plasmid: HA-NanoLuc-WEIS

Backbone: Cil2_no_eGFP (A and B)

Insert sequence cloned into Cil2_no_eGFP:

Ccgtcgactccggaatagccaccatgtacccatacgatgtcccggactatgctggtggcgtattcacactcgaagatttcgttggcgattg
gaggcaaacggcgggttacaatcttgaccaggttctggaacaaggtggcgtgagctctcttccagaacttgggcgtttcagtaactccaat
ccagcggatcgtgctcagtggggaaaatggcctcaaaatagacatacacgtaataataccctatgaaggactgtccggtgaccaaatgggg
caaatcgaaaagatttttaaagtcgtttacccagtggatgaccatcacttcaaggtcatccttcactacgggactctcgtcatagatggggttac
gcctaacatgatcgattattttggacgcccttacgaaggaattgccgtgttcgatgggaaaaagattactgttaccggcacactgtggaatgg
gaacaagataattgacgaacggttgataaatcccgacgggtcactcctgttccgggtcactataaatggggtcaccggatggagactttgcg
aaagaatacttgctggcgggggtgggtccggtggaggtggatccttcaacgtattgatggtccataaaagatcacacacgggagaaagac
cattctggtgtgagatatgcggggcttcctttacccaaaaaggtaatctgctcagacatatcaagttgcacacgggtgagaaacccttcaagt
gccacttgtgtaactatgcttgccagcgccgggacgctctgtaaatgcatgagtaactgaggatccgcccctc

Full sequence:

cgaacgaccgagcgcagcgagtcagtgagcgaggaagcggaagagcgcccaatacgcaaaccgcctctccccgcgcgttggccga
ttcattaatgcagctggcacgacaggtttcccgactggaaagcgggcagtgagcgcaacgcaattaatgtgagttagctcactcattaggca
ccccaggctttacactttatgcttccggctcgtatgttgtgtggaattgtgagcggataacaatttcacacaggaaacagctatgaccatgatta

cgccaagcgcgcaattaaccctcactaaagggaacaaaagctggagctgcaagcttaatgtagtcttatgcaatactcttgtagtcttgcaac atggtaacgatgagttagcaacatgccttacaaggagagaaaaagcaccgtgcatgccgattggtggaagtaaggtggtacgatcgtgcct tattaggaaggcaacagacgggtctgacatggattggacgaaccactgaattgccgcattgcagagatattgtatttaagtgcctagctcgat acataaacgggtctctctggttagaccagatctgagcctgggagctctctggctaactagggaacccactgcttaagcctcaataaagcttgc cttgagtgcttcaagtagtgtgtgcccgtctgttgtgtgactctggtaactagagatccctcagacccttttagtcagtgtggaaaatctctagca gtggcgcccgaacagggacttgaaagcgaaagggaaaccagaggagctctctcgacgcaggactcggcttgctgaagcgcgcacggc aagaggcgaggggcggcgactggtgagtacgccaaaaattttgactagcggaggctagaaggagagagatgggtgcgagagcgtcagt attaagcgggggagaattagatcgcgatgggaaaaaattcggttaaggccagggggaaagaaaaaatataaattaaaacatatagtatggg caagcagggagctagaacgattcgcagttaatcctggcctgttagaaacatcagaaggctgtagacaaatactgggacagctacaaccatc ccttcagacaggatcagaagaacttagatcattatataatacagtagcaaccctctattgtgtgcatcaaaggatagagataaaagacaccaa ggaagctttagacaagatagaggaagagcaaaacaaaagtaagaccaccgcacagcaagcggccgctgatcttcagacctggaggagg agatatgagggacaattggagaagtgaattatataaatataaagtagtaaaaattgaaccattaggagtagcacccaccaaggcaaagaga agagtggtgcagagagaaaaaagagcagtgggaataggagctttgttccttgggttcttgggagcagcaggaagcactatgggcgcagc gtcaatgacgctgacggtacaggccagacaattattgtctggtatagtgcagcagcagaacaatttgctgagggctattgaggcgcaacag catctgttgcaactcacagtctggggcatcaagcagctccaggcaagaatcctggctgtggaaagatacctaaaggatcaacagctcctgg ggatttggggttgctctggaaaactcatttgcaccactgctgtgccttggaatgctagttggagtaataaatctctggaacagatttggaatcac acgacctggatggagtgggacagagaaattaacaattacacaagcttaatacactccttaattgaagaatcgcaaaaccagcaagaaaaga atgaacaagaattattggaattagataaatgggcaagtttgtggaattggtttaacataacaaattggctgtggtatataaaattattcataatgat agtaggaggcttggtaggtttaagaatagtttttgctgtactttctatagtgaatagagttaggcagggatattcaccattatcgtttcagacccac ctcccaaccccgaggggacccttgcgccttttccaaggcagccctgggtttgcgcagggacgcggctgctctgggcgtggttccgggaaa cgcagcggcgccgaccctgggtctcgcacattcttcacgtccgttcgcagcgtcacccggatcttcgccgctacccttgtgggccccccgg cgacgcttcctgctccgcccctaagtcgggaaggttccttgcggttcgcggcgtgccggacgtgacaaacggaagccgcacgactcacta gtaccctcgcagacggacagcgccagggagcaatggcagcgcgccgaccgcgatgggctgtggccaatagcggctgctcagcagggc

gcgccgagagcagcggccgggaaggggcggtgcgggaggcggggtgtggggcggtagtgtgggccctgttcctgcccgcgcggtgt

tccgcattctgcaagcctccggagcgcacgtcggcagtcggctccctcgttgaccgaatcaccgacctctctccccagggatcgataccgt

cgactccggaatagccaccatgtacccatacgatgtcccggactatgctggtggcgtattcacactcgaagatttcgttggcgattggaggc

aaacggcgggttacaatcttgaccaggttctggaacaaggtggcgtgagctctctcttccagaacttgggcgtttcagtaactccaatccagc

ggatcgtgctcagtggggaaaatggcctcaaaatagacatacacgtaataataccctatgaaggactgtccggtgaccaaatggggcaaat

cgaaaagatttttaaagtcgtttacccagtggatgaccatcacttcaaggtcatccttcactacgggactctcgtcatagatggggttacgccta

acatgatcgattattttggacgcccttacgaaggaattgccgtgttcgatgggaaaaagattactgttaccggcacactgtggaatgggaaca

agataattgacgaacggttgataaatcccgacgggtcactcctgttccgggtcactataaatggggtcaccggatggagactttgcgaaaga

atacttgctggcgggggtgggtccggtggaggtggatccttcaacgtattgatggtccataaaagatcacacacgggagaaagaccattct

ggtgtgagatatgcggggcttcctttacccaaaaaggtaatctgctcagacatatcaagttgcacacgggtgagaaacccttcaagtgccact

tgtgtaactatgcttgccagcgccgggacgctctgtaaatgcatgagtaactgaggatccgcccctctccctcccccccccctaacgttactg

gccgaagccgcttggaataaggccggtgtgcgtttgtctatatgttattttccaccatattgccgtcttttggcaatgtgagggcccggaaacct

ggccctgtcttcttgacgagcattcctaggggtctttcccctctcgccaaaggaatgcaaggtctgttgaatgtcgtgaaggaagcagttcctc

tggaagcttcttgaagacaaacaacgtctgtagcgaccctttgcaggcagcggaaccccccacctggcgacaggtgcctctgcggccaaa

agccacgtgtataagatacacctgcaaaggcggcacaaccccagtgccacgttgtgagttggatagttgtggaaagagtcaaatggctctc

ctcaagcgtattcaacaaggggctgaaggatgcccagaaggtaccccattgtatgggatctgatctggggcctcggtacacatgctttacat

gtgtttagtcgaggttaaaaaaacgtctaggccccccgaaccacggggacgtggttttcctttgaaaaacacgatgataatatggccacaac

cctggaattcgccaccatggtgagcaagggcgaggaggataacatggccatcatcaaggagttcatgcgcttcaaggtgcacatggaggg

ctccgtgaacggccacgagttcgagatcgagggcgagggcgagggccgcccctacgagggcacccagaccgccaagctgaaggtga

ccaagggtggccccctgcccttcgcctgggacatcctgtcccctcagttcatgtacggctccaaggcctacgtgaagcaccccgccgacat

ccccgactacttgaagctgtccttccccgagggcttcaagtgggagcgcgtgatgaacttcgaggacggcggcgtggtgaccgtgaccca

ggactcctccctgcaggacggcgagttcatctacaaggtgaagctgcgcggcaccaacttcccctccgacggccccgtaatgcagaagaa

gaccatgggctgggaggcctcctccgagcggatgtaccccgaggacggcgccctgaagggcgagatcaagcagaggctgaagctgaa

ggacggcggccactacgacgctgaggtcaagaccacctacaaggccaagaagcccgtgcagctgcccggcgcctacaacgtcaacatc
aagttggacatcacctcccacaacgaggactacaccatcgtggaacagtacgaacgcgccgagggccgccactccaccggcggcatgg
acgagctgtacaagtaaactagtaagcttggcgtaactagatcttgagacaaatggcagtattcatccacaattttaaaagaaaaggggggat
tggggggtacagtgcaggggaaagaatagtagacataatagcaacagacatacaaactaaagaattacaaaaacaaattacaaaaattcaa
aattttcgggtttattacagggacagcagagatccactttgggctcgagggggcccgggtgcaaagatggataaagttttaaacagagagg
aatctttgcagctaatggaccttctaggtcttgaaaggagtgggaattggctccggtgcccgtcagtgggcagagcgcacatcgcccacagt
ccccgagaagttggggggaggggtcggcaattgatccggtgcctagagaaggtggcgcggggtaaactgggaaagtgatgtcgtgtact
ggctccgcctttttcccgagggtgggggagaaccgtatataagtgcagtagtcgccgtgaacgttctttttcgcaacgggtttgccgccagaa
cacaggtaagtgccgtgtgtggttcccgcgggcctggcctctttacgggttatggcccttgcgtgccttgaattacttccacctggctgcagta
cgtgattcttgatcccgagcttcgggttggaagtgggtgggagagttcgaggccttgcgcttaaggagccccttcgcctcgtgcttgagttga
ggcctggcctgggcgctggggccgccgcgtgcgaatctggtggcaccttcgcgcctgtctcgctgctttcgataagtctctagccatttaaa
atttttgatgacctgctgcgacgctttttttctggcaagatagtcttgtaaatgcgggccaagatctgcacactggtatttcggtttttggggccgc
gggcggcgacggggcccgtgcgtcccagcgcacatgttcggcgaggcggggcctgcgagcgcggccaccgagaatcggacggggg
tagtctcaagctggccggcctgctctggtgcctggcctcgcgccgccgtgtatcgccccgccctgggcggcaaggctggcccggtcggc
accagttgcgtgagcggaaagatggccgcttcccggccctgctgcagggagctcaaaatggaggacgcggcgctcgggagagcgggc
gggtgagtcacccacacaaaggaaaagggcctttccgtcctcagccgtcgcttcatgtgactccacggagtaccgggcgccgtccaggca
cctcgattagttctcgagcttttggagtacgtcgtctttaggttggggggaggggttttatgcgatggagtttccccacactgagtgggtggag
actgaagttaggccagcttggcacttgatgtaattctccttggaatttgccctttttgagtttggatcttggttcattctcaagcctcagacagtggt
tcaaagtttttttcttccatttcaggtgtcgtgacgtacggccaccatgaccgagtacaagcccacggtgcgcctcgccacccgcgacgacgt
ccccagggccgtacgcaccctcgccgccgcgttcgccgactaccccgccacgcgccacaccgtcgatccggaccgccacatcgagcg
ggtcaccgagctgcaagaactcttcctcacgcgcgtcgggctcgacatcggcaaggtgtgggtcgcggacgacggcgccgccgtggcg
gtctggaccacgccggagagcgtcgaagcgggggcggtgttcgccgagatcggcccgcgcatggccgagttgagcggttcccggctg
gccgcgcagcaacagatggaaggcctcctggcgccgcaccggcccaaggagcccgcgtggttcctggccaccgtcggagtctcgccc

gaccaccagggcaagggtctgggcagcgccgtcgtgctccccggagtggaggcggccgagcgcgccggggtgcccgccttcctgga

gacctccgcgccccgcaacctccccttctacgagcggctcggcttcaccgtcaccgccgacgtcgaggtgcccgaaggaccgcgcacct

ggtgcatgacccgcaagcccggtgcctgaacgcgttaagtcgacaatcaacctctggattacaaaatttgtgaaagattgactggtattcttaa

ctatgttgctccttttacgctatgtggatacgctgctttaatgcctttgtatcatgctattgcttcccgtatggctttcattttctcctccttgtataaatc

ctggttgctgtctctttatgaggagttgtggcccgttgtcaggcaacgtggcgtggtgtgcactgtgtttgctgacgcaacccccactggttgg

ggcattgccaccacctgtcagctcctttccgggactttcgctttccccctccctattgccacggcggaactcatcgccgcctgccttgcccgct

gctggacaggggctcggctgttgggcactgacaattccgtggtgttgtcggggaaatcatcgtcctttccttggctgctcgcctgtgttgccac

ctggattctgcgcgggacgtccttctgctacgtcccttcggccctcaatccagcggaccttccttcccgcggcctgctgccggctctgcggc

ctcttccgcgtcttcgccttcgccctcagacgagtcggatctccctttgggccgcctccccgcgtcgactttaagaccaatgacttacaaggc

agctgtagatcttagccactttttaaaagaaaaggggggactggaagggctaattcactcccaacgaagacaagatctgctttttgcttgtact

gggtctctctggttagaccagatctgagcctgggagctctctggctaactagggaacccactgcttaagcctcaataaagcttgccttgagtg

cttcaagtagtgtgtgcccgtctgttgtgtgactctggtaactagagatccctcagacccttttagtcagtgtggaaaatctctagcagtacgtat

agtagttcatgtcatcttattattcagtatttataacttgcaaagaaatgaatatcagagagtgagaggaacttgtttattgcagcttataatggtta

caaataaagcaatagcatcacaaatttcacaaataaagcatttttttcactgcattctagttgtggtttgtccaaactcatcaatgtatcttatcatgt

ctggctctagctatcccgcccctaactccgcccatcccgcccctaactccgcccagttccgcccattctccgccccatggctgactaattttttt

tatttatgcagaggccgaggccgcctcggcctctgagctattccagaagtagtgaggaggcttttttggaggcctagggacgtacccaattc

gccctatagtgagtcgtattacgcgcgctcactggccgtcgttttacaacgtcgtgactgggaaaaccctggcgttacccaacttaatcgcctt

gcagcacatccccctttcgccagctggcgtaatagcgaagaggcccgcaccgatcgcccttcccaacagttgcgcagcctgaatggcgaa

tgggacgcgccctgtagcggcgcattaagcgcggcgggtgtggtggttacgcgcagcgtgaccgctacacttgccagcgccctagcgcc

cgctcctttcgctttcttcccttcctttctcgccacgttcgccggctttccccgtcaagctctaaatcgggggctccctttagggttccgatttagtg

ctttacggcacctcgaccccaaaaaacttgattagggtgatggttcacgtagtgggccatcgccctgatagacggtttttcgccctttgacgtt

ggagtccacgttctttaatagtggactcttgttccaaactggaacaacactcaaccctatctcggtctattcttttgatttataagggattttgccga

tttcggcctattggttaaaaaatgagctgatttaacaaaaatttaacgcgaattttaacaaaatattaacgcttacaatttaggtggcacttttcggg

gaaatgtgcgcggaacccctatttgtttatttttctaaatacattcaaatatgtatccgctcatgagacaataaccctgataaatgcttcaataatat
tgaaaaaggaagagtatgagtattcaacatttccgtgtcgcccttattcccttttttgcggcattttgccttcctgtttttgctcacccagaaacgct
ggtgaaagtaaaagatgctgaagatcagttgggtgcacgagtgggttacatcgaactggatctcaacagcggtaagatccttgagagttttc
gccccgaagaacgttttccaatgatgagcacttttaaagttctgctatgtggcgcggtattatcccgtattgacgccgggcaagagcaactcg
gtcgccgcatacactattctcagaatgacttggttgagtactcaccagtcacagaaaagcatcttacggatggcatgacagtaagagaattat
gcagtgctgccataaccatgagtgataacactgcggccaacttacttctgacaacgatcggaggaccgaaggagctaaccgcttttttgcac
aacatgggggatcatgtaactcgccttgatcgttgggaaccggagctgaatgaagccataccaaacgacgagcgtgacaccacgatgcct
gtagcaatggcaacaacgttgcgcaaactattaactggcgaactacttactctagcttcccggcaacaattaatagactggatggaggcgga
taaagttgcaggaccacttctgcgctcggcccttccggctggctggtttattgctgataaatctggagccggtgagcgtgggtctcgcggtat
cattgcagcactggggccagatggtaagccctcccgtatcgtagttatctacacgacggggagtcaggcaactatggatgaacgaaataga
cagatcgctgagataggtgcctcactgattaagcattggtaactgtcagaccaagtttactcatatatactttagattgatttaaaacttcattttta
atttaaaaggatctaggtgaagatcctttttgataatctcatgaccaaaatcccttaacgtgagttttcgttccactgagcgtcagaccccgtaga
aaagatcaaaggatcttcttgagatcctttttttctgcgcgtaatctgctgcttgcaaacaaaaaaaccaccgctaccagcggtggtttgtttgcc
ggatcaagagctaccaactctttttccgaaggtaactggcttcagcagagcgcagataccaaatactgttcttctagtgtagccgtagttaggc
caccacttcaagaactctgtagcaccgcctacatacctcgctctgctaatcctgttaccagtggctgctgccagtggcgataagtcgtgtctta
ccgggttggactcaagacgatagttaccggataaggcgcagcggtcgggctgaacggggggttcgtgcacacagcccagcttggagcg
aacgacctacaccgaactgagatacctacagcgtgagctatgagaaagcgccacgcttcccgaagggagaaaggcggacaggtatccg
gtaagcggcagggtcggaacaggagagcgcacgagggagcttccagggggaaacgcctggtatctttatagtcctgtcgggtttcgccac
ctctgacttgagcgtcgatttttgtgatgctcgtcaggggggcggagcctatggaaaaacgccagcaacgcggcctttttacggttcctggcc
ttttgctggccttttgctcacatgttctttcctgcgttatcccctgattctgtggataaccgtattaccgcctttgagtgagctgataccgctcgccg
cagc

Plasmid map:

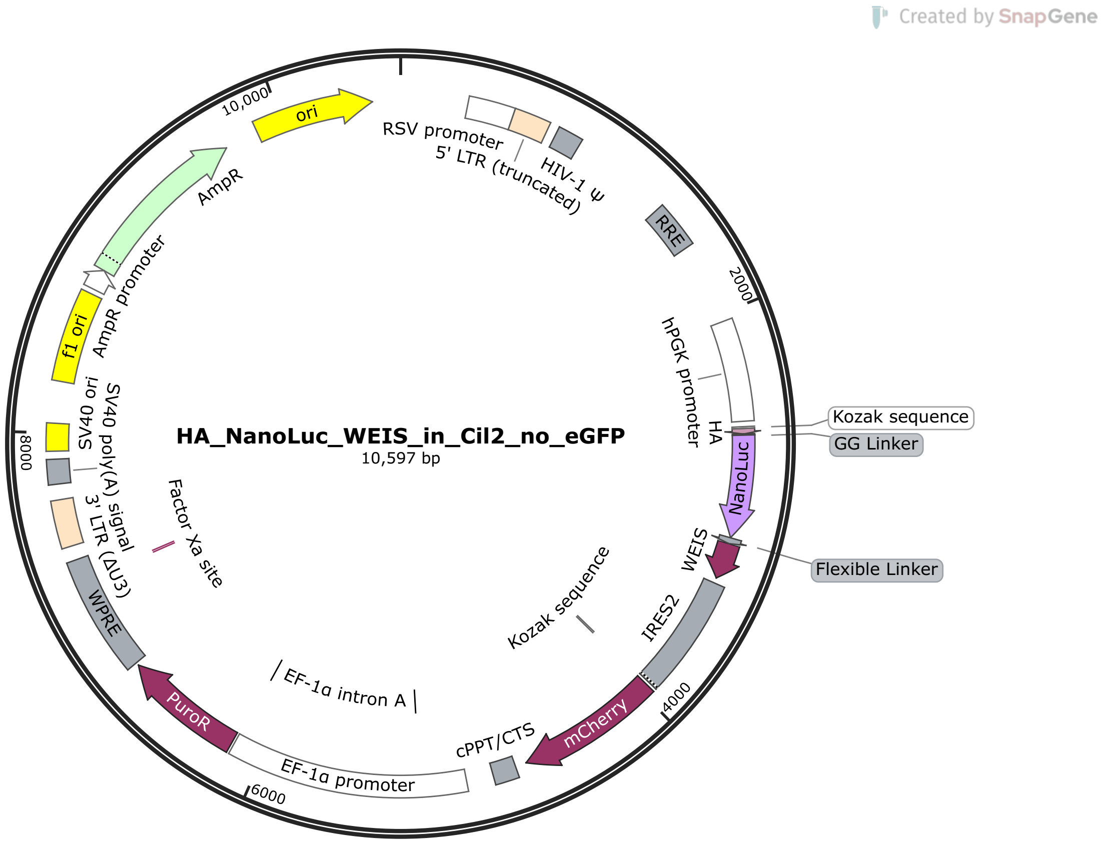

Plasmid: HA-CDK9-WEIS

Backbone: Cil2_no_eGFP (A and B)

Insert sequence cloned into Cil2_no_eGFP:

ccgtcgactccggaatagccaccatgtacccttatgacgttccagactatgccggcggtgcaaagcaatatgatagcgtagagtgtccctt
ctgtgatgaggtttctaaatatgagaaactggccaaaattggacagggaaccttcggagaagtgtttaaggcccggcatcgcaaaacggga
caaaaggtcgccctcaagaaagtattgatggagaacgagaaagagggctttcctatcacagcgctgcgcgaaataaagatccttcaactcc

tgaaacatgagaatgtagtcaacctgattgagatctgccggactaaggcgagcccatataatcggtgtaaagggagcatctatcttgttttcga
cttttgcgaacacgacttggctggcctgttatctaatgtgttggttaaatttacgctgtcagaaataaagcgcgtcatgcagatgctcctgaatgg
tctgtattacattcatcgcaacaaaatacttcatagagacatgaaagctgctaacgtcctcataacgcgggacggcgtgttgaagttggctgat
tttggtctggcaagagcattttccttagccaagaacagtcagcccaaccgctacactaaccgggttgtgactctttggtacagacccccagaa
cttttactcggtgaacgggattatgggccaccaatcgacctttggggcgccgggtgcatcatggcagaaatgtggaccaggagtcccattat
gcagggtaacaccgagcaacatcaacttgcgttaattagccaattatgcgggtccatcacccctgaggtgtggccaaacgtagataactatg
aactgtacgaaaagctggagctcgttaaggggcagaaacgtaaagtgaaagaccggcttaaagcttacgtgagggatccctatgcgctcg
accttatcgataaattgttagtgcttgacccggcccaaaggattgatagcgacgacgctctgaaccatgacttcttttggagtgatccaatgcc
atcagacctcaaaggaatgctctccacgcatctcacttccatgtttgagtatcttgccccgccgcgacgcaagggaagtcagatcacacagc
aatcaactaaccagtccagaaaccccgcgaccactaaccaaactgagttcgagcgggtattcggtggcggcggctcaggtggcggcgga
agtttcaacgtattaatggtccacaaacggagccatacaggggaacggcccttttggtgtgaaatctgcggtgcttccttcacacaaaaggga
aatctcctgaggcatataaagttgcacacaggagaaaagcctttcaaatgccatctttgtaactatgcctgccaaagacgagatgctttgtaaa
tgcatgagtaactgaggatccgcccctc

Full sequence:

cgaacgaccgagcgcagcgagtcagtgagcgaggaagcggaagagcgcccaatacgcaaaccgcctctccccgcgcgttggccga
ttcattaatgcagctggcacgacaggtttcccgactggaaagcgggcagtgagcgcaacgcaattaatgtgagttagctcactcattaggca
ccccaggctttacactttatgcttccggctcgtatgttgtgtggaattgtgagcggataacaatttcacacaggaaacagctatgaccatgatta
cgccaagcgcgcaattaaccctcactaaagggaacaaaagctggagctgcaagcttaatgtagtcttatgcaatactcttgtagtcttgcaac
atggtaacgatgagttagcaacatgccttacaaggagagaaaaagcaccgtgcatgccgattggtggaagtaaggtggtacgatcgtgcct
tattaggaaggcaacagacgggtctgacatggattggacgaaccactgaattgccgcattgcagagatattgtatttaagtgcctagctcgat
acataaacgggtctctctggttagaccagatctgagcctgggagctctctggctaactagggaacccactgcttaagcctcaataaagcttgc

cttgagtgcttcaagtagtgtgtgcccgtctgttgtgtgactctggtaactagagatccctcagacccttttagtcagtgtggaaaatctctagca

gtggcgcccgaacagggacttgaaagcgaaagggaaaccagaggagctctctcgacgcaggactcggcttgctgaagcgcgcacggc

aagaggcgaggggcggcgactggtgagtacgccaaaaattttgactagcggaggctagaaggagagagatgggtgcgagagcgtcagt

attaagcgggggagaattagatcgcgatgggaaaaaattcggttaaggccagggggaaagaaaaaatataaattaaaacatatagtatggg

caagcagggagctagaacgattcgcagttaatcctggcctgttagaaacatcagaaggctgtagacaaatactgggacagctacaaccatc

ccttcagacaggatcagaagaacttagatcattatataatacagtagcaaccctctattgtgtgcatcaaaggatagagataaaagacaccaa

ggaagctttagacaagatagaggaagagcaaaacaaaagtaagaccaccgcacagcaagcggccgctgatcttcagacctggaggagg

agatatgagggacaattggagaagtgaattatataaatataaagtagtaaaaattgaaccattaggagtagcacccaccaaggcaaagaga

agagtggtgcagagagaaaaaagagcagtgggaataggagctttgttccttgggttcttgggagcagcaggaagcactatgggcgcagc

gtcaatgacgctgacggtacaggccagacaattattgtctggtatagtgcagcagcagaacaatttgctgagggctattgaggcgcaacag

catctgttgcaactcacagtctggggcatcaagcagctccaggcaagaatcctggctgtggaaagatacctaaaggatcaacagctcctgg

ggatttggggttgctctggaaaactcatttgcaccactgctgtgccttggaatgctagttggagtaataaatctctggaacagatttggaatcac

acgacctggatggagtgggacagagaaattaacaattacacaagcttaatacactccttaattgaagaatcgcaaaaccagcaagaaaaga

atgaacaagaattattggaattagataaatgggcaagtttgtggaattggtttaacataacaaattggctgtggtatataaaattattcataatgat

agtaggaggcttggtaggtttaagaatagtttttgctgtactttctatagtgaatagagttaggcagggatattcaccattatcgtttcagacccac

ctcccaaccccgaggggacccttgcgccttttccaaggcagccctgggtttgcgcagggacgcggctgctctgggcgtggttccgggaaa

cgcagcggcgccgaccctgggtctcgcacattcttcacgtccgttcgcagcgtcacccggatcttcgccgctacccttgtgggccccccgg

cgacgcttcctgctccgcccctaagtcgggaaggttccttgcggttcgcggcgtgccggacgtgacaaacggaagccgcacgactcacta

gtaccctcgcagacggacagcgccagggagcaatggcagcgcgccgaccgcgatgggctgtggccaatagcggctgctcagcagggc

gcgccgagagcagcggccgggaaggggcggtgcgggaggcggggtgtggggcggtagtgtgggccctgttcctgcccgcgcggtgt

tccgcattctgcaagcctccggagcgcacgtcggcagtcggctccctcgttgaccgaatcaccgacctctctccccagggatcgataccgt

cgactccggaatagccaccatgtacccttatgacgttccagactatgccggcggtgcaaagcaatatgatagcgtagagtgtcccttctgtga

tgaggtttctaaatatgagaaactggccaaaattggacagggaaccttcggagaagtgtttaaggcccggcatcgcaaaacgggacaaaa

ggtcgccctcaagaaagtattgatggagaacgagaaagagggctttcctatcacagcgctgcgcgaaataaagatccttcaactcctgaaa

catgagaatgtagtcaacctgattgagatctgccggactaaggcgagcccatataatcggtgtaaagggagcatctatcttgttttcgacttttg

cgaacacgacttggctggcctgttatctaatgtgttggttaaatttacgctgtcagaaataaagcgcgtcatgcagatgctcctgaatggtctgt

attacattcatcgcaacaaaatacttcatagagacatgaaagctgctaacgtcctcataacgcgggacggcgtgttgaagttggctgattttgg

tctggcaagagcattttccttagccaagaacagtcagcccaaccgctacactaaccgggttgtgactctttggtacagacccccagaactttta

ctcggtgaacgggattatgggccaccaatcgacctttggggcgccgggtgcatcatggcagaaatgtggaccaggagtcccattatgcag

ggtaacaccgagcaacatcaacttgcgttaattagccaattatgcgggtccatcacccctgaggtgtggccaaacgtagataactatgaactg

tacgaaaagctggagctcgttaaggggcagaaacgtaaagtgaaagaccggcttaaagcttacgtgagggatccctatgcgctcgacctta

tcgataaattgttagtgcttgacccggcccaaaggattgatagcgacgacgctctgaaccatgacttcttttggagtgatccaatgccatcaga

cctcaaaggaatgctctccacgcatctcacttccatgtttgagtatcttgccccgccgcgacgcaagggaagtcagatcacacagcaatcaa

ctaaccagtccagaaaccccgcgaccactaaccaaactgagttcgagcgggtattcggtggcggcggctcaggtggcggcggaagtttc

aacgtattaatggtccacaaacggagccatacaggggaacggcccttttggtgtgaaatctgcggtgcttccttcacacaaaagggaaatct

cctgaggcatataaagttgcacacaggagaaaagcctttcaaatgccatctttgtaactatgcctgccaaagacgagatgctttgtaaatgcat

gagtaactgaggatccgcccctctccctcccccccccctaacgttactggccgaagccgcttggaataaggccggtgtgcgtttgtctatatg

ttattttccaccatattgccgtcttttggcaatgtgagggcccggaaacctggccctgtcttcttgacgagcattcctaggggtctttcccctctcg

ccaaaggaatgcaaggtctgttgaatgtcgtgaaggaagcagttcctctggaagcttcttgaagacaaacaacgtctgtagcgaccctttgca

ggcagcggaaccccccacctggcgacaggtgcctctgcggccaaaagccacgtgtataagatacacctgcaaaggcggcacaacccca

gtgccacgttgtgagttggatagttgtggaaagagtcaaatggctctcctcaagcgtattcaacaaggggctgaaggatgcccagaaggtac

cccattgtatgggatctgatctggggcctcggtacacatgctttacatgtgtttagtcgaggttaaaaaaacgtctaggccccccgaaccacg

gggacgtggttttcctttgaaaaacacgatgataatatggccacaaccctggaattcgccaccatggtgagcaagggcgaggaggataaca

tggccatcatcaaggagttcatgcgcttcaaggtgcacatggagggctccgtgaacggccacgagttcgagatcgagggcgagggcgag

ggccgcccctacgagggcacccagaccgccaagctgaaggtgaccaagggtggcccccctgcccttcgcctgggacatcctgtcccctca

gttcatgtacggctccaaggcctacgtgaagcaccccgccgacatccccgactacttgaagctgtccttccccgagggcttcaagtgggag

cgcgtgatgaacttcgaggacggcggcgtggtgaccgtgacccaggactcctccctgcaggacggcgagttcatctacaaggtgaagct

gcgcggcaccaacttcccctccgacggccccgtaatgcagaagaagaccatgggctgggaggcctcctccgagcggatgtaccccgag

gacggcgccctgaagggcgagatcaagcagaggctgaagctgaaggacggcggccactacgacgctgaggtcaagaccacctacaag

gccaagaagcccgtgcagctgcccggcgcctacaacgtcaacatcaagttggacatcacctcccacaacgaggactacaccatcgtgga

acagtacgaacgcgccgagggccgccactccaccggcggcatggacgagctgtacaagtaaactagtaagcttggcgtaactagatcttg

agacaaatggcagtattcatccacaattttaaaagaaaagggggggattggggggtacagtgcaggggaaagaatagtagacataatagca

acagacatacaaactaaagaattacaaaaacaaattacaaaaattcaaaattttcgggtttattacagggacagcagagatccactttgggctc

gagggggcccgggtgcaaagatggataaagttttaaacagagaggaatctttgcagctaatggaccttctaggtcttgaaaggagtgggaa

ttggctccggtgcccgtcagtgggcagagcgcacatcgcccacagtccccgagaagttggggggaggggtcggcaattgatccggtgcc

tagagaaggtggcgcggggtaaactgggaaagtgatgtcgtgtactggctccgcctttttcccgagggtgggggagaaccgtatataagtg

cagtagtcgccgtgaacgttctttttcgcaacgggtttgccgccagaacacaggtaagtgccgtgtgtggttcccgcgggcctggcctcttta

cgggttatggcccttgcgtgccttgaattacttccacctggctgcagtacgtgattcttgatcccgagcttcgggttggaagtgggtgggaga

gttcgaggccttgcgcttaaggagccccttcgcctcgtgcttgagttgaggcctggcctgggcgctggggccgccgcgtgcgaatctggtg

gcaccttcgcgcctgtctcgctgctttcgataagtctctagccatttaaaatttttgatgacctgctgcgacgctttttttctggcaagatagtcttgt

aaatgcgggccaagatctgcacactggtatttcggtttttggggccgcgggcggcgacggggcccgtgcgtcccagcgcacatgttcggc

gaggcggggcctgcgagcgcggccaccgagaatcggacgggggtagtctcaagctggccggcctgctctggtgcctggcctcgcgcc

gccgtgtatcgccccgccctgggcggcaaggctggcccggtcggcaccagttgcgtgagcggaaagatggccgcttcccggccctgct

gcagggagctcaaaatggaggacgcggcgctcgggagagcgggcgggtgagtcacccacacaaaggaaaagggcctttccgtcctca

gccgtcgcttcatgtgactccacggagtaccgggcgccgtccaggcacctcgattagttctcgagcttttggagtacgtcgtctttaggttgg

ggggaggggttttatgcgatggagtttccccacactgagtgggtggagactgaagttaggccagcttggcacttgatgtaattctccttggaa

tttgccctttttgagtttggatcttggttcattctcaagcctcagacagtggttcaaagtttttttcttccatttcaggtgtcgtgacgtacggccacc

atgaccgagtacaagcccacggtgcgcctcgccacccgcgacgacgtccccagggccgtacgcaccctcgccgccgcgttcgccgact

accccgccacgcgccacaccgtcgatccggaccgccacatcgagcgggtcaccgagctgcaagaactcttcctcacgcgcgtcgggctc

gacatcggcaaggtgtgggtcgcggacgacggcgccgccgtggcggtctggaccacgccggagagcgtcgaagcgggggcggtgtt
cgccgagatcggcccgcgcatggccgagttgagcggttcccggctggccgcgcagcaacagatggaaggcctcctggcgccgcaccg
gcccaaggagcccgcgtggttcctggccaccgtcggagtctcgcccgaccaccagggcaagggtctgggcagcgccgtcgtgctcccc
ggagtggaggcggccgagcgcgccggggtgcccgccttcctggagacctccgcgccccgcaacctccccttctacgagcggctcggct
tcaccgtcaccgccgacgtcgaggtgcccgaaggaccgcgcacctggtgcatgacccgcaagcccggtgcctgaacgcgttaagtcga
caatcaacctctggattacaaaatttgtgaaagattgactggtattcttaactatgttgctccttttacgctatgtggatacgctgctttaatgccttt
gtatcatgctattgcttcccgtatggctttcattttctcctccttgtataaatcctggttgctgtctctttatgaggagttgtggcccgttgtcaggcaa
cgtggcgtggtgtgcactgtgtttgctgacgcaacccccactggttggggcattgccaccacctgtcagctcctttccgggactttcgctttcc
ccctccctattgccacggcggaactcatcgccgcctgccttgcccgctgctggacaggggctcggctgttgggcactgacaattccgtggt
gttgtcggggaaatcatcgtcctttccttggctgctcgcctgtgttgccacctggattctgcgcgggacgtccttctgctacgtcccttcggccc
tcaatccagcggaccttccttcccgcggcctgctgccggctctgcggcctcttccgcgtcttcgccttcgccctcagacgagtcggatctccc
tttgggccgcctccccgcgtcgactttaagaccaatgacttacaaggcagctgtagatcttagccactttttaaaagaaaaggggggactgga
agggctaattcactcccaacgaagacaagatctgctttttgcttgtactgggtctctctggttagaccagatctgagcctgggagctctctggct
aactagggaacccactgcttaagcctcaataaagcttgccttgagtgcttcaagtagtgtgtgcccgtctgttgtgtgactctggtaactagag
atccctcagacccttttagtcagtgtggaaaatctctagcagtacgtatagtagttcatgtcatcttattattcagtatttataacttgcaaagaaat
gaatatcagagagtgagaggaacttgtttattgcagcttataatggttacaaataaagcaatagcatcacaaatttcacaaataaagcatttttttc
actgcattctagttgtggtttgtccaaactcatcaatgtatcttatcatgtctggctctagctatcccgcccctaactccgcccatcccgcccctaa
ctccgcccagttccgcccattctccgccccatggctgactaattttttttatttatgcagaggccgaggccgcctcggcctctgagctattccag
aagtagtgaggaggcttttttggaggcctagggacgtacccaattcgccctatagtgagtcgtattacgcgcgctcactggccgtcgttttaca
acgtcgtgactgggaaaaccctggcgttacccaacttaatcgccttgcagcacatccccctttcgccagctggcgtaatagcgaagaggcc
cgcaccgatcgcccttcccaacagttgcgcagcctgaatggcgaatgggacgcgccctgtagcggcgcattaagcgcggcgggtgtggt
ggttacgcgcagcgtgaccgctacacttgccagcgccctagcgcccgctcctttcgctttcttcccttcctttctcgccacgttcgccggctttc
cccgtcaagctctaaatcgggggctccctttagggttccgatttagtgctttacggcacctcgaccccaaaaaacttgattagggtgatggttc

acgtagtgggccatcgccctgatagacggtttttcgccctttgacgttggagtccacgttctttaatagtggactcttgttccaaactggaacaa
cactcaaccctatctcggtctattcttttgatttataagggattttgccgatttcggcctattggttaaaaaatgagctgatttaacaaaaatttaacg
cgaattttaacaaaatattaacgcttacaatttaggtggcacttttcggggaaatgtgcgcggaacccctatttgtttatttttctaaatacattcaa
atatgtatccgctcatgagacaataaccctgataaatgcttcaataatattgaaaaaggaagagtatgagtattcaacatttccgtgtcgcccttа
ttcccttttttgcggcattttgccttcctgtttttgctcacccagaaacgctggtgaaagtaaaagatgctgaagatcagttgggtgcacgagtgg
gttacatcgaactggatctcaacagcggtaagatccttgagagttttcgccccgaagaacgttttccaatgatgagcacttttaaagttctgctat
gtggcgcggtattatcccgtattgacgccgggcaagagcaactcggtcgccgcatacactattctcagaatgacttggttgagtactcacca
gtcacagaaaagcatcttacggatggcatgacagtaagagaattatgcagtgctgccataaccatgagtgataacactgcggccaacttactt
ctgacaacgatcggaggaccgaaggagctaaccgcttttttgcacaacatgggggatcatgtaactcgccttgatcgttgggaaccggagc
tgaatgaagccataccaaacgacgagcgtgacaccacgatgcctgtagcaatggcaacaacgttgcgcaaactattaactggcgaactact
tactctagcttcccggcaacaattaatagactggatggaggcggataaagttgcaggaccacttctgcgctcggcccttccggctggctggtt
tattgctgataaatctggagccggtgagcgtgggtctcgcggtatcattgcagcactggggccagatggtaagccctcccgtatcgtagttat
ctacacgacggggagtcaggcaactatggatgaacgaaatagacagatcgctgagataggtgcctcactgattaagcattggtaactgtca
gaccaagtttactcatatatactttagattgatttaaaacttcatttttaatttaaaaggatctaggtgaagatcctttttgataatctcatgaccaaaa
tcccttaacgtgagttttcgttccactgagcgtcagaccccgtagaaaagatcaaaggatcttcttgagatcctttttttctgcgcgtaatctgctg
cttgcaaacaaaaaaaccaccgctaccagcggtggtttgtttgccggatcaagagctaccaactctttttccgaaggtaactggcttcagcag
agcgcagataccaaatactgttcttctagtgtagccgtagttaggccaccacttcaagaactctgtagcaccgcctacatacctcgctctgcta
atcctgttaccagtggctgctgccagtggcgataagtcgtgtcttaccgggttggactcaagacgatagttaccggataaggcgcagcggtc
gggctgaacggggggttcgtgcacacagcccagcttggagcgaacgacctacaccgaactgagatacctacagcgtgagctatgagaaa
gcgccacgcttcccgaagggagaaaggcggacaggtatccggtaagcggcagggtcggaacaggagagcgcacgagggagcttcca
gggggaaacgcctggtatctttatagtcctgtcgggtttcgccacctctgacttgagcgtcgatttttgtgatgctcgtcaggggggcggagc
ctatggaaaaacgccagcaacgcggcctttttacggttcctggccttttgctggccttttgctcacatgttctttcctgcgttatcccctgattctgt
ggataaccgtattaccgcctttgagtgagctgataccgctcgccgcagc

Plasmid map:

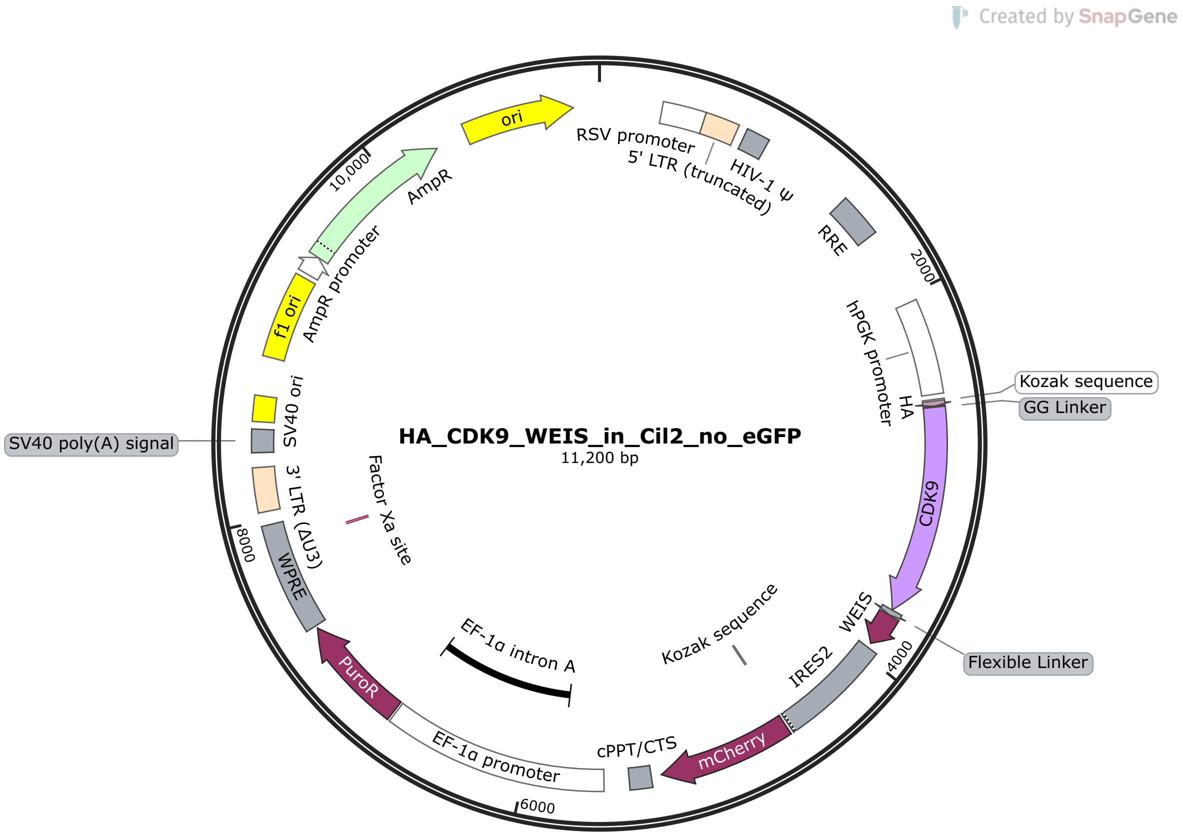

Plasmid: HA-HPRT1-WEIS

Backbone: Cil2_no_eGFP (A and B)

Insert sequence cloned into Cil2_no_eGFP:

Ccgtcgactccggaatagccaccatgtatccctatgacgtcccggactatgctggcggtgctacccgctcccccggagtggtcatctca gacgatgaaccgggttacgatctcgatctgttttgtatccctaatcattatgcagaagatcttgaacgcgtgtttattcctcacgggctgatcatg gaccggacagaacggctcgccagagacgtgatgaaggagatgggcggacaccatattgtcgccctttgcgtcctgaaaggcggatataa

gttcttcgccgaccttttggactacatcaaggcactgaatcgtaacagtgatcggagcatcccaatgactgtggattttatccgattaaagtcat
attgtaacgatcaatccactggggacattaaggtgataggcggggatgacctcagcactcttacaggcaagaatgtgctgatagtggaaga
cattattgacacgggtaagacgatgcaaacgttactcagcttggttcgacaatacaaccccaagatggtgaaagtagcttcactcttagttaag
cggaccccaaggtcagtcggttataagcctgacttcgtcgggtttgaaattcctgacaagtttgtcgtcgggtatgccttggattataatgaata
tttccgggacctcaatcacgtctgcgttataagcgaaacaggaaaggctaaatacaaagcaggtggtggtggcagtggcggaggaggttc
ctttaacgttcttatggtccataagcgcagtcataccggcgaacgtcccttctggtgcgagatctgcggggcatcctttactcagaaaggcaat
ctcctcaggcatatcaagttacatacaggtgagaagccttttaaatgtcatctgtgcaattacgcatgccaacgtcgagacgctctctaaatgc
atgagtaactgaggatccgcccctc

Full sequence:

cgaacgaccgagcgcagcgagtcagtgagcgaggaagcggaagagcgcccaatacgcaaaccgcctctccccgcgcgttggccga
ttcattaatgcagctggcacgacaggtttcccgactggaaagcgggcagtgagcgcaacgcaattaatgtgagttagctcactcattaggca
ccccaggctttacactttatgcttccggctcgtatgttgtgtggaattgtgagcggataacaatttcacacaggaaacagctatgaccatgatta
cgccaagcgcgcaattaaccctcactaaagggaacaaaagctggagctgcaagcttaatgtagtcttatgcaatactcttgtagtcttgcaac
atggtaacgatgagttagcaacatgccttacaaggagagaaaaagcaccgtgcatgccgattggtggaagtaaggtggtacgatcgtgcct
tattaggaaggcaacagacgggtctgacatggattggacgaaccactgaattgccgcattgcagagatattgtatttaagtgcctagctcgat
acataaacgggtctctctggttagaccagatctgagcctgggagctctctggctaactagggaacccactgcttaagcctcaataaagcttgc
cttgagtgcttcaagtagtgtgtgcccgtctgttgtgtgactctggtaactagagatccctcagacccttttagtcagtgtggaaaatctctagca
gtggcgcccgaacagggacttgaaagcgaaagggaaaccagaggagctctctcgacgcaggactcggcttgctgaagcgcgcacggc
aagaggcgaggggcggcgactggtgagtacgccaaaaattttgactagcggaggctagaaggagagagatgggtgcgagagcgtcagt
attaagcgggggagaattagatcgcgatgggaaaaaattcggttaaggccagggggaaagaaaaaatataaattaaaacatatagtatggg
caagcagggagctagaacgattcgcagttaatcctggcctgttagaaacatcagaaggctgtagacaaatactgggacagctacaaccatc

ccttcagacaggatcagaagaacttagatcattatataatacagtagcaaccctctattgtgtgcatcaaaggatagagataaaagacaccaa

ggaagctttagacaagatagaggaagagcaaaacaaaagtaagaccaccgcacagcaagcggccgctgatcttcagacctggaggagg

agatatgagggacaattggagaagtgaattatataaatataaagtagtaaaaattgaaccattaggagtagcacccaccaaggcaaagaga

agagtggtgcagagagaaaaaagagcagtgggaataggagctttgttccttgggttcttgggagcagcaggaagcactatgggcgcagc

gtcaatgacgctgacggtacaggccagacaattattgtctggtatagtgcagcagcagaacaatttgctgagggctattgaggcgcaacag

catctgttgcaactcacagtctggggcatcaagcagctccaggcaagaatcctggctgtggaaagatacctaaaggatcaacagctcctgg

ggatttggggttgctctggaaaactcatttgcaccactgctgtgccttggaatgctagttggagtaataaatctctggaacagatttggaatcac

acgacctggatggagtgggacagagaaattaacaattacacaagcttaatacactccttaattgaagaatcgcaaaaccagcaagaaaaga

atgaacaagaattattggaattagataaatgggcaagtttgtggaattggtttaacataacaaattggctgtggtatataaaattattcataatgat

agtaggaggcttggtaggtttaagaatagtttttgctgtactttctatagtgaatagagttaggcagggatattcaccattatcgtttcagacccac

ctcccaaccccgaggggacccttgcgccttttccaaggcagccctgggtttgcgcagggacgcggctgctctgggcgtggttccgggaaa

cgcagcggcgccgaccctgggtctcgcacattcttcacgtccgttcgcagcgtcacccggatcttcgccgctacccttgtgggccccccgg

cgacgcttcctgctccgcccctaagtcgggaaggttccttgcggttcgcggcgtgccggacgtgacaaacggaagccgcacgactcacta

gtaccctcgcagacggacagcgccagggagcaatggcagcgcgccgaccgcgatgggctgtggccaatagcggctgctcagcagggc

gcgccgagagcagcggccgggaaggggcggtgcgggaggcggggtgtggggcggtagtgtgggccctgttcctgcccgcgcggtgt

tccgcattctgcaagcctccggagcgcacgtcggcagtcggctccctcgttgaccgaatcaccgacctctctccccagggatcgataccgt

cgactccggaatagccaccatgtatccctatgacgtcccggactatgctggcggtgctacccgctcccccggagtggtcatctcagacgat

gaaccgggttacgatctcgatctgttttgtatccctaatcattatgcagaagatcttgaacgcgtgtttattcctcacgggctgatcatggaccgg

acagaacggctcgccagagacgtgatgaaggagatgggcggacaccatattgtcgccctttgcgtcctgaaaggcggatataagttcttcg

ccgaccttttggactacatcaaggcactgaatcgtaacagtgatcggagcatcccaatgactgtggattttatccgattaaagtcatattgtaac

gatcaatccactggggacattaaggtgataggcggggatgacctcagcactcttacaggcaagaatgtgctgatagtggaagacattattga

cacgggtaagacgatgcaaacgttactcagcttggttcgacaatacaaccccaagatggtgaaagtagcttcactcttagttaagcggaccc

caaggtcagtcggttataagcctgacttcgtcgggtttgaaattcctgacaagtttgtcgtcgggtatgccttggattataatgaatatttccggg

acctcaatcacgtctgcgttataagcgaaacaggaaaggctaaatacaaagcaggtggtggtggcagtggcggaggaggttcctttaacgt

tcttatggtccataagcgcagtcataccggcgaacgtcccttctggtgcgagatctgcggggcatcctttactcagaaaggcaatctcctcag

gcatatcaagttacatacaggtgagaagccttttaaatgtcatctgtgcaattacgcatgccaacgtcgagacgctctctaaatgcatgagtaa

ctgaggatccgcccctctccctccccccccctaacgttactggccgaagccgcttggaataaggccggtgtgcgtttgtctatatgttattttc

caccatattgccgtcttttggcaatgtgagggcccggaaacctggccctgtcttcttgacgagcattcctaggggtctttcccctctcgccaaa

ggaatgcaaggtctgttgaatgtcgtgaaggaagcagttcctctggaagcttcttgaagacaaacaacgtctgtagcgaccctttgcaggca

gcggaaccccccacctggcgacaggtgcctctgcggccaaaagccacgtgtataagatacacctgcaaaggcggcacaaccccagtgc

cacgttgtgagttggatagttgtggaaagagtcaaatggctctcctcaagcgtattcaacaaggggctgaaggatgcccagaaggtacccc

attgtatgggatctgatctggggcctcggtacacatgctttacatgtgtttagtcgaggttaaaaaaacgtctaggccccccgaaccacgggg

acgtggttttcctttgaaaaacacgatgataatatggccacaaccctggaattcgccaccatggtgagcaagggcgaggaggataacatgg

ccatcatcaaggagttcatgcgcttcaaggtgcacatggagggctccgtgaacggccacgagttcgagatcgagggcgagggcgaggg

ccgcccctacgagggcacccagaccgccaagctgaaggtgaccaagggtggccccctgcccttcgcctgggacatcctgtcccctcagtt

catgtacggctccaaggcctacgtgaagcaccccgccgacatccccgactacttgaagctgtccttccccgagggcttcaagtgggagcg

cgtgatgaacttcgaggacggcggcgtggtgaccgtgacccaggactcctccctgcaggacggcgagttcatctacaaggtgaagctgc

gcggcaccaacttcccctccgacggccccgtaatgcagaagaagaccatgggctgggaggcctcctccgagcggatgtaccccgagga

cggcgccctgaagggcgagatcaagcagaggctgaagctgaaggacggcggccactacgacgctgaggtcaagaccacctacaaggc

caagaagcccgtgcagctgcccggcgcctacaacgtcaacatcaagttggacatcacctcccacaacgaggactacaccatcgtggaac

agtacgaacgcgccgagggccgccactccaccggcggcatggacgagctgtacaagtaaactagtaagcttggcgtaactagatcttgag

acaaatggcagtattcatccacaattttaaaagaaaaggggggattggggggtacagtgcaggggaaagaatagtagacataatagcaac

agacatacaaactaaagaattacaaaaacaaattacaaaaattcaaaattttcgggtttattacagggacagcagagatccactttgggctcga

gggggcccgggtgcaaagatggataaagttttaaacagagaggaatctttgcagctaatggaccttctaggtcttgaaaggagtgggaattg

gctccggtgcccgtcagtgggcagagcgcacatcgcccacagtccccgagaagttggggggaggggtcggcaattgatccggtgccta

gagaaggtggcgcggggtaaactgggaaagtgatgtcgtgtactggctccgcctttttcccgagggtgggggagaaccgtatataagtgc

agtagtcgccgtgaacgttctttttcgcaacgggtttgccgccagaacacaggtaagtgccgtgtgtggttcccgcgggcctggcctctttac

gggttatggcccttgcgtgccttgaattacttccacctggctgcagtacgtgattcttgatcccgagcttcgggttggaagtgggtgggagagt

tcgaggccttgcgcttaaggagccccttcgcctcgtgcttgagttgaggcctggcctgggcgctggggccgccgcgtgcgaatctggtgg

caccttcgcgcctgtctcgctgctttcgataagtctctagccatttaaaatttttgatgacctgctgcgacgctttttttctggcaagatagtcttgta

aatgcgggccaagatctgcacactggtatttcggtttttggggccgcgggcggcgacggggcccgtgcgtcccagcgcacatgttcggcg

aggcggggcctgcgagcgcggccaccgagaatcggacgggggtagtctcaagctggccggcctgctctggtgcctggcctcgcgccg

ccgtgtatcgccccgccctgggcggcaaggctggcccggtcggcaccagttgcgtgagcggaaagatggccgcttcccggccctgctgc

agggagctcaaaatggaggacgcggcgctcgggagagcgggcgggtgagtcacccacacaaaggaaaagggcctttccgtcctcagc

cgtcgcttcatgtgactccacggagtaccgggcgccgtccaggcacctcgattagttctcgagcttttggagtacgtcgtctttaggttgggg

ggaggggttttatgcgatggagtttccccacactgagtgggtggagactgaagttaggccagcttggcacttgatgtaattctccttggaattt

gcccttttttgagtttggatcttggttcattctcaagcctcagacagtggttcaaagtttttttcttccatttcaggtgtcgtgacgtacggccaccat

gaccgagtacaagcccacggtgcgcctcgccacccgcgacgacgtccccagggccgtacgcaccctcgccgccgcgttcgccgactac

cccgccacgcgccacaccgtcgatccggaccgccacatcgagcgggtcaccgagctgcaagaactcttcctcacgcgcgtcgggctcg

acatcggcaaggtgtgggtcgcggacgacggcgccgccgtggcggtctggaccacgccggagagcgtcgaagcgggggcggtgttc

gccgagatcggcccgcgcatggccgagttgagcggttcccggctggccgcgcagcaacagatggaaggcctcctggcgccgcaccgg

cccaaggagcccgcgtggttcctggccaccgtcggagtctcgcccgaccaccagggcaagggtctgggcagcgccgtcgtgctccccg

gagtggaggcggccgagcgcgccggggtgcccgccttcctggagacctccgcgccccgcaacctccccttctacgagcggctcggctt

caccgtcaccgccgacgtcgaggtgcccgaaggaccgcgcacctggtgcatgacccgcaagcccggtgcctgaacgcgttaagtcgac

aatcaacctctggattacaaaatttgtgaaagattgactggtattcttaactatgttgctccttttacgctatgtggatacgctgctttaatgcctttgt

atcatgctattgcttcccgtatggctttcattttctcctccttgtataaatcctggttgctgtctctttatgaggagttgtggcccgttgtcaggcaac

gtggcgtggtgtgcactgtgtttgctgacgcaacccccactggttggggcattgccaccacctgtcagctcctttccgggactttcgctttccc

cctccctattgccacggcggaactcatcgccgcctgccttgcccgctgctggacaggggctcggctgttgggcactgacaattccgtggtgt

tgtcggggaaatcatcgtcctttccttggctgctcgcctgtgttgccacctggattctgcgcgggacgtccttctgctacgtcccttcggccctc

aatccagcggaccttccttcccgcggcctgctgccggctctgcggcctcttccgcgtcttcgccttcgccctcagacgagtcggatctcccttt

gggccgcctccccgcgtcgactttaagaccaatgacttacaaggcagctgtagatcttagccactttttaaaagaaaaggggggactggaa

gggctaattcactcccaacgaagacaagatctgctttttgcttgtactgggtctctctggttagaccagatctgagcctgggagctctctggcta

actagggaacccactgcttaagcctcaataaagcttgccttgagtgcttcaagtagtgtgtgcccgtctgttgtgtgactctggtaactagagat

ccctcagacccttttagtcagtgtggaaaatctctagcagtacgtatagtagttcatgtcatcttattattcagtatttataacttgcaaagaaatga

atatcagagagtgagaggaacttgtttattgcagcttataatggttacaaataaagcaatagcatcacaaatttcacaaataaagcatttttttcac

tgcattctagttgtggtttgtccaaactcatcaatgtatcttatcatgtctggctctagctatcccgcccctaactccgcccatcccgcccctaact

ccgcccagttccgcccattctccgccccatggctgactaattttttttatttatgcagaggccgaggccgcctcggcctctgagctattccagaa

gtagtgaggaggcttttttggaggcctagggacgtacccaattcgccctatagtgagtcgtattacgcgcgctcactggccgtcgttttacaac

gtcgtgactgggaaaaccctggcgttacccaacttaatcgccttgcagcacatccccctttcgccagctggcgtaatagcgaagaggcccg

caccgatcgcccttcccaacagttgcgcagcctgaatggcgaatgggacgcgccctgtagcggcgcattaagcgcggcgggtgtggtgg

ttacgcgcagcgtgaccgctacacttgccagcgccctagcgcccgctcctttcgctttcttcccttcctttctcgccacgttcgccggctttccc

cgtcaagctctaaatcgggggctccctttagggttccgatttagtgctttacggcacctcgaccccaaaaaacttgattagggtgatggttcac

gtagtgggccatcgccctgatagacggtttttcgccctttgacgttggagtccacgttctttaatagtggactcttgttccaaactggaacaaca

ctcaaccctatctcggtctattcttttgatttataagggattttgccgatttcggcctattggttaaaaaatgagctgatttaacaaaaatttaacgcg

aattttaacaaaatattaacgcttacaatttaggtggcacttttcggggaaatgtgcgcggaacccctatttgtttatttttctaaatacattcaaata

tgtatccgctcatgagacaataaccctgataaatgcttcaataatattgaaaaaggaagagtatgagtattcaacatttccgtgtcgcccttattc

ccttttttgcggcattttgccttcctgtttttgctcacccagaaacgctggtgaaagtaaaagatgctgaagatcagttgggtgcacgagtgggtt

acatcgaactggatctcaacagcggtaagatccttgagagttttcgccccgaagaacgttttccaatgatgagcacttttaaagttctgctatgt

ggcgcggtattatcccgtattgacgccgggcaagagcaactcggtcgccgcatacactattctcagaatgacttggttgagtactcaccagtc

acagaaaagcatcttacggatggcatgacagtaagagaattatgcagtgctgccataaccatgagtgataacactgcggccaacttacttctg

acaacgatcggaggaccgaaggagctaaccgcttttttgcacaacatgggggatcatgtaactcgccttgatcgttgggaaccggagctga

atgaagccataccaaacgacgagcgtgacaccacgatgcctgtagcaatggcaacaacgttgcgcaaactattaactggcgaactacttac

tctagcttcccggcaacaattaatagactggatggaggcggataaagttgcaggaccacttctgcgctcggcccttccggctggctggtttat

tgctgataaatctggagccggtgagcgtgggtctcgcggtatcattgcagcactggggccagatggtaagccctcccgtatcgtagttatcta

cacgacggggagtcaggcaactatggatgaacgaaatagacagatcgctgagataggtgcctcactgattaagcattggtaactgtcagac

caagtttactcatatatactttagattgatttaaaacttcatttttaatttaaaaggatctaggtgaagatcctttttgataatctcatgaccaaaatccc

ttaacgtgagttttcgttccactgagcgtcagaccccgtagaaaagatcaaaggatcttcttgagatcctttttttctgcgcgtaatctgctgcttg

caaacaaaaaaaccaccgctaccagcggtggtttgtttgccggatcaagagctaccaactctttttccgaaggtaactggcttcagcagagc

gcagataccaaatactgttcttctagtgtagccgtagttaggccaccacttcaagaactctgtagcaccgcctacatacctcgctctgctaatcc

tgttaccagtggctgctgccagtggcgataagtcgtgtcttaccgggttggactcaagacgatagttaccggataaggcgcagcggtcggg

ctgaacggggggttcgtgcacacagcccagcttggagcgaacgacctacaccgaactgagatacctacagcgtgagctatgagaaagcg

ccacgcttcccgaagggagaaaggcggacaggtatccggtaagcggcagggtcggaacaggagagcgcacgagggagcttccaggg

ggaaacgcctggtatctttatagtcctgtcgggtttcgccacctctgacttgagcgtcgatttttgtgatgctcgtcaggggggcggagcctat

ggaaaaacgccagcaacgcggcctttttacggttcctggccttttgctggccttttgctcacatgttctttcctgcgttatcccctgattctgtgga

taaccgtattaccgcctttgagtgagctgataccgctcgccgcagc

Plasmid map:

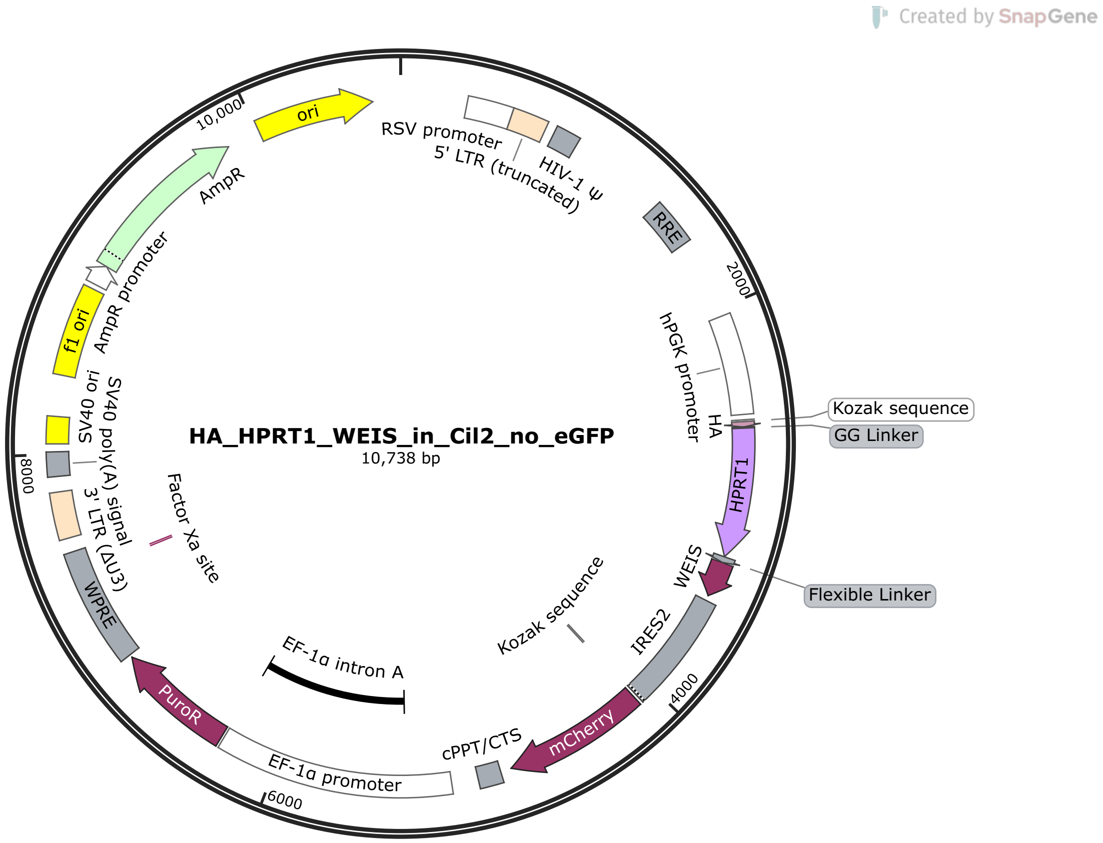
Created by SnapGene
HA_HPRT1_WEIS_in_Cil2_no_eGFP
10,738 bp
ori
RSV promoter
5' LTR (truncated)
HIV-1 ψ
RRE
2000
hPGK promoter
HA
Kozak sequence
GG Linker
HPRT1
WEIS
Flexible Linker
IRES2
4000
mCherry
cPPT/CTS
Kozak sequence
EF-1α intron A
EF-1α promoter
6000
PuroR
WPRE
3' LTR (ΔU3)
SV40 poly(A) signal
Factor Xa site
8000
SV40 ori
f1 ori
AmpR promoter
AmpR
10,000

Sequences for NGS

NGS PCR1 primers

Name	Sequence
PCR1 F	CAGGTGTCGTGAGGATCTAT TTCCG
PCR1 R	GTGCAGATGAATTTCAGGGT CAGG

NGS PCR2 P5 primers

Sample	Name	Sequence
Replicate 1	N502 index P5 0 nt stagger	AATGATACGGCGACCACCGAGATCTACACCTCTCTATACACTCTTTCCCTAC<br>ACGACGCTCTTCCGATCTAGCGAAGCCATACTGGTGAA*C
Replicate 1	N502 index P5 1 nt stagger	AATGATACGGCGACCACCGAGATCTACACCTCTCTATACACTCTTTCCCTAC<br>ACGACGCTCTTCCGATCTCAGCGAAGCCATACTGGTGAA*C
Replicate 1	N502 index P5 2 nt stagger	AATGATACGGCGACCACCGAGATCTACACCTCTCTATACACTCTTTCCCTAC<br>ACGACGCTCTTCCGATCTGCAGCGAAGCCATACTGGTGAA*C
Replicate 1	N502 index P5 3 nt stagger	AATGATACGGCGACCACCGAGATCTACACCTCTCTATACACTCTTTCCCTAC<br>ACGACGCTCTTCCGATCTAGCAGCGAAGCCATACTGGTGAA*C
Replicate 1	N502 index P5 4 nt stagger	AATGATACGGCGACCACCGAGATCTACACCTCTCTATACACTCTTTCCCTAC<br>ACGACGCTCTTCCGATCTCAACAGCGAAGCCATACTGGTGAA*C
Replicate 1	N502 index P5 6 nt stagger	AATGATACGGCGACCACCGAGATCTACACCTCTCTATACACTCTTTCCCTAC<br>ACGACGCTCTTCCGATCTTGCACCAGCGAAGCCATACTGGTGAA*C
Replicate 1	N502 index P5 7 nt stagger	AATGATACGGCGACCACCGAGATCTACACCTCTCTATACACTCTTTCCCTAC<br>ACGACGCTCTTCCGATCTACGCAACAGCGAAGCCATACTGGTGAA*C
Replicate 1	N502 index P5 8 nt stagger	AATGATACGGCGACCACCGAGATCTACACCTCTCTATACACTCTTTCCCTAC<br>ACGACGCTCTTCCGATCTGAAGACCCAGCGAAGCCATACTGGTGAA*C
Replicate 2	N505 index P5 0 nt stagger	AATGATACGGCGACCACCGAGATCTACACGTAAGGAGACACTCTTTCCCTA<br>CACGACGCTCTTCCGATCTAGCGAAGCCATACTGGTGAA*C
Replicate 2	N505 index P5 1 nt stagger	AATGATACGGCGACCACCGAGATCTACACGTAAGGAGACACTCTTTCCCTA<br>CACGACGCTCTTCCGATCTCAGCGAAGCCATACTGGTGAA*C
Replicate 2	N505 index P5 2 nt stagger	AATGATACGGCGACCACCGAGATCTACACGTAAGGAGACACTCTTTCCCTA<br>CACGACGCTCTTCCGATCTGCAGCGAAGCCATACTGGTGAA*C
Replicate 2	N505 index P5 3 nt stagger	AATGATACGGCGACCACCGAGATCTACACGTAAGGAGACACTCTTTCCCTA<br>CACGACGCTCTTCCGATCTAGCAGCGAAGCCATACTGGTGAA*C
Replicate 2	N505 index P5 4 nt stagger	AATGATACGGCGACCACCGAGATCTACACGTAAGGAGACACTCTTTCCCTA<br>CACGACGCTCTTCCGATCTCAACAGCGAAGCCATACTGGTGAA*C
Replicate 2	N505 index P5 6 nt stagger	AATGATACGGCGACCACCGAGATCTACACGTAAGGAGACACTCTTTCCCTA<br>CACGACGCTCTTCCGATCTTGCACCAGCGAAGCCATACTGGTGAA*C
Replicate 2	N505 index P5 7 nt stagger	AATGATACGGCGACCACCGAGATCTACACGTAAGGAGACACTCTTTCCCTA<br>CACGACGCTCTTCCGATCTACGCAACAGCGAAGCCATACTGGTGAA*C
Replicate 2	N505 index P5 8 nt stagger	AATGATACGGCGACCACCGAGATCTACACGTAAGGAGACACTCTTTCCCTA<br>CACGACGCTCTTCCGATCTGAAGACCCAGCGAAGCCATACTGGTGAA*C
Replicate 3	N506 index P5 0 nt stagger	AATGATACGGCGACCACCGAGATCTACACACTGCATAACACTCTTTCCCTAC<br>ACGACGCTCTTCCGATCTAGCGAAGCCATACTGGTGAA*C
Replicate 3	N506 index P5 1 nt stagger	AATGATACGGCGACCACCGAGATCTACACACTGCATAACACTCTTTCCCTAC<br>ACGACGCTCTTCCGATCTCAGCGAAGCCATACTGGTGAA*C
Replicate 3	N506 index P5 2 nt stagger	AATGATACGGCGACCACCGAGATCTACACACTGCATAACACTCTTTCCCTAC<br>ACGACGCTCTTCCGATCTGCAGCGAAGCCATACTGGTGAA*C
Replicate 3	N506 index P5 3 nt stagger	AATGATACGGCGACCACCGAGATCTACACACTGCATAACACTCTTTCCCTAC<br>ACGACGCTCTTCCGATCTAGCAGCGAAGCCATACTGGTGAA*C
Replicate 3	N506 index P5 4 nt stagger	AATGATACGGCGACCACCGAGATCTACACACTGCATAACACTCTTTCCCTAC<br>ACGACGCTCTTCCGATCTCAACAGCGAAGCCATACTGGTGAA*C
Replicate 3	N506 index P5 6 nt stagger	AATGATACGGCGACCACCGAGATCTACACACTGCATAACACTCTTTCCCTAC<br>ACGACGCTCTTCCGATCTTGCACCAGCGAAGCCATACTGGTGAA*C
Replicate 3	N506 index P5 7 nt stagger	AATGATACGGCGACCACCGAGATCTACACACTGCATAACACTCTTTCCCTAC<br>ACGACGCTCTTCCGATCTACGCAACAGCGAAGCCATACTGGTGAA*C
Replicate 3	N506 index P5 8 nt stagger	AATGATACGGCGACCACCGAGATCTACACACTGCATAACACTCTTTCCCTAC<br>ACGACGCTCTTCCGATCTGAAGACCCAGCGAAGCCATACTGGTGAA*C

P5 flowcell attachment sequence
Illumina sequencing primer
Vector primer binding sequence
Barcode region
Stagger region
* = Phosphorothioate bond

NGS PCR2 P7 primers

Compound	Name	Sequence
DMSO -ve	N703 index P7.3	CAAGCAGAAGACGGCATACGAGATTTCTGCCTGTGACTGGAGTTCAGACGTG TGCTCTTCCGATCTGTGACACTTAAAAGGTTTTTCCCCT*G
Thal -ve	N704 index P7.3	CAAGCAGAAGACGGCATACGAGATGCTCAGGAGTGACTGGAGTTCAGACGTG TGCTCTTCCGATCTGTGACACTTAAAAGGTTTTTCCCCT*G
19 -ve	N705 index P7.3	CAAGCAGAAGACGGCATACGAGATAGGAGTCCGTGACTGGAGTTCAGACGTG TGCTCTTCCGATCTGTGACACTTAAAAGGTTTTTCCCCT*G
23 -ve	N706 index P7.3	CAAGCAGAAGACGGCATACGAGATCATGCCTAGTGACTGGAGTTCAGACGTG TGCTCTTCCGATCTGTGACACTTAAAAGGTTTTTCCCCT*G
Len -ve	N707 index P7.3	CAAGCAGAAGACGGCATACGAGATGTAGAGAGGTGACTGGAGTTCAGACGTG TGCTCTTCCGATCTGTGACACTTAAAAGGTTTTTCCCCT*G
33 -ve	N710 index P7.3	CAAGCAGAAGACGGCATACGAGATCAGCCTCGGTGACTGGAGTTCAGACGTG TGCTCTTCCGATCTGTGACACTTAAAAGGTTTTTCCCCT*G
22 -ve	N711 index P7.3	CAAGCAGAAGACGGCATACGAGATTGCCTCTTGTGACTGGAGTTCAGACGTG TGCTCTTCCGATCTGTGACACTTAAAAGGTTTTTCCCCT*G
DMSO +ve	N714 index P7.3	CAAGCAGAAGACGGCATACGAGATTCATGAGCGTGACTGGAGTTCAGACGTG TGCTCTTCCGATCTGTGACACTTAAAAGGTTTTTCCCCT*G
Thal +ve	N715 index P7.3	CAAGCAGAAGACGGCATACGAGATCCTGAGATGTGACTGGAGTTCAGACGTG TGCTCTTCCGATCTGTGACACTTAAAAGGTTTTTCCCCT*G
19 +ve	N716 index P7.3	CAAGCAGAAGACGGCATACGAGATTAGCGAGTGTGACTGGAGTTCAGACGTG TGCTCTTCCGATCTGTGACACTTAAAAGGTTTTTCCCCT*G
23 +ve	N718 index P7.3	CAAGCAGAAGACGGCATACGAGATGTAGCTCCGTGACTGGAGTTCAGACGTG TGCTCTTCCGATCTGTGACACTTAAAAGGTTTTTCCCCT*G
Len +ve	N719 index P7.3	CAAGCAGAAGACGGCATACGAGATTACTACGCGTGACTGGAGTTCAGACGTG TGCTCTTCCGATCTGTGACACTTAAAAGGTTTTTCCCCT*G
33 +ve	N720 index P7.3	CAAGCAGAAGACGGCATACGAGATAGGCTCCGGTGACTGGAGTTCAGACGTG TGCTCTTCCGATCTGTGACACTTAAAAGGTTTTTCCCCT*G
22 +ve	N721 index P7.3	CAAGCAGAAGACGGCATACGAGATGCAGCGTAGTGACTGGAGTTCAGACGTG TGCTCTTCCGATCTGTGACACTTAAAAGGTTTTTCCCCT*G

P7 flowcell attachment sequence
Illumina sequencing primer
Vector primer binding sequence
Barcode region
* = Phosphorothioate bond

sgRNA

sgRNA	Sequence
TRIM28 sgRNA 1	AUCCCUGCUUCUCGAAGUGG
TRIM28 sgRNA 2	GUGCUUCUCCAAAGACAUCG
TRIM28 sgRNA 3	CGACGCCCAGGAUGCGAACC

PCR primers for amplifying sgRNA target site

Primer	Sequence
TRIM28 F	CGAAGTGATCGGTGCCAC
TRIM28 R	GCATTATCCTCACAGCTAGTGC

Computational Methods

Docking was carried out using the crystal structure DDB1-CRBN-pomalidomide complex bound to IKZF1(ZF2) from the Protein Data Bank (PDB ID: 6H0F) as a template [52].

Protein energetics and mutation evaluation were carried out using the PositionScan function in FoldX 5. The 3D coordinate files generated for the mutant proteins were then used for subsequent docking.

Docking was carried out using GOLD version 2020.1 from the Cambridge Crystallographic Data Centre. The position of the original pomalidomide ligand in the template crystal structure was used to define the binding pocket for new ligands using GOLD's 'cavity' functionality. The imide ring region of the original pomalidomide ligand was used to specify the position onto which the imide region of screened ligands should be superimposed using GOLD's 'scaffold' functionality. All parameters were kept as default during docking screens, except constraint weight, which was increased from the default value of 5 to 10.

Mutational analysis for use in the lentiviral library screen was performed using the PositionScan functionality in FoldX and the PositionMutation functionality in Rosetta. The same crystal structure (PDB ID: 6H0F) was used with the pomalidomide ligand removed [52].

Safety Comment

No unexpected or unusually high safety hazards were encountered.

NMR Spectra

2-(2,6-Dioxopiperidin-3-yl)isoindoline-1,3-dione (**1**)

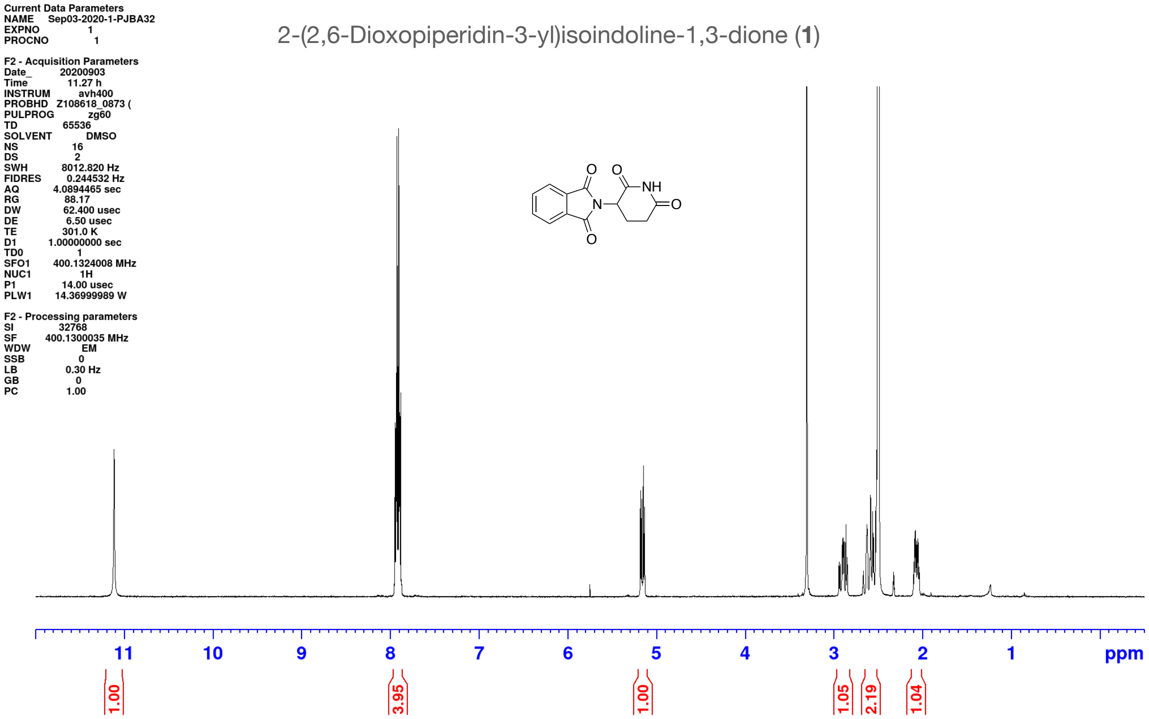

Current Data Parameters
NAME Nov26-2019-1-PJBA03-crude-prod
EXPNO 1
PROCNO 1

F2 - Acquisition Parameters
Date_ 20191126
Time 18.56 h
INSTRUM avh400
PROBHD Z108618_0873 (
PULPROG zg60
TD 65536
SOLVENT DMSO
NS 16
DS 2
SWH 8012.820 Hz
FIDRES 0.244532 Hz
AQ 4.0894465 sec
RG 88.17
DW 62.400 usec
DE 6.50 usec
TE 300.2 K
D1 1.00000000 sec
TD0 1
SFO1 400.1324008 MHz
NUC1 1H
P1 14.00 usec
PLW1 14.36999989 W

F2 - Processing parameters
SI 32768
SF 400.1300030 MHz
WDW EM
SSB 0
LB 0.30 Hz
GB 0
PC 1.00

4-Amino-2-(2,6-dioxopiperidin-3-yl)isoindoline-1,3-dione (**2**)

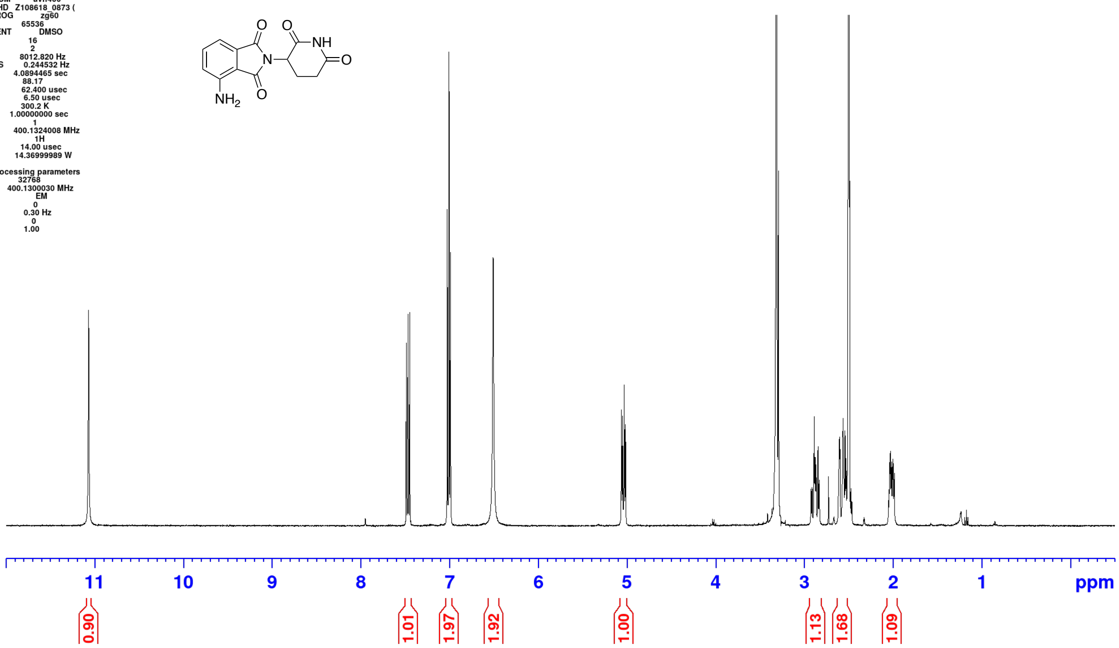

Current Data Parameters
NAME Sep09-2022-3-PJBA48_clean
EXPNO 1
PROCNO 1

F2 - Acquisition Parameters
Date_ 20220909
Time 18.56 h
INSTRUM avh400
PROBHD Z116098_0219 (
PULPROG zg60
TD 65536
SOLVENT DMSO
NS 16
DS 2
SWH 8012.820 Hz
FIDRES 0.244532 Hz
AQ 4.0894465 sec
RG 88.17
DW 62.400 usec
DE 6.50 usec
TE 296.5 K
D1 1.00000000 sec
TD0 1
SFO1 400.1324008 MHz
NUC1 1H
P1 10.00 usec
PLW1 16.00000000 W

F2 - Processing parameters
SI 32768
SF 400.1300033 MHz
WDW EM
SSB 0
LB 0.30 Hz
GB 0
PC 1.00

5-Amino-2-(2,6-dioxopiperidin-3-yl)isoindoline-1,3-dione (**3**)

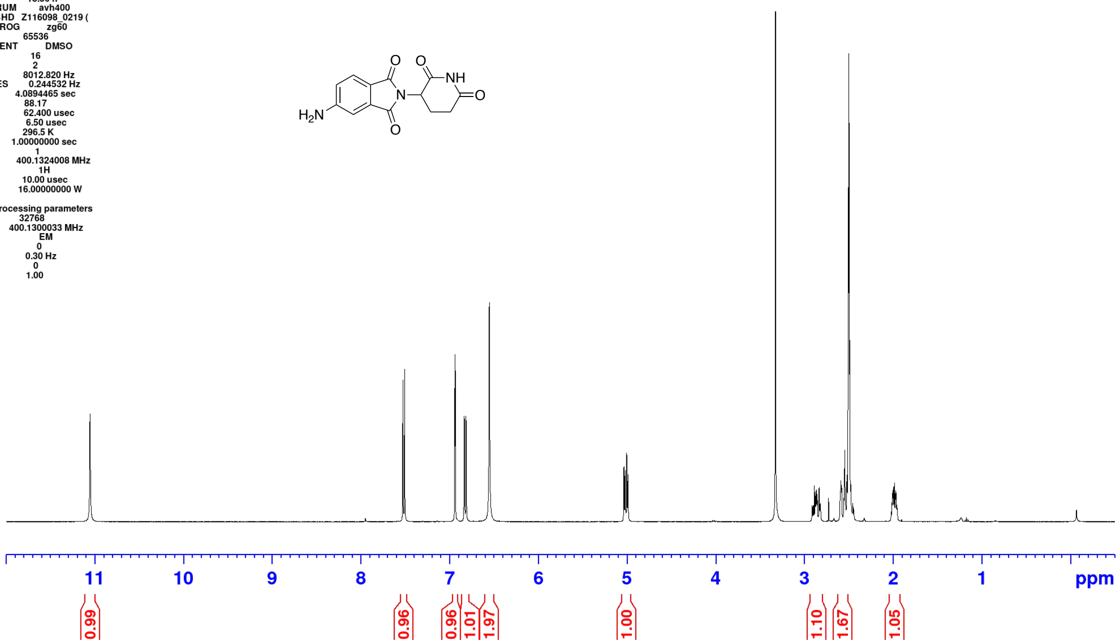

Current Data Parameters
NAME Feb18-2020-6-PJBA17-postextract1
EXPNO 1
PROCNO 1

F2 - Acquisition Parameters
Date_ 20200218
Time 19.42 h
INSTRUM avh400
PROBHD Z108618_0873 (
PULPROG zg60
TD 65536
SOLVENT DMSO
NS 16
DS 2
SWH 8012.820 Hz
FIDRES 0.244532 Hz
AQ 4.0894465 sec
RG 88.17
DW 62.400 usec
DE 6.50 usec
TE 299.2 K
D1 1.00000000 sec
TD0 1
SFO1 400.1324008 MHz
NUC1 1H
P1 14.00 usec
PLW1 14.36999989 W

F2 - Processing parameters
SI 32768
SF 400.1300034 MHz
WDW EM
SSB 0
LB 0.30 Hz
GB 0
PC 1.00

2-(2,6-Dioxopiperidin-3-yl)-4-hydroxyisoindoline-1,3-dione (**4**)

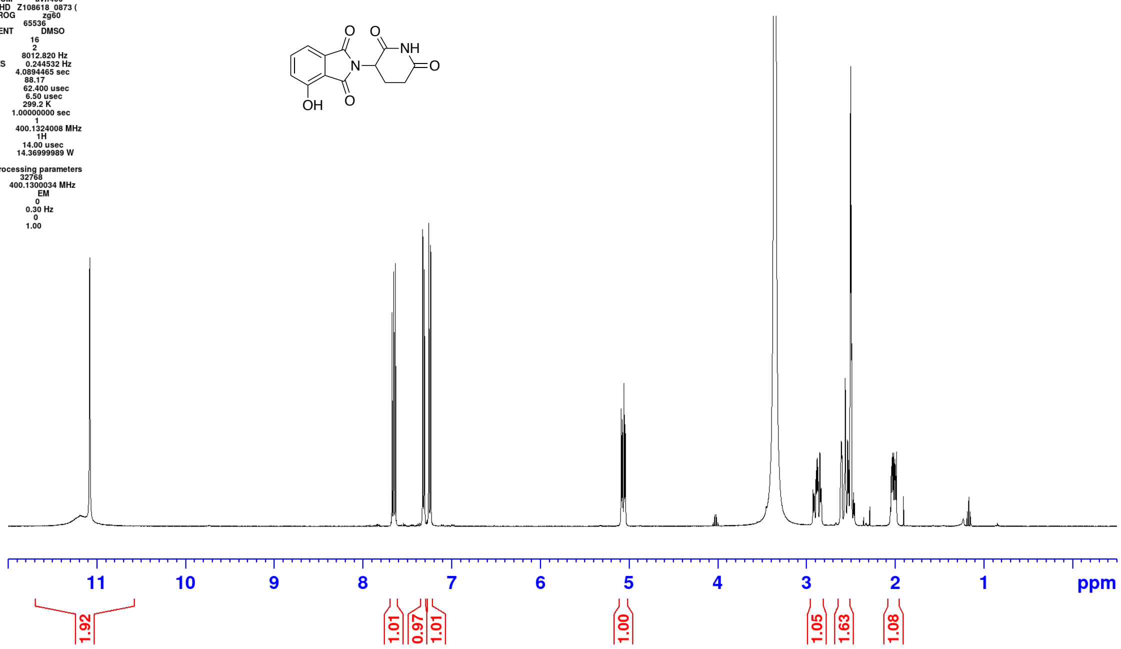

Current Data Parameters
NAME Sep20-2022-1-PJBA75
EXPNO 1
PROCNO 1

F2 - Acquisition Parameters
Date_ 20220920
Time 11.57 h
INSTRUM avg400
PROBHD Z8400_0179 (PH
PULPROG zg60
TD 65536
SOLVENT DMSO
NS 16
DS 2
SWH 8012.820 Hz
FIDRES 0.244532 Hz
AQ 4.0894465 sec
RG 206.87
DW 62.400 usec
DE 6.50 usec
TE 293.5 K
D1 1.00000000 sec
TD0 1
SFO1 400.2024012 MHz
NUC1 1H
P1 11.00 usec
PLW1 14.00000000 W

F2 - Processing parameters
SI 32768
SF 400.2000036 MHz
WDW EM
SSB 0
LB 0.30 Hz
GB 0
PC 1.00

2-(2,6-Dioxopiperidin-3-yl)-5-hydroxyisoindoline-1,3-dione (**5**)

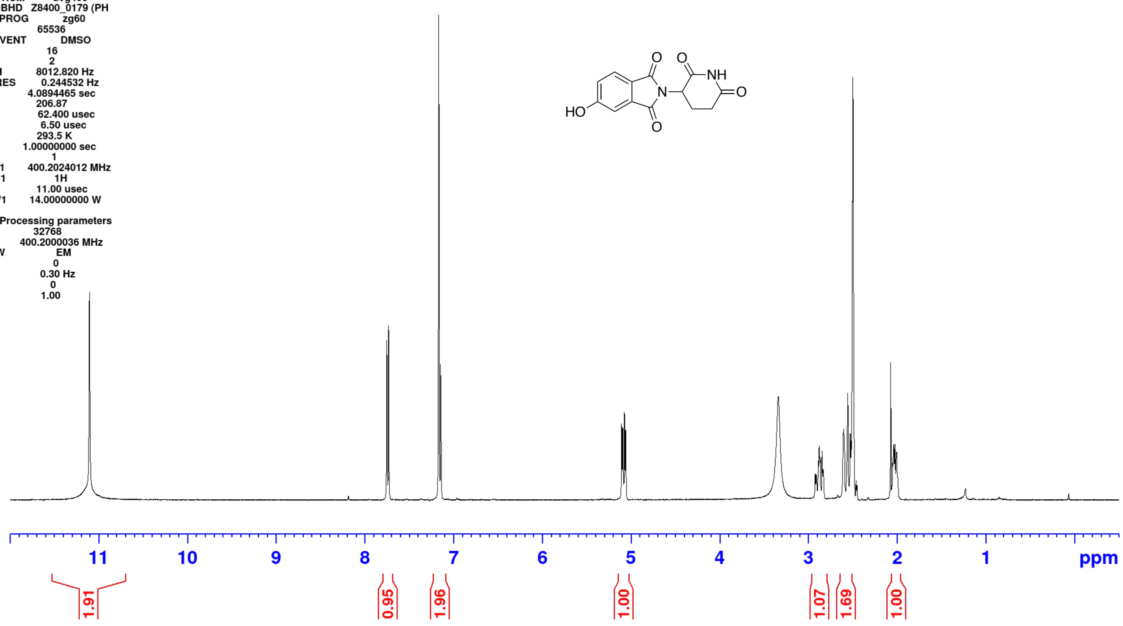

Current Data Parameters
NAME Sep09-2022-10-PJBB05
EXPNO 1
PROCNO 1

F2 - Acquisition Parameters
Date_ 20220909
Time 19.27 h
INSTRUM avh400
PROBHD Z116098_0219 (
PULPROG zg60
TD 65536
SOLVENT DMSO
NS 16
DS 2
SWH 8012.820 Hz
FIDRES 0.244532 Hz
AQ 4.0894465 sec
RG 88.17
DW 62.400 usec
DE 6.50 usec
TE 296.3 K
D1 1.00000000 sec
TD0 1
SFO1 400.1324008 MHz
NUC1 1H
P1 10.00 usec
PLW1 16.00000000 W

F2 - Processing parameters
SI 32768
SF 400.1300032 MHz
WDW EM
SSB 0
LB 0.30 Hz
GB 0
PC 1.00

2-(2,6-Dioxopiperidin-3-yl)-4-methoxyisoindoline-1,3-dione (**6**)

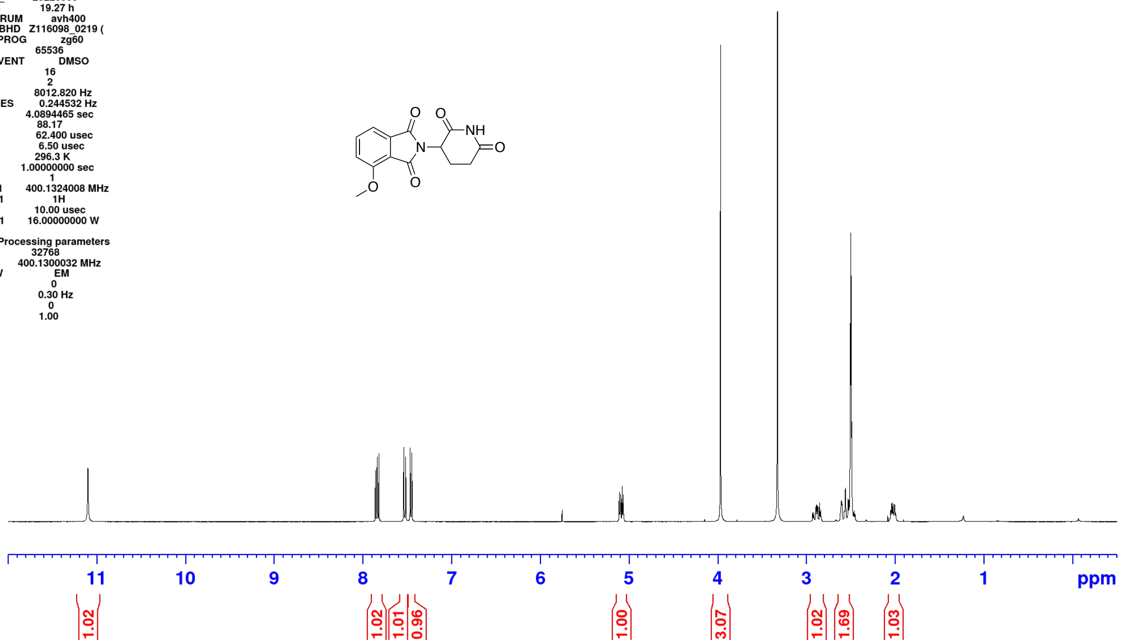

2-(2,6-Dioxopiperidin-3-yl)-5-methoxyisoindoline-1,3-dione (**7**)

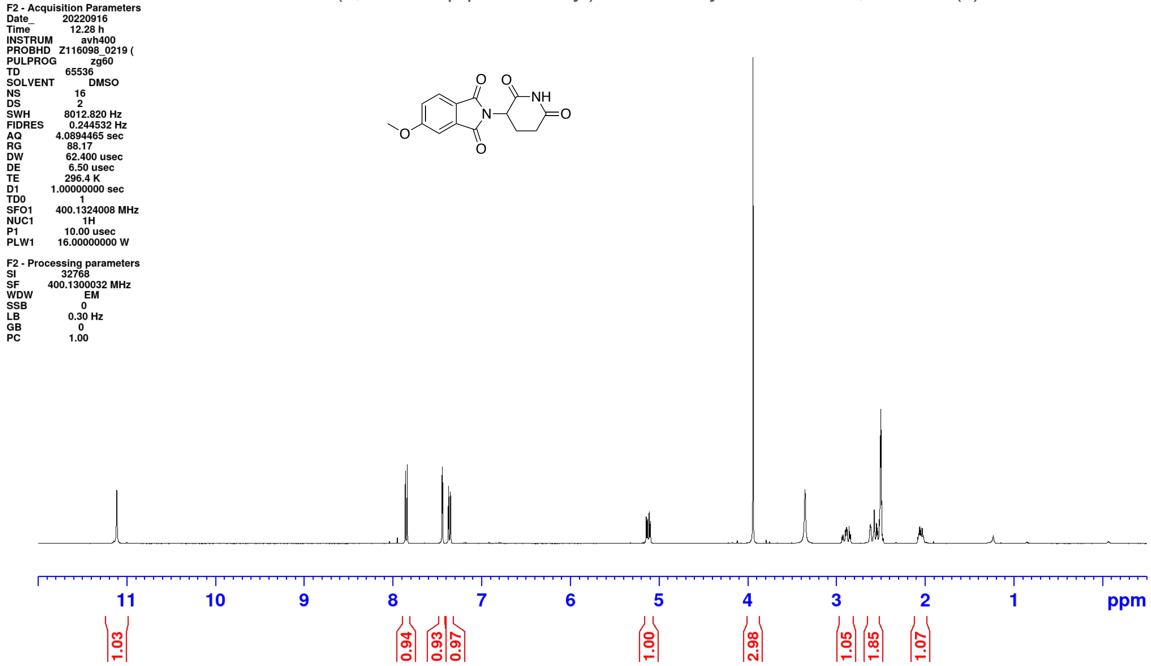

2-(2,6-Dioxopiperidin-3-yl)-5-methoxyisoindoline-1,3-dione (**7**)

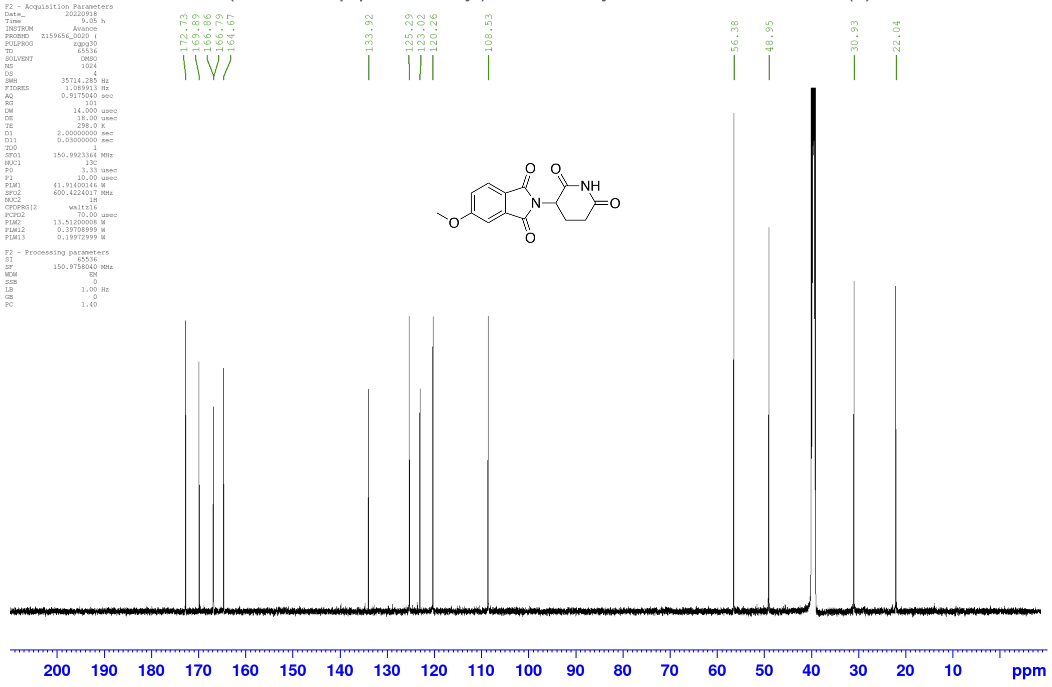

2-(2,6-Dioxopiperidin-3-yl)-4-fluoroisoindoline-1,3-dione (**8**)

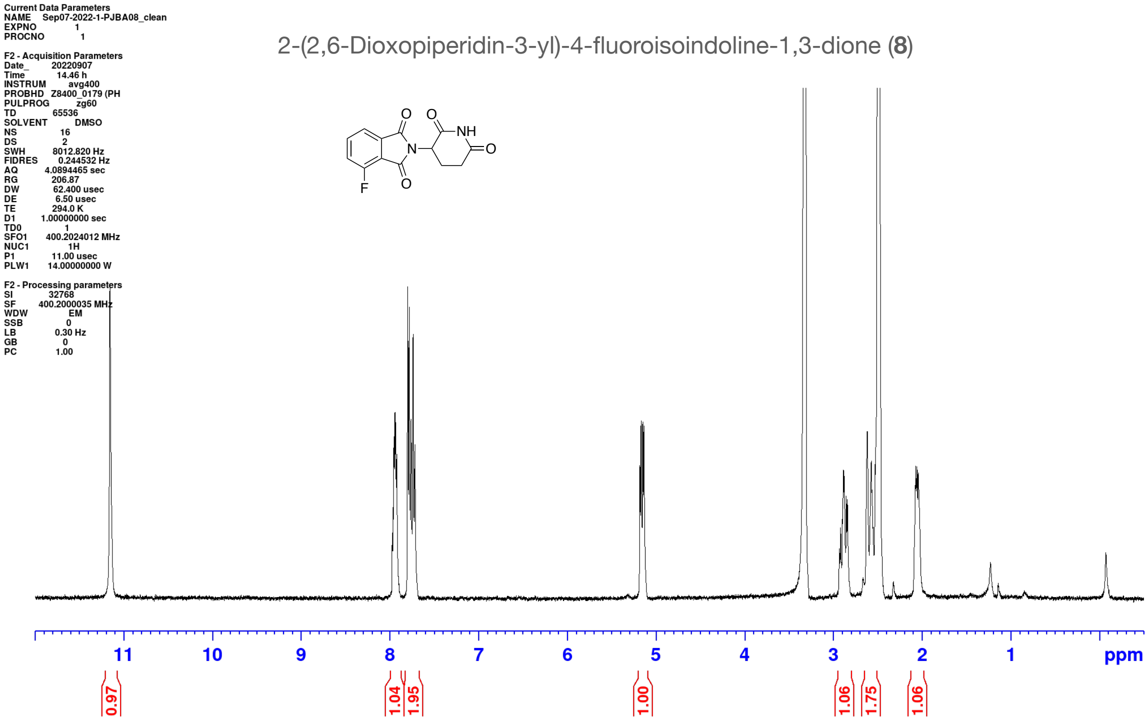

2-(2,6-Dioxopiperidin-3-yl)-5-fluoroisoindoline-1,3-dione (**9**)

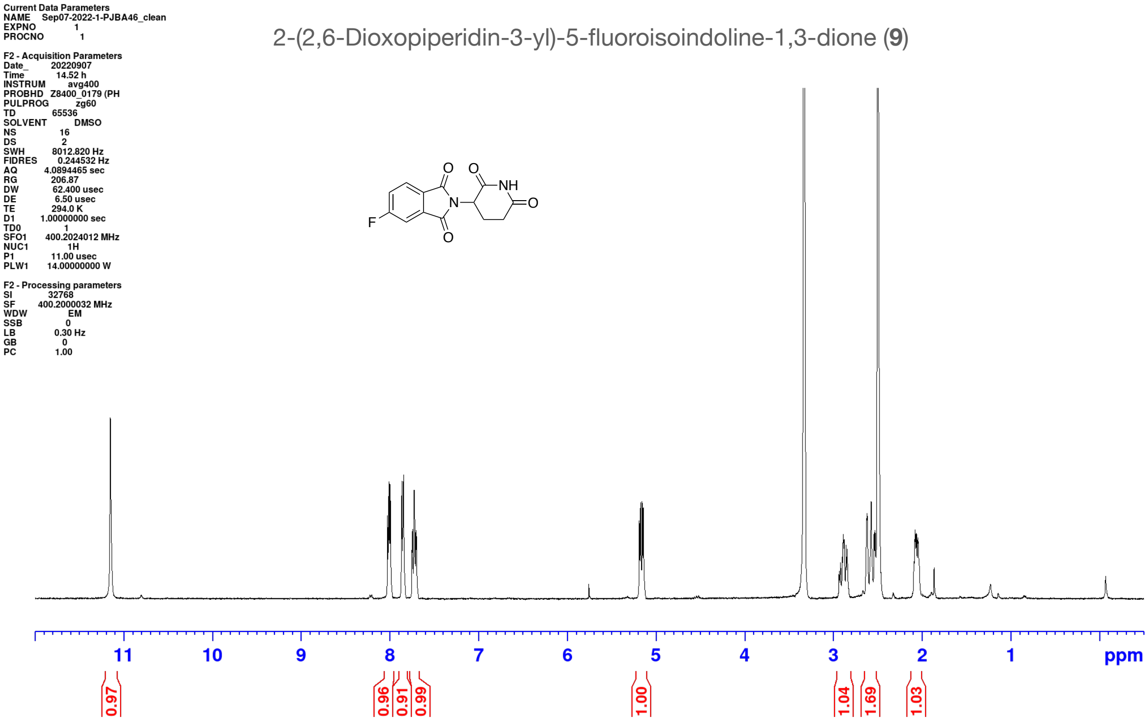

4-Chloro-2-(2,6-dioxopiperidin-3-yl)isoindoline-1,3-dione (**10**)

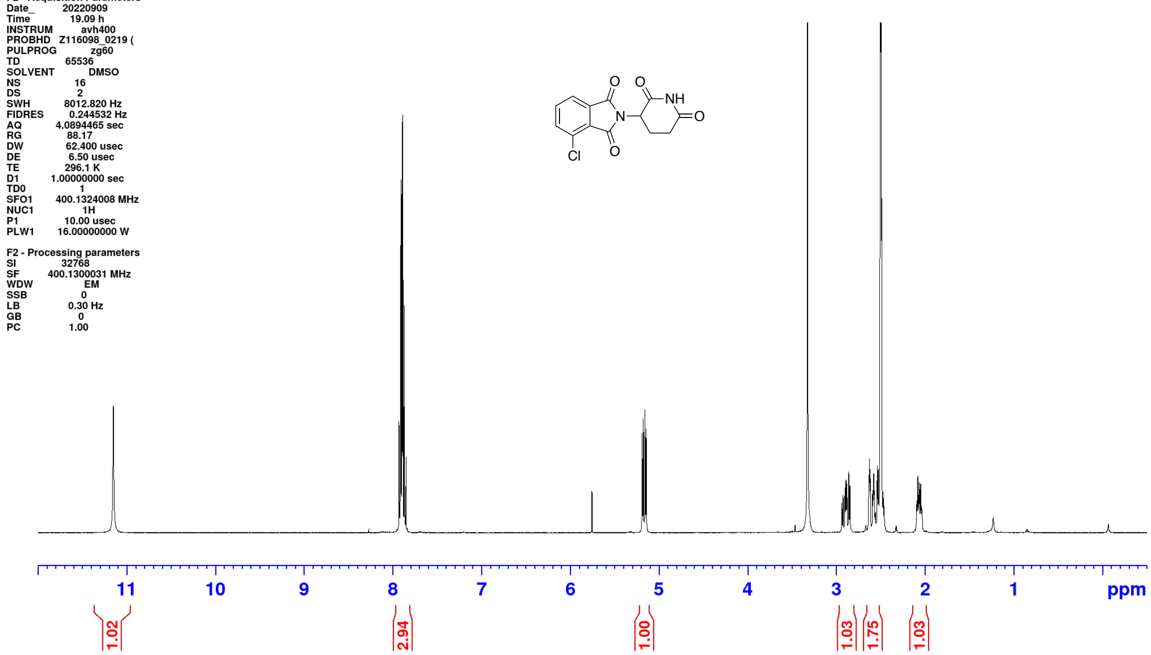

5-Chloro-2-(2,6-dioxopiperidin-3-yl)isoindoline-1,3-dione (**11**)

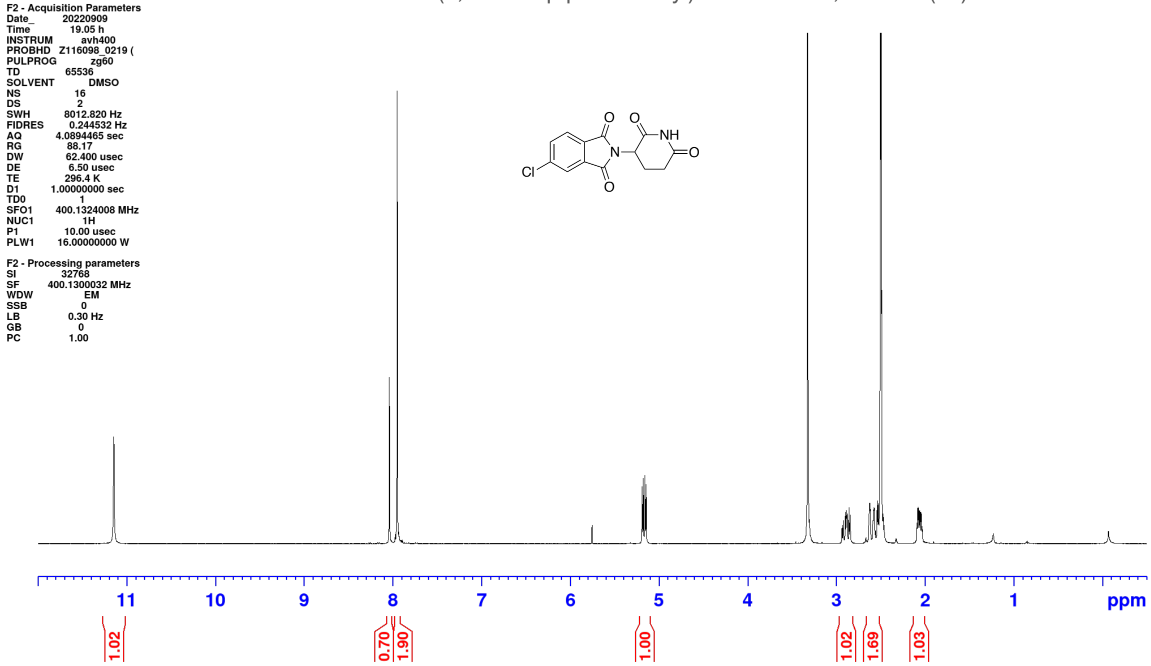

Current Data Parameters
NAME Sep09-2022-7-PJBA93
EXPNO 1
PROCNO 1

F2 - Acquisition Parameters
Date_ 20220909
Time 19.13 h
INSTRUM avh400
PROBHD Z116098_0219 (
PULPROG zg60
TD 65536
SOLVENT DMSO
NS 16
DS 2
SWH 8012.820 Hz
FIDRES 0.244532 Hz
AQ 4.0894465 sec
RG 88.17
DW 62.400 usec
DE 6.50 usec
TE 296.0 K
D1 1.00000000 sec
TD0 1
SFO1 400.1324008 MHz
NUC1 1H
P1 10.00 usec
PLW1 16.00000000 W

F2 - Processing parameters
SI 32768
SF 400.1300033 MHz
WDW EM
SSB 0
LB 0.30 Hz
GB 0
PC 1.00

4-Bromo-2-(2,6-dioxopiperidin-3-yl)isoindoline-1,3-dione (**12**)

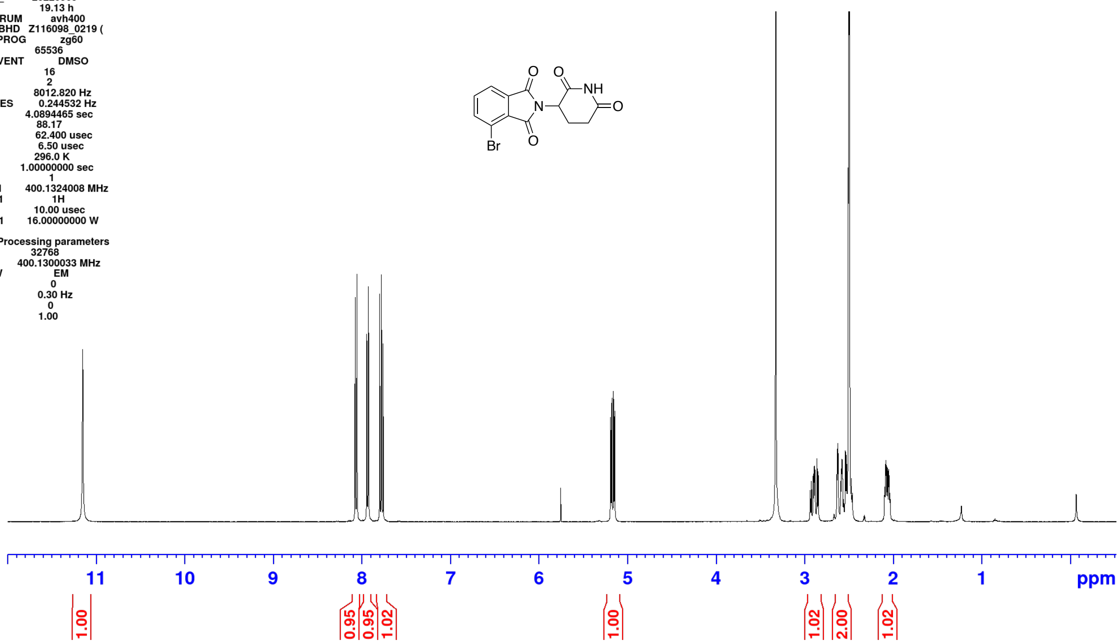

Current Data Parameters
NAME Sep09-2022-4-PJBA52
EXPNO 1
PROCNO 1

F2 - Acquisition Parameters
Date_ 20220909
Time 19.00 h
INSTRUM avh400
PROBHD Z116098_0219 (
PULPROG zg60
TD 65536
SOLVENT DMSO
NS 16
DS 2
SWH 8012.820 Hz
FIDRES 0.244532 Hz
AQ 4.0894465 sec
RG 88.17
DW 62.400 usec
DE 6.50 usec
TE 296.5 K
D1 1.00000000 sec
TD0 1
SFO1 400.1324008 MHz
NUC1 1H
P1 10.00 usec
PLW1 16.00000000 W

F2 - Processing parameters
SI 32768
SF 400.1300032 MHz
WDW EM
SSB 0
LB 0.30 Hz
GB 0
PC 1.00

5-Bromo-2-(2,6-dioxopiperidin-3-yl)isoindoline-1,3-dione (**13**)

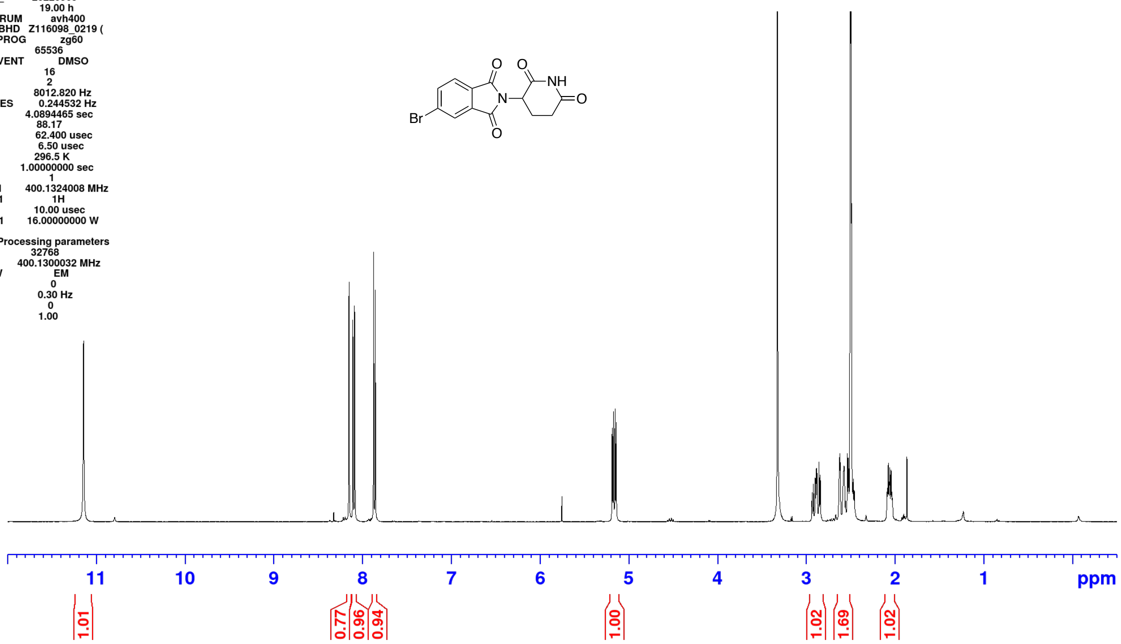

Current Data Parameters
NAME Sep09-2022-8-PJBB01
EXPNO 1
PROCNO 1

F2 - Acquisition Parameters
Date_ 20220909
Time 19.18 h
INSTRUM avh400
PROBHD Z116098_0219 (
PULPROG zg60
TD 65536
SOLVENT DMSO
NS 16
DS 2
SWH 8012.820 Hz
FIDRES 0.244532 Hz
AQ 4.0894465 sec
RG 88.17
DW 62.400 usec
DE 6.50 usec
TE 295.9 K
D1 1.00000000 sec
TD0 1
SFO1 400.1324008 MHz
NUC1 1H
P1 10.00 usec
PLW1 16.00000000 W

F2 - Processing parameters
SI 32768
SF 400.1300033 MHz
WDW EM
SSB 0
LB 0.30 Hz
GB 0
PC 1.00

2-(2,6-Dioxopiperidin-3-yl)-4-iodoisoindoline-1,3-dione (**14**)

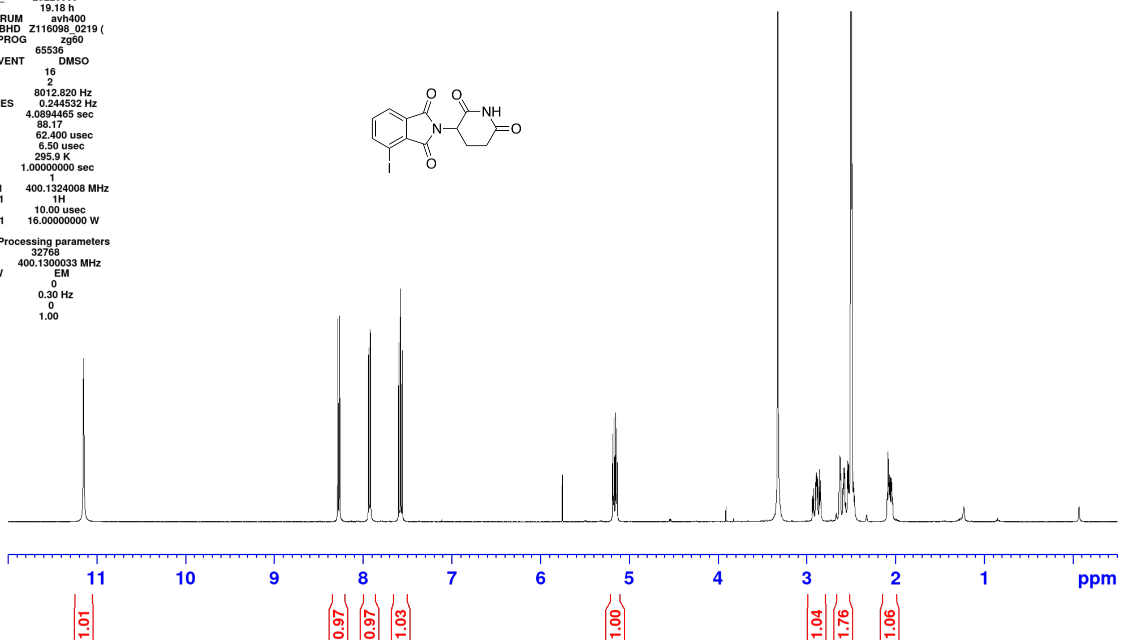

2-(2,6-Dioxopiperidin-3-yl)-5-iodoisoindoline-1,3-dione (**15**)

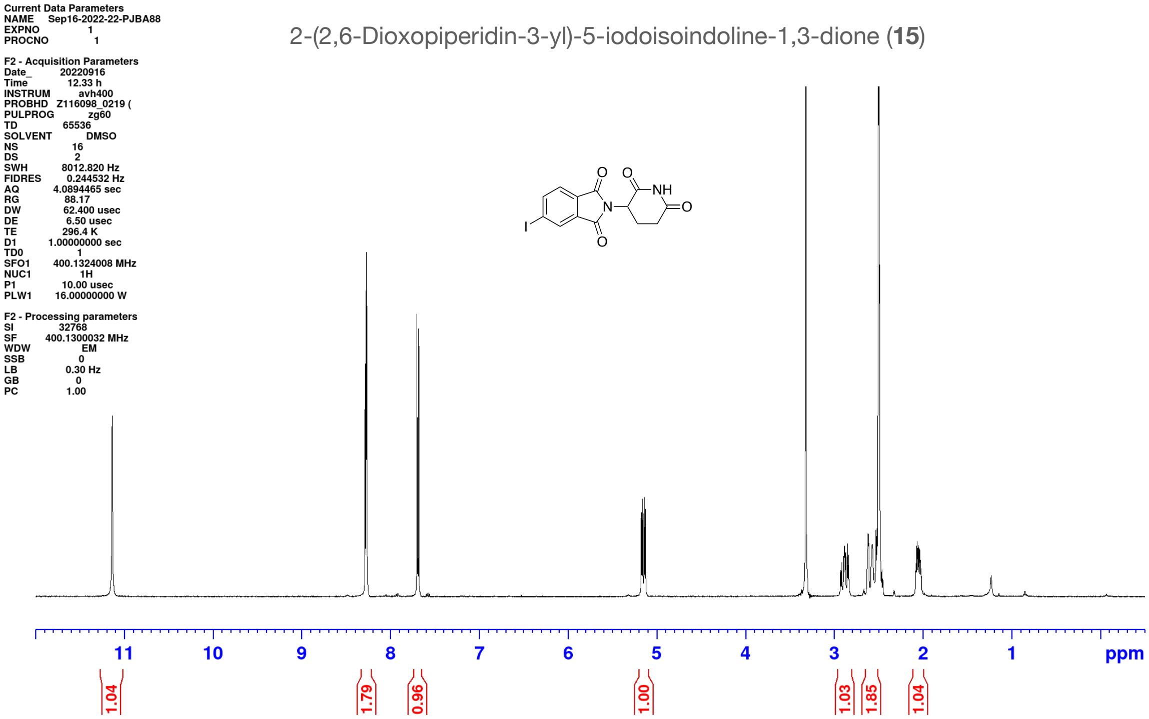

2-(2,6-Dioxopiperidin-3-yl)-5-iodoisoindoline-1,3-dione (**15**)

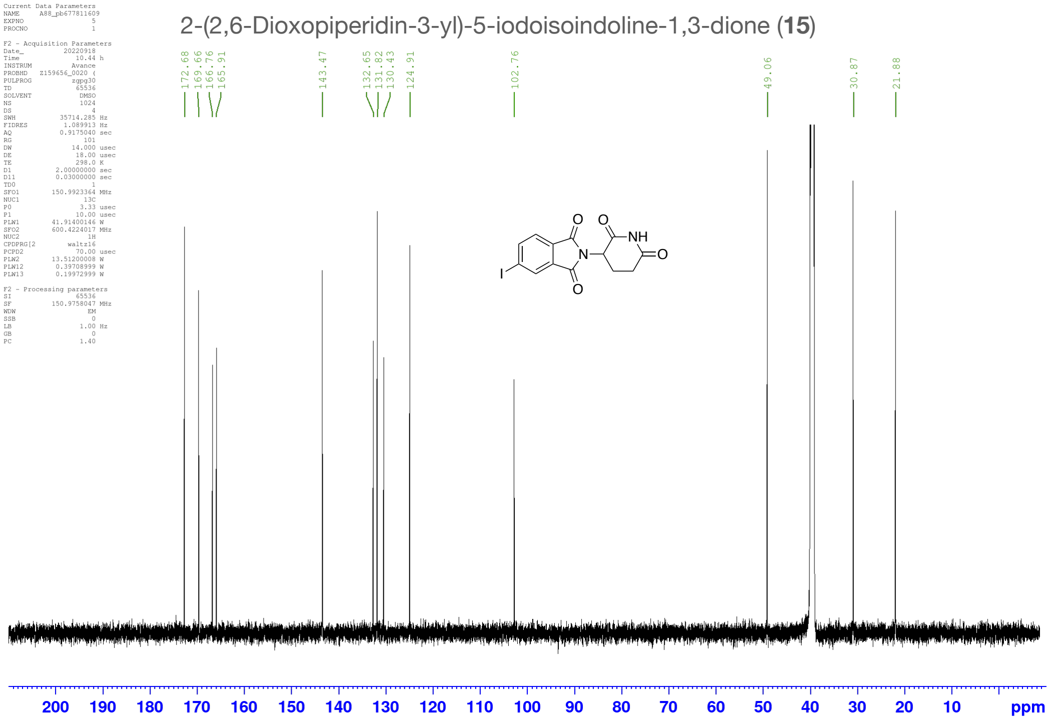

Current Data Parameters
NAME Feb04-2020-1-PJB-postcol1-DMSO__A13
EXPNO 1
PROCNO 1

F2 - Acquisition Parameters
Date_ 20200204
Time 12.15 h
INSTRUM avh400
PROBHD Z108618_0873 (
PULPROG zg60
TD 65536
SOLVENT DMSO
NS 16
DS 2
SWH 8012.820 Hz
FIDRES 0.244532 Hz
AQ 4.0894465 sec
RG 88.17
DW 62.400 usec
DE 6.50 usec
TE 298.8 K
D1 1.00000000 sec
TD0 1
SFO1 400.1324008 MHz
NUC1 1H
P1 14.00 usec
PLW1 14.36999989 W

F2 - Processing parameters
SI 32768
SF 400.1300031 MHz
WDW EM
SSB 0
LB 0.30 Hz
GB 0
PC 1.00

2-(2,6-Dioxopiperidin-3-yl)-4-methylisoindoline-1,3-dione (**16**)

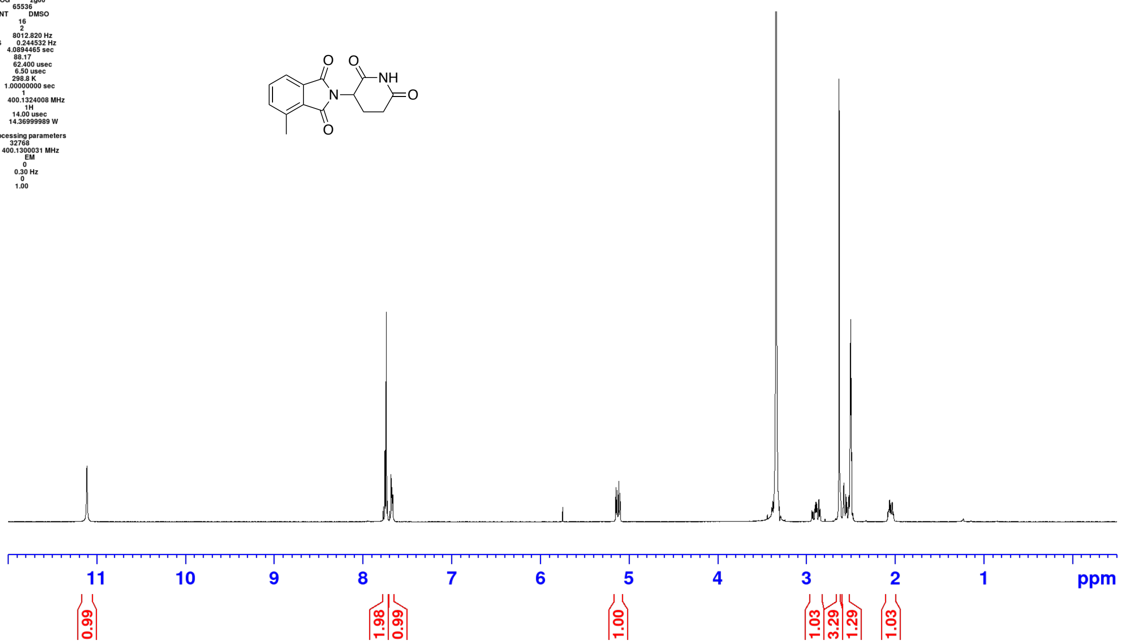

Current Data Parameters
NAME Sep09-2022-2-PJBA45
EXPNO 1
PROCNO 1

F2 - Acquisition Parameters
Date_ 20220909
Time 18.51 h
INSTRUM avh400
PROBHD Z116098_0219 (
PULPROG zg60
TD 65536
SOLVENT DMSO
NS 16
DS 2
SWH 8012.820 Hz
FIDRES 0.244532 Hz
AQ 4.0894465 sec
RG 88.17
DW 62.400 usec
DE 6.50 usec
TE 296.4 K
D1 1.00000000 sec
TD0 1
SFO1 400.1324008 MHz
NUC1 1H
P1 10.00 usec
PLW1 16.00000000 W

F2 - Processing parameters
SI 32768
SF 400.1300034 MHz
WDW EM
SSB 0
LB 0.30 Hz
GB 0
PC 1.00

2-(2,6-Dioxopiperidin-3-yl)-5-methylisoindoline-1,3-dione (**17**)

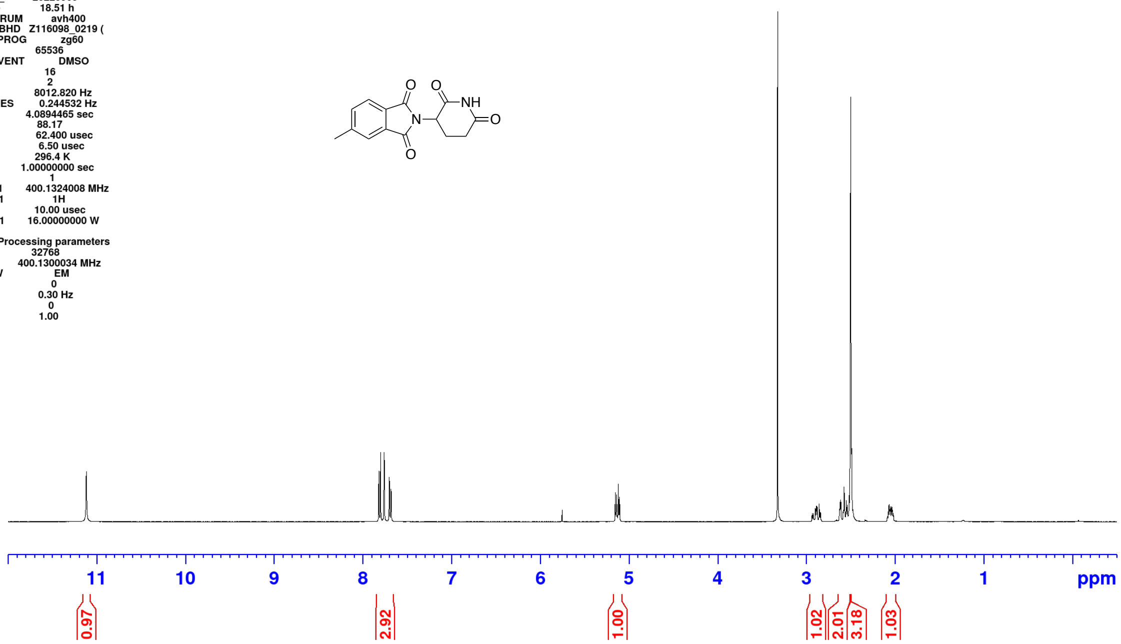

2-(2,6-Dioxopiperidin-3-yl)-5-(trifluoromethyl)isoindoline-1,3-dione (**18**)

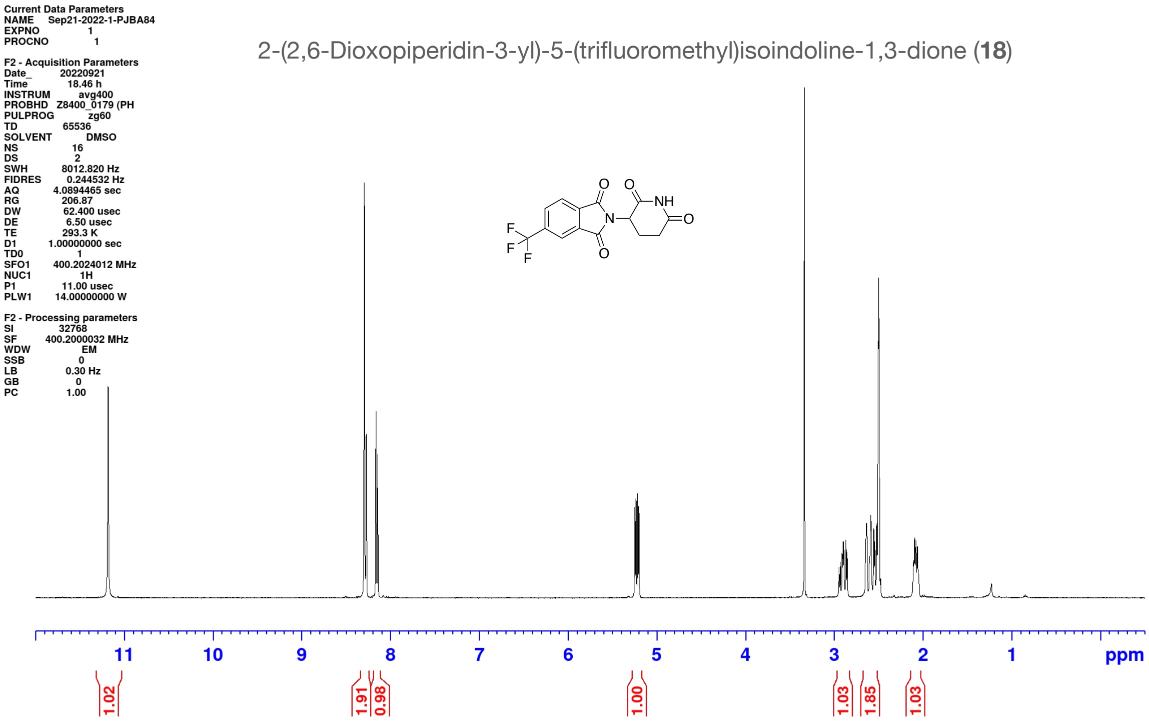

2-(2,6-Dioxopiperidin-3-yl)-5-(trifluoromethyl)isoindoline-1,3-dione (**18**)

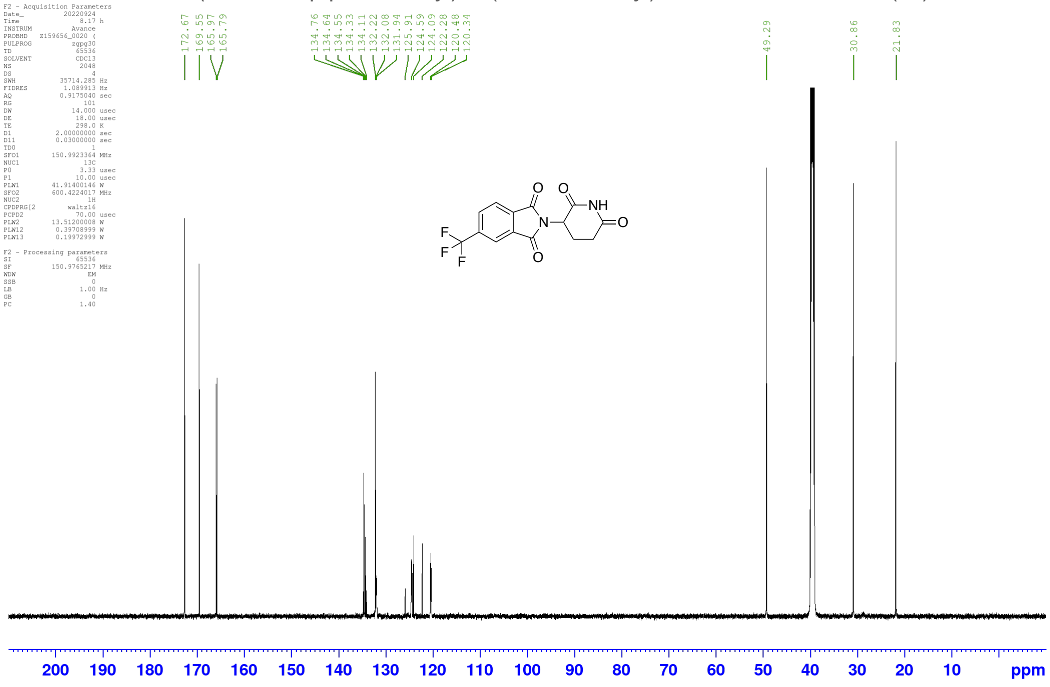

Current Data Parameters
NAME Sep09-2022-11-PJBA63_clean
EXPNO 1
PROCNO 1

F2 - Acquisition Parameters
Date_ 20220909
Time 19.31 h
INSTRUM avh400
PROBHD Z116098_0219 (
PULPROG zg60
TD 65536
SOLVENT DMSO
NS 16
DS 2
SWH 8012.820 Hz
FIDRES 0.244532 Hz
AQ 4.0894465 sec
RG 88.17
DW 62.400 usec
DE 6.50 usec
TE 296.4 K
D1 1.00000000 sec
TD0 1
SFO1 400.1324008 MHz
NUC1 1H
P1 10.00 usec
PLW1 16.00000000 W

F2 - Processing parameters
SI 32768
SF 400.1300032 MHz
WDW EM
SSB 0
LB 0.30 Hz
GB 0
PC 1.00

5-(Tert-butyl)-2-(2,6-dioxopiperidin-3-yl)isoindoline-1,3-dione (**19**)

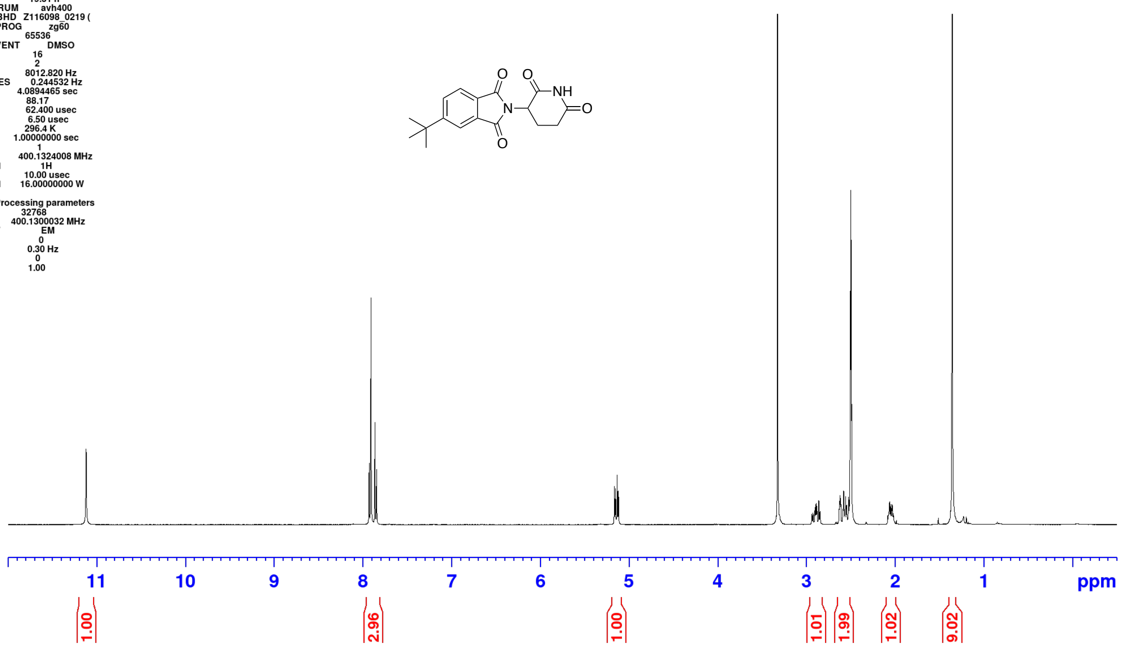

Current Data Parameters
NAME Sep09-2022-9-PJBB02
EXPNO 1
PROCNO 1

F2 - Acquisition Parameters
Date_ 20220909
Time 19.22 h
INSTRUM avh400
PROBHD Z116098_0219 (
PULPROG zg60
TD 65536
SOLVENT DMSO
NS 16
DS 2
SWH 8012.820 Hz
FIDRES 0.244532 Hz
AQ 4.0894465 sec
RG 88.17
DW 62.400 usec
DE 6.50 usec
TE 296.0 K
D1 1.00000000 sec
TD0 1
SFO1 400.1324008 MHz
NUC1 1H
P1 10.00 usec
PLW1 16.00000000 W

F2 - Processing parameters
SI 32768
SF 400.1300032 MHz
WDW EM
SSB 0
LB 0.30 Hz
GB 0
PC 1.00

2-(2,6-Dioxopiperidin-3-yl)-5-phenylisoindoline-1,3-dione (**20**)

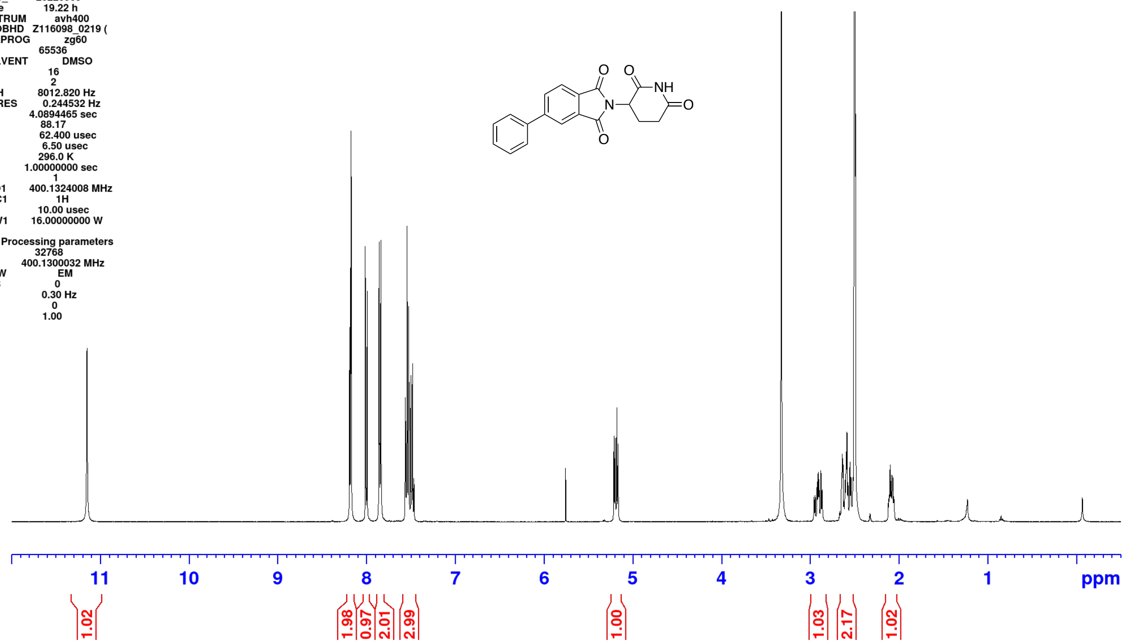

Current Data Parameters
NAME Dec17-2020-3-PJBA62-postcol1
EXPNO 1
PROCNO 1

F2 - Acquisition Parameters
Date_ 20201217
Time 18.34 h
INSTRUM avh400
PROBHD Z108618_0873 (
PULPROG zg60
TD 65536
SOLVENT CDCl3
NS 16
DS 2
SWH 8012.820 Hz
FIDRES 0.244532 Hz
AQ 4.0894465 sec
RG 88.17
DW 62.400 usec
DE 6.50 usec
TE 299.8 K
D1 1.00000000 sec
TD0 1
SFO1 400.1324008 MHz
NUC1 1H
P1 14.00 usec
PLW1 14.36999989 W

F2 - Processing parameters
SI 32768
SF 400.1300101 MHz
WDW EM
SSB 0
LB 0.30 Hz
GB 0
PC 1.00

5-(Dimethylamino)-2-(2,6-dioxopiperidin-3-yl)isoindoline-1,3-dione (**21**)

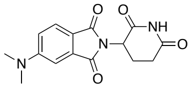

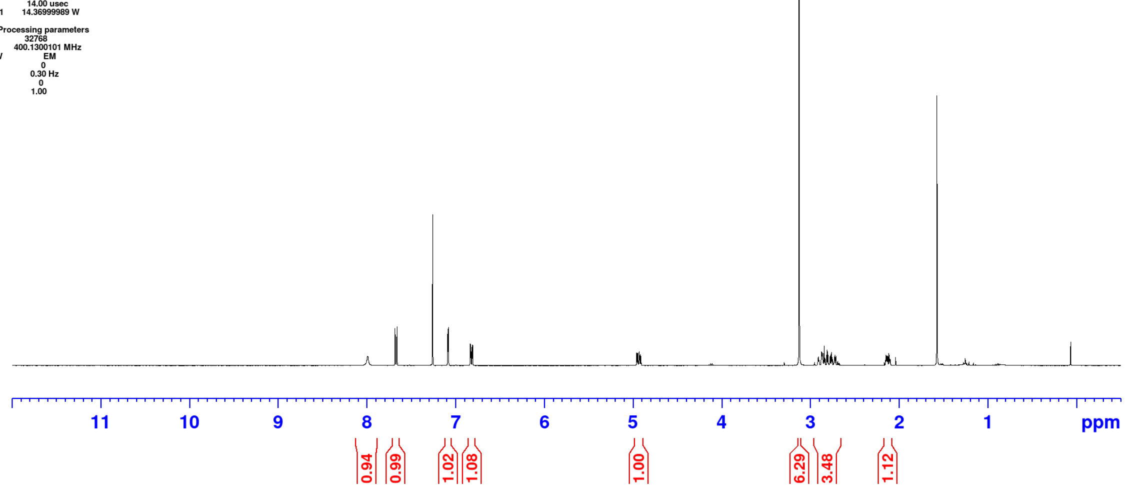

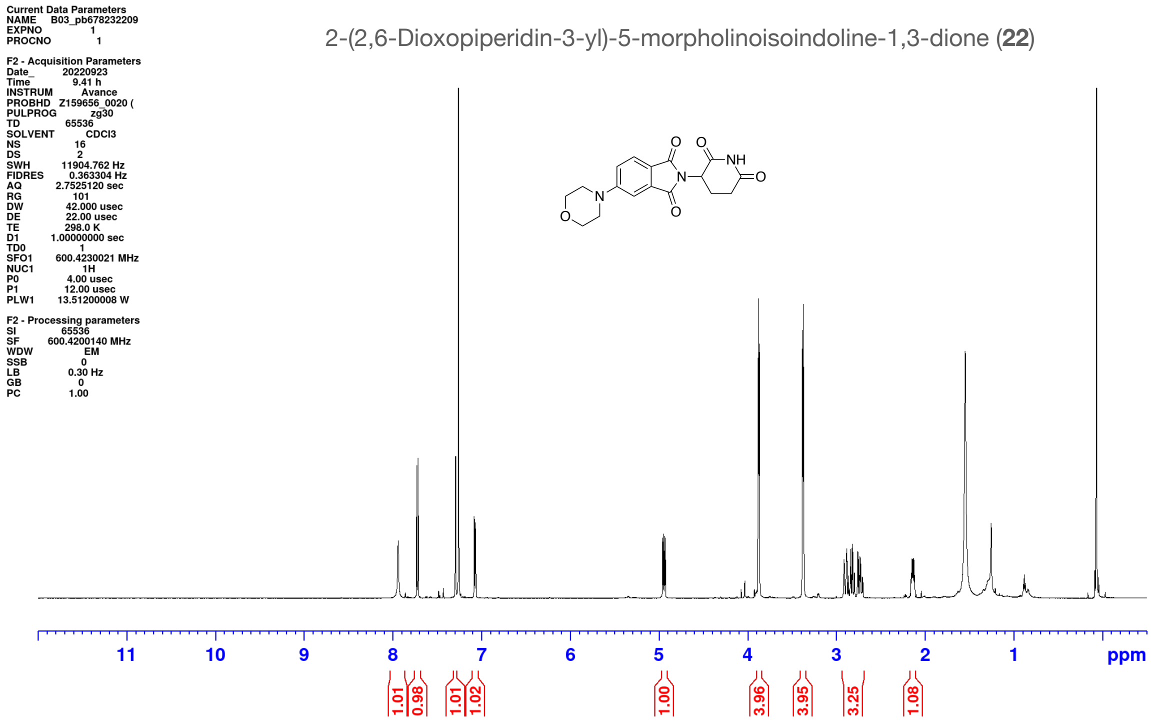

Current Data Parameters
NAME B03_pb678232209
EXPNO 1
PROCNO 1
F2 - Acquisition Parameters
Date_ 20220923
Time 9.41 h
INSTRUM Avance
PROBHD Z159656_0020 (
PULPROG zg30
TD 65536
SOLVENT CDCl3
NS 16
DS 2
SWH 11904.762 Hz
FIDRES 0.363304 Hz
AQ 2.7525120 sec
RG 101
DW 42.000 usec
DE 22.00 usec
TE 298.0 K
D1 1.00000000 sec
TD0 1
SFO1 600.4230021 MHz
NUC1 1H
P0 4.00 usec
P1 12.00 usec
PLW1 13.51200008 W
F2 - Processing parameters
SI 65536
SF 600.4200140 MHz
WDW EM
SSB 0
LB 0.30 Hz
GB 0
PC 1.00
2-(2,6-Dioxopiperidin-3-yl)-5-morpholinoisoindoline-1,3-dione (22)
11 10 9 8 7 6 5 4 3 2 1 ppm
1.01 0.98 1.01 1.02 1.00 3.96 3.95 3.25 1.08

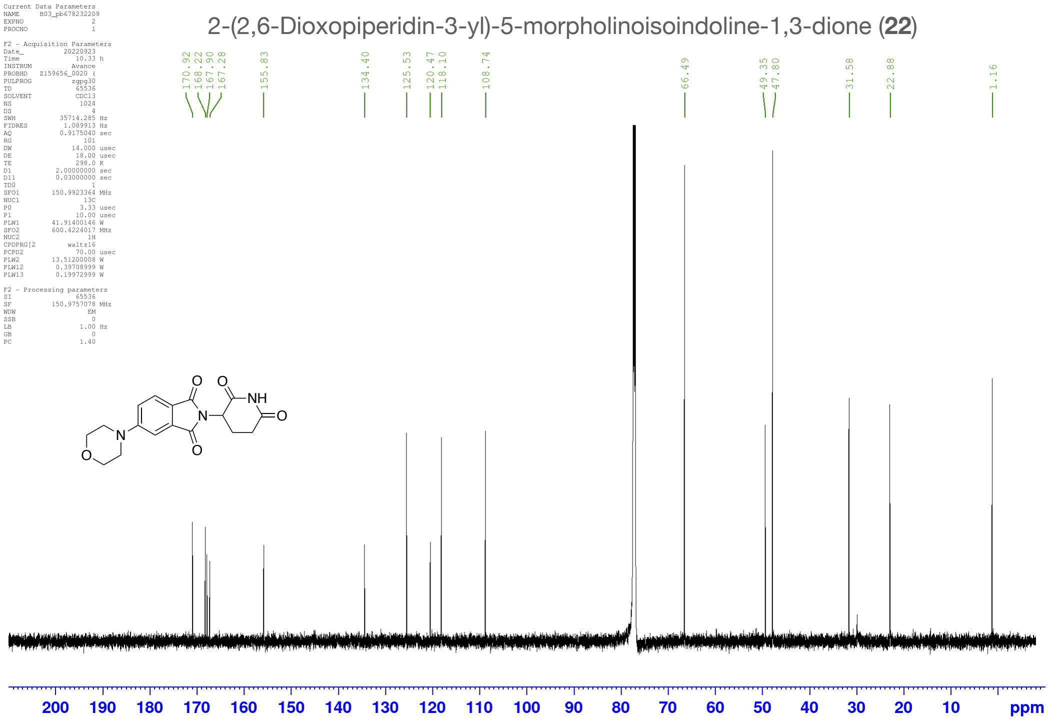

Current Data Parameters
NAME B03_pb678232209
EXPNO 2
PROCNO 1
F2 - Acquisition Parameters
Date_ 20220923
Time 10.33 h
INSTRUM Avance
PROBHD Z159656_0020 (
PULPROG zgpg30
TD 65536
SOLVENT CDCl3
NS 1024
DS 4
SWH 35714.285 Hz
FIDRES 1.089913 Hz
AQ 0.9175040 sec
RG 101
DW 14.000 usec
DE 18.00 usec
TE 298.0 K
D1 2.00000000 sec
D11 0.03000000 sec
TD0 1
SFO1 150.9923364 MHz
NUC1 13C
P0 3.33 usec
P1 10.00 usec
PLW1 41.91400146 W
SFO2 600.4224017 MHz
NUC2 1H
CPDPRG[2 waltz16
PCPD2 70.00 usec
PLW2 13.51200008 W
PLW12 0.39708999 W
PLW13 0.19972999 W
F2 - Processing parameters
SI 65536
SF 150.9757078 MHz
WDW EM
SSB 0
LB 1.00 Hz
GB 0
PC 1.40
2-(2,6-Dioxopiperidin-3-yl)-5-morpholinoisoindoline-1,3-dione (22)
170.92 168.22 167.90 167.28 155.83 134.40 125.53 120.47 118.10 108.74 66.49 49.35 47.80 31.58 22.88 1.16
200 190 180 170 160 150 140 130 120 110 100 90 80 70 60 50 40 30 20 10 ppm

2-(2,6-Dioxopiperidin-3-yl)-5-(4-methylpiperazin-1-yl)isoindoline-1,3-dione (**23**)

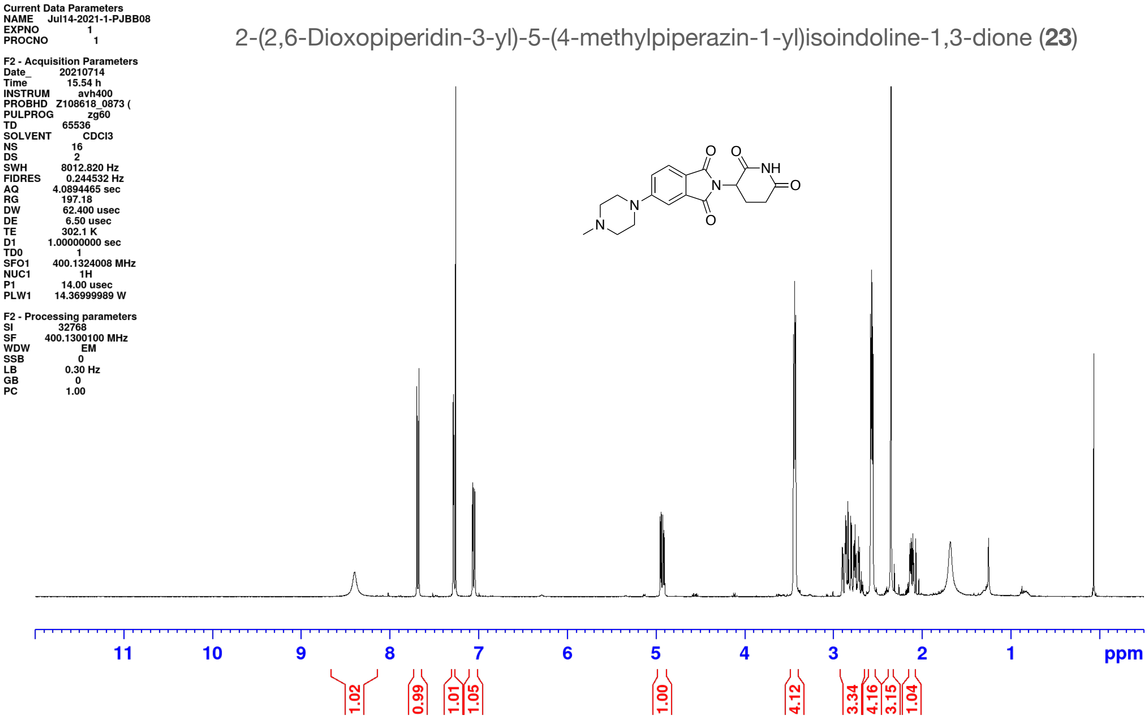

2-(2,6-Dioxopiperidin-3-yl)-5-(4-methylpiperazin-1-yl)isoindoline-1,3-dione (**23**)

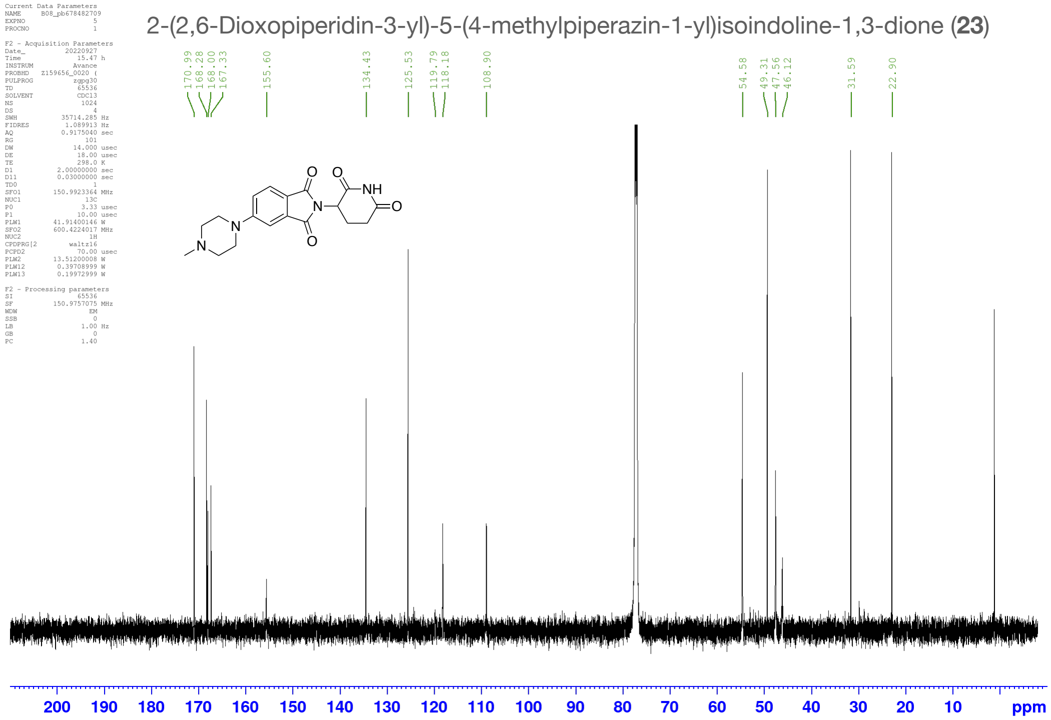

2-(2,6-Dioxopiperidin-3-yl)-5-(4-methylpiperazin-1-yl)isoindoline-1,3-dione (**23**)

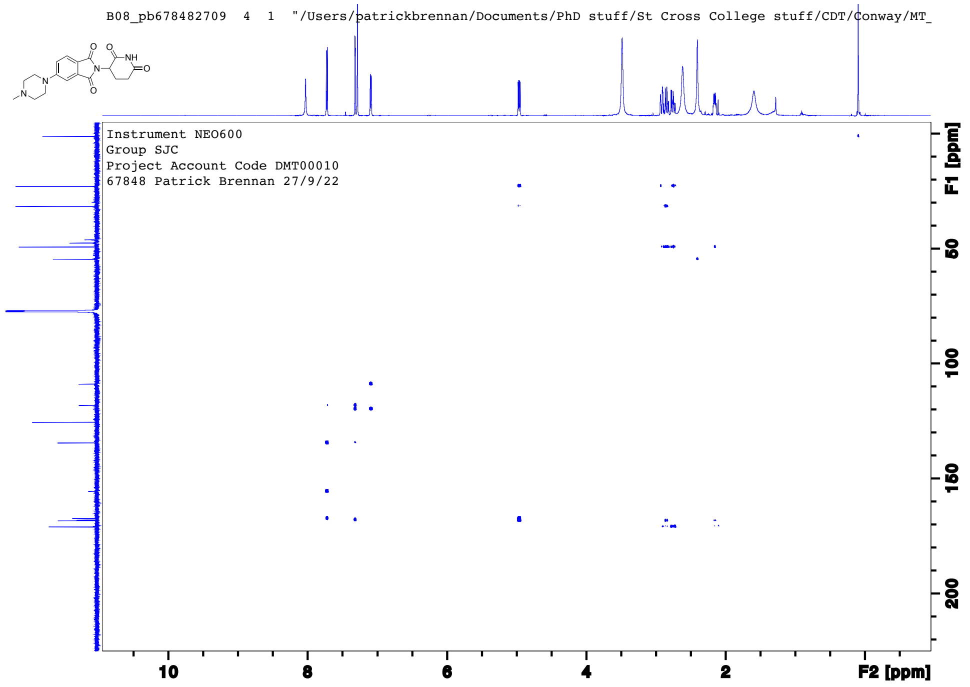

2-(2,6-Dioxopiperidin-3-yl)-5-(4-methylpiperazin-1-yl)isoindoline-1,3-dione (**23**)

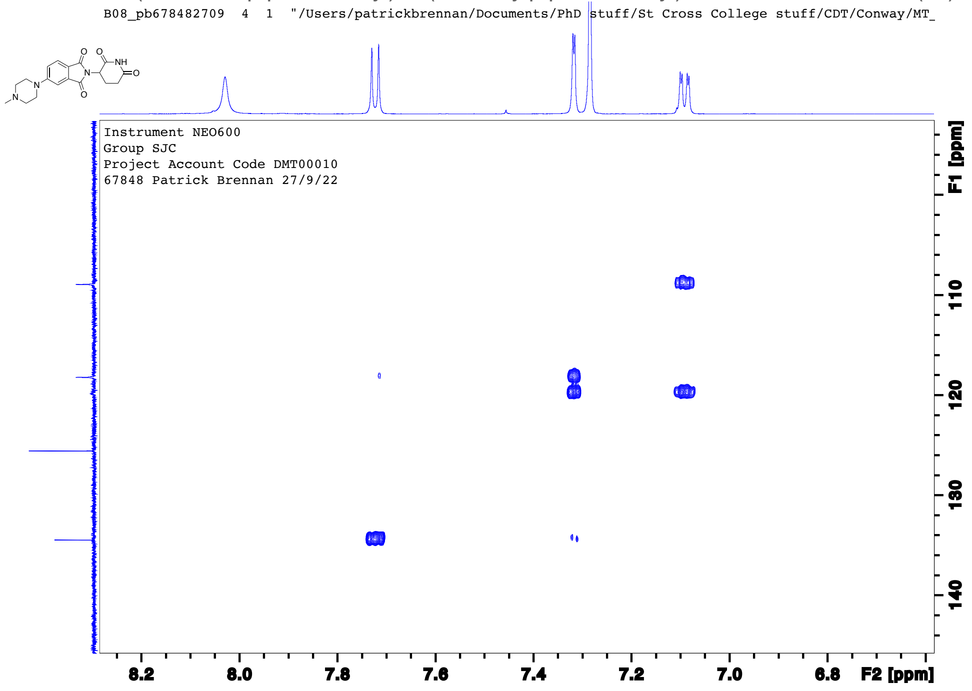

Current Data Parameters
NAME Mar10-2020-1-PJBA27-postcol-25to42
EXPNO 1
PROCNO 1

F2 - Acquisition Parameters
Date_ 20200310
Time 9.28 h
INSTRUM avh400
PROBHD Z108618_0873 (
PULPROG zg60
TD 65536
SOLVENT CDCl3
NS 16
DS 2
SWH 8012.820 Hz
FIDRES 0.244532 Hz
AQ 4.0894465 sec
RG 88.17
DW 62.400 usec
DE 6.50 usec
TE 299.8 K
D1 1.00000000 sec
TD0 1
SFO1 400.1324008 MHz
NUC1 1H
P1 14.00 usec
PLW1 14.36999989 W

F2 - Processing parameters
SI 32768
SF 400.1300097 MHz
WDW EM
SSB 0
LB 0.30 Hz
GB 0
PC 1.00

2-(2,6-Dioxopiperidin-3-yl)-4-(ethylamino)isoindoline-1,3-dione (**24**)

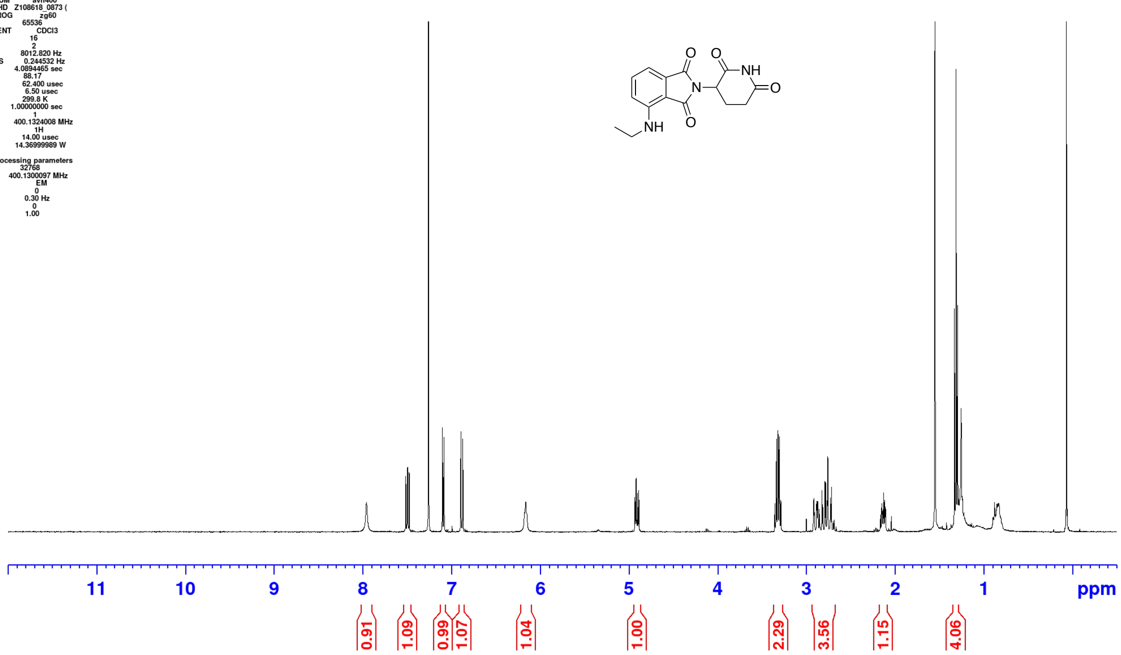

Current Data Parameters
NAME Sep13-2022-1-PJBA28_1_4
EXPNO 1
PROCNO 1

F2 - Acquisition Parameters
Date_ 20220913
Time 14.32 h
INSTRUM avg400
PROBHD Z8400_0179 (PH
PULPROG zg60
TD 65536
SOLVENT CDCl3
NS 16
DS 2
SWH 8012.820 Hz
FIDRES 0.244532 Hz
AQ 4.0894465 sec
RG 206.87
DW 62.400 usec
DE 6.50 usec
TE 293.9 K
D1 1.00000000 sec
TD0 1
SFO1 400.2024012 MHz
NUC1 1H
P1 11.00 usec
PLW1 14.00000000 W

F2 - Processing parameters
SI 32768
SF 400.2000100 MHz
WDW EM
SSB 0
LB 0.30 Hz
GB 0
PC 1.00

2-(2,6-Dioxopiperidin-3-yl)-4-(prop-2-yn-1-ylamino)isoindoline-1,3-dione (**25**)

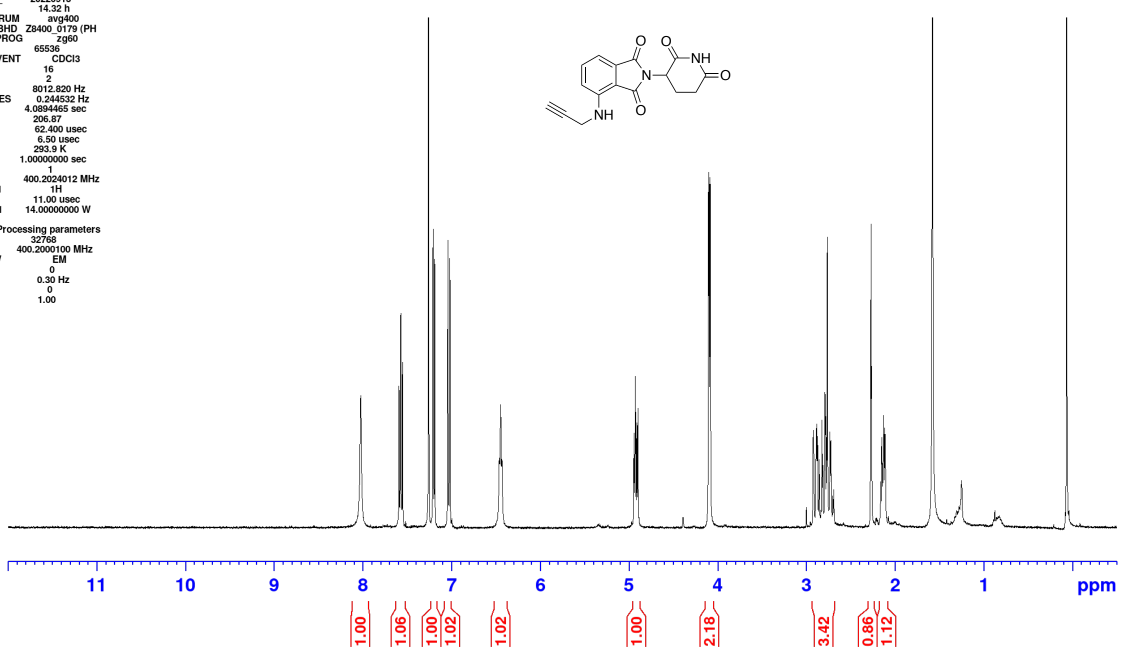

4-(Benzylamino)-2-(2,6-dioxopiperidin-3-yl)isoindoline-1,3-dione (**26**)

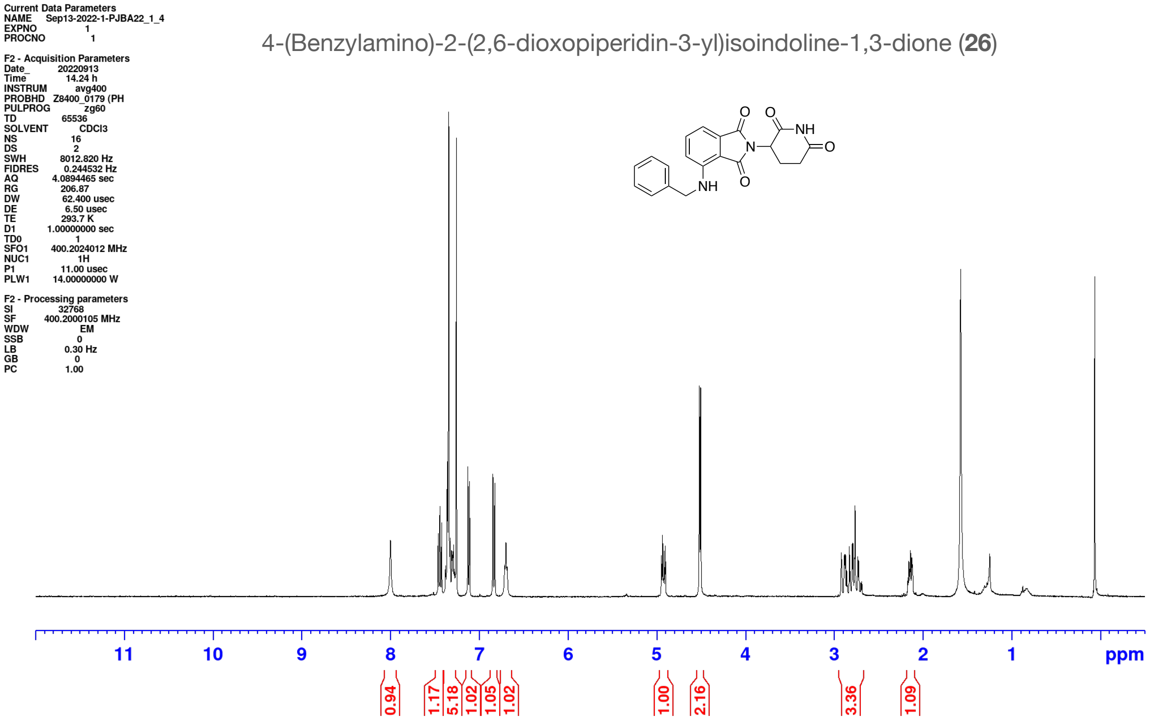

2-(2,6-Dioxopiperidin-3-yl)-4-((pyridin-3-ylmethyl)amino)isoindoline-1,3-dione (**27**)

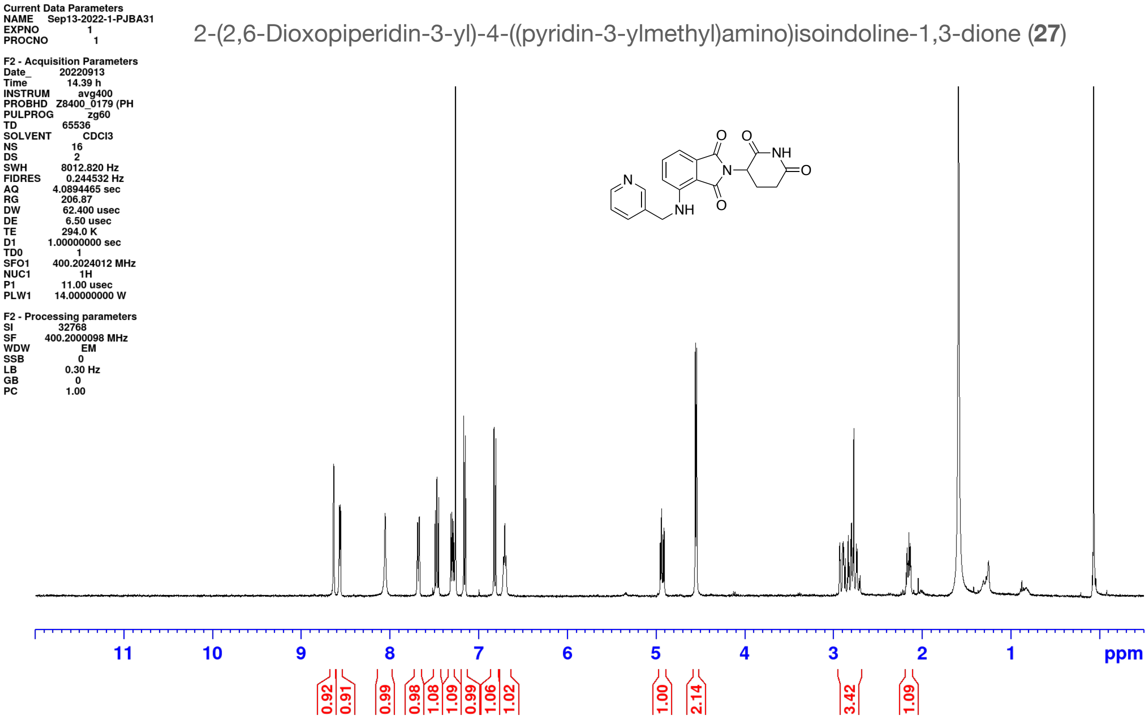

2-(2,6-Dioxopiperidin-3-yl)-4-((pyridin-3-ylmethyl)amino)isoindoline-1,3-dione (**27**)

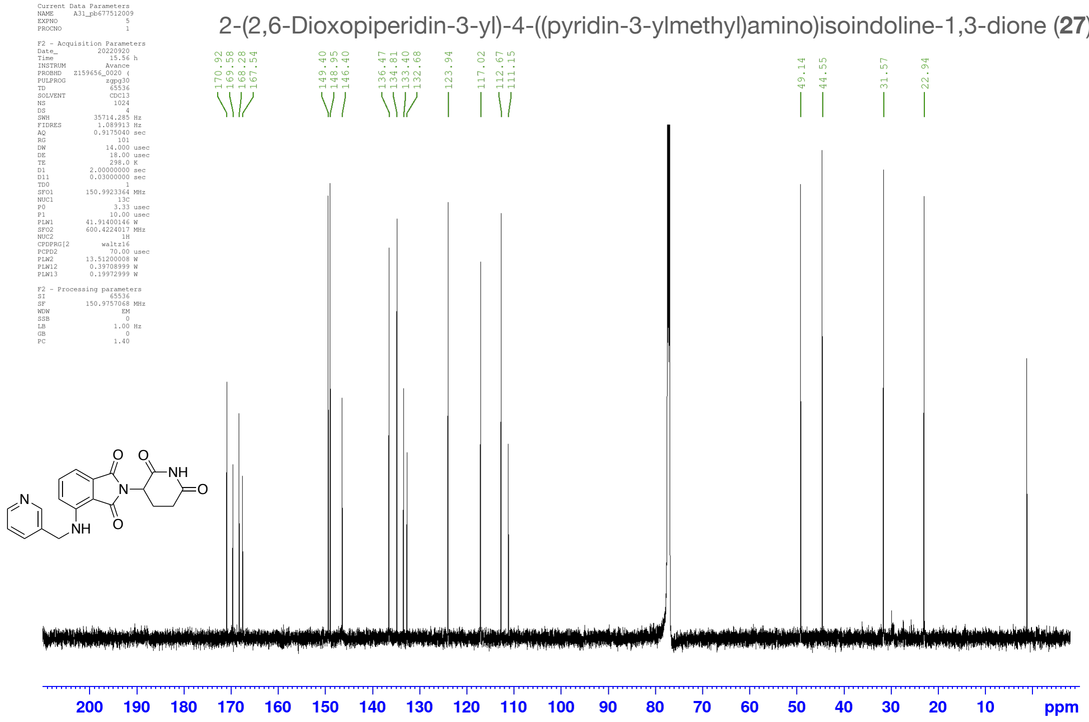

2-(2,6-Dioxopiperidin-3-yl)-4-((2-methylbenzyl)amino)isoindoline-1,3-dione (**28**)

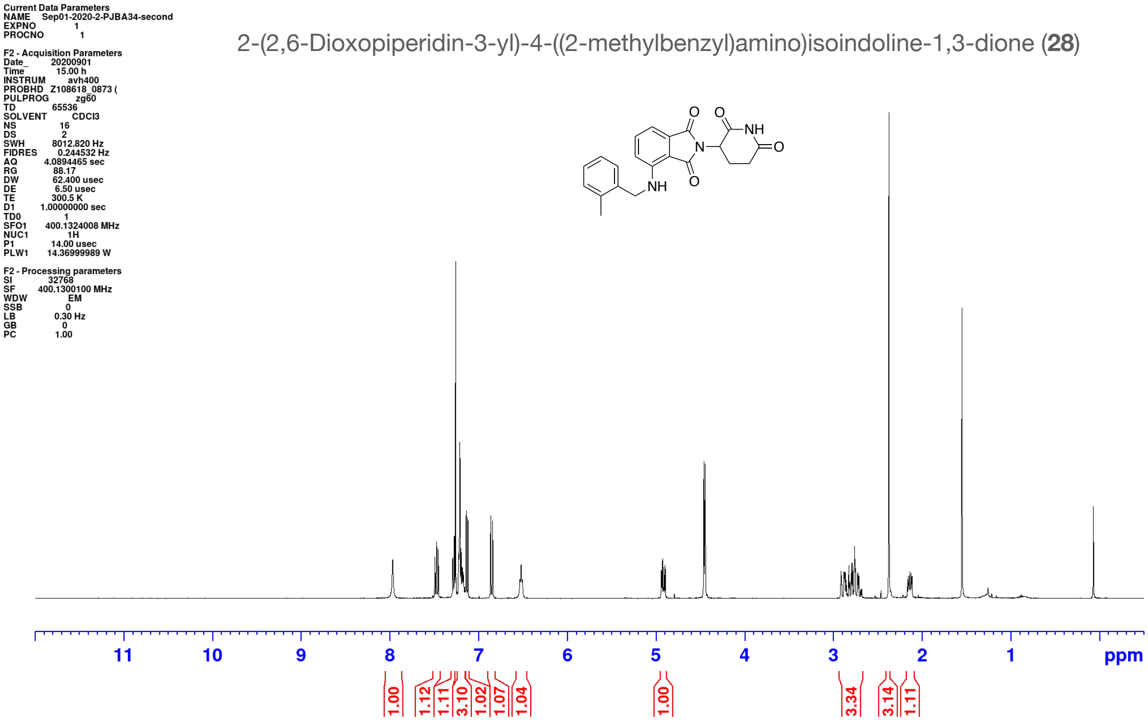

2-(2,6-Dioxopiperidin-3-yl)-4-((2-methylbenzyl)amino)isoindoline-1,3-dione (**28**)

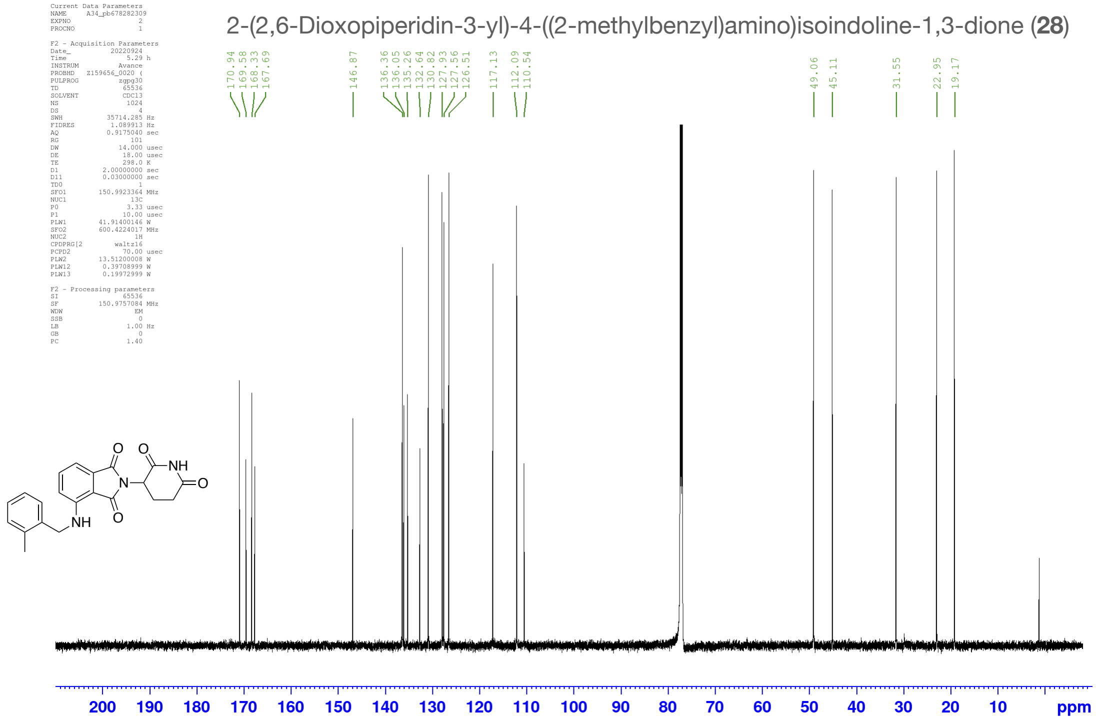

2-(2,6-Dioxopiperidin-3-yl)-4-(((4-methylpyridin-3-yl)methyl)amino)isoindoline-1,3-dione (**29**)

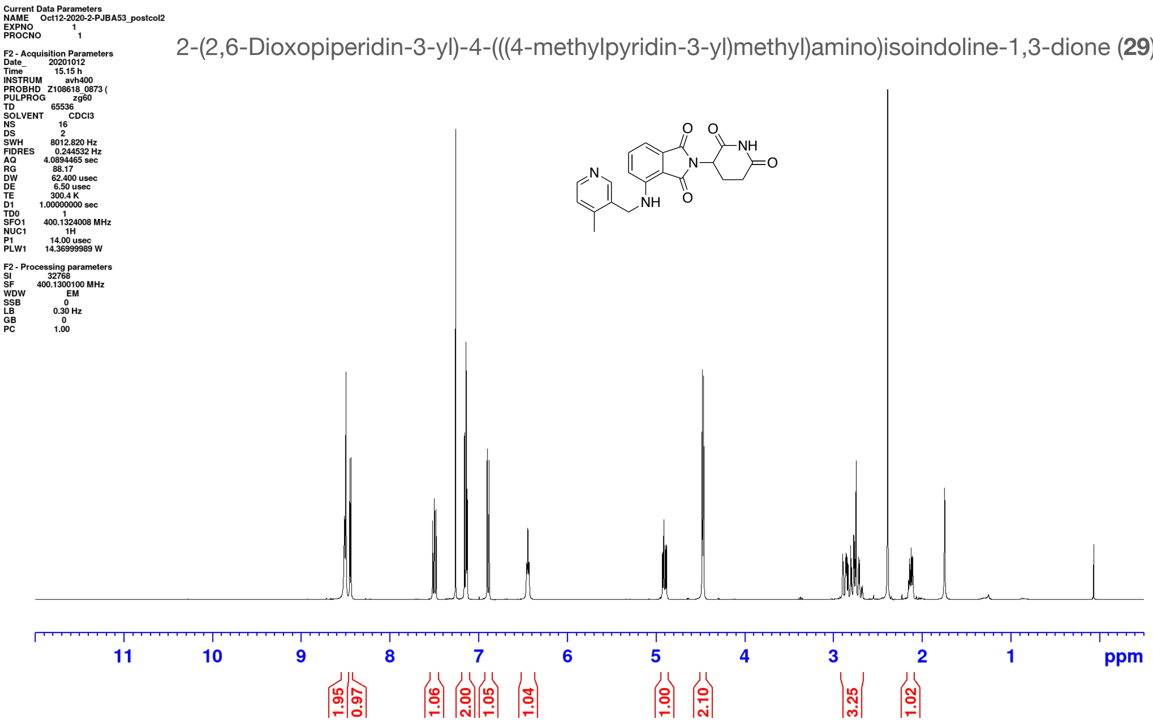

2-(2,6-Dioxopiperidin-3-yl)-4-(((4-methylpyridin-3-yl)methyl)amino)isoindoline-1,3-dione (**29**)

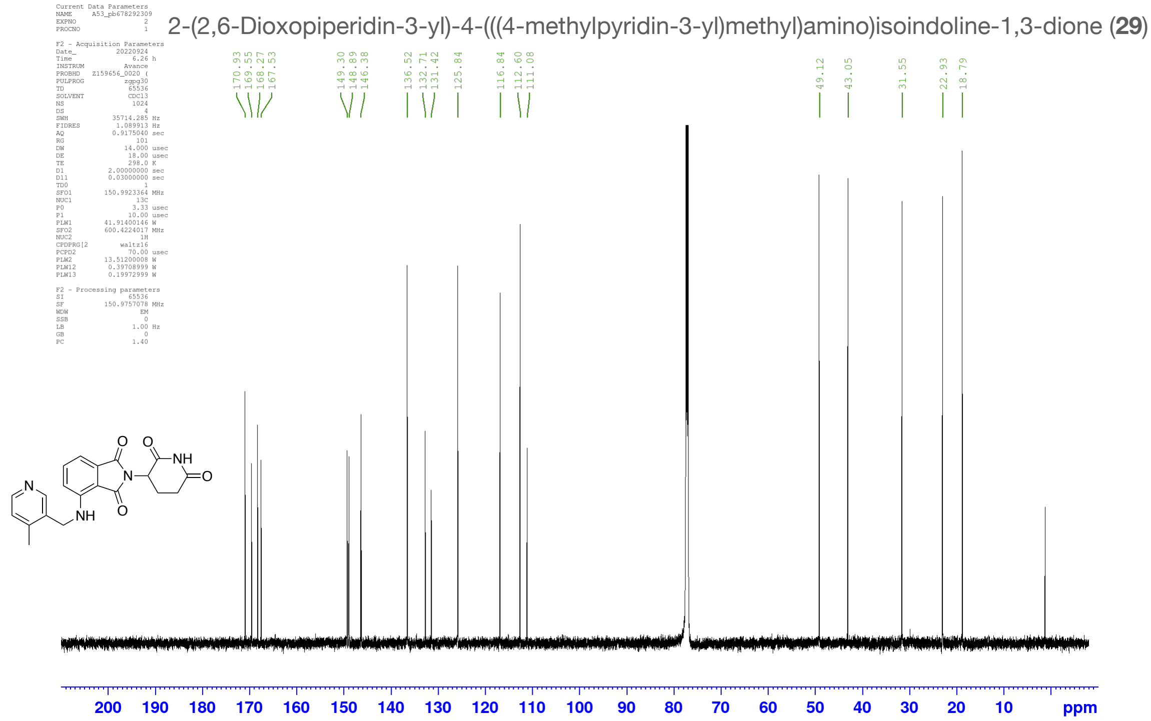

Current Data Parameters
NAME Feb21-2020-6-PJBA20-DMSO
EXPNO 1
PROCNO 1

F2 - Acquisition Parameters
Date_ 20200221
Time 18.31 h
INSTRUM avh400
PROBHD Z108618_0873 (
PULPROG zg60
TD 65536
SOLVENT DMSO
NS 16
DS 2
SWH 8012.820 Hz
FIDRES 0.244532 Hz
AQ 4.0894465 sec
RG 88.17
DW 62.400 usec
DE 6.50 usec
TE 298.8 K
D1 1.00000000 sec
TD0 1
SFO1 400.1324008 MHz
NUC1 1H
P1 14.00 usec
PLW1 14.36999989 W

F2 - Processing parameters
SI 32768
SF 400.1300028 MHz
WDW EM
SSB 0
LB 0.30 Hz
GB 0
PC 1.00

4-(Benzyloxy)-2-(2,6-dioxopiperidin-3-yl)isoindoline-1,3-dione (**30**)

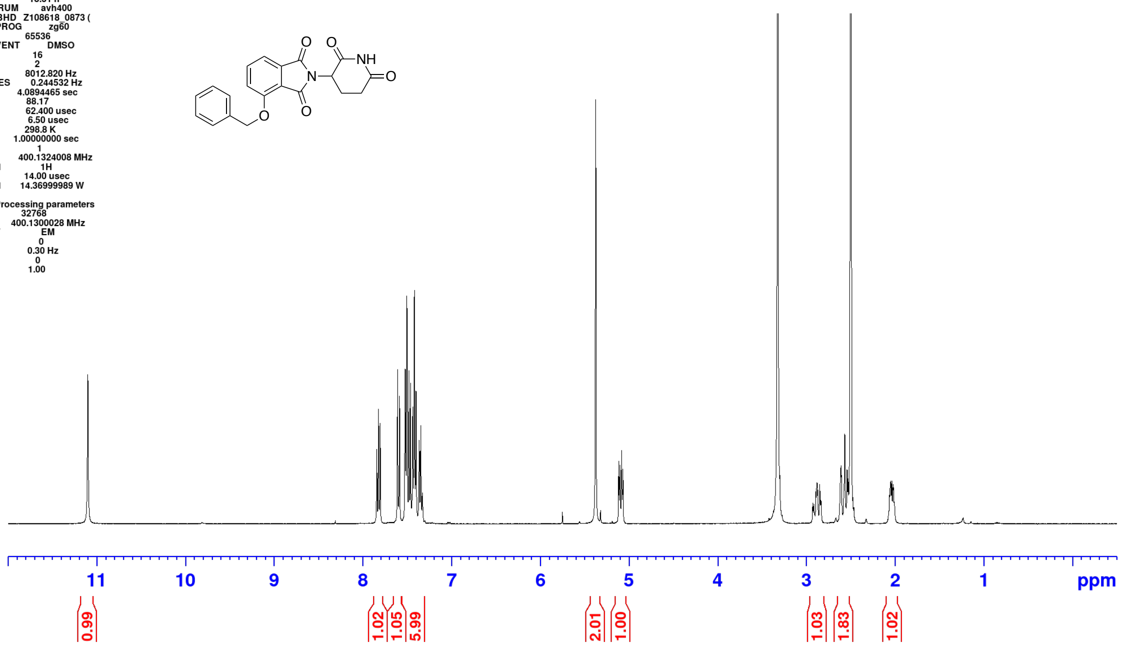

2-(2,6-Dioxopiperidin-3-yl)-4-(pyridin-3-ylmethoxy)isoindoline-1,3-dione (**31**)

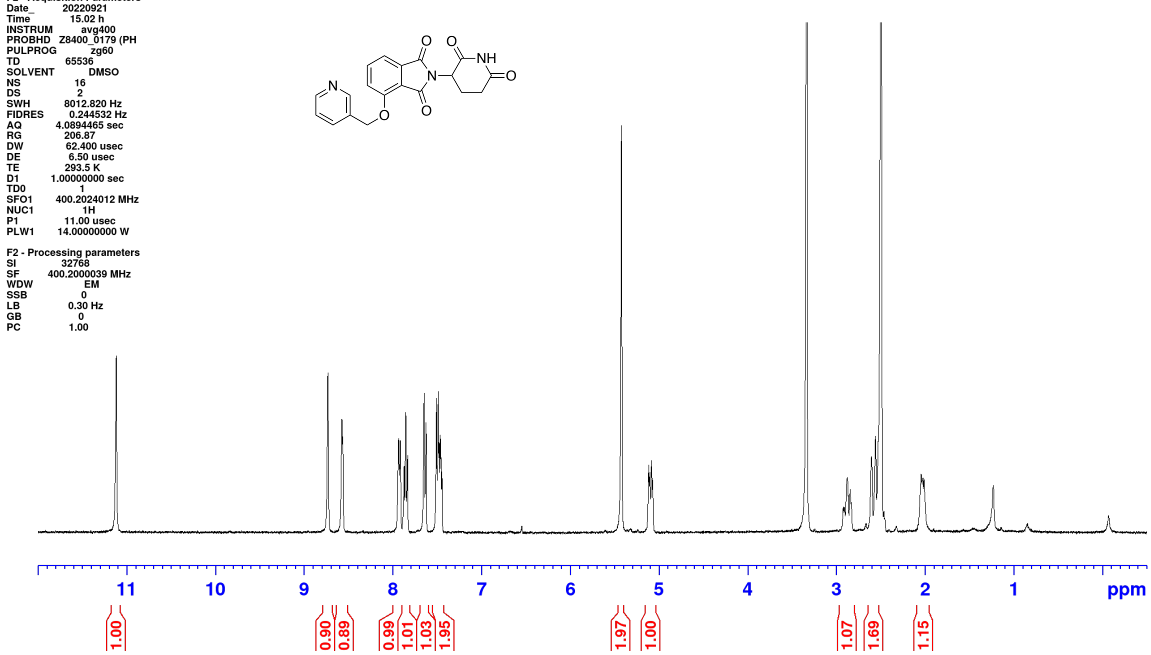

2-(2,6-Dioxopiperidin-3-yl)-4-(pyridin-3-ylmethoxy)isoindoline-1,3-dione (**31**)

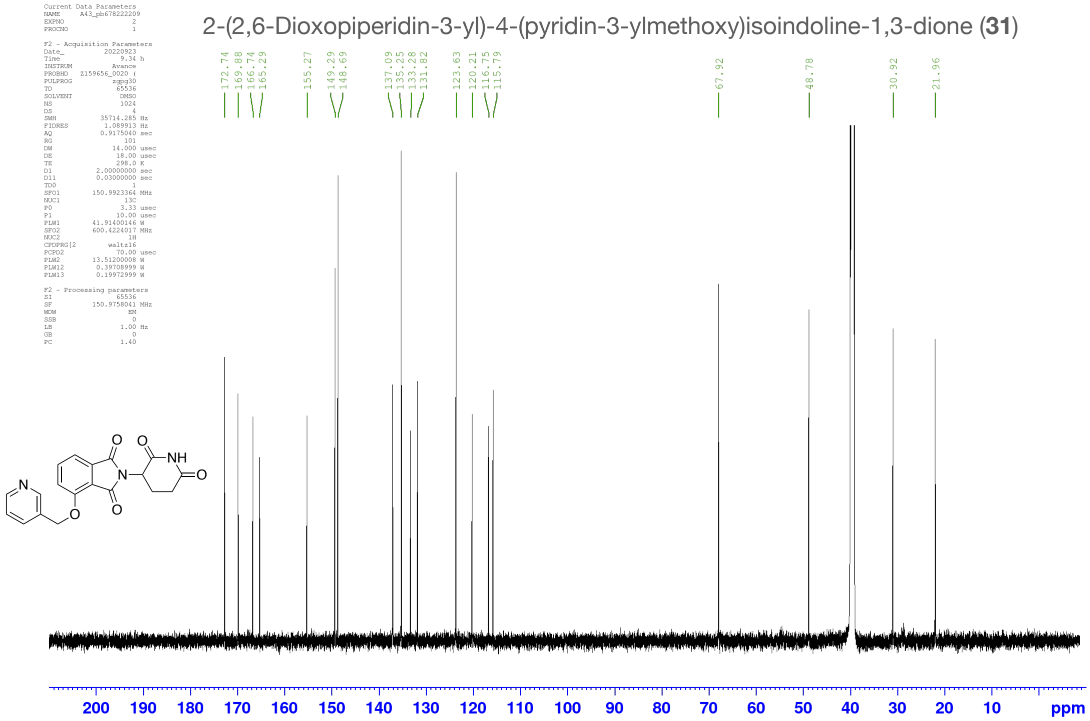

Current Data Parameters
NAME Sep21-2022-1-PJBA42
EXPNO 1
PROCNO 1

F2 - Acquisition Parameters
Date_ 20220921
Time 10.32 h
INSTRUM avg400
PROBHD Z8400_0179 (PH
PULPROG zg60
TD 65536
SOLVENT DMSO
NS 16
DS 2
SWH 8012.820 Hz
FIDRES 0.244532 Hz
AQ 4.0894465 sec
RG 206.87
DW 62.400 usec
DE 6.50 usec
TE 294.0 K
D1 1.00000000 sec
TD0 1
SFO1 400.2024012 MHz
NUC1 1H
P1 11.00 usec
PLW1 14.00000000 W

F2 - Processing parameters
SI 32768
SF 400.2000035 MHz
WDW EM
SSB 0
LB 0.30 Hz
GB 0
PC 1.00

2-(2,6-Dioxopiperidin-3-yl)-4-((2-methylbenzyl)oxy)isoindoline-1,3-dione (**32**)

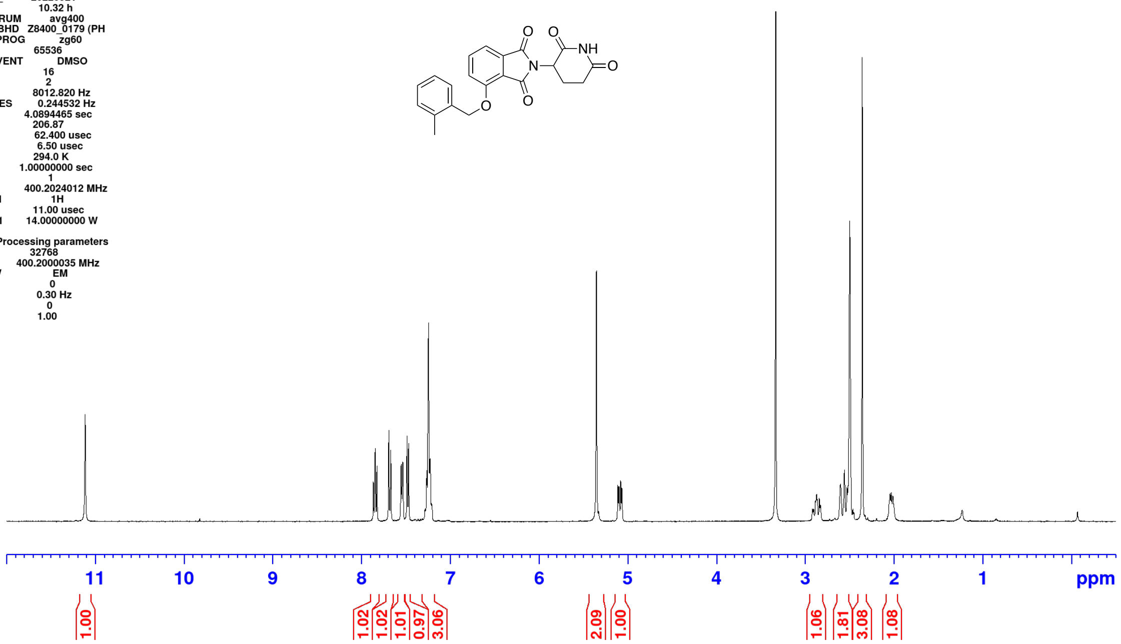

Current Data Parameters
NAME A42_pb678172209
EXPNO 5
PROCNO 1

F2 - Acquisition Parameters
Date_ 20220923
Time 4.45 h
INSTRUM Avance
PROBHD Z159656_0020 (
PULPROG zgpg30
TD 65536
SOLVENT DMSO
NS 512
DS 4
SWH 35714.285 Hz
FIDRES 1.089913 Hz
AQ 0.9175040 sec
RG 101
DW 14.000 usec
DE 18.00 usec
TE 298.0 K
D1 2.00000000 sec
D11 0.03000000 sec
TD0 1
SFO1 150.9923364 MHz
NUC1 13C
P0 3.33 usec
P1 10.00 usec
PLW1 41.91400146 W
SFO2 600.4224017 MHz
NUC2 1H
CPDPRG[2 waltz16
PCPD2 70.00 usec
PLW2 13.51200008 W
PLW12 0.39708999 W
PLW13 0.19972999 W

F2 - Processing parameters
SI 65536
SF 150.9758043 MHz
WDW EM
SSB 0
LB 1.00 Hz
GB 0
PC 1.40

2-(2,6-Dioxopiperidin-3-yl)-4-((2-methylbenzyl)oxy)isoindoline-1,3-dione (**32**)

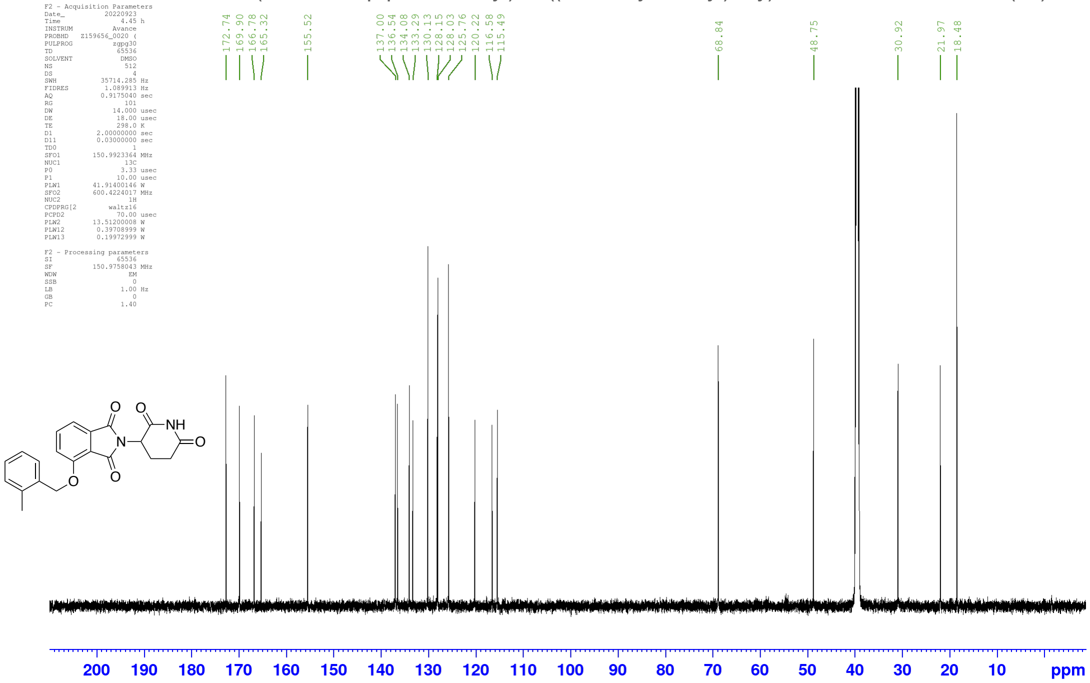

4-(Benzhydrylamino)-2-(2,6-dioxopiperidin-3-yl)isoindoline-1,3-dione (**33**)

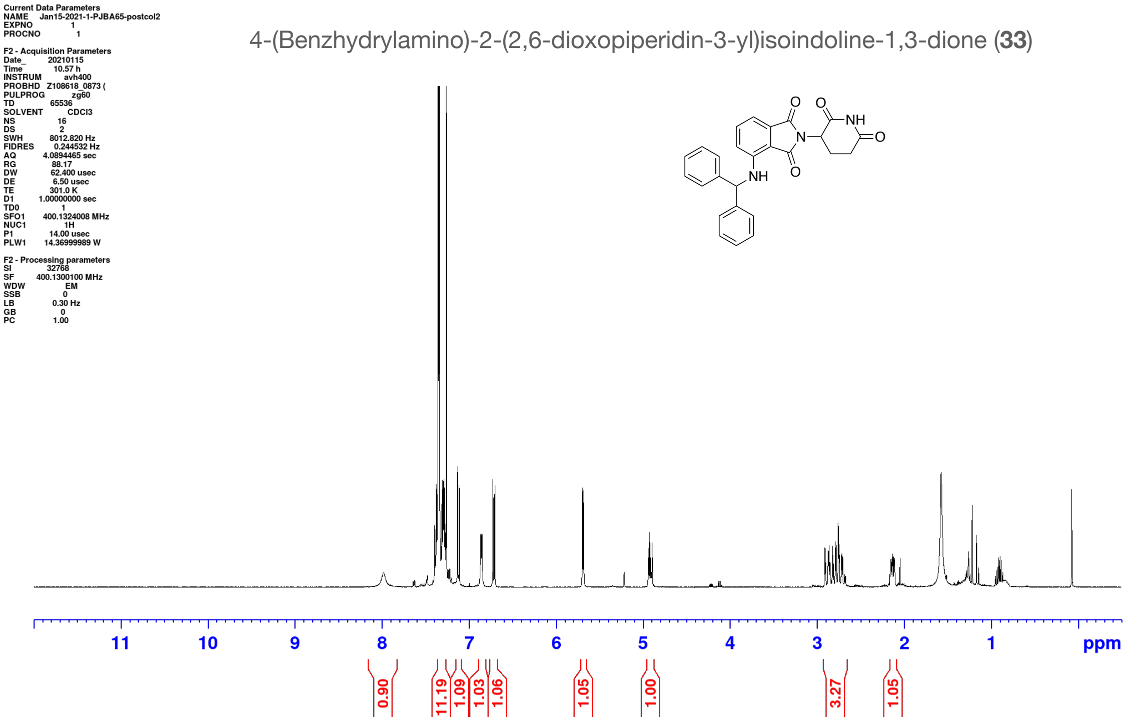

4-(Benzhydrylamino)-2-(2,6-dioxopiperidin-3-yl)isoindoline-1,3-dione (**33**)

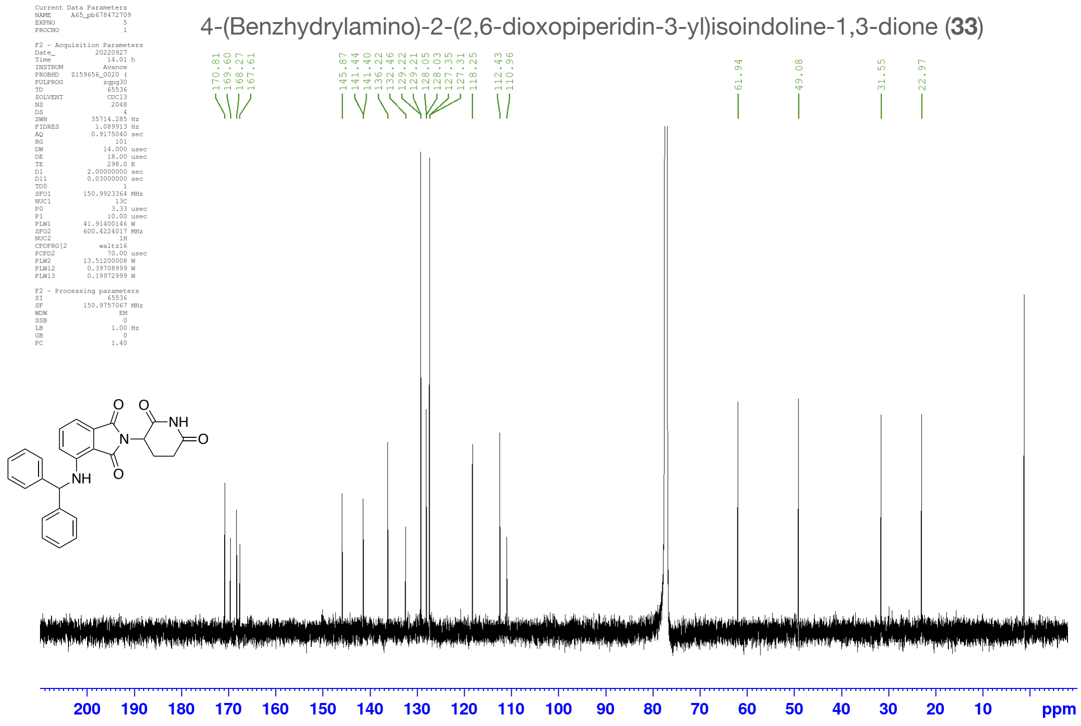

Current Data Parameters
NAME Sep26-2022-1-PJBA83
EXPNO 1
PROCNO 1

F2 - Acquisition Parameters
Date_ 20220926
Time 11.11 h
INSTRUM avg400
PROBHD Z8400_0179 (PH
PULPROG zg60
TD 65536
SOLVENT DMSO
NS 16
DS 2
SWH 8012.820 Hz
FIDRES 0.244532 Hz
AQ 4.0894465 sec
RG 206.87
DW 62.400 usec
DE 6.50 usec
TE 293.4 K
D1 1.00000000 sec
TD0 1
SFO1 400.2024012 MHz
NUC1 1H
P1 11.00 usec
PLW1 14.00000000 W

F2 - Processing parameters
SI 32768
SF 400.2000031 MHz
WDW EM
SSB 0
LB 0.30 Hz
GB 0
PC 1.00

4-(Benzhydryloxy)-2-(2,6-dioxopiperidin-3-yl)isoindoline-1,3-dione (**34**)

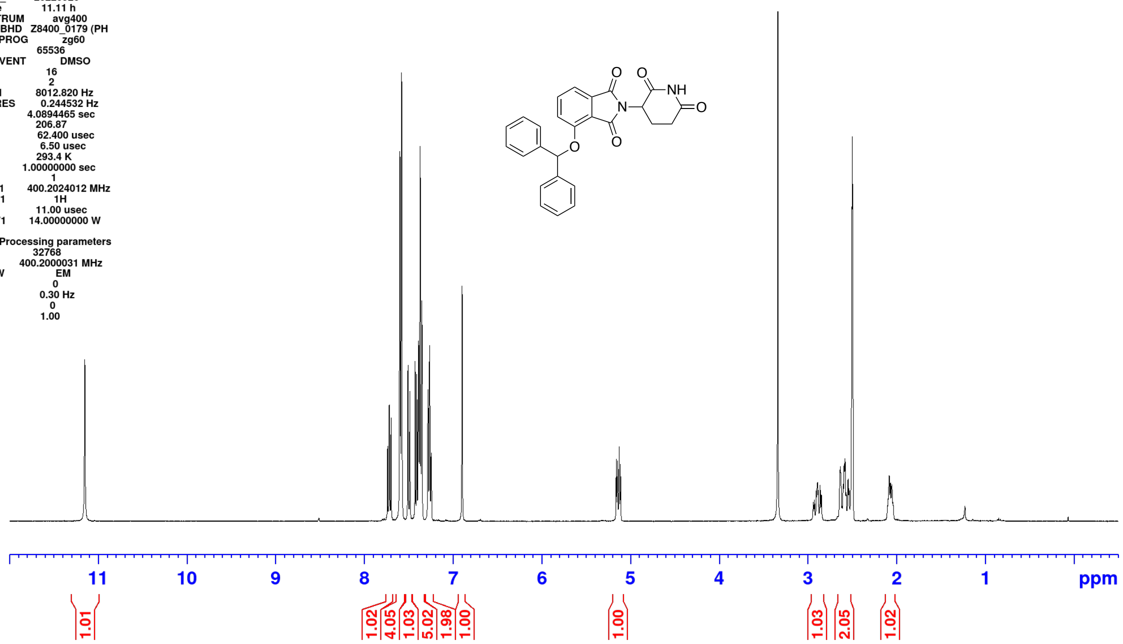

Current Data Parameters
NAME A83_pb678442609
EXPNO 5
PROCNO 1

F2 - Acquisition Parameters
Date_ 20220927
Time 5.30 h
INSTRUM Avance
PROBHD Z159656_0020 (
PULPROG zgpg30
TD 65536
SOLVENT DMSO
NS 512
DS 4
SWH 35714.285 Hz
FIDRES 1.089913 Hz
AQ 0.9175040 sec
RG 101
DW 14.000 usec
DE 18.00 usec
TE 298.0 K
D1 2.00000000 sec
D11 0.03000000 sec
TD0 1
SFO1 150.9923364 MHz
NUC1 13C
P0 3.33 usec
P1 10.00 usec
PLW1 41.91400146 W
SFO2 600.4224017 MHz
NUC2 1H
CPDPRG[2 waltz16
PCPD2 70.00 usec
PLW2 13.51200008 W
PLW12 0.39708999 W
PLW13 0.19972999 W

F2 - Processing parameters
SI 65536
SF 150.9758037 MHz
WDW EM
SSB 0
LB 1.00 Hz
GB 0
PC 1.40

4-(Benzhydryloxy)-2-(2,6-dioxopiperidin-3-yl)isoindoline-1,3-dione (**34**)

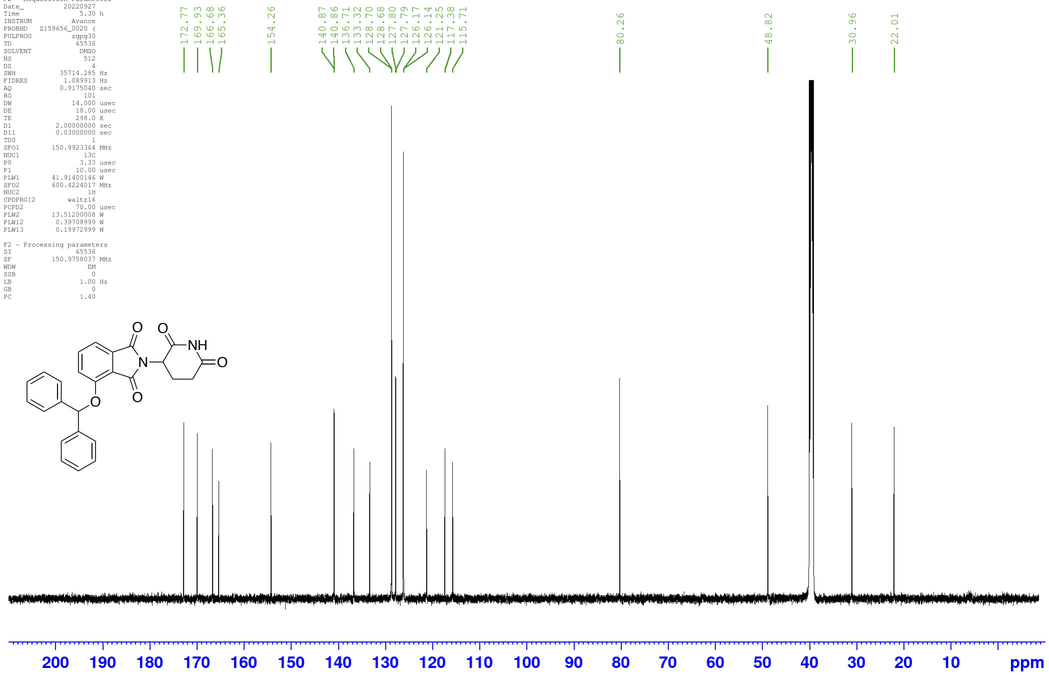

Current Data Parameters
NAME Sep20-2022-1-PJBA66
EXPNO 1
PROCNO 1

F2 - Acquisition Parameters
Date_ 20220920
Time 11.44 h
INSTRUM avg400
PROBHD Z8400_0179 (PH
PULPROG zg60
TD 65536
SOLVENT DMSO
NS 16
DS 2
SWH 8012.820 Hz
FIDRES 0.244532 Hz
AQ 4.0894465 sec
RG 206.87
DW 62.400 usec
DE 6.50 usec
TE 294.0 K
D1 1.00000000 sec
TD0 1
SFO1 400.2024012 MHz
NUC1 1H
P1 11.00 usec
PLW1 14.00000000 W

F2 - Processing parameters
SI 32768
SF 400.2000036 MHz
WDW EM
SSB 0
LB 0.30 Hz
GB 0
PC 1.00

6-(2,6-Dioxopiperidin-3-yl)-5H-pyrrolo[3,4-b]pyridine-5,7(6H)-dione (**35**)

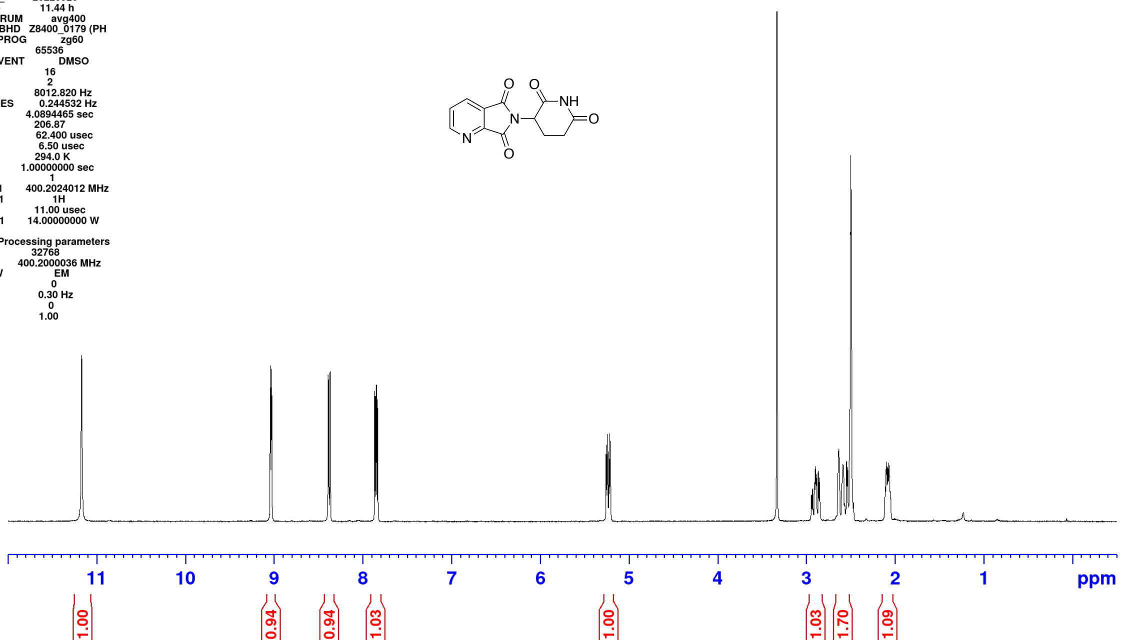

Current Data Parameters
NAME Sep20-2022-1-PJBA74
EXPNO 1
PROCNO 1

F2 - Acquisition Parameters
Date_ 20220920
Time 11.51 h
INSTRUM avg400
PROBHD Z8400_0179 (PH
PULPROG zg60
TD 65536
SOLVENT DMSO
NS 16
DS 2
SWH 8012.820 Hz
FIDRES 0.244532 Hz
AQ 4.0894465 sec
RG 206.87
DW 62.400 usec
DE 6.50 usec
TE 293.7 K
D1 1.00000000 sec
TD0 1
SFO1 400.2024012 MHz
NUC1 1H
P1 11.00 usec
PLW1 14.00000000 W

F2 - Processing parameters
SI 32768
SF 400.2000035 MHz
WDW EM
SSB 0
LB 0.30 Hz
GB 0
PC 1.00

2-(2,6-Dioxopiperidin-3-yl)-1H-pyrrolo[3,4-c]pyridine-1,3(2H)-dione (**36**)

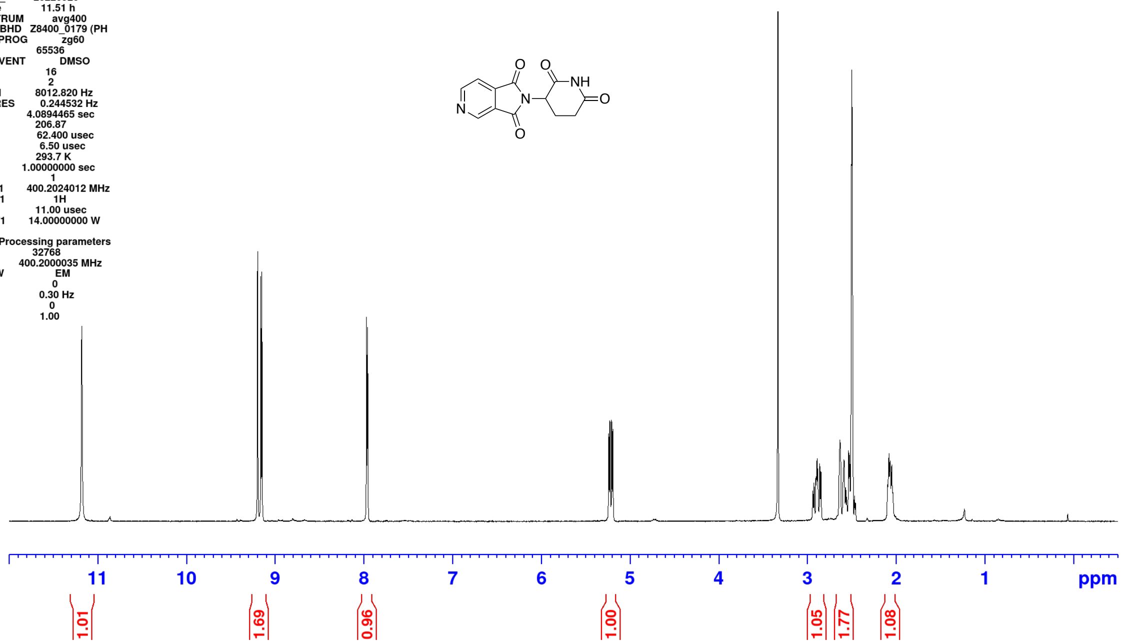

HPLC Traces

2-(2,6-Dioxopiperidin-3-yl)isoindoline-1,3-dione **(1)**

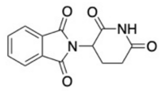

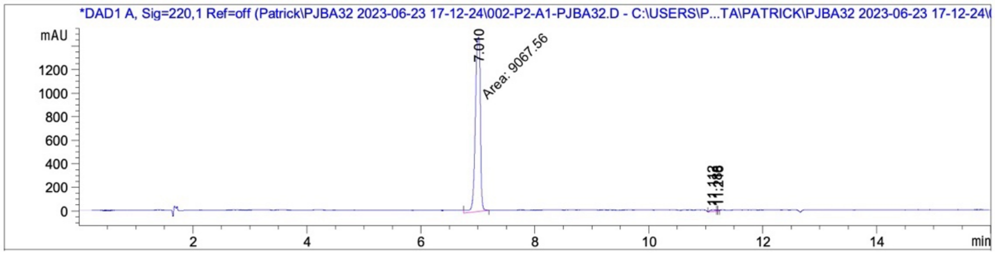

Peak #	RetTime [min]	Type	Width [min]	Area [mAU*s]	Height [mAU]	Area %
1	7.010	MM	0.1021	9067.55762	1480.10632	98.7473
2	11.112	BV	0.0775	89.24358	13.60625	0.9719
3	11.188	VV	0.0158	11.46414	9.67118	0.1248
4	11.216	VV	0.0207	14.32457	8.69674	0.1560

Totals : 9182.58990 1512.08049

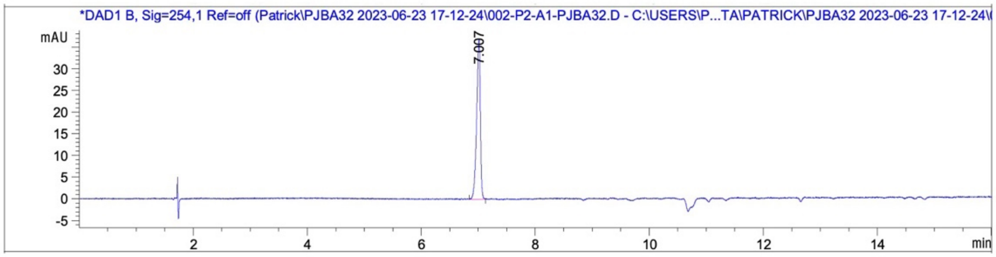

Peak #	RetTime [min]	Type	Width [min]	Area [mAU*s]	Height [mAU]	Area %
1	7.007	BB	0.0659	165.96967	36.92858	100.0000

Totals : 165.96967 36.92858

4-Amino-2-(2,6-dioxopiperidin-3-yl)isoindoline-1,3-dione **(2)**

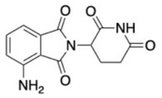

PJB_pom_220 : Injection 1

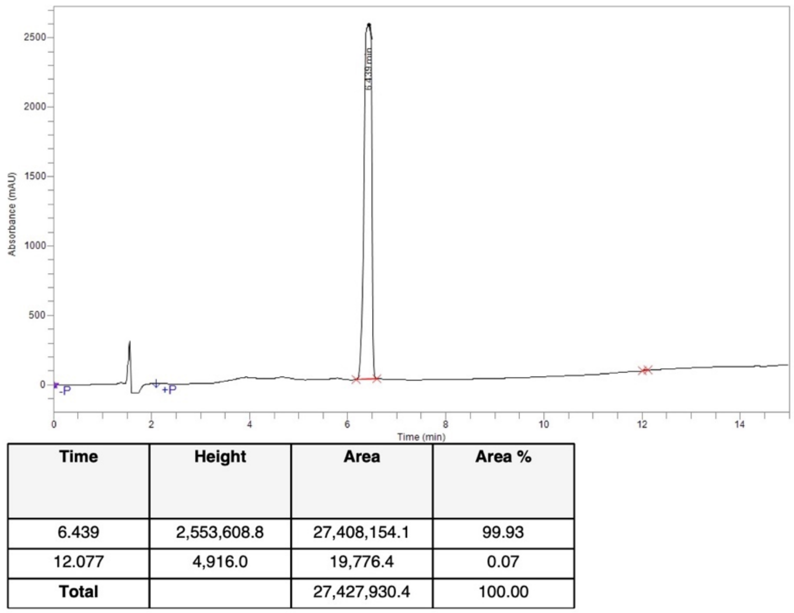

Time	Height	Area	Area %
6.439	2,553,608.8	27,408,154.1	99.93
12.077	4,916.0	19,776.4	0.07
**Total**		27,427,930.4	100.00

PJB_pom_254 : Injection 1

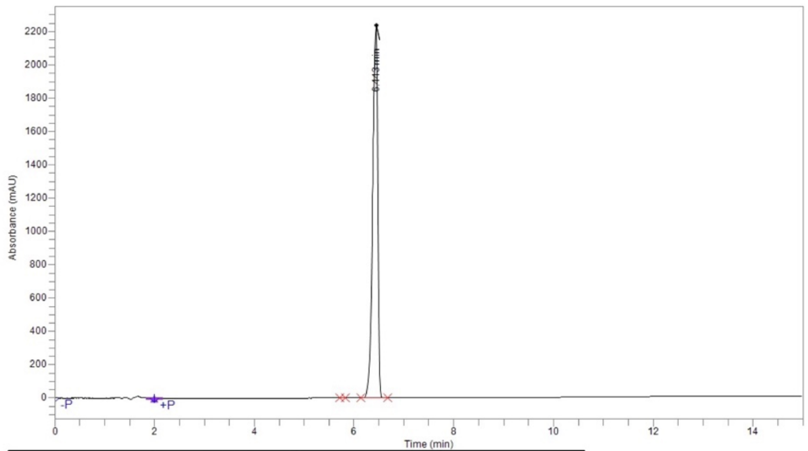

Time	Height	Area	Area %
5.789	340.4	1,487.8	0.01
6.443	2,239,247.0	15,788,772.2	99.99
**Total**		15,790,260.0	100.00

5-Amino-2-(2,6-dioxopiperidin-3-yl)isoindoline-1,3-dione **(3)**

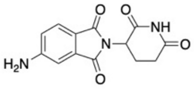

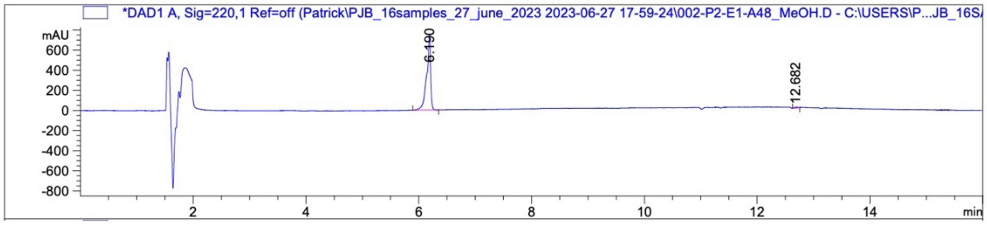

Peak #	RetTime [min]	Type	Width [min]	Area [mAU*s]	Height [mAU]	Area %
1	6.190	BB	0.0657	3589.78223	744.89728	98.7363
2	12.682	BV	0.0706	45.94411	7.98587	1.2637

Totals : 3635.72633 752.88315

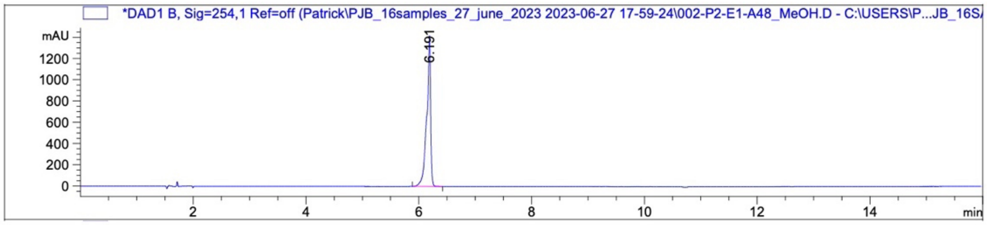

Peak #	RetTime [min]	Type	Width [min]	Area [mAU*s]	Height [mAU]	Area %
1	6.191	BB	0.0653	6732.34521	1419.69299	100.0000

Totals : 6732.34521 1419.69299

2-(2,6-Dioxopiperidin-3-yl)-4-hydroxyisoindoline-1,3-dione **(4)**

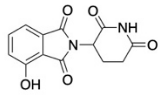

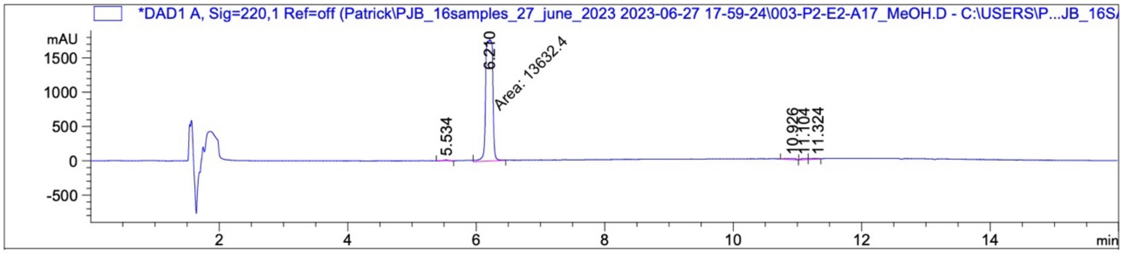

Peak #	RetTime [min]	Type	Width [min]	Area [mAU*s]	Height [mAU]	Area %
1	5.534	BB	0.0646	65.99696	12.70768	0.4697
2	6.210	MM	0.1278	1.36324e4	1777.73010	97.0149
3	10.926	BB	0.1220	141.60933	13.88067	1.0078
4	11.104	BV	0.0815	105.82807	15.42453	0.7531
5	11.324	VV	0.1264	106.02243	9.86394	0.7545
Totals :				1.40519e4	1829.60693	

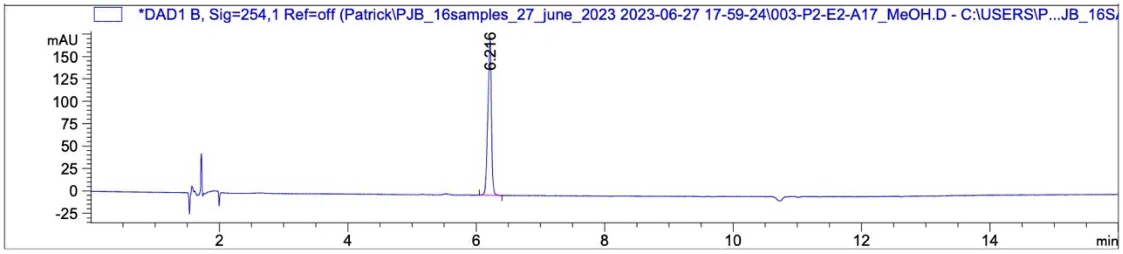

Peak #	RetTime [min]	Type	Width [min]	Area [mAU*s]	Height [mAU]	Area %
1	6.216	BB	0.0554	675.15735	173.32211	100.0000
Totals :				675.15735	173.32211	

2-(2,6-Dioxopiperidin-3-yl)-5-hydroxyisoindoline-1,3-dione **(5)**

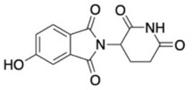

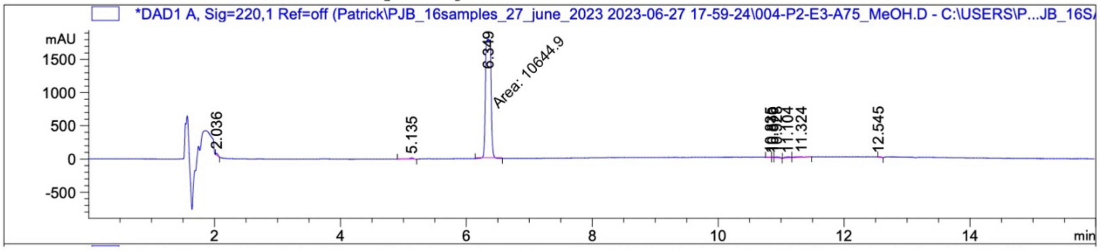

Peak #	RetTime [min]	Type	Width [min]	Area [mAU*s]	Height [mAU]	Area %
1	2.036	BB	0.0318	63.73929	32.55661	0.5708
2	5.135	BB	0.0519	80.56284	20.23950	0.7214
3	6.349	MM	0.0991	1.06449e4	1791.00110	95.3208
4	10.835	BV	0.0522	27.88848	7.70735	0.2497
5	10.870	VV	0.0245	18.61944	9.87828	0.1667
6	10.928	VB	0.0819	87.11492	13.66248	0.7801
7	11.104	BV	0.0830	105.74609	15.13966	0.9469
8	11.324	VV R	0.1459	110.80653	9.01607	0.9922
9	12.545	VB	0.0465	28.06925	7.27520	0.2513
Totals :				1.11674e4	1906.47626	

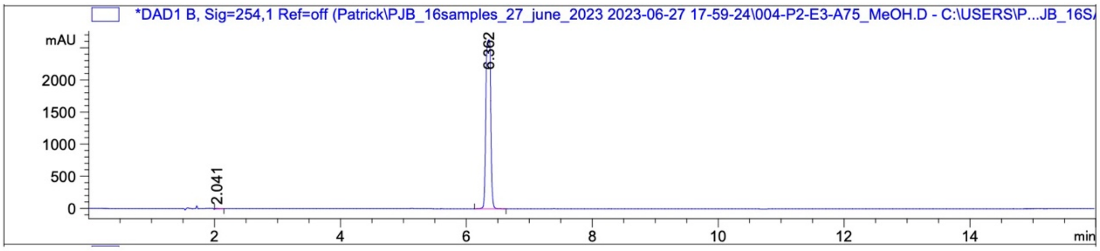

Peak #	RetTime [min]	Type	Width [min]	Area [mAU*s]	Height [mAU]	Area %
1	2.041	BB	0.0587	47.90619	10.50316	0.3428
2	6.362	BB	0.0638	1.39278e4	2630.26196	99.6572
Totals :				1.39757e4	2640.76512	

2-(2,6-Dioxopiperidin-3-yl)-4-methoxyisoindoline-1,3-dione **(6)**

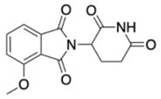

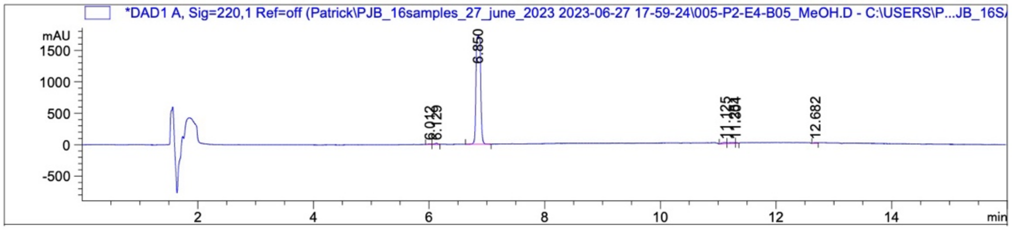

Peak #	RetTime [min]	Type	Width [min]	Area [mAU*s]	Height [mAU]	Area %
1	6.012	BV	0.0440	25.54466	7.36038	0.2574
2	6.129	VB	0.0474	57.92704	15.93125	0.5838
3	6.850	BB	0.0661	9592.31934	1717.97754	96.6734
4	11.125	BV	0.0756	93.57810	14.94160	0.9431
5	11.281	VV	0.1129	89.21191	9.51512	0.8991
6	11.304	VB	0.0268	18.89025	8.87971	0.1904
7	12.682	BV	0.0587	44.93058	9.40365	0.4528
Totals :				9922.40189	1784.00926	

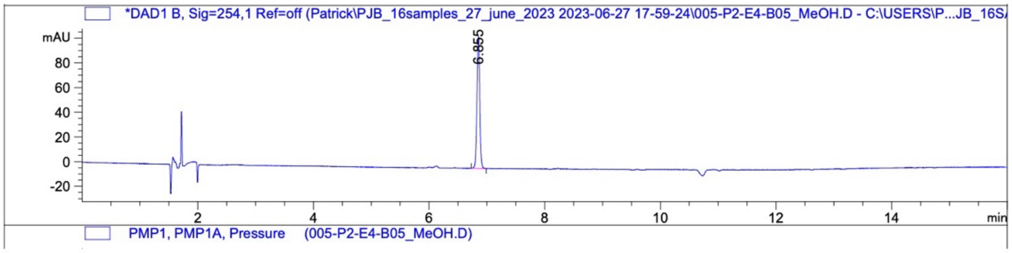

Signal 2: DAD1 B, Sig=254,1 Ref=off
Signal has been modified after loading from rawdata file!

Peak #	RetTime [min]	Type	Width [min]	Area [mAU*s]	Height [mAU]	Area %
1	6.855	BB	0.0527	358.38605	106.73189	100.0000
Totals :				358.38605	106.73189	

2-(2,6-Dioxopiperidin-3-yl)-5-methoxyisoindoline-1,3-dione **(7)**

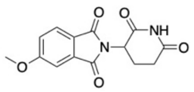

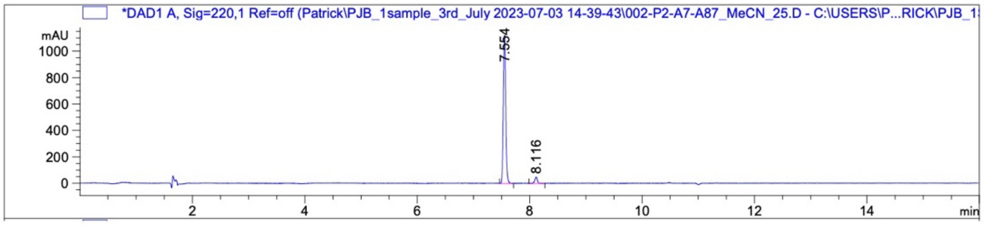

Peak #	RetTime [min]	Type	Width [min]	Area [mAU*s]	Height [mAU]	Area %
1	7.554	VB R	0.0463	3345.32910	1122.03931	95.5459
2	8.116	BB	0.0463	155.95166	48.19432	4.4541

Totals : 3501.28076 1170.23363

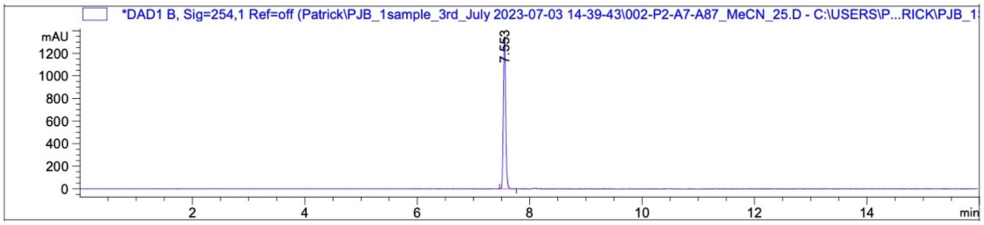

Peak #	RetTime [min]	Type	Width [min]	Area [mAU*s]	Height [mAU]	Area %
1	7.553	BB	0.0436	3819.21582	1347.94446	100.0000

Totals : 3819.21582 1347.94446

2-(2,6-Dioxopiperidin-3-yl)-4-fluoroisoindoline-1,3-dione **(8)**

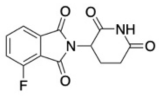

PJBA16_220 : Injection 1

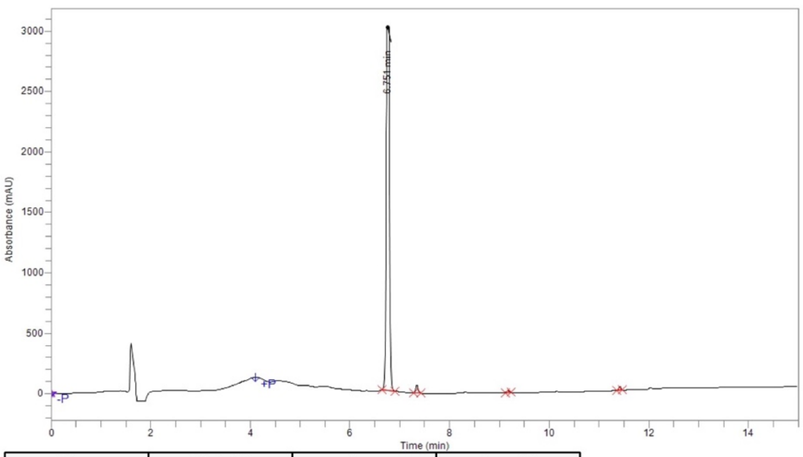

Time	Height	Area	Area %
6.751	3,028,627.9	14,117,851.2	97.52
7.346	63,271.4	219,745.1	1.52
9.173	16,466.0	52,777.1	0.36
11.416	28,380.4	86,527.3	0.60
**Total**		14,476,900.7	100.00

PJBA16_254 : Injection 1

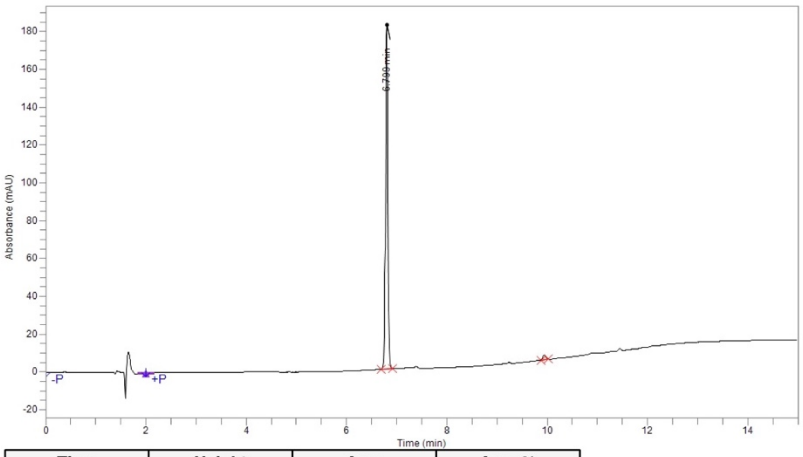

Time	Height	Area	Area %
6.799	182,217.2	630,377.0	98.51
9.945	2,444.7	9,551.0	1.49
**Total**		639,928.0	100.00

2-(2,6-Dioxopiperidin-3-yl)-5-fluoroisoindoline-1,3-dione **(9)**

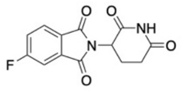

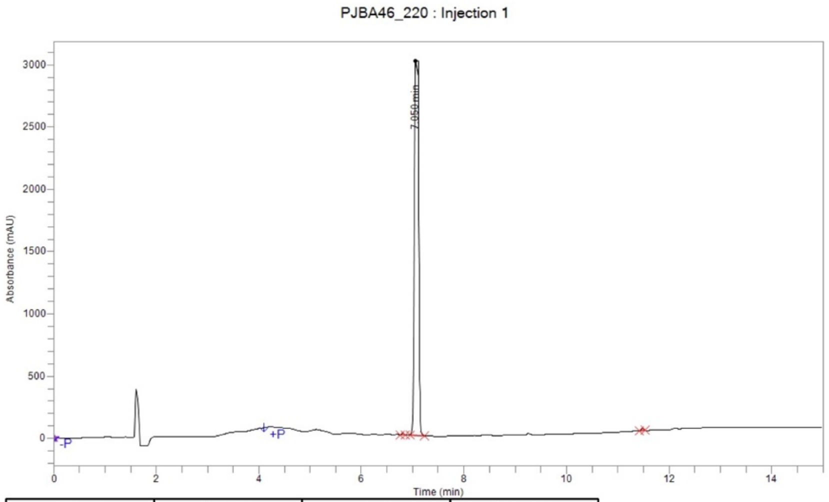

Time	Height	Area	Area %
6.809	13,303.9	39,412.8	0.22
7.050	3,039,466.0	18,002,760.7	99.45
11.482	20,006.3	60,644.8	0.34
**Total**		18,102,818.3	100.00

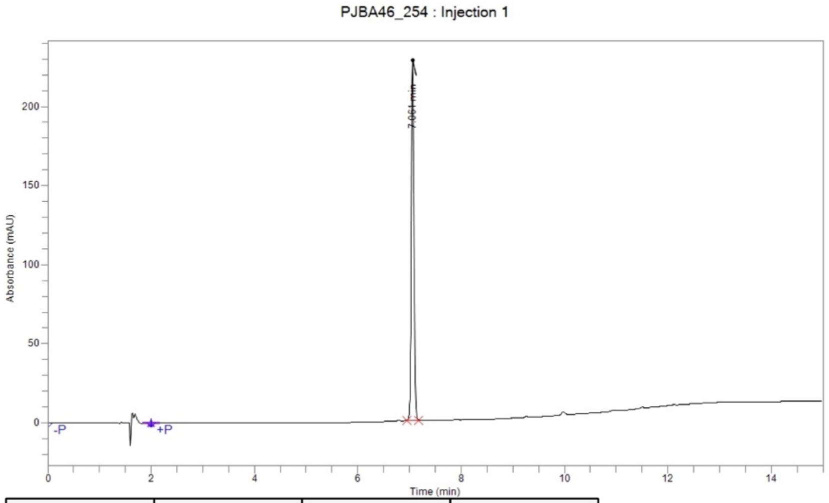

Time	Height	Area	Area %
7.061	227,924.9	796,290.9	100.00
**Total**		796,290.9	100.00

4-Chloro-2-(2,6-dioxopiperidin-3-yl)isoindoline-1,3-dione **(10)**

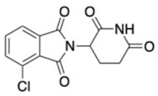

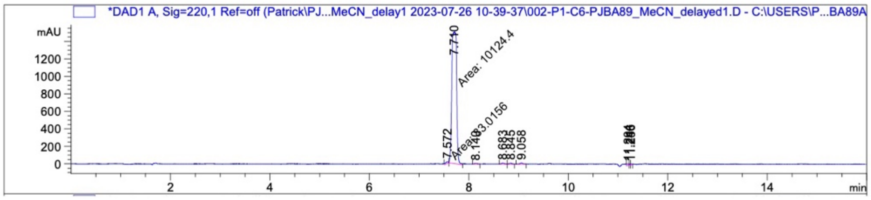

Peak #	RetTime [min]	Type	Width [min]	Area [mAU*s]	Height [mAU]	Area %
1	7.572	MM	0.0716	83.01558	19.31462	0.7948
2	7.710	MM	0.1120	1.01244e4	1506.12683	96.9367
3	8.140	BB	0.0456	28.66743	7.59032	0.2745
4	8.683	BV	0.0456	39.57912	10.96288	0.3790
5	8.845	VB	0.0483	35.57435	10.30676	0.3406
6	9.058	BV R	0.0457	46.73374	12.61280	0.4475
7	11.204	VV	0.0401	47.28849	14.10421	0.4528
8	11.246	VV	0.0244	19.43041	9.87937	0.1860
9	11.256	VV	0.0257	19.65645	9.27698	0.1882

Totals : 1.04443e4 1600.17477

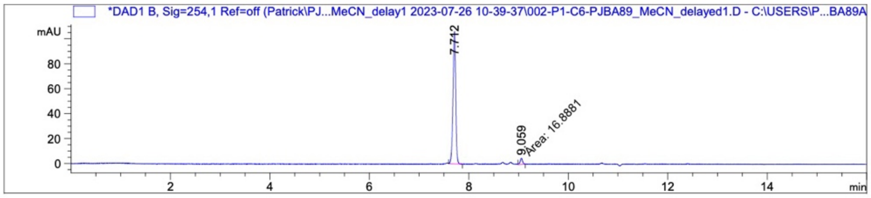

Peak #	RetTime [min]	Type	Width [min]	Area [mAU*s]	Height [mAU]	Area %
1	7.712	BB	0.0580	400.58704	106.22004	95.9547
2	9.059	MM	0.0571	16.88810	4.93309	4.0453

Totals : 417.47514 111.15313

5-Chloro-2-(2,6-dioxopiperidin-3-yl)isoindoline-1,3-dione **(11)**

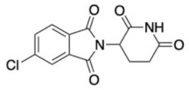

PJBA86_220_actual : Injection 1

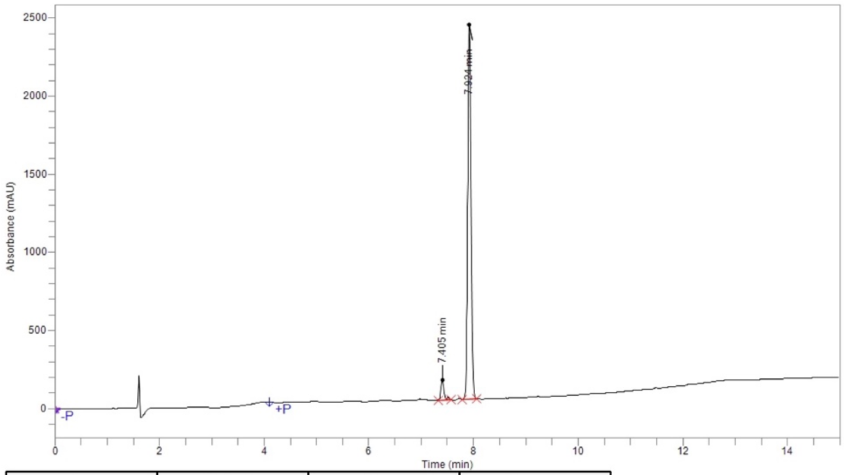

Time	Height	Area	Area %
7.405	129,530.5	482,204.2	4.35
7.518	16,496.3	52,657.9	0.48
7.924	2,400,093.6	10,540,490.2	95.17
**Total**		11,075,352.3	100.00

PJBA86_254 : Injection 1

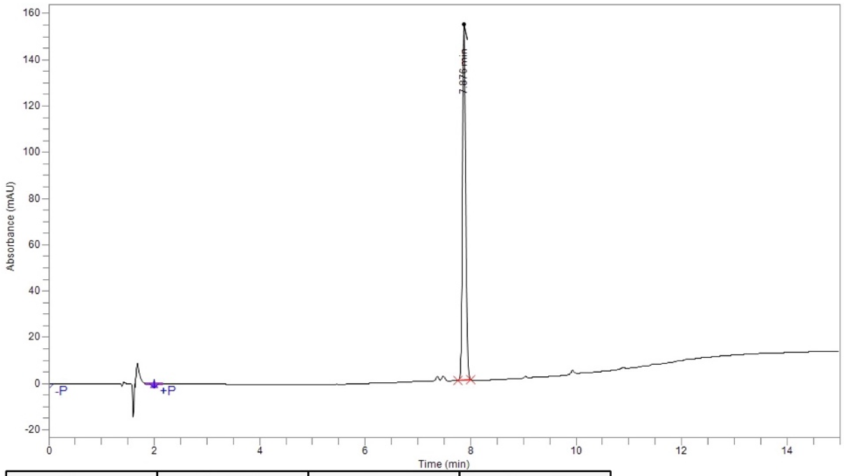

Time	Height	Area	Area %
7.876	153,998.7	642,358.4	100.00
**Total**		642,358.4	100.00

4-Bromo-2-(2,6-dioxopiperidin-3-yl)isoindoline-1,3-dione **(12)**

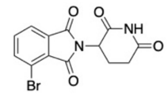

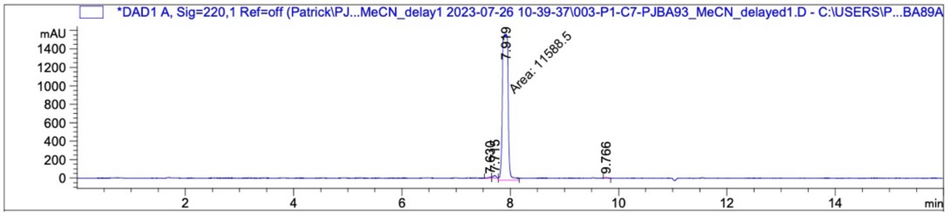

Peak #	RetTime [min]	Type	Width [min]	Area [mAU*s]	Height [mAU]	Area %
1	7.630	BV	0.0488	47.52709	11.97518	0.4039
2	7.715	VB	0.0466	107.22497	28.36489	0.9112
3	7.919	MM	0.1219	1.15885e4	1584.42163	98.4798
4	9.766	BB	0.0395	24.13687	7.50915	0.2051
Totals :				1.17674e4	1632.27086	

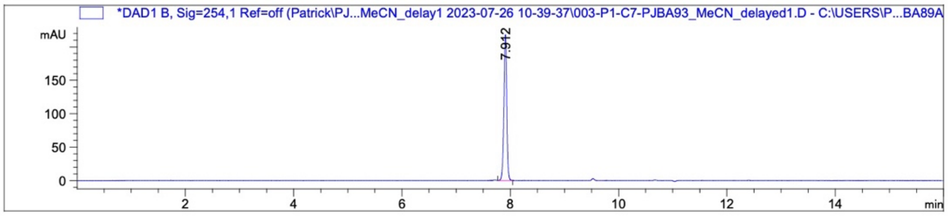

Peak #	RetTime [min]	Type	Width [min]	Area [mAU*s]	Height [mAU]	Area %
1	7.912	BB	0.0561	796.89526	218.08075	100.0000
Totals :				796.89526	218.08075	

5-Bromo-2-(2,6-dioxopiperidin-3-yl)isoindoline-1,3-dione **(13)**

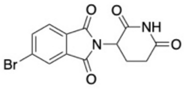

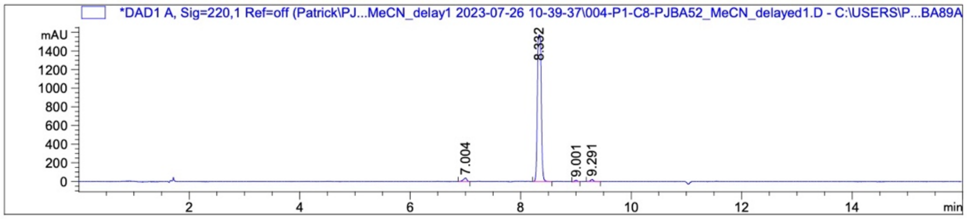

Peak #	RetTime [min]	Type	Width [min]	Area [mAU*s]	Height [mAU]	Area %
1	7.004	VB R	0.0601	169.77148	37.28113	2.1269
2	8.332	BB	0.0589	7684.37988	1575.89148	96.2706
3	9.001	BB	0.0400	47.47767	14.40140	0.5948
4	9.291	BV R	0.0449	80.43294	22.11587	1.0077

Totals : 7982.06198 1649.68988

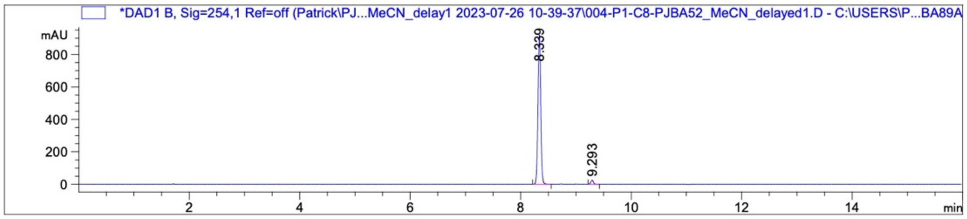

Peak #	RetTime [min]	Type	Width [min]	Area [mAU*s]	Height [mAU]	Area %
1	8.339	BB	0.0550	3232.09131	920.24182	97.5726
2	9.293	BB	0.0479	80.40845	25.42469	2.4274

Totals : 3312.49976 945.66651

2-(2,6-Dioxopiperidin-3-yl)-4-iodoisoindoline-1,3-dione **(14)**

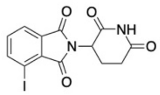

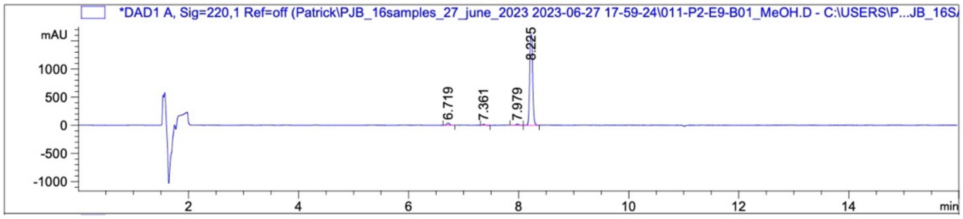

Peak #	RetTime [min]	Type	Width [min]	Area [mAU*s]	Height [mAU]	Area %
1	6.719	BB	0.0445	133.82944	37.15325	2.0950
2	7.361	BB	0.0403	49.76325	15.35464	0.7790
3	7.979	BB	0.0524	77.25655	20.54155	1.2094
4	8.225	BB	0.0468	6127.28809	1613.76160	95.9167

Totals : 6388.13733 1686.81104

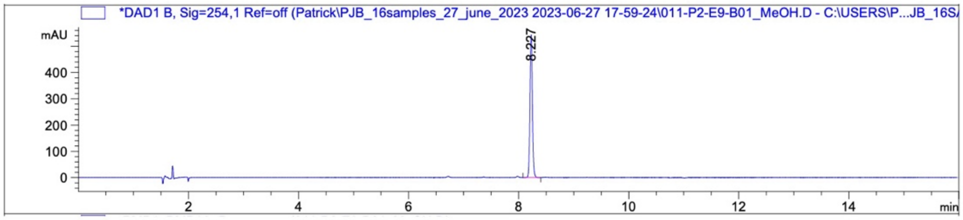

Peak #	RetTime [min]	Type	Width [min]	Area [mAU*s]	Height [mAU]	Area %
1	8.227	BB	0.0480	1676.24133	543.46442	100.0000

Totals : 1676.24133 543.46442

2-(2,6-Dioxopiperidin-3-yl)-5-iodoisoindoline-1,3-dione **(15)**

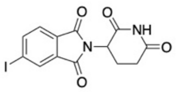

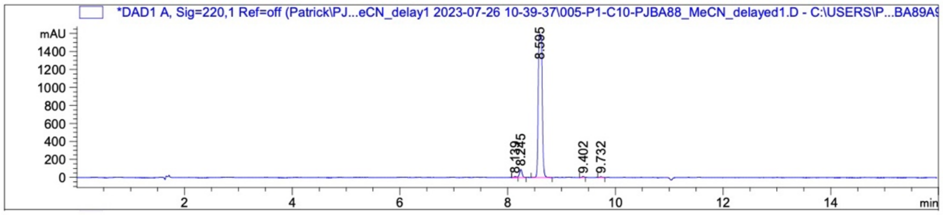

Peak #	RetTime [min]	Type	Width [min]	Area [mAU*s]	Height [mAU]	Area %
1	8.139	BV E	0.0379	31.36179	10.05772	0.3685
2	8.245	VV R	0.0507	303.46432	89.25816	3.5662
3	8.595	VB R	0.0608	8107.98242	1594.07239	95.2812
4	9.402	BV	0.0366	35.78897	11.90493	0.4206
5	9.732	VB	0.0454	30.93200	8.13432	0.3635

Totals : 8509.52951 1713.42752

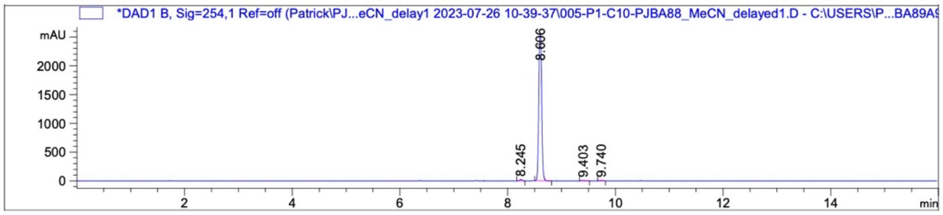

Peak #	RetTime [min]	Type	Width [min]	Area [mAU*s]	Height [mAU]	Area %
1	8.245	BB	0.0493	65.21466	19.61108	0.6621
2	8.606	BB	0.0543	9735.32910	2534.51929	98.8364
3	9.403	BB	0.0462	24.64587	7.71999	0.2502
4	9.740	BB	0.0482	24.74964	7.86426	0.2513

Totals : 9849.93928 2569.71462

2-(2,6-Dioxopiperidin-3-yl)-4-methylisoindoline-1,3-dione **(16)**

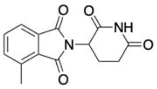

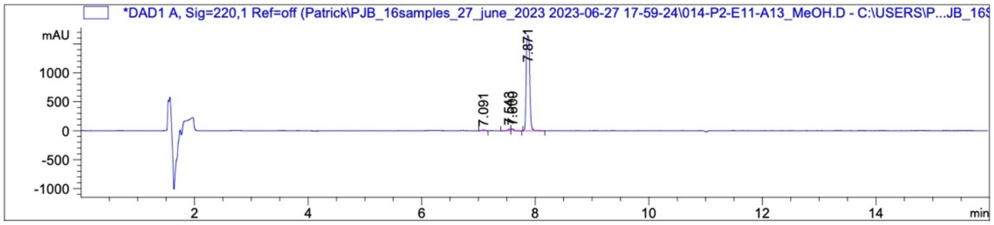

Peak #	RetTime [min]	Type	Width [min]	Area [mAU*s]	Height [mAU]	Area %
1	7.091	BB	0.0601	48.40011	12.24416	0.6610
2	7.543	BV	0.0466	84.10289	26.80878	1.1486
3	7.600	VB	0.0487	106.54169	32.09800	1.4550
4	7.871	BB	0.0705	7083.42627	1636.31177	96.7355
Totals :				7322.47095	1707.46271	

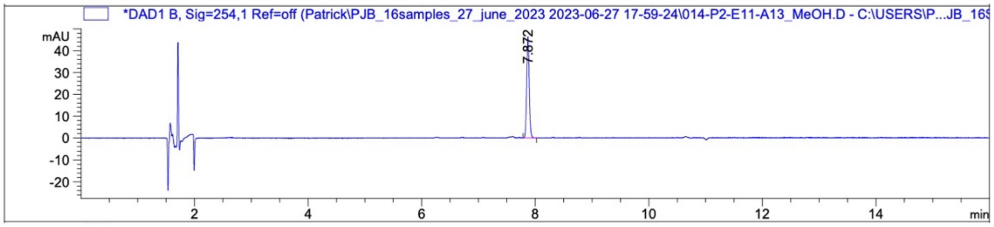

Peak #	RetTime [min]	Type	Width [min]	Area [mAU*s]	Height [mAU]	Area %
1	7.872	BB	0.0472	144.07738	46.43835	100.0000
Totals :				144.07738	46.43835	

2-(2,6-Dioxopiperidin-3-yl)-5-methylisoindoline-1,3-dione **(17)**

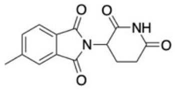

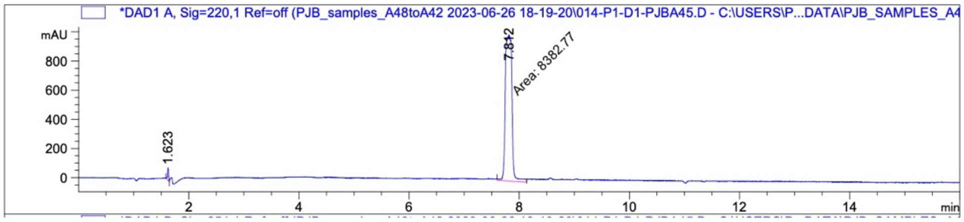

Peak #	RetTime [min]	Type	Width [min]	Area [mAU*s]	Height [mAU]	Area %
1	1.623	BB	0.0208	108.92145	85.71591	1.2827
2	7.812	MM	0.1389	8382.77344	1005.74152	98.7173

Totals : 8491.69489 1091.45743

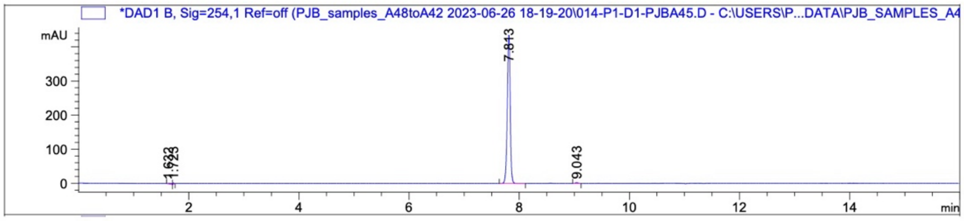

Peak #	RetTime [min]	Type	Width [min]	Area [mAU*s]	Height [mAU]	Area %
1	1.632	BB	0.0811	11.08154	1.72119	0.6859
2	1.723	BB	0.0229	5.64052	3.67605	0.3491
3	7.813	BB	0.0567	1589.70142	434.66150	98.3937
4	9.043	BB	0.0501	9.23091	2.82392	0.5713

Totals : 1615.65439 442.88267

2-(2,6-Dioxopiperidin-3-yl)-5-(trifluoromethyl)isoindoline-1,3-dione **(18)**

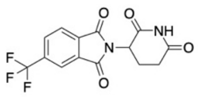

A84_220_12_7_2021 : Injection 1

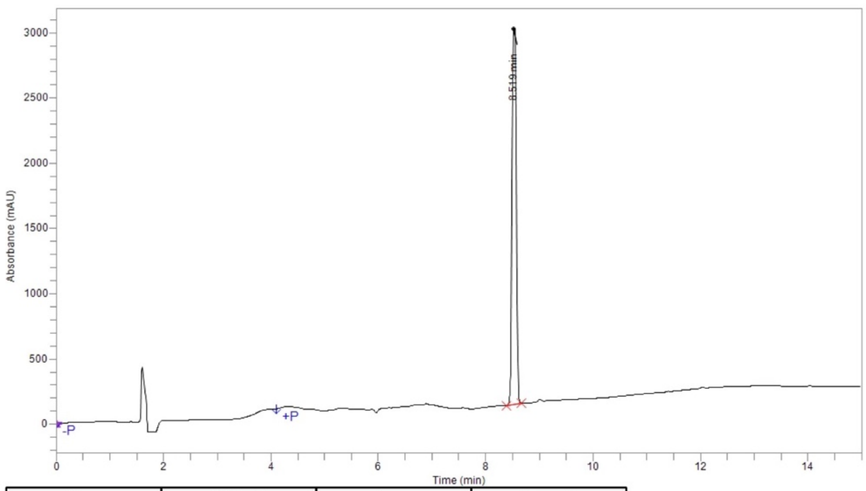

Time	Height	Area	Area %
8.519	2,921,498.9	15,168,796.2	100.00
**Total**		15,168,796.2	100.00

A84_254_12_7_2021 : Injection 1

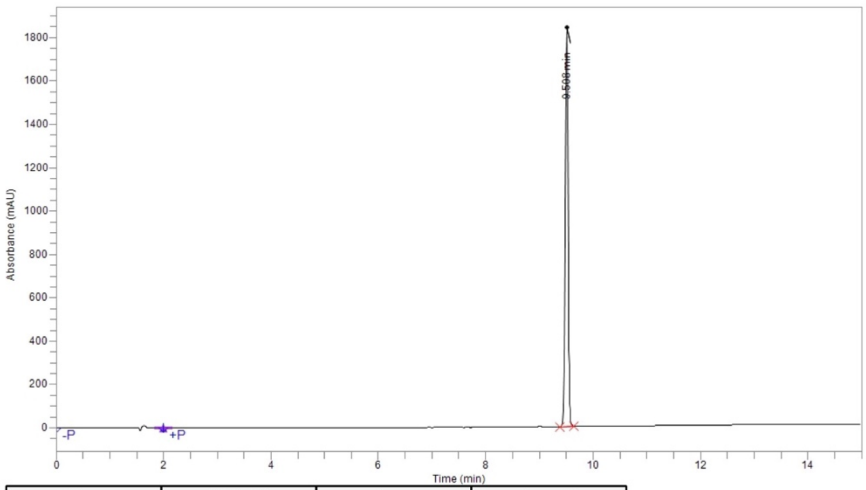

Time	Height	Area	Area %
9.508	1,844,947.1	6,668,456.4	100.00
**Total**		6,668,456.4	100.00

5-(Tert-butyl)-2-(2,6-dioxopiperidin-3-yl)isoindoline-1,3-dione **(19)**

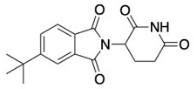

PJB5tBu_220_3_2_2021 : Injection 1

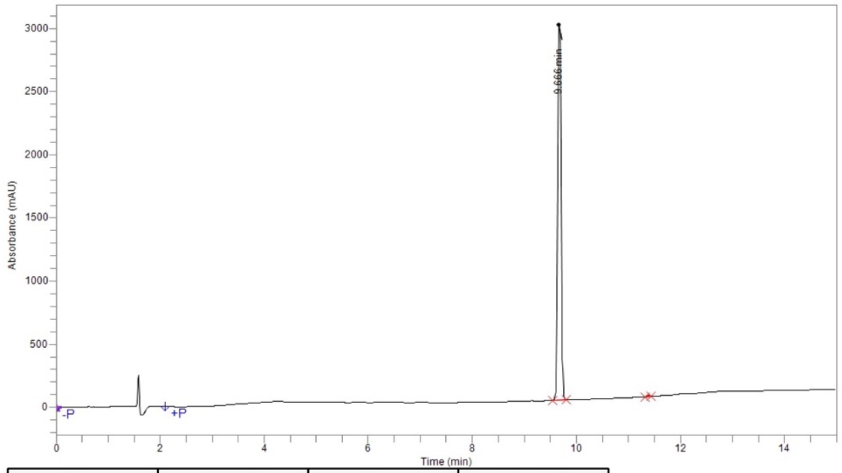

Time	Height	Area	Area %
9.666	3,015,607.0	13,255,833.1	99.79
11.388	8,856.5	28,068.3	0.21
**Total**		13,283,901.5	100.00

PJB5tBu_254_3_2_2021 : Injection 1

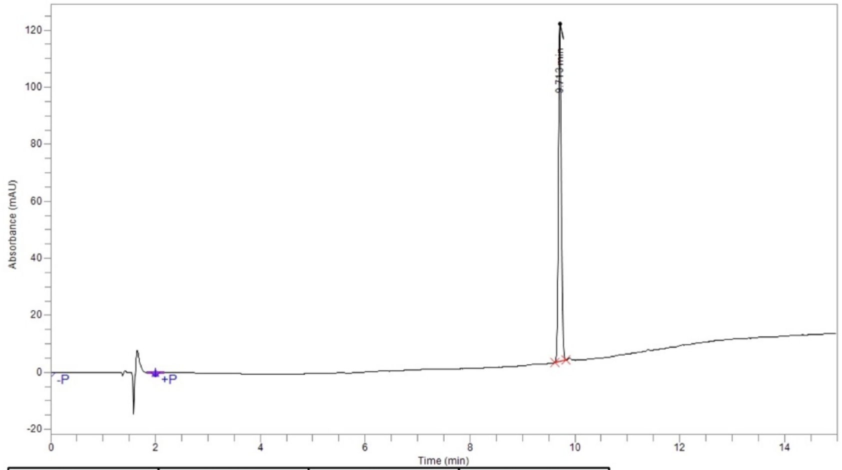

Time	Height	Area	Area %
9.713	118,463.5	453,471.6	100.00
**Total**		453,471.6	100.00

2-(2,6-Dioxopiperidin-3-yl)-5-phenylisoindoline-1,3-dione **(20)**

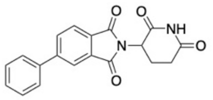

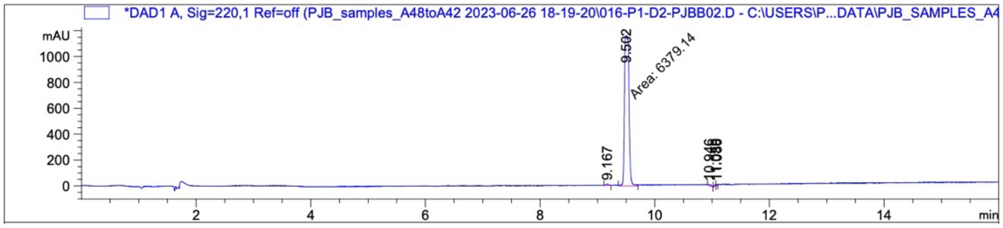

Peak #	RetTime [min]	Type	Width [min]	Area [mAU*s]	Height [mAU]	Area %
1	9.167	VV	0.0397	38.47590	12.08027	0.5924
2	9.502	MM	0.0916	6379.13721	1160.77014	98.2165
3	10.946	VB	0.0532	41.28960	10.78413	0.6357
4	11.059	BV	0.0189	16.44551	11.05400	0.2532
5	11.086	VV	0.0242	19.62358	10.30767	0.3021
Totals :				6494.97180	1204.99620	

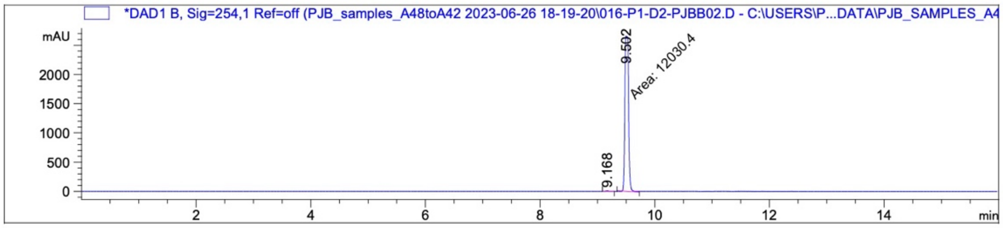

Peak #	RetTime [min]	Type	Width [min]	Area [mAU*s]	Height [mAU]	Area %
1	9.168	BB	0.0474	32.15612	10.45554	0.2666
2	9.502	MM	0.0756	1.20304e4	2653.79126	99.7334
Totals :				1.20626e4	2664.24680	

5-(Dimethylamino)-2-(2,6-dioxopiperidin-3-yl)isoindoline-1,3-dione **(21)**

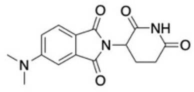

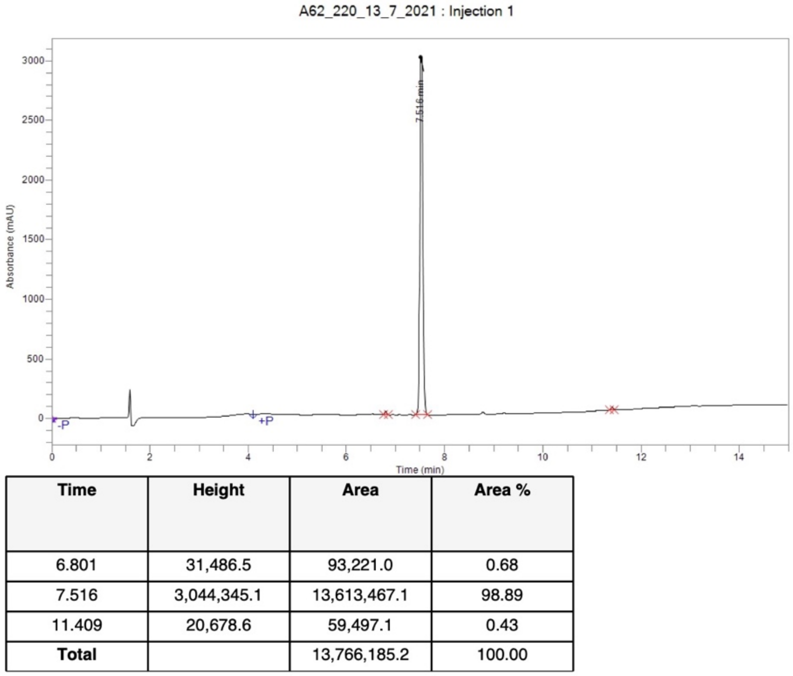

Time	Height	Area	Area %
6.801	31,486.5	93,221.0	0.68
7.516	3,044,345.1	13,613,467.1	98.89
11.409	20,678.6	59,497.1	0.43
**Total**		13,766,185.2	100.00

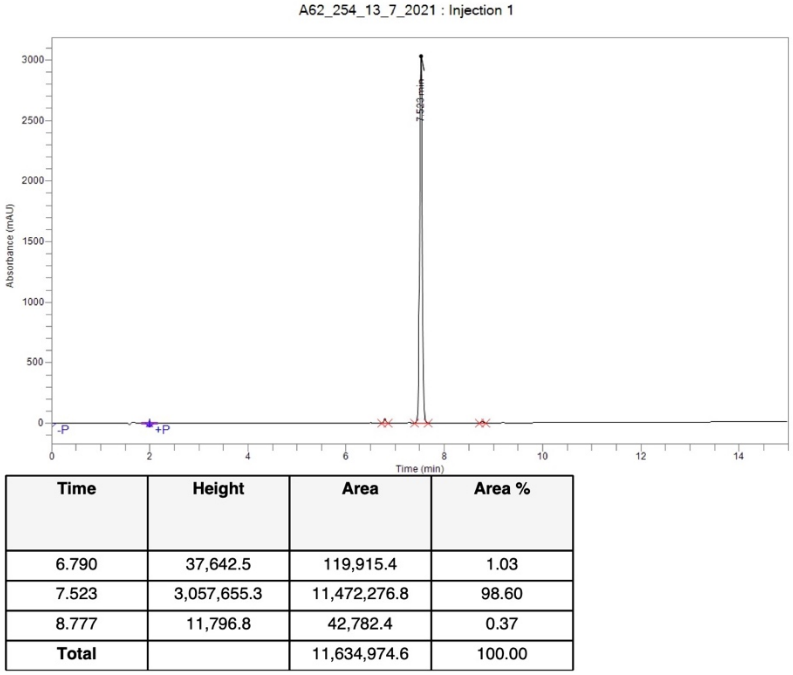

Time	Height	Area	Area %
6.790	37,642.5	119,915.4	1.03
7.523	3,057,655.3	11,472,276.8	98.60
8.777	11,796.8	42,782.4	0.37
**Total**		11,634,974.6	100.00

2-(2,6-Dioxopiperidin-3-yl)-5-morpholinoisoindoline-1,3-dione **(22)**

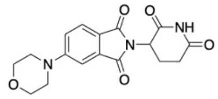

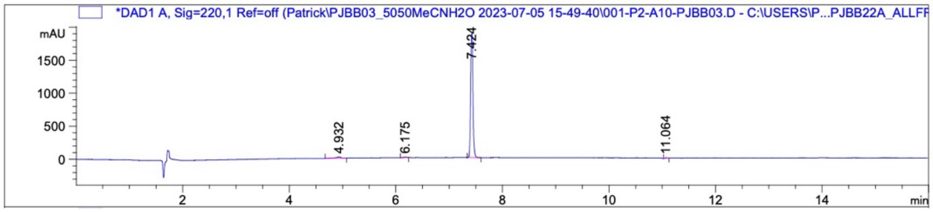

Peak #	RetTime [min]	Type	Width [min]	Area [mAU*s]	Height [mAU]	Area %
1	4.932	BB	0.0854	133.68457	19.52182	2.2990
2	6.175	BB	0.0472	36.72113	10.41101	0.6315
3	7.424	BB	0.0469	5612.18750	1878.12195	96.5140
4	11.064	BB	0.0438	32.30088	10.53894	0.5555
Totals :				5814.89408	1918.59372	

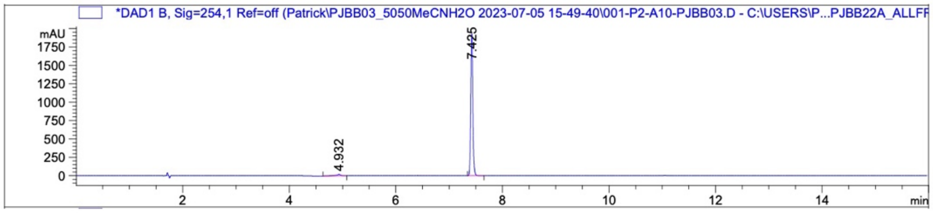

Peak #	RetTime [min]	Type	Width [min]	Area [mAU*s]	Height [mAU]	Area %
1	4.932	BB	0.0914	132.48828	19.23407	2.5003
2	7.425	BB	0.0413	5166.39648	1927.11658	97.4997
Totals :				5298.88477	1946.35064	

2-(2,6-Dioxopiperidin-3-yl)-5-(4-methylpiperazin-1-yl)isoindoline-1,3-dione **(23)**

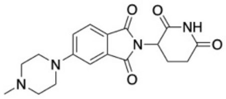

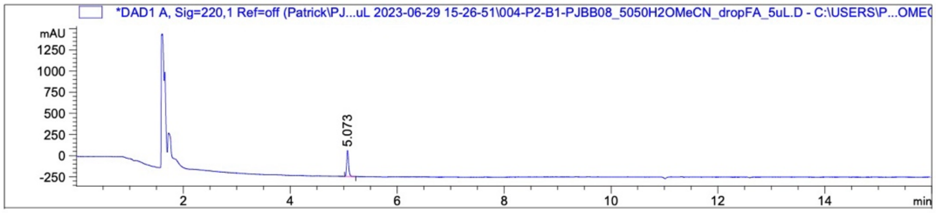

Peak #	RetTime [min]	Type	Width [min]	Area [mAU*s]	Height [mAU]	Area %
1	5.073	BB	0.0382	760.88721	309.12369	100.0000
Totals :				760.88721	309.12369	

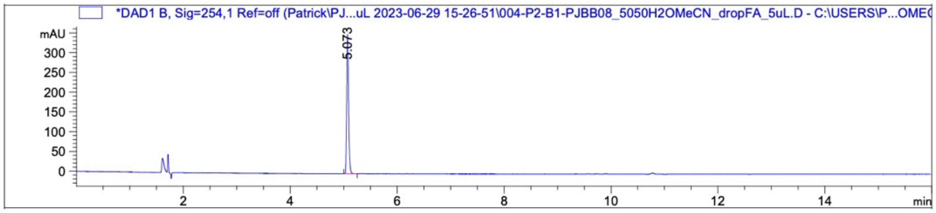

Peak #	RetTime [min]	Type	Width [min]	Area [mAU*s]	Height [mAU]	Area %
1	5.073	BB	0.0380	864.64014	353.40967	100.0000
Totals :				864.64014	353.40967	

2-(2,6-Dioxopiperidin-3-yl)-4-(ethylamino)isoindoline-1,3-dione **(24)**

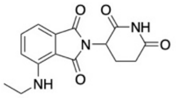

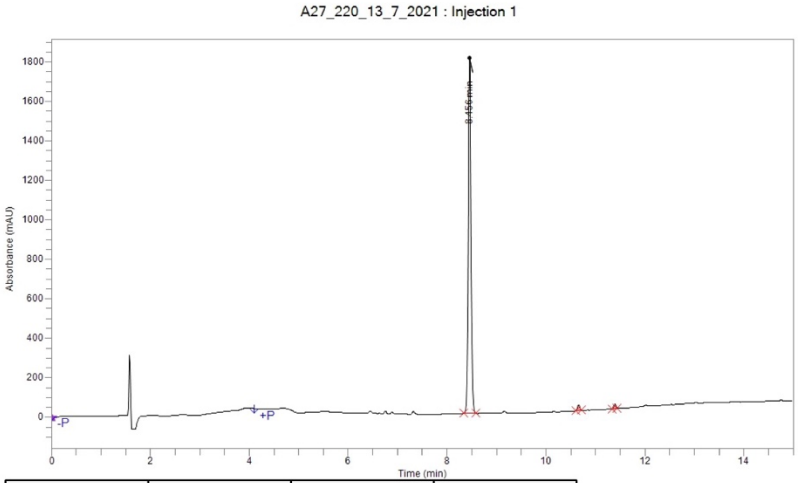

Time	Height	Area	Area %
8.456	1,800,428.2	7,219,094.3	97.92
10.660	27,073.5	88,235.7	1.20
11.390	22,352.5	65,484.8	0.89
**Total**		7,372,814.8	100.00

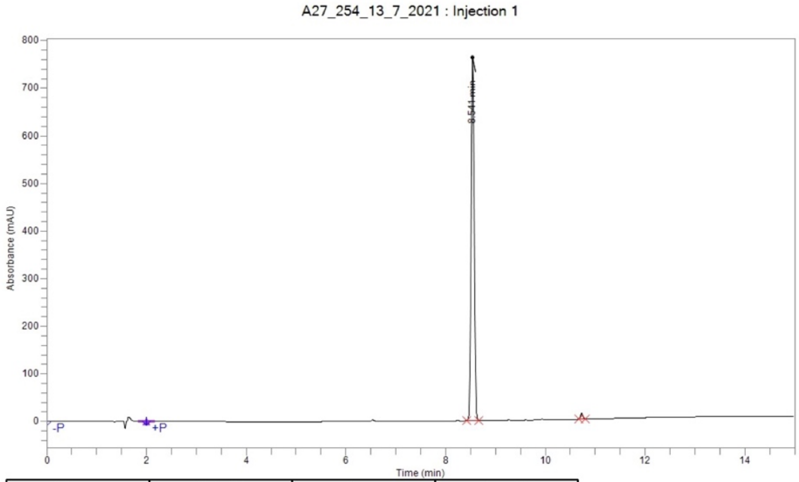

Time	Height	Area	Area %
8.541	763,584.8	3,012,532.3	98.61
10.731	12,178.1	42,392.6	1.39
**Total**		3,054,924.9	100.00

2-(2,6-Dioxopiperidin-3-yl)-4-(prop-2-yn-1-ylamino)isoindoline-1,3-dione **(25)**

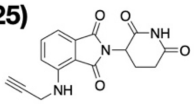

A28_220_13_7_2021 : Injection 1

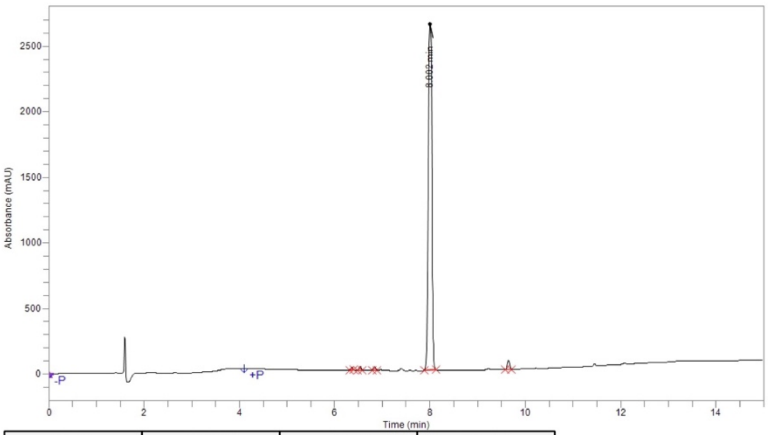

Time	Height	Area	Area %
6.357	21,251.7	61,668.2	0.45
6.531	27,158.9	83,361.4	0.61
6.838	28,107.5	86,355.7	0.63
8.002	2,641,521.8	13,311,843.4	96.69
9.649	69,316.8	224,418.0	1.63
**Total**		13,767,646.8	100.00

A28_254_13_7_2021 : Injection 1

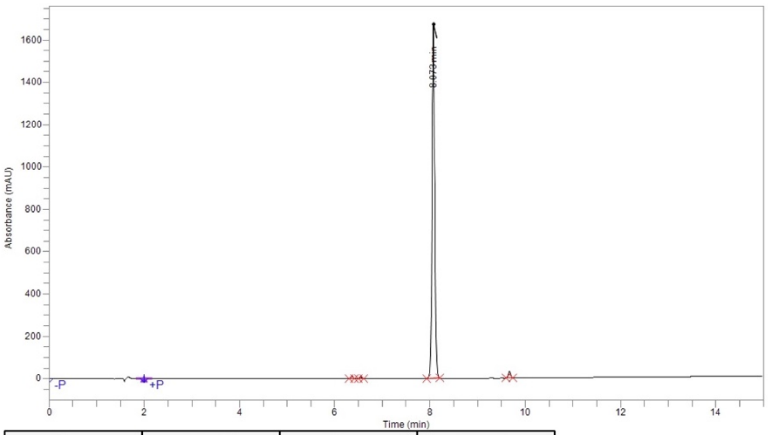

Time	Height	Area	Area %
6.365	10,841.5	35,164.2	0.49
6.547	8,636.8	27,331.6	0.38
8.073	1,674,556.2	7,007,741.9	97.68
9.670	31,892.5	103,799.2	1.45
**Total**		7,174,036.9	100.00

4-(Benzylamino)-2-(2,6-dioxopiperidin-3-yl)isoindoline-1,3-dione **(26)**

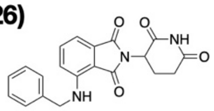

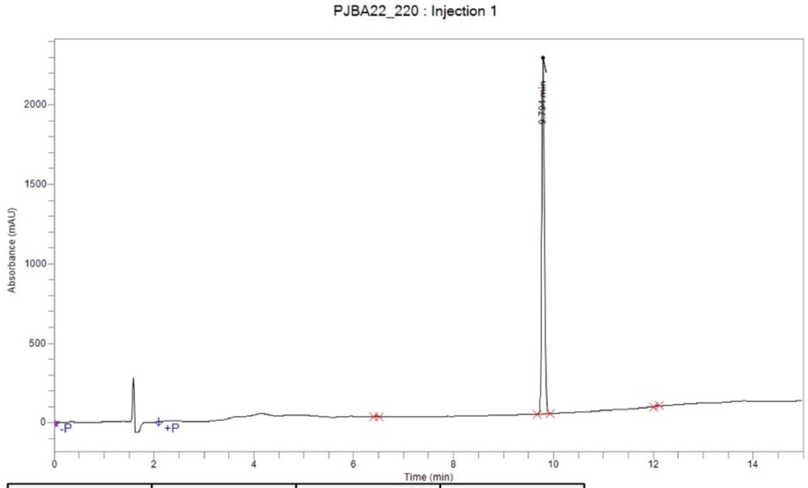

Time	Height	Area	Area %
6.453	5,600.9	19,763.4	0.22
9.794	2,243,243.4	8,880,223.9	99.53
12.077	5,477.1	22,020.7	0.25
**Total**		8,922,008.0	100.00

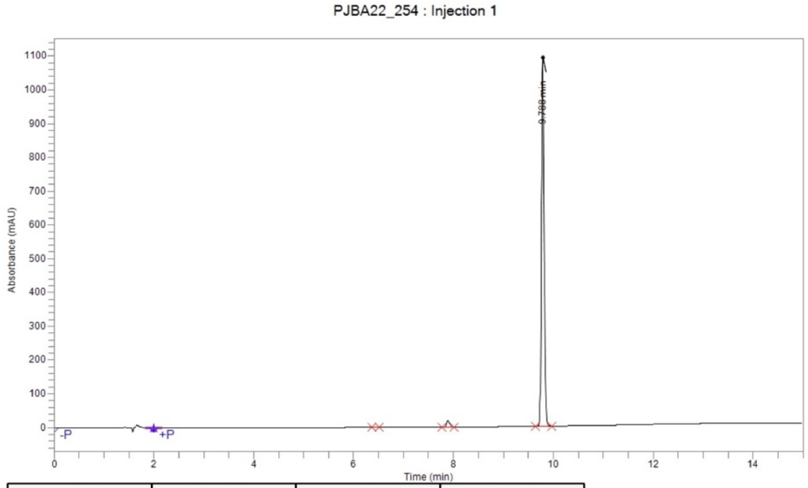

Time	Height	Area	Area %
6.444	1,859.3	7,084.6	0.16
7.894	19,164.5	93,740.9	2.17
9.788	1,093,396.3	4,221,720.4	97.67
**Total**		4,322,546.0	100.00

2-(2,6-Dioxopiperidin-3-yl)-4-((pyridin-3-ylmethyl)amino)isoindoline-1,3-dione **(27)**

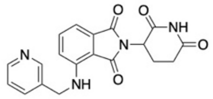

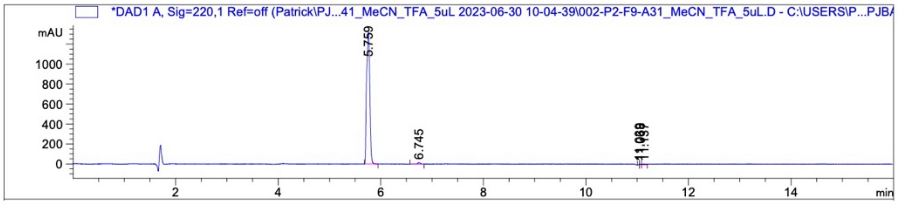

Peak #	RetTime [min]	Type	Width [min]	Area [mAU*s]	Height [mAU]	Area %
1	5.759	BB	0.0630	5757.53711	1316.86975	97.6576
2	6.745	VV R	0.0474	57.94011	14.87543	0.9828
3	11.039	BV	0.0169	10.67051	8.06211	0.1810
4	11.068	VV	0.0292	21.24766	9.12582	0.3604
5	11.137	VV	0.0710	48.23938	8.21837	0.8182

Totals : 5895.63477 1357.15148

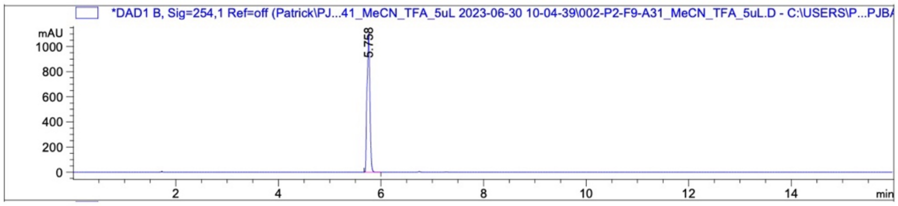

Peak #	RetTime [min]	Type	Width [min]	Area [mAU*s]	Height [mAU]	Area %
1	5.758	BB	0.0542	4278.00879	1104.08264	100.0000

Totals : 4278.00879 1104.08264

2-(2,6-Dioxopiperidin-3-yl)-4-((2-methylbenzyl)amino)isoindoline-1,3-dione **(28)**

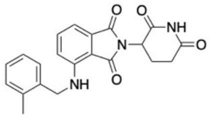

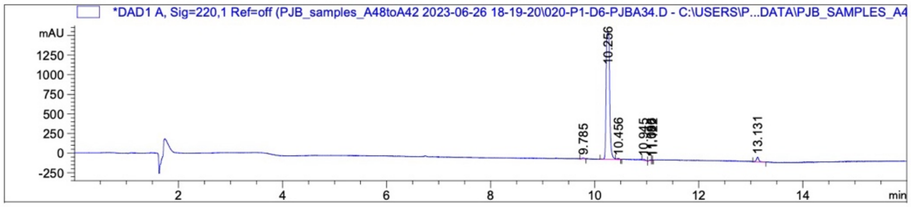

Peak #	RetTime [min]	Type	Width [min]	Area [mAU*s]	Height [mAU]	Area %
1	9.785	VV	0.0444	45.78736	12.75951	0.5763
2	10.256	BV R	0.0579	7577.73291	1609.09106	95.3733
3	10.456	VV E	0.0356	30.46284	10.73134	0.3834
4	10.945	VB	0.0530	46.46904	10.63425	0.5849
5	11.086	BV	0.0376	31.33801	10.12394	0.3944
6	11.096	VV	0.0175	9.68551	9.24094	0.1219
7	11.122	VV	0.0131	6.09629	7.05964	0.0767
8	13.131	BV R	0.0402	197.76942	61.97969	2.4891
Totals :				7945.34139	1731.62037	

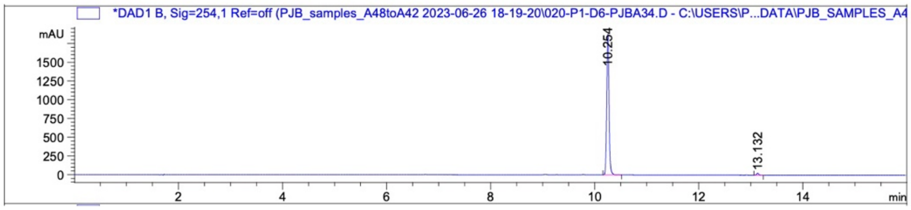

Peak #	RetTime [min]	Type	Width [min]	Area [mAU*s]	Height [mAU]	Area %
1	10.254	BV R	0.0481	5814.16699	1877.96265	98.7680
2	13.132	BB	0.0450	72.52560	24.90003	1.2320
Totals :				5886.69259	1902.86268	

2-(2,6-Dioxopiperidin-3-yl)-4-(((4-methylpyridin-3-yl)methyl)amino)isoindoline-1,3-dione **(29)**

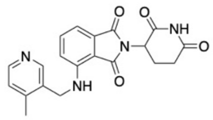

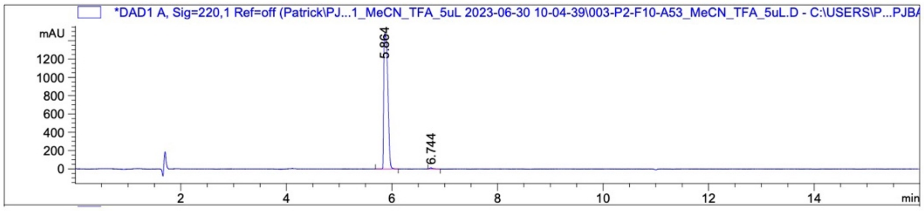

Peak #	RetTime [min]	Type	Width [min]	Area [mAU*s]	Height [mAU]	Area %
1	5.864	VB R	0.0630	7864.15283	1479.57996	99.4521
2	6.744	BB	0.0488	43.32656	10.80010	0.5479
Totals :				7907.47939	1490.38006	

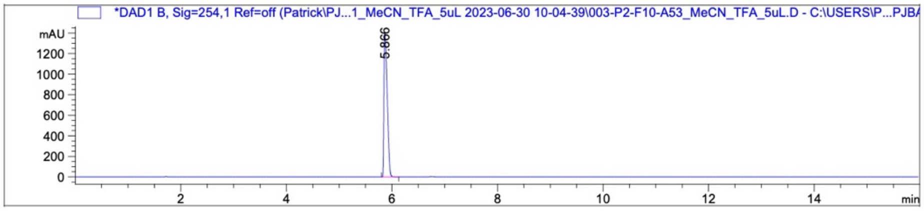

Peak #	RetTime [min]	Type	Width [min]	Area [mAU*s]	Height [mAU]	Area %
1	5.866	BB	0.0655	6021.60986	1392.17969	100.0000
Totals :				6021.60986	1392.17969	

4-(Benzyloxy)-2-(2,6-dioxopiperidin-3-yl)isoindoline-1,3-dione **(30)**

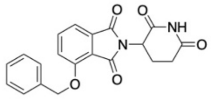

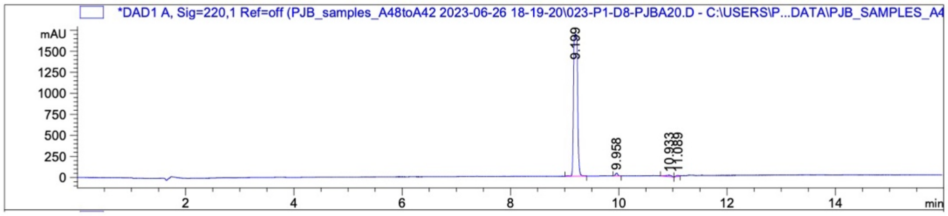

Peak #	RetTime [min]	Type	Width [min]	Area [mAU*s]	Height [mAU]	Area %
1	9.199	VV R	0.0616	8759.21680	1687.26782	96.7374
2	9.958	BB	0.0428	104.18281	33.90955	1.1506
3	10.933	BB	0.0947	129.00211	16.15368	1.4247
4	11.089	BV	0.0602	62.22880	12.37132	0.6873

Totals : 9054.63051 1749.70237

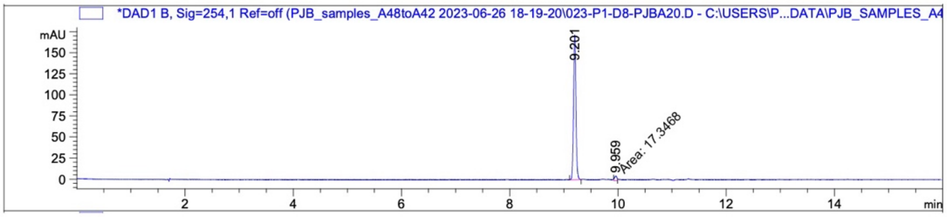

Peak #	RetTime [min]	Type	Width [min]	Area [mAU*s]	Height [mAU]	Area %
1	9.201	BB	0.0474	529.15289	171.89610	96.8258
2	9.959	MM	0.0531	17.34685	5.44532	3.1742

Totals : 546.49974 177.34143

2-(2,6-Dioxopiperidin-3-yl)-4-(pyridin-3-ylmethoxy)isoindoline-1,3-dione **(31)**

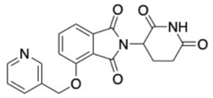

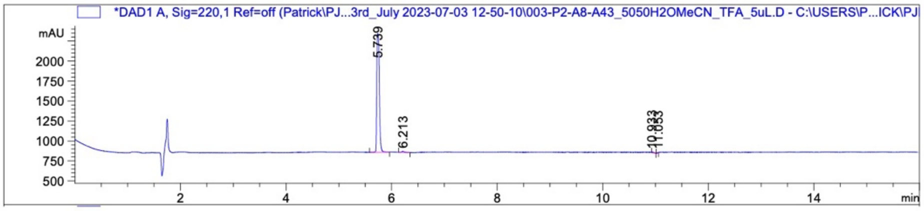

Peak #	RetTime [min]	Type	Width [min]	Area [mAU*s]	Height [mAU]	Area %
1	5.739	BB	0.0422	5070.66211	1490.58447	98.3312
2	6.213	VV R	0.0412	47.04042	14.54279	0.9122
3	10.933	VB	0.0443	27.20971	7.49671	0.5277
4	11.053	BV	0.0186	11.80451	8.07088	0.2289
Totals :				5156.71675	1520.69486	

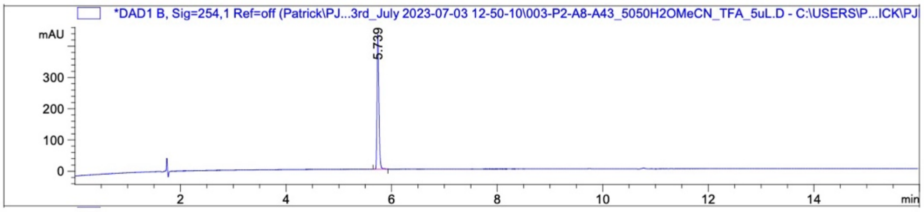

Peak #	RetTime [min]	Type	Width [min]	Area [mAU*s]	Height [mAU]	Area %
1	5.739	BV R	0.0364	1051.10193	431.90494	100.0000
Totals :				1051.10193	431.90494	

2-(2,6-Dioxopiperidin-3-yl)-4-((2-methylbenzyl)oxy)isoindoline-1,3-dione **(32)**

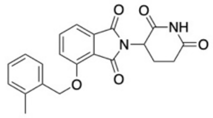

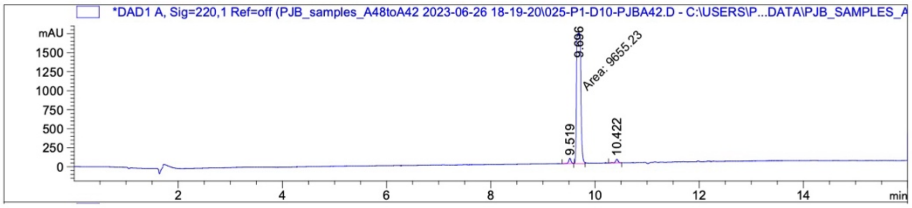

Peak #	RetTime [min]	Type	Width [min]	Area [mAU*s]	Height [mAU]	Area %
1	9.519	BV	0.0462	220.06575	71.82719	2.1918
2	9.696	MM	0.0935	9655.23340	1721.78210	96.1649
3	10.422	VB R	0.0463	164.99365	49.65711	1.6433

Totals : 1.00403e4 1843.26640

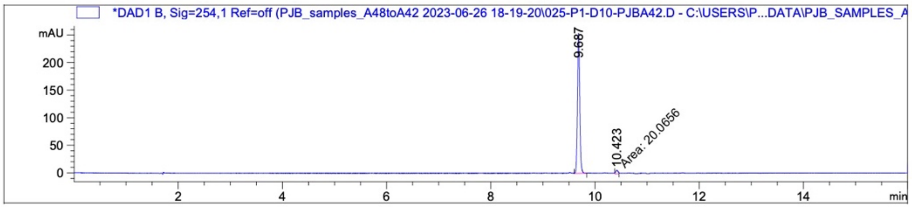

Peak #	RetTime [min]	Type	Width [min]	Area [mAU*s]	Height [mAU]	Area %
1	9.687	BB	0.0462	766.29730	253.89758	97.4483
2	10.423	MM	0.0522	20.06562	6.41060	2.5517

Totals : 786.36293 260.30818

4-(Benzhydrylamino)-2-(2,6-dioxopiperidin-3-yl)isoindoline-1,3-dione **(33)**

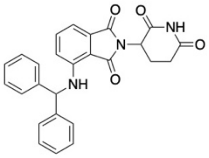

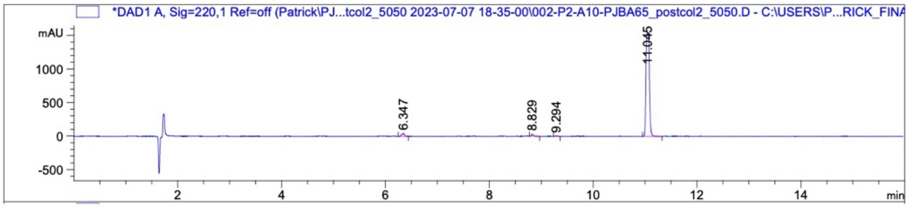

Peak #	RetTime [min]	Type	Width [min]	Area [mAU*s]	Height [mAU]	Area %
1	6.347	BB	0.0499	170.95700	46.51646	2.3214
2	8.829	BB	0.0469	96.15903	30.37664	1.3057
3	9.294	BB	0.0451	28.13963	9.34437	0.3821
4	11.045	BB	0.0635	7069.25488	1541.79138	95.9908

Totals : 7364.51055 1628.02885

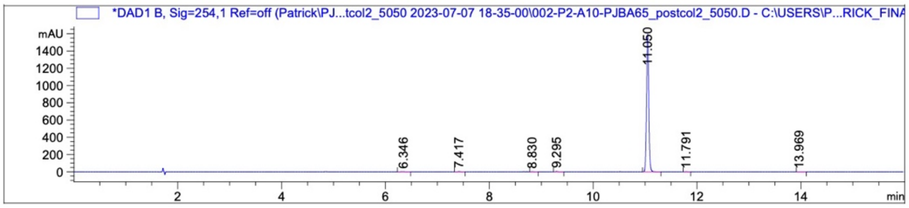

Peak #	RetTime [min]	Type	Width [min]	Area [mAU*s]	Height [mAU]	Area %
1	6.346	BB	0.0537	8.49456	2.12141	0.1766
2	7.417	BB	0.0469	9.34295	3.12326	0.1943
3	8.830	BB	0.0450	3.33332	1.05111	0.0693
4	9.295	BB	0.0453	11.41339	3.77203	0.2373
5	11.050	BB	0.0458	4765.96533	1597.53052	99.1084
6	11.791	BB	0.0543	3.98341	9.63068e-1	0.0828
7	13.969	BB	0.0718	6.30632	1.08459	0.1311

Totals : 4808.83929 1609.64599

4-(Benzhydryloxy)-2-(2,6-dioxopiperidin-3-yl)isoindoline-1,3-dione **(34)**

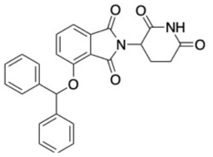

A83_220_14_7_2021 : Injection 1

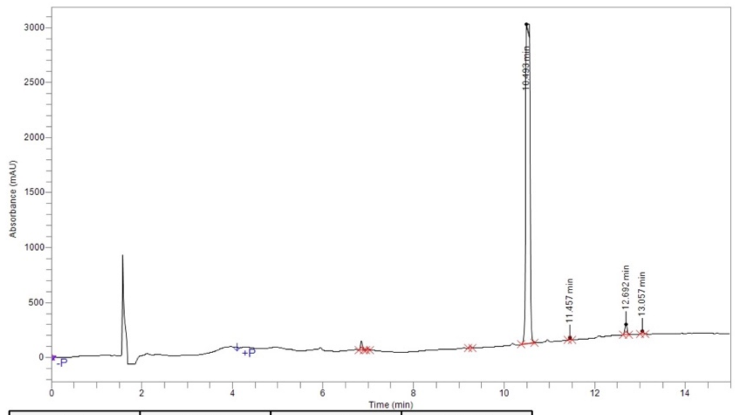

Time	Height	Area	Area %
6.850	84,966.7	237,594.8	1.19
6.994	19,324.9	50,593.3	0.25
9.255	18,338.3	54,587.1	0.27
10.493	2,942,967.8	19,196,493.1	95.94
11.457	20,516.2	58,680.1	0.29
12.692	95,320.7	316,917.6	1.58
13.057	27,577.0	93,787.6	0.47
**Total**		20,008,653.7	100.00

A83_254_14_7_2021 : Injection 1

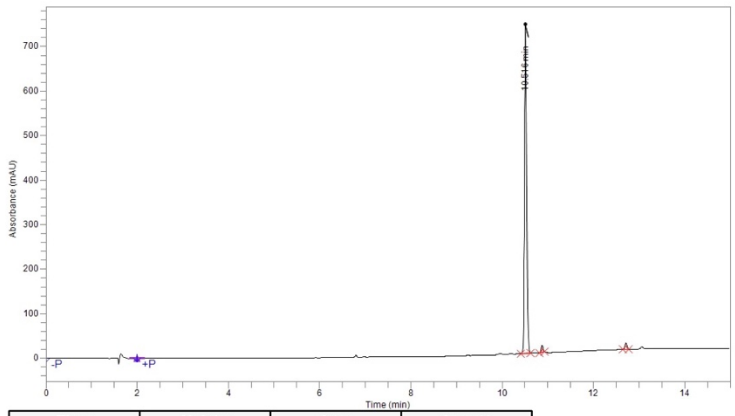

Time	Height	Area	Area %
10.516	739,977.7	2,443,080.6	96.27
10.876	15,065.8	48,078.3	1.89
12.714	14,528.5	46,657.1	1.84
**Total**		2,537,816.0	100.00

6-(2,6-Dioxopiperidin-3-yl)-5H-pyrrolo[3,4-b]pyridine-5,7(6H)-dione **(35)**

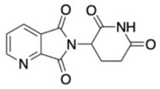

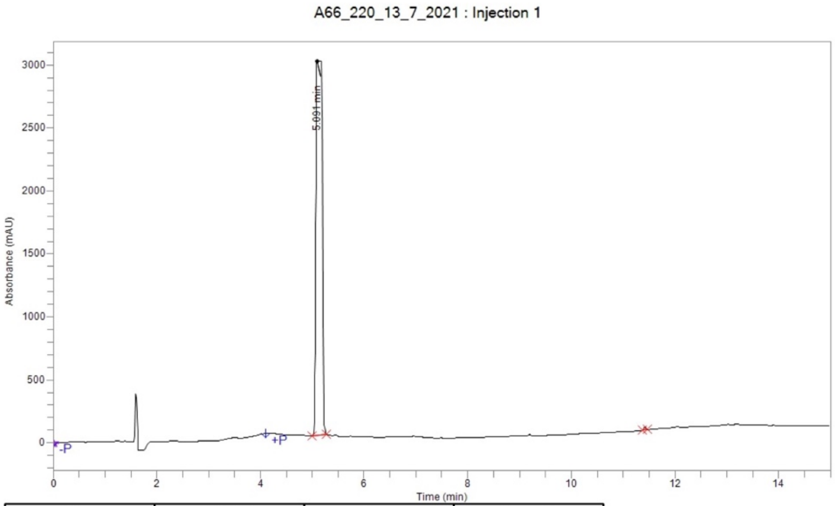

Time	Height	Area	Area %
5.091	3,000,256.3	24,431,285.3	99.74
11.418	22,738.6	64,527.0	0.26
**Total**		24,495,812.2	100.00

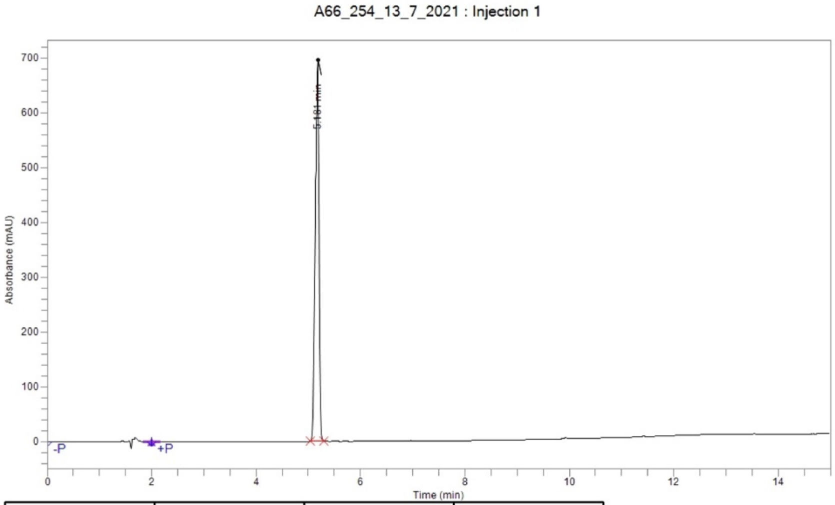

Time	Height	Area	Area %
5.181	697,023.0	3,671,621.6	100.00
**Total**		3,671,621.6	100.00

2-(2,6-Dioxopiperidin-3-yl)-1H-pyrrolo[3,4-c]pyridine-1,3(2H)-dione **(36)**

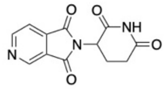

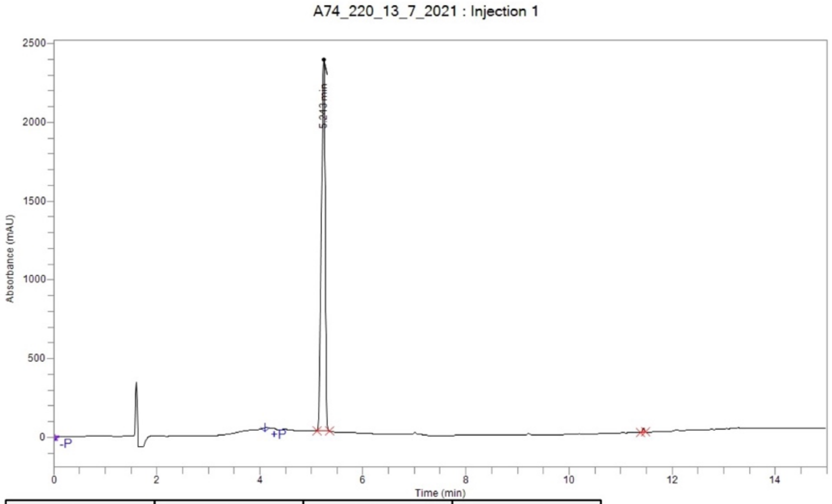

Time	Height	Area	Area %
5.243	2,361,847.5	12,178,523.5	99.41
11.440	23,934.0	72,061.0	0.59
**Total**		12,250,584.5	100.00

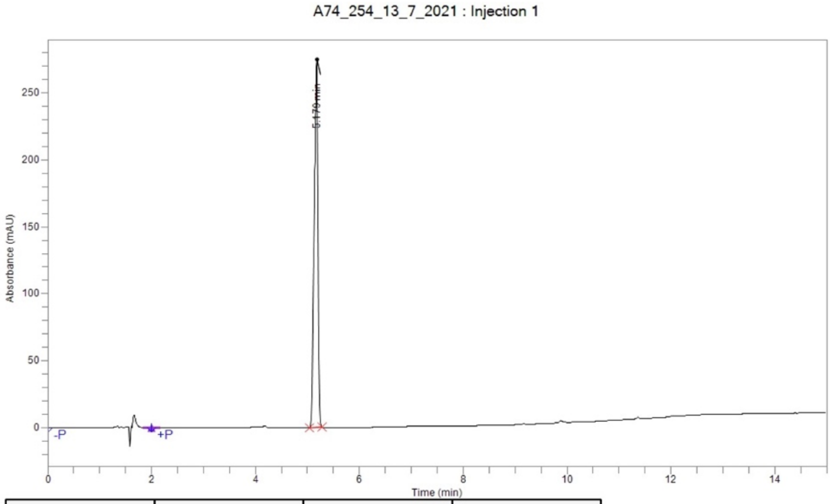

Time	Height	Area	Area %
5.179	275,514.1	1,450,941.8	100.00
**Total**		1,450,941.8	100.00

HPLC Traces for Compound **23** Stability Assay

Representative traces from one of three biological repeats; indicated peak contained compound **23** mass of 356.15.

0.5 h

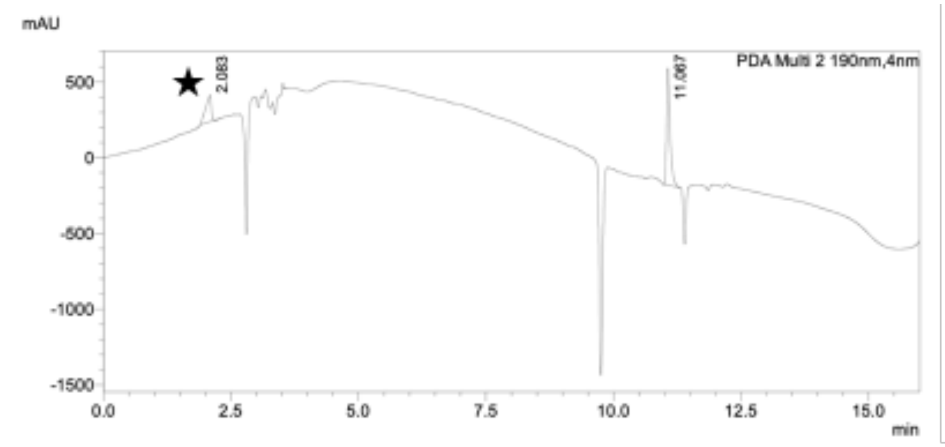

PDA Ch2 190nm

Peak#	Ret. Time	Area	Height	Conc.	Unit	Mark	Name
★ 1	2.083	1324242	175304	0.000		M	
2	11.067	3743080	765941	0.000		M	
Total		5067321	941245				

6 h

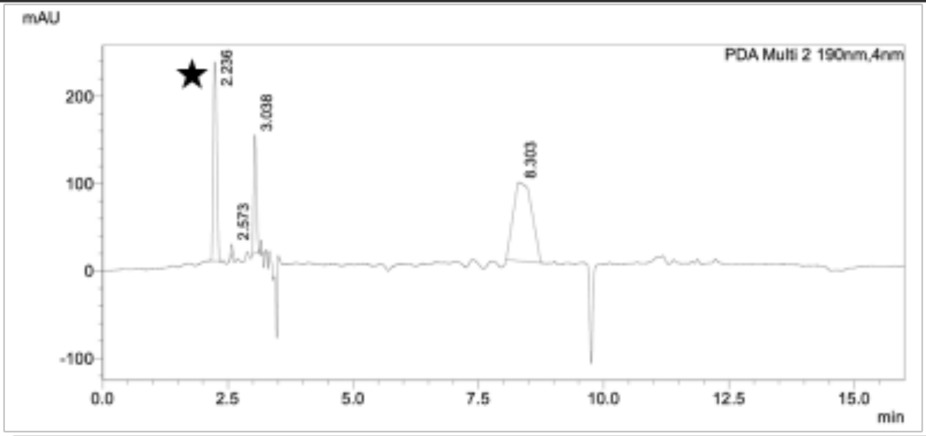

PDA Ch2 190nm

Peak#	Ret. Time	Area	Height	Conc.	Unit	Mark	Name
★ 1	2.236	1074303	226387	0.000		M	
2	2.573	27201	13100	0.000		M	
3	3.038	486892	134518	0.000		M	
4	8.303	2322038	89974	0.000		M	
Total		3910433	463979				

24 h

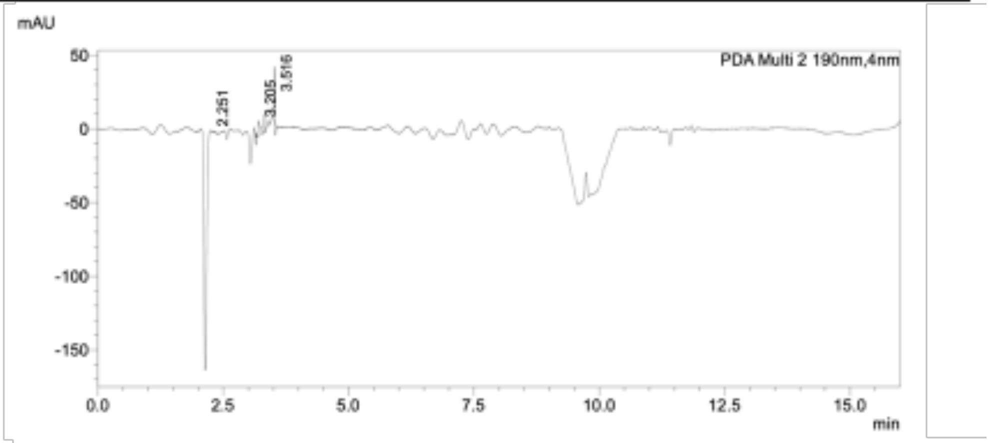

PDA Ch2 190nm

Peak#	Ret. Time	Area	Height	Conc.	Unit	Mark	Name
1	2.251	3729	414	0.000		M	
2	3.205	31848	9897	0.000		M	
3	3.516	39348	35098	0.000		M	
Total		74925	45409				

REFERENCES

(1) Fulmer, G. R.; Miller, A. J. M.; Sherden, N. H.; Gottlieb, H. E.; Nudelman, A.; Stoltz, B. M.; Bercaw, J. E.; Goldberg, K. I. NMR Chemical Shifts of Trace Impurities: Common Laboratory Solvents, Organics, and Gases in Deuterated Solvents Relevant to the Organometallic Chemist. *Organometallics* **2010**, *29* (9), 2176–2179. https://doi.org/10.1021/OM100106E/SUPPL_FILE/OM100106E_SI_001.PDF.

(2) Vu, B. D.; Ba, N. M. H.; Phan, D. C. Facile Synthesis of Thalidomide. *Org. Process Res. Rev.* **2019**, *23* (7), 1374–1377. https://doi.org/10.1021/ACS.OPRD.9B00122/SUPPL_FILE/OP9B00122_SI_001.PDF.

(3) De, A. U.; Pal, D. Possible Antineoplastic Agents I. *J. Pharm. Sci.* **1975**, *64* (2), 262–266. https://doi.org/10.1002/jps.2600640214.

(4) Nguyen, T. D.; Ba, N. M. H.; Phan, D. C.; Vu, B. D. Microwave Assisted Synthesis of Thalidomide on Hectogram Scale. *Org. Prep. Proced. Int.* **2022**, *54* (3), 294–298. https://doi.org/10.1080/00304948.2021.2024681.

(5) Hao, E.; Su, F.; Li, G.; Liu, Y.; Zhang, Q.; Zhang, M. Preparation Method of Anti-Tumor Therapeutic Medicine Pomalyst; CN109553603A, 2019.

(6) Zhang, Z.; Deng, Y.; Hou, M.; Lai, X.; Guan, M.; Zhang, F.; Qi, R.; Qiu, G. Iron/Photoredox Dual Catalysis for Acyl Nitrene-Based C–O Bond Formation towards Phthalides. *Chem. Commun.* **2022**, *58* (98), 13644–13647. https://doi.org/10.1039/D2CC04917A.

(7) Sharma, C.; Choi, M. A.; Song, Y.; Seo, Y. H. Rational Design and Synthesis of HSF1-PROTACs for Anticancer Drug Development. *Molecules* **2022**, *27* (5), 1655. https://doi.org/10.3390/MOLECULES27051655.

(8) Muller, G. W.; Stirling, D. I.; Chen, R. S.-C. Substituted 2(2,6-Dioxopiperidin-3-Yl)Isoindolines; US6335349, 2002.

(9) Luo, W.; Yu, Q. S.; Tweedie, D.; Deschamps, J.; Parrish, D.; Holloway, H. W.; Li, Y.; Brossi, A.; Greig, N. H. Syntheses of Aromatic Substituted-Thiothalidomides. *Synthesis* **2008**, *2008* (21), 3415–3422. https://doi.org/10.1055/S-0028-1083179.

(10) Bricelj, A.; Ng, Y. L. D.; Ferber, D.; Kuchta, R.; Müller, S.; Monschke, M.; Wagner, K. G.; Krönke, J.; Sosič, I.; Gütschow, M.; Steinebach, C. Influence of Linker Attachment Points on the Stability and Neosubstrate Degradation of Cereblon Ligands. *ACS Med. Chem. Lett.* **2021**, *12* (11), 1733–1738. https://doi.org/10.1021/ACSMEDCHEMLETT.1C00368/SUPPL_FILE/ML1C00368_SI_001.PDF.

(11) Wei, M.; Zhao, R.; Cao, Y.; Wei, Y.; Li, M.; Dong, Z.; Liu, Y.; Ruan, H.; Li, Y.; Cao, S.; Tang, Z.; Zhou, Y.; Song, W.; Wang, Y.; Wang, J.; Yang, G.; Yang, C. First Orally Bioavailable Prodrug of Proteolysis Targeting Chimera (PROTAC) Degrades Cyclin-Dependent Kinases 2/4/6 in Vivo. *Eur. J. Med. Chem.* **2021**, *209*, 112903. https://doi.org/10.1016/J.EJMECH.2020.112903.

(12) Nakamura, T.; Noguchi, T.; Kobayashi, H.; Miyachi, H.; Hashimoto, Y. Mono- and Dihydroxylated Metabolites of Thalidomide: Synthesis and TNF-Alpha Production-Inhibitory Activity. *Chem. Pharm. Bull.* **2006**, *54* (12), 1709–1714. https://doi.org/10.1248/CPB.54.1709.

(13) Robb, C. M.; Contreras, J. I.; Kour, S.; Taylor, M. A.; Abid, M.; Sonawane, Y. A.; Zahid, M.; Murry, D. J.; Natarajan, A.; Rana, S. Chemically Induced Degradation of CDK9 by a Proteolysis Targeting Chimera (PROTAC). *Chem. Commun.* **2017**, *53* (54), 7577–7580. https://doi.org/10.1039/C7CC03879H.

(14) Ménard, M.; Erichomovitch, L.; Brooy, M. L.; Chubb, F. L. QUELQUES METABOLITES POSSIBLES DE LA THALIDOMIDE. *Can. J. Chem.* **2011**, *41* (7), 1722–1725. https://doi.org/10.1139/V63-247.

(15) Burslem, G. M.; Ottis, P.; Jaime-Figueroa, S.; Morgan, A.; Cromm, P. M.; Toure, M.; Crews, C. M. Efficient Synthesis of Immunomodulatory Drug Analogues Enables Exploration of Structure-Degradation Relationships. *ChemMedChem* **2018**, *13* (15), 1508–1512. https://doi.org/10.1002/CMDC.201800271.

(16) Man, H.-W.; Muller, G. W. 1-Oxo- and 1,3-Dioxoisoindolines and Method of Reducing Inflammatory Cytokine Levels; US2001006973A1, 2001.

(17) Ghose, D. Possible Antineoplastic Agents: III. Synthesis of 6-Alkyl-2-[4' Methoxyphthalimido] and 6-Alkyl-3-[3'-4'-Dimethoxyphenyl] Glutarimides. *J. Indian Chem. Soc.* **1976**, *53* (11), 1122.

(18) Tseng, Y. L.; Lu, P. C.; Lee, C. C.; He, R. Y.; Huang, Y. A.; Tseng, Y. C.; Cheng, T. J. R.; Huang, J. J. T.; Fang, J. M. Degradation of Neurodegenerative Disease-Associated TDP-43 Aggregates and Oligomers via a Proteolysis-Targeting Chimera. *J. Biomed. Sci.* **2023**, *30* (1), 1–21. https://doi.org/10.1186/S12929-023-00921-7/FIGURES/6.

(19) Wang, Y.; Zhou, Y.; Cao, S.; Sun, Y.; Dong, Z.; Li, C.; Wang, H.; Yao, Y.; Yu, H.; Song, X.; Li, M.; Wang, J.; Wei, M.; Yang, G.; Yang, C. In Vitro and in Vivo Degradation of Programmed Cell Death Ligand 1 (PD-L1) by a Proteolysis Targeting Chimera (PROTAC). *Bioorg. Chem.* **2021**, *111*, 104833. https://doi.org/10.1016/J.BIOORG.2021.104833.

(20) Zhou, B.; Hu, J.; Xu, F.; Chen, Z.; Bai, L.; Fernandez-Salas, E.; Lin, M.; Liu, L.; Yang, C.-Y.; Zhao, Y.; Mceachern, D.; Przybranowski, S.; Wen, B.; Sun, D.; Wang, S. Discovery of a Small-Molecule Degrader of Bromodomain and Extra-Terminal (BET) Proteins with Picomolar Cellular Potencies and Capable of Achieving Tumor Regression. *J. Med. Chem.* **2017**, *61*, 462–481. https://doi.org/10.1021/acs.jmedchem.6b01816.

(21) Vannam, R.; Sayilgan, J.; Ojeda, S.; Karakyriakou, B.; Hu, E.; Kreuzer, J.; Morris, R.; Lopez, X. I. H.; Rai, S.; Haas, W.; Lawrence, M.; Ott, C. J. Targeted Degradation of the Enhancer Lysine Acetyltransferases CBP and P300. *Cell Chem. Biol.* **2021**, *28* (4), 503–514. https://doi.org/10.1016/J.CHEMBIOL.2020.12.004.

(22) Hanafi, M.; Chen, X.; Neamati, N. Discovery of a Napabucasin PROTAC as an Effective Degrader of the E3 Ligase ZFP91. *J. Med. Chem.* **2021**, *64* (3), 1626–1648. https://doi.org/10.1021/ACS.JMEDCHEM.0C01897/SUPPL_FILE/JM0C01897_SI_002.CSV.

(23) Stewart, S. G.; Braun, C. J.; Ng, S. L.; Polomska, M. E.; Karimi, M.; Abraham, L. J. New Thalidomide Analogues Derived through Sonogashira or Suzuki Reactions and Their TNF Expression Inhibition Profiles. *Bioorgan. Med. Chem.* **2010**, *18* (2), 650–662. https://doi.org/10.1016/J.BMC.2009.12.001.

(24) Kloster-Jensen, E. Synthesis of 4-Bromophthalyl-D,L-Glutamic Imide. *Acta Chem. Scand.* **1964**, *19*, 266–267.

(25) Kawai, H.; Furukawa, T.; Nomura, Y.; Tokunaga, E.; Shibata, N. Cu-Mediated Chemoselective Trifluoromethylation of Benzyl Bromides Using Shelf-Stable Electrophilic Trifluoromethylating Reagents. *Org. Lett.* **2011**, *13* (14), 3596–3599. https://doi.org/10.1021/OL201205T/SUPPL_FILE/OL201205T_SI_001.PDF.

(26) Kampmann, S. S.; Skelton, B. W.; Yeoh, G. C.; Abraham, L. J.; Lengkeek, N. A.; Stubbs, K. A.; Heath, C. H.; Stewart, S. G. The Synthesis and Fluorescence Profile of Novel Thalidomide Analogues. *Tetrahedron* **2015**, *71* (42), 8140–8149. https://doi.org/10.1016/J.TET.2015.08.036.

(27) Yang, K.; Song, Y.; Xie, H.; Wu, H.; Wu, Y. T.; Leisten, E. D.; Tang, W. Development of the First Small Molecule Histone Deacetylase 6 (HDAC6) Degraders. *Bioorgan. Med. Chem. Lett.* **2018**, *28* (14), 2493–2497. https://doi.org/10.1016/J.BMCL.2018.05.057.

(28) Robarge, M. J.; Chen, R. S.-C.; Muller, G. W.; Man, H. W. Isoindole-Imide Compounds, Compositions, and Uses Thereof; US2003045552A1, 2003.

(29) Brownsey, D. K.; Rowley, B. C.; Gorobets, E.; Gelfand, B. S.; Derksen, D. J. Rapid Synthesis of Pomalidomide-Conjugates for the Development of Protein Degrader Libraries. *Chem. Sci.* **2021**, *12* (12), 4519–4525. https://doi.org/10.1039/D0SC05442A.

(30) Ruchelman, A. L.; Muller, G. W.; Man, H.-W. 4’-O-SUBSTITUTED ISOINDOLINE DERIVATIVES AND COMPOSITIONS COMPOSITIONS COMPRISING AND METHODS OF USING THE SAME; WO2008115516A2, 2008.

(31) Fickentscher, K. Synthese von N-Chinolyl-Glutaminsäureimid. *Arch. Pharm.* **1974**, *307* (6), 473–476. https://doi.org/10.1002/ARDP.19743070614.

(32) Luo, W.; Yu, Q. S.; Salcedo, I.; Holloway, H. W.; Lahiri, D. K.; Brossi, A.; Tweedie, D.; Greig, N. H. Design, Synthesis and Biological Assessment of Novel N-Substituted 3-(Phthalimidin-2-Yl)-2,6-Dioxopiperidines and 3-Substituted 2,6-Dioxopiperidines for TNF-α Inhibitory Activity. *Bioorgan. Med. Chem.* **2011**, *19* (13), 3965–3972. https://doi.org/10.1016/J.BMC.2011.05.029.

(33) Greig, N. H.; Holloway, H.; Brossi, A.; Zhu, X.; Giordano, T.; Yu, Q.-S. THALIDOMIDE ANALOGS; WO2005028436A2, 2005.

(34) Robinson, M. W.; Hill, A. P.; Readshaw, S. A.; Hollerton, J. C.; Upton, R. J.; Lynn, S. M.; Besley, S. C.; Boughtflower, B. J. Use of Calculated Physicochemical Properties to Enhance Quantitative Response When Using Charged Aerosol Detection. *Anal. Chem.* **2017**, *89* (3), 1772–1777. https://doi.org/10.1021/ACS.ANALCHEM.6B04060.

(35) Rognes, T.; Flouri, T.; Nichols, B.; Quince, C.; Mahé, F. VSEARCH: A Versatile Open Source Tool for Metagenomics. *PeerJ* **2016**, *4* (10), 2584. https://doi.org/10.7717/PEERJ.2584.

(36) Law, C. W.; Chen, Y.; Shi, W.; Smyth, G. K. Voom: Precision Weights Unlock Linear Model Analysis Tools for RNA-Seq Read Counts. *Genome Biol.* **2014**, *15* (2), 1–17. https://doi.org/10.1186/GB-2014-15-2-R29/FIGURES/11.

(37) Synthego - CRISPR Performance Analysis. https://ice.synthego.com/#/.

(38) O'Connor, L. J.; Cazares-Körner, C.; Saha, J.; Evans, C. N. G.; Stratford, M. R. L.; Hammond, E. M.; Conway, S. J. Design, Synthesis and Evaluation of Molecularly Targeted Hypoxia-Activated Prodrugs. *Nat Protoc* **2016**, *11* (4), 781–794. https://doi.org/10.1038/nprot.2016.034.

(39) Skwarska, A.; Calder, E. D. D.; Sneddon, D.; Bolland, H.; Odyniec, M. L.; Mistry, I. N.; Martin, J.; Folkes, L. K.; Conway, S. J.; Hammond, E. M. Development and Pre-Clinical Testing of a Novel Hypoxia-Activated KDAC Inhibitor. *Cell Chemical Biology* **2021**, *28* (9), 1258-1270.e13. https://doi.org/10.1016/j.chembiol.2021.04.004.

(40) Wang, Y.; Yang, F.; Gritsenko, M. A.; Wang, Y.; Clauss, T.; Liu, T.; Shen, Y.; Monroe, M. E.; Lopez-Ferrer, D.; Reno, T.; Moore, R. J.; Klemke, R. L.; Camp, D. G.; Smith, R. D. Reversed-Phase Chromatography with Multiple Fraction Concatenation Strategy for Proteome Profiling of Human MCF10A Cells. *Proteomics* **2011**, *11* (10), 2019–2026. https://doi.org/10.1002/pmic.201000722.

(41) Paulo, J. A.; O'Connell, J. D.; Everley, R. A.; O'Brien, J.; Gygi, M. A.; Gygi, S. P. Quantitative Mass Spectrometry-Based Multiplexing Compares the Abundance of 5000 S. Cerevisiae Proteins across 10 Carbon Sources. *J. Proteomics* **2016**, *148*, 85–93. https://doi.org/10.1016/j.jprot.2016.07.005.

(42) McAlister, G. C.; Nusinow, D. P.; Jedrychowski, M. P.; Wühr, M.; Huttlin, E. L.; Erickson, B. K.; Rad, R.; Haas, W.; Gygi, S. P. MultiNotch MS3 Enables Accurate, Sensitive, and Multiplexed Detection of Differential Expression across Cancer Cell Line Proteomes. *Anal. Chem.* **2014**, *86* (14), 7150–7158. https://doi.org/10.1021/ac502040v.

(43) Paulo, J. A.; O'Connell, J. D.; Gygi, S. P. A Triple Knockout (TKO) Proteomics Standard for Diagnosing Ion Interference in Isobaric Labeling Experiments. *J. Am. Soc. Mass. Spectrom.* **2016**, *27* (10), 1620–1625. https://doi.org/10.1007/s13361-016-1434-9.

(44) Schweppe, D. K.; Prasad, S.; Belford, M. W.; Navarrete-Perea, J.; Bailey, D. J.; Huguet, R.; Jedrychowski, M. P.; Rad, R.; McAlister, G.; Abbatiello, S. E.; Woulters, E. R.; Zabrouskov,

V.; Dunyach, J.-J.; Paulo, J. A.; Gygi, S. P. Characterization and Optimization of Multiplexed Quantitative Analyses Using High-Field Asymmetric-Waveform Ion Mobility Mass Spectrometry. *Anal Chem* **2019**, *91* (6), 4010–4016. https://doi.org/10.1021/acs.analchem.8b05399.

(45) Erickson, B. K.; Mintseris, J.; Schweppe, D. K.; Navarrete-Perea, J.; Erickson, A. R.; Nusinow, D. P.; Paulo, J. A.; Gygi, S. P. Active Instrument Engagement Combined with a Real-Time Database Search for Improved Performance of Sample Multiplexing Workflows. *J. Proteome. Res.* **2019**, *18* (3), 1299–1306. https://doi.org/10.1021/acs.jproteome.8b00899.

(46) Schweppe, D. K.; Eng, J. K.; Yu, Q.; Bailey, D.; Rad, R.; Navarrete-Perea, J.; Huttlin, E. L.; Erickson, B. K.; Paulo, J. A.; Gygi, S. P. Full-Featured, Real-Time Database Searching Platform Enables Fast and Accurate Multiplexed Quantitative Proteomics. *J. Proteome. Res.* **2020**, *19* (5), 2026–2034. https://doi.org/10.1021/acs.jproteome.9b00860.

(47) Rad, R.; Li, J.; Mintseris, J.; O'Connell, J.; Gygi, S. P.; Schweppe, D. K. Improved Monoisotopic Mass Estimation for Deeper Proteome Coverage. *J. Proteome. Res.* **2021**, *20* (1), 591–598. https://doi.org/10.1021/acs.jproteome.0c00563.

(48) Elias, J. E.; Gygi, S. P. Target-Decoy Search Strategy for Increased Confidence in Large-Scale Protein Identifications by Mass Spectrometry. *Nat. Methods* **2007**, *4* (3), 207–214. https://doi.org/10.1038/nmeth1019.

(49) Huttlin, E. L.; Jedrychowski, M. P.; Elias, J. E.; Goswami, T.; Rad, R.; Beausoleil, S. A.; Villén, J.; Haas, W.; Sowa, M. E.; Gygi, S. P. A Tissue-Specific Atlas of Mouse Protein Phosphorylation and Expression. *Cell* **2010**, *143* (7), 1174–1189. https://doi.org/10.1016/j.cell.2010.12.001.

(50) Savitski, M. M.; Wilhelm, M.; Hahne, H.; Kuster, B.; Bantscheff, M. A Scalable Approach for Protein False Discovery Rate Estimation in Large Proteomic Data Sets. *Mol. Cell. Proteomics.* **2015**, *14* (9), 2394–2404. https://doi.org/10.1074/mcp.M114.046995.

(51) Tyanova, S.; Temu, T.; Sinitcyn, P.; Carlson, A.; Hein, M. Y.; Geiger, T.; Mann, M.; Cox, J. The Perseus Computational Platform for Comprehensive Analysis of (Prote)Omics Data. *Nat. Methods.* **2016**, *13* (9), 731–740. https://doi.org/10.1038/nmeth.3901.

(52) Sievers, Q. L.; Petzold, G.; Bunker, R. D.; Renneville, A.; Słabicki, M.; Liddicoat, B. J.; Abdulrahman, W.; Mikkelsen, T.; Ebert, B. L.; Thomä, N. H. Defining the Human C2H2 Zinc Finger Degrome Targeted by Thalidomide Analogs through CRBN. *Science* **2018**, *362* (6414), eaat0572. https://doi.org/10.1126/SCIENCE.AAT0572.
